# PAMP orchestrates proline metabolic rewiring to suppress LUAD via PYCR1 inhibition

**DOI:** 10.1038/s44321-026-00460-2

**Published:** 2026-06-09

**Authors:** Yun Ma, Jiani Yi, Chengcai Zheng, Juze Yang, Jia Li, Mengqian Yu, Xinyi Qian, Jiayi Ren, Xiaofan Wu, Yan Lu, Pengyuan Liu

**Affiliations:** 1 Department of Respiratory Medicine, Sir Run Run Shaw Hospital and Institute of Translational Medicine, Zhejiang University School of Medicine, Hangzhou, Zhejiang 310016 China; 2 Zhejiang Provincial Key Laboratory of Precision Diagnosis and Therapy for Major Gynecological Diseases, Women’s Hospital and Institute of Translational Medicine, Zhejiang University School of Medicine, Hangzhou, Zhejiang 310006 China; 3 Laboratory of Frontier Medical Research on Cancer Metabolism, Zhejiang University School of Medicine, Hangzhou, Zhejiang 310029 China

**Keywords:** Cancer, Metabolism, RNA Biology

## Abstract

Recent advances in next-generation sequencing have revealed that long non-coding RNAs (lncRNAs) can encode functional micropeptides through small open reading frames (sORFs), altering the perception of the non-coding genome. In this study, we identified a 48-amino acid micropeptide named PAMP (proline-associated micropeptide), encoded by the lncRNA PSMA3-AS1, as a novel tumor suppressor in lung adenocarcinoma (LUAD). PAMP is significantly downregulated in LUAD tissues and positively correlates with favorable prognosis. Functional assays demonstrated that PAMP inhibits LUAD cell proliferation in vitro and suppresses tumor growth in vivo. Mechanistically, PAMP directly interacts with PYCR1, a key enzyme in proline biosynthesis. Structural modeling and mutagenesis revealed that the PAMP-F16 and PYCR1-N123 residues are critical for the interaction, resulting in the inhibition of PYCR1 enzymatic activity and decreased proline accumulation. Notably, synthetic PAMP administration recapitulates these anti-tumor effects, effectively reducing intracellular proline levels and impairing tumor progression in cellular and animal models. Together, our findings uncover a previously uncharacterized lncRNA-encoded micropeptide that orchestrates proline metabolic reprogramming to restrain LUAD development, offering new opportunities for metabolic intervention in precision oncology.

## Synopsis

PSMA3-AS1 encodes a 48-aa micropeptide, PAMP, that binds and inhibits PYCR1 to suppress proline synthesis and LUAD proliferation; synthetic PAMP phenocopies these effects in cells and mice.

- PSMA3-AS1 encodes a 48-aa micropeptide (PAMP) that is downregulated in LUAD and associated with favorable prognosis.
- PAMP directly binds PYCR1 (PAMP-F16 ↔ PYCR1-N123), inhibits enzymatic activity, and reduces intracellular proline.
- Genetic gain/loss shows PAMP suppresses LUAD cell proliferation in vitro and limits tumor growth in vivo.
- Synthetic PAMP is cell-permeable, inhibits PYCR1 and proline accumulation, and suppresses tumor burden in mouse models-supporting a peptide-based metabolic intervention.

## Abstract (web summary)

PSMA3-AS1 encodes a 48-aa micropeptide, PAMP, that binds and inhibits PYCR1 to suppress proline synthesis and LUAD proliferation; synthetic PAMP phenocopies these effects in cells and mice.

## Graphical abstract

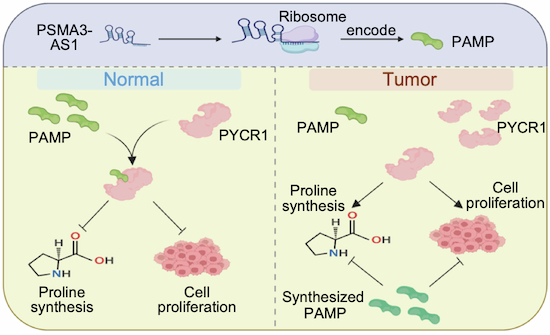

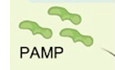

The paper explained **Problem**

Metabolic rewiring is a hallmark of LUAD, yet actionable vulnerabilities and therapeutic modalities beyond small molecules remain limited. Although lncRNAs can encode micropeptides, it remains unclear whether such peptides can directly target metabolic enzymes to restrain LUAD growth and be further developed as therapeutic agents.

**Results**

We identify PAMP, a 48-aa micropeptide encoded by PSMA3-AS1, as a tumor suppressor that is downregulated in LUAD and predicts better survival. PAMP binds PYCR1, the terminal enzyme in proline biosynthesis, and inhibits its activity, lowering intracellular proline. Structure-guided mutagenesis pinpoints a PAMP-F16/PYCR1-N123 interface critical for binding and function. Genetic perturbations show that PAMP suppresses LUAD proliferation in vitro and slows tumor growth in vivo. Importantly, synthetic PAMP is taken up by tumor cells, inhibits PYCR1/proline synthesis, and reduces tumor burden in mouse models.

**Impact**

These findings establish a lncRNA-encoded peptide and metabolic-enzyme regulatory axis that reprograms proline metabolism to constrain LUAD. By demonstrating that a synthetic micropeptide can directly inhibit PYCR1 and suppress tumor progression, this work expands therapeutic strategies for cancer metabolism beyond conventional small molecules and nominates PAMP–PYCR1 as a tractable precision-oncology target.

## Introduction

LUAD is the most prevalent form of lung cancer, accounting for ~35–40% of all cases worldwide (Leiter et al, 2023; Siegel et al, 2022). Despite recent advances in targeted therapy and immunotherapy, the overall survival rate for LUAD patients remains disappointingly low (Relli et al, 2019). Therefore, it is imperative to explore uncharted territories in search of novel molecular targets and therapeutic approaches that can improve clinical outcomes for LUAD patients.

lncRNAs, traditionally defined as transcripts longer than 200 nucleotides that do not code for proteins, have recently been shown to contain sORFs capable of encoding bioactive micropeptides (Herman et al, 2022; Ma et al, 2022; Wu et al, 2020). These micropeptides, typically composed of fewer than 100 amino acids, have emerged as important regulators of diverse cellular processes, including calcium homeostasis, metabolic reprogramming, embryonic development, and tumorigenesis (Huang et al, 2017; Zhang, 2024). For instance, the micropeptide AC115619–22aa inhibits hepatocellular carcinoma progression by binding WTAP and impeding the assembly of the *N*^6^-methyladenosine (m^6^A) methyltransferase complex, thereby modulating tumor-associated genes such as SOCS2 and ATG14 (Zhang et al, 2023). Similarly, RASON, a micropeptide encoded by LINC00673, directly binds KRAS, stabilizing it in its GTP-bound hyperactive state, which is essential for KRAS-driven tumorigenesis and tumor maintenance (Cheng et al, 2023a). These findings challenge the traditional view of lncRNAs as non-coding molecules, highlighting their dual functionality as both RNA regulators and sources of bioactive peptides.

Cancer cells are known to rewire their metabolism to meet the heightened demand for energy and biosynthetic precursors required for rapid proliferation (Kansara et al, 2023; Pavlova et al, 2022). These metabolic reprogramming includes enhanced synthesis of nucleic acids, proteins, and lipids, while maintaining redox and energy balance (Martinez-Reyes and Chandel, 2021). Recent studies have shown that lncRNA-encoded micropeptides can regulate key aspects of cancer metabolism, including glucose, lipid and amino acids metabolism, as well as mitochondrial function (Baena-Angulo et al, 2025; Wang et al, 2023). For example, the lncRNA-derived micropeptide XBP1SBM is upregulated in glutamine-deprived TNBC (triple-negative breast cancer) cells, and enhances glutamine uptake and promotes metastasis via the XBP1s/VEGF axis (Wu et al, 2022). Likewise, an ATP synthase–associated peptide (ASAP), encoded by LINC00467, enhances ATP synthase activity and promotes cancer cell proliferation by interacting with ATP synthase subunits ATP5A and ATP5C (Ge et al, 2021). Despite these advances, the roles of lncRNA-encode micropeptides in LUAD, particularly in the context of metabolic regulation, remain poorly understood.

In this study, we identified PSMA3-AS1, a highly conserved lncRNA that negatively regulates LUAD cell proliferation, and demonstrated that it encodes a bioactive micropeptide, which we named PAMP (proline-associated micropeptide). PAMP expression is significantly downregulated in LUAD tissues and is positively correlated with a favorable patient prognosis. Functional assays revealed that loss of PAMP markedly enhances LUAD cell proliferation, while its overexpression inhibits proliferation. Mechanistically, PAMP binds to and inhibits the enzymatic activity of PYCR1, a key enzyme in proline biosynthesis. PYCR1 is upregulated in LUAD. Although small-molecule inhibitors targeting PYCR1 (such as PYCR1-IN-1) and other inhibitors of proline metabolism, including PRODH inhibitors such as L-selenomethionine, quinolinic acid, phenylbenzimidazole and ascivystatin, have been explored preclinically, the development of highly effective and specific agents for clinical use remains challenging, pointing to the need for novel targeting strategies (Chen et al, 2024; Shin et al, 2025). We further found that the binding of PAMP to PYCR1, mediated through PAMP-F16 and PYCR1-N123 residues, disrupts proline production and suppresses tumor growth. To translate these findings into potential therapeutic applications, we synthesized PAMP and demonstrated its ability to effectively inhibit proline synthesis and LUAD proliferation both in vitro and in vivo. These findings uncover a previously unrecognized lncRNA-encoded micropeptide that regulates tumor metabolism, offering a promising avenue for the development of new therapeutic strategies in LUAD.

## Result

### PSMA3-AS1 acts as a tumor suppressor in LUAD

**Figure 1:**
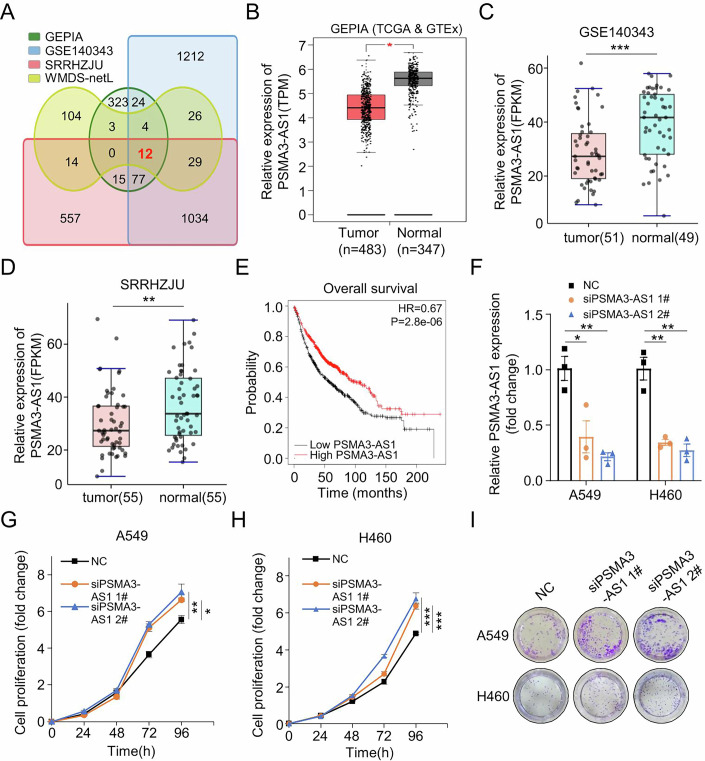
PSMA3-AS1 acts as a tumor suppressor in LUAD. (**A**) Venn diagram depicting the intersection of differentially expressed lncRNAs across the four datasets (GEPIA, GSE140343, SRRHZJU, and WMDS-netL co-expression network). (**B**–**D**) Relative expression of PSMA3-AS1 in LUAD tissues and adjacent normal tissues (**B**: *P* = 0.017; **C**: *P* = 1.2E-04; **D**: *P* = 0.009). Box plots show the median as the center line, the interquartile range (IQR; 25th–75th percentiles) as the box bounds, and whiskers extending to the minimum and maximum values within 1.5 × IQR. (**E**) Kaplan–Meier survival curves showing the association between PSMA3-AS1 expression and overall survival in LUAD patients. (**F**) qPCR analysis of PSMA3-AS1 expression in A549 and H460 cells transfected with PSMA3-AS1 siRNA or negative control (NC) siRNA (*n* = 3, A549: *P* (siPSMA3-AS1 1#) =0.028; *P* (siPSMA3-AS1 2#) = 0.002; H460: *P* (siPSMA3-AS1 1#) = 0.004; *P* (siPSMA3-AS1 2#) = 0.008). Expression levels are presented as fold changes relative to the NC group, which was used as the reference condition and set to 1. (**G**,** H**) CCK-8 assays assessing cell proliferation in LUAD cells transfected with NC siRNA or PSMA3-AS1 siRNA (*n* = 5, **G**: *P* (siPSMA3-AS1 1#) = 0.022; *P* (siPSMA3-AS1 2#) = 0.004; **H**: *P* (siPSMA3-AS1 1#) = 2.88E-05; *P* (siPSMA3-AS1 2#) = 2.99E-04). Cell proliferation is presented as fold change relative to the 0 h time point, which served as the reference condition. (**I**) Colony formation assays assessing the impact of PSMA3-AS1 knockdown on LUAD cell viability. Data were shown as mean ± SEM in (**C**,** D**, **F**–**H**). ^*^P < 0.05; ^**^P < 0.01; ^***^P < 0.001; two-tailed unpaired *t*-test. Source data are available online for this figure.

**Figure EV1:**
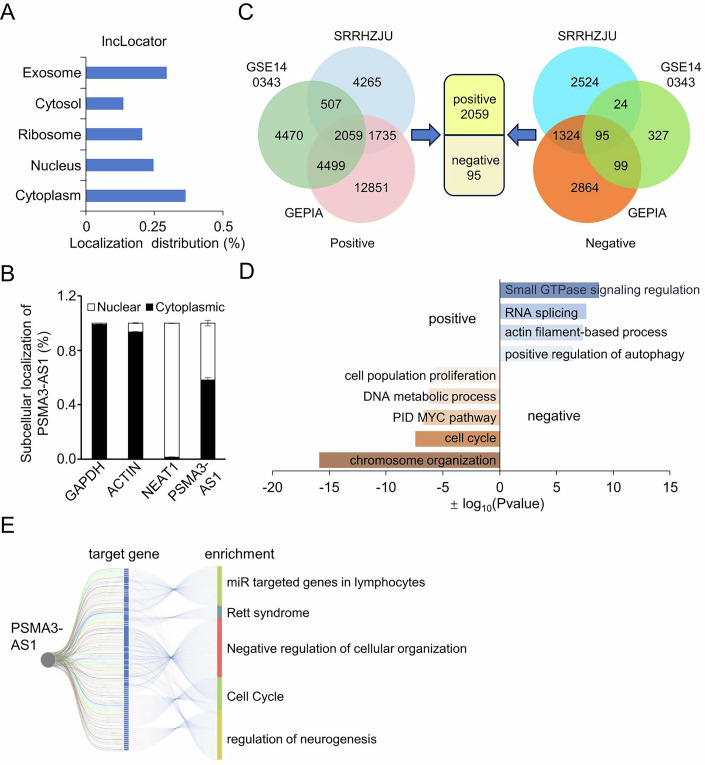
PSMA3-AS1 localizes to both the nucleus and cytoplasm and correlates with proliferation. (**A**) Predicted subcellular localization of PSMA3-AS1 using lncLocator. (**B**) qPCR analysis of cytoplasmic or nuclear PSMA3-AS1 RNA levels in A549 cells. GAPDH and ACTIN served as cytoplasmic controls, while NEAT1 served as a nuclear control (*n* = 3). (**C**) The gene intersection results of PSMA3-AS1 correlation analysis based on three RNA-seq data (Up: genes positively correlated with PSMA3-AS1; Down: genes negatively correlated with PSMA3-AS1). (**D**) Enrichment analysis of signaling pathways and biological processes derived from PSMA3-AS1 correlation analysis. (**E**) Sankey diagram showing the enrichment analysis results for mRNA genes associated with PSMA3-AS1 in the WMDS.netL network. Data were shown as mean ± SEM in (**B**). The correlation analysis retained all the genes with a $P$ value less than 0.05; statistical analysis was performed using Pearson correlation (**C**,** E**). Source data are available online for this figure.

To identify lncRNAs involved in LUAD, we integrated significantly differentially expressed lncRNAs from multiple RNA-seq datasets (TCGA, GSE140343, and SRRHZJU) with lncRNAs identified via WMDS-netL (Weighted Minimum Dominating Set)-based co-expression network analysis (Cheng et al, 2023b, Data ref: Xu et al, 2020). This integrative approach yielded 12 candidate lncRNAs, among which PSMA3-AS1 emerged as the most prominent (Fig. 1A). PSMA3-AS1 was markedly downregulated in LUAD tissues compared to adjacent normal tissues (Fig. 1B–D). Kaplan–Meier survival analysis demonstrated that higher PSMA3-AS1 expression was significantly associated with improved overall survival (HR = 0.67, *p* = 2.8e-06) (Fig. 1E). Subcellular localization analysis using lncLocator and fractionation qPCR confirmed the presence of PSMA3-AS1 in both the nucleus and cytoplasm (Fig. EV1A,B).

To investigate its functional role, we conducted weighted gene co-expression network analysis using three independent LUAD RNA-seq datasets. PSMA3-AS1 was consistently co-expressed with gene modules enriched in cell proliferation pathways (Fig. EV1C). Further enrichment analysis revealed a strong negative association between PSMA3-AS1 expression and signatures related to the cell cycle and cell population proliferation (Fig. EV1D). Similarly, WMDS-netL-based network analysis linked PSMA3-AS1 to cell cycle regulatory pathways (Fig. EV1E).

To functionally validate its role, we performed siRNA-mediated knockdown in LUAD cell lines. Quantitative PCR confirmed efficient knockdown of PSMA3-AS1 in A549 and H460 cells (Fig. 1F). Functional assays demonstrated that silencing PSMA3-AS1 significantly enhanced LUAD cell proliferation, as shown by CCK-8 and colony formation assays (Fig. 1G–I). Together, these findings indicate that PSMA3-AS1 acts as a tumor suppressor in LUAD by negatively regulating cell proliferation.

### PSMA3-AS1 encodes a micropeptide, PAMP

**Figure 2:**
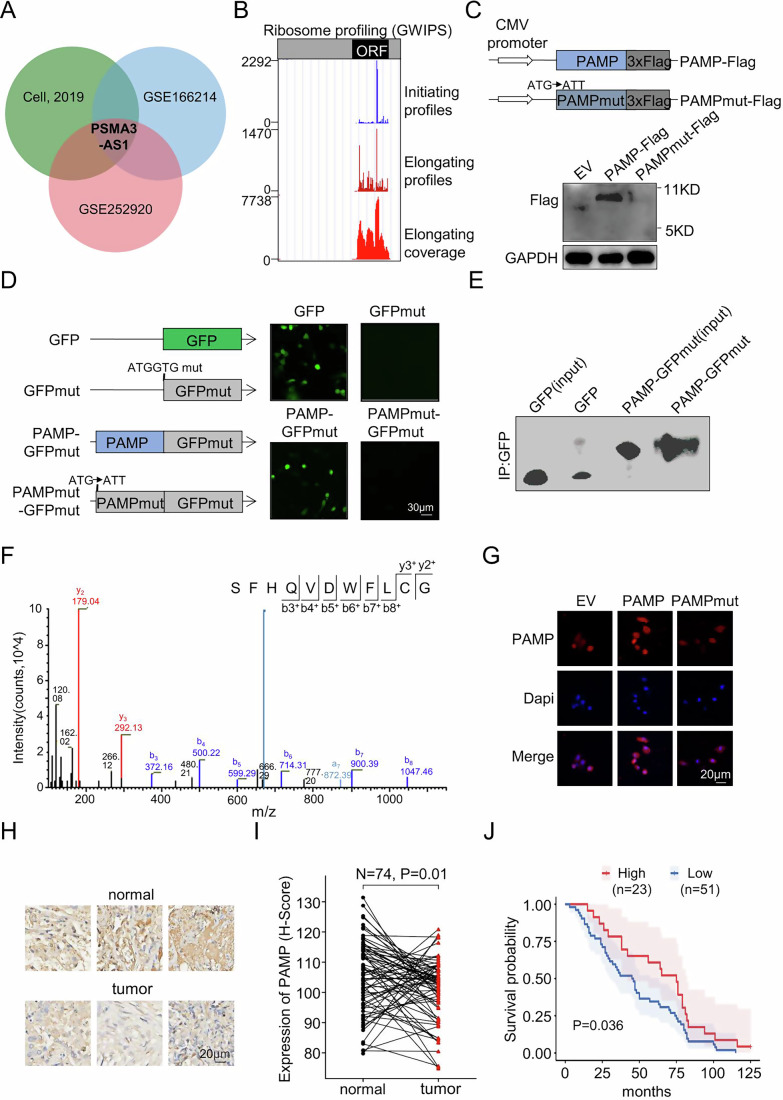
PSMA3-AS1 encodes a micropeptide, PAMP. (**A**) Venn diagram depicting the overlap of lncRNAs with translational potential among three human ribosome-profiling datasets (Cell, 2019; GSE166214; GSE252920), identifying PSMA3-AS1 as a shared element. (**B**) Ribosome-profiling data retrieved from the GWIPS-viz Genome Browser showing ribosome occupancy on the PSMA3-AS1 ORF. (**C**) Schematic representation of PAMP-3xFlag and PAMPmut-3xFlag constructs, cloned into PLVX vector and transfected into A549 cells for 24 h (top). Western blot analysis of PAMP/PAMPmut-Flag fusion proteins using anti-Flag antibody (down). (**D**) Diagram of GFP fusion constructs used for transfection (left). The GFP gene start codon ATGGTG was deleted (GFPmut), and the PAMP start codon ATG was mutated to ATT. Constructs were transfected into A549 cells for 24 h, and GFP fluorescence was detected (right). (**E**) Immunoprecipitation (IP) of cell lysates using an anti-GFP antibody, followed by immunoblotting. (**F**) Unique PAMP peptide identified via mass spectrometry analysis. (**G**) Immunofluorescence detection of PAMP in A549 cells. Nuclei were stained with DAPI (blue). (**H**,** I**) IHC analysis of PAMP expression in LUAD tissues and adjacent normal tissues. (**J**) Kaplan–Meier survival analysis assessing the association between PAMP expression and LUAD patient prognosis. Kaplan–Meier survival curves for patients stratified by PAMP expression level (high vs. low). The optimal cut-off was determined by tertile. $P$ values were calculated using the log-rank test. Source data are available online for this figure.

**Figure EV2:**
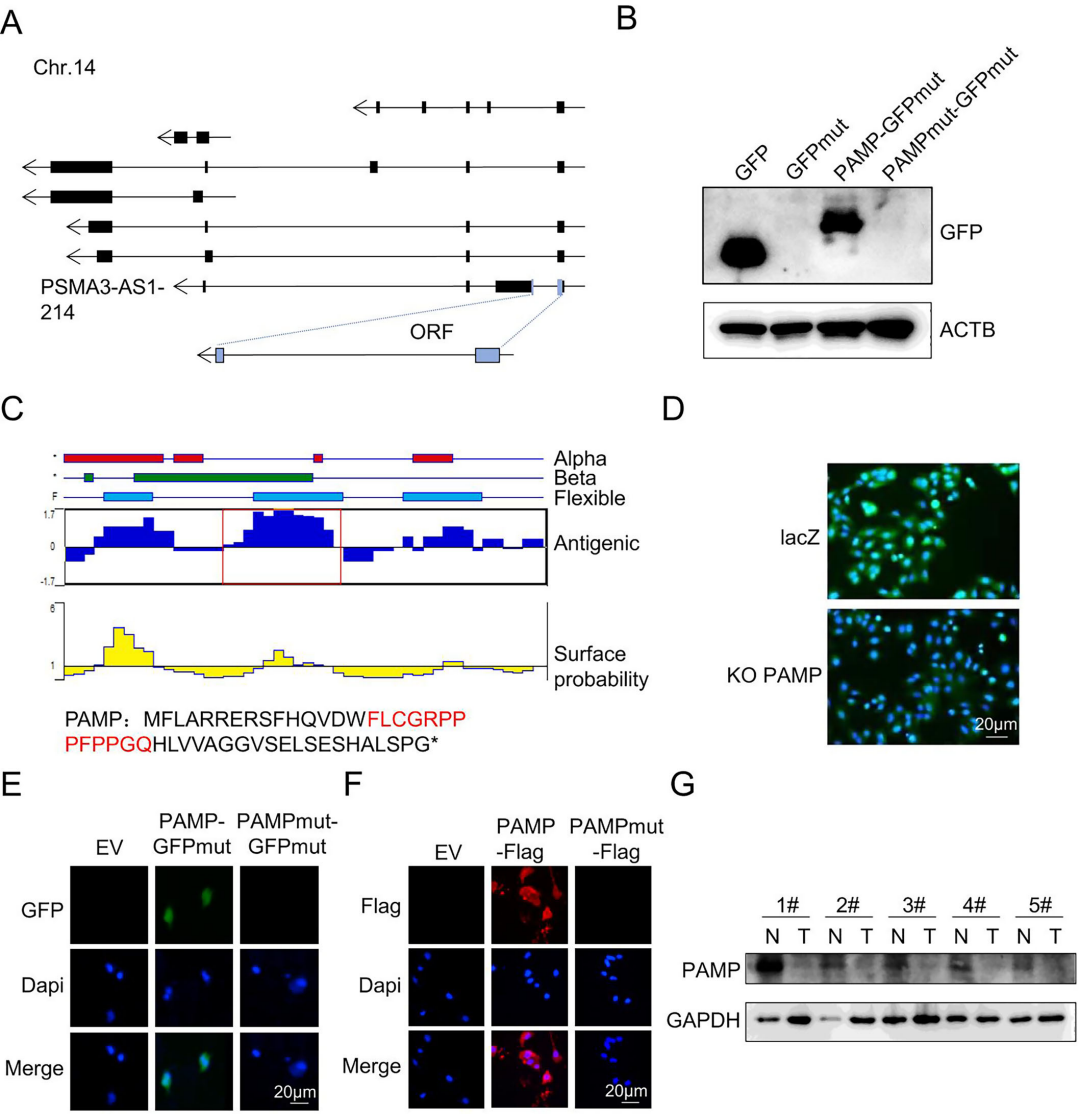
Validation of PAMP translation, antibody specificity, and clinical relevance. (**A**) Schematic representation of the genomic location of PSMA3-AS1. (**B**) Western blot analysis of fusion protein levels in A549 cells transfected with the indicated constructs for 24 h, using anti-GFP antibodies. (**C**) Prediction of the PAMP epitope using Protean software. The amino acid sequence highlighted in red was selected for polyclonal antibody preparation. (**D**) Immunostaining determined the PAMP antibody specificity by using KO-PAMP A549 cells. Nuclei were stained with DAPI (blue), Green color indicates the intensity of PAMP-specific signals. (**E**,** F**) Immunofluorescence detection of GFP and Flag signals in A549 cells transfected with the indicated constructs. Nuclei were stained with DAPI (blue). (**G**) Western blot analysis of PAMP peptide levels in an independent cohort of five pairs of LUAD tissues and matched adjacent normal tissues. Results were obtained from three independent experiments. Source data are available online for this figure.

To evaluate the coding potential of PSMA3-AS1, we integrated three ribosome-profiling datasets (Cell, 2019; GSE166214; GSE252920) derived from diverse tissue specimens (Data ref: Chi et al, 2024; Data ref: Douka et al, 2021; Data ref: van Heesch et al, 2019). Among seven lncRNAs with ribosome-interacting potential (Table EV1), PSMA3-AS1 exhibited the most significant enrichment in the polysome fraction (Fig. 2A). Further analysis of ribosome-profiling datasets combined with GWIPS-viz revealed substantial ribosome occupancy on PSMA3-AS1 transcript 214 (isoform 214) (Tierney et al, 2025), suggesting that its 147-nucleotide ORF undergoes translation (Figs. 2B and EV2A). Sequence comparisons indicated that the resulting 48-amino acid micropeptide lacks known protein domains or motifs, implying that it represents a previously uncharacterized micropeptide. Based on its functional properties, we designated this micropeptide PAMP (proline-associated micropeptide).

To validate the translational potential of PAMP, we constructed C-terminal 3xFlag-fused vectors containing either the wild-type PAMP or a start codon mutant. Robust expression of the PAMP-Flag fusion peptide was observed in PAMP-3xFlag (PAMP-Flag)-transfected cells, whereas mutation of the start codon (PAMPmut-Flag) abolished translation (Fig. 2C). To further confirm translation initiation from the PAMP start codon, we fused a GFP variant lacking its own start codon (Mut-GFP) to the C-terminus of PAMP. GFP signals were detected by microscope and Western blot analysis in PAMP–GFPmut–transfected cells, while mutation of the PAMP start codon (ATG to ATT) abolished GFP expression (Figs. 2D and EV2B). These results confirmed that the PAMP start codon is essential for translation initiation.

To further validate PAMP translation, we performed immunoprecipitation (IP) using an anti-GFP antibody on lysates from 293T cells transfected with PAMP–GFPmut. Western blot analysis confirmed successful enrichment of the expressed PAMP–GFPmut protein (Fig. 2E). Mass spectrometry analysis of the GFP immunoprecipitates identified a unique peptide fragment corresponding to PAMP (Fig. 2F), further supporting its translation.

To enable rigorous detection of endogenous PAMP, we developed a rabbit polyclonal antibody targeting conformational epitopes of PAMP based on its secondary structure (Fig. EV2C). Immunostaining assays confirmed the specificity of the antibody, as evidenced by a marked reduction in PAMP signal in PAMP knockout cells (Fig. EV2D). Immunofluorescence staining and immunohistochemistry demonstrated that PAMP is predominantly localized in the cytoplasm (Figs. 2G,H and EV2E,F). Clinically, PAMP expression was significantly lower in LUAD tissues compared to adjacent normal tissues (Figs. 2I and EV2G). Kaplan–Meier survival analysis revealed that LUAD patients with higher PAMP expression had a more favorable prognosis (Fig. 2J).

### PAMP inhibits LUAD cell proliferation

**Figure 3:**
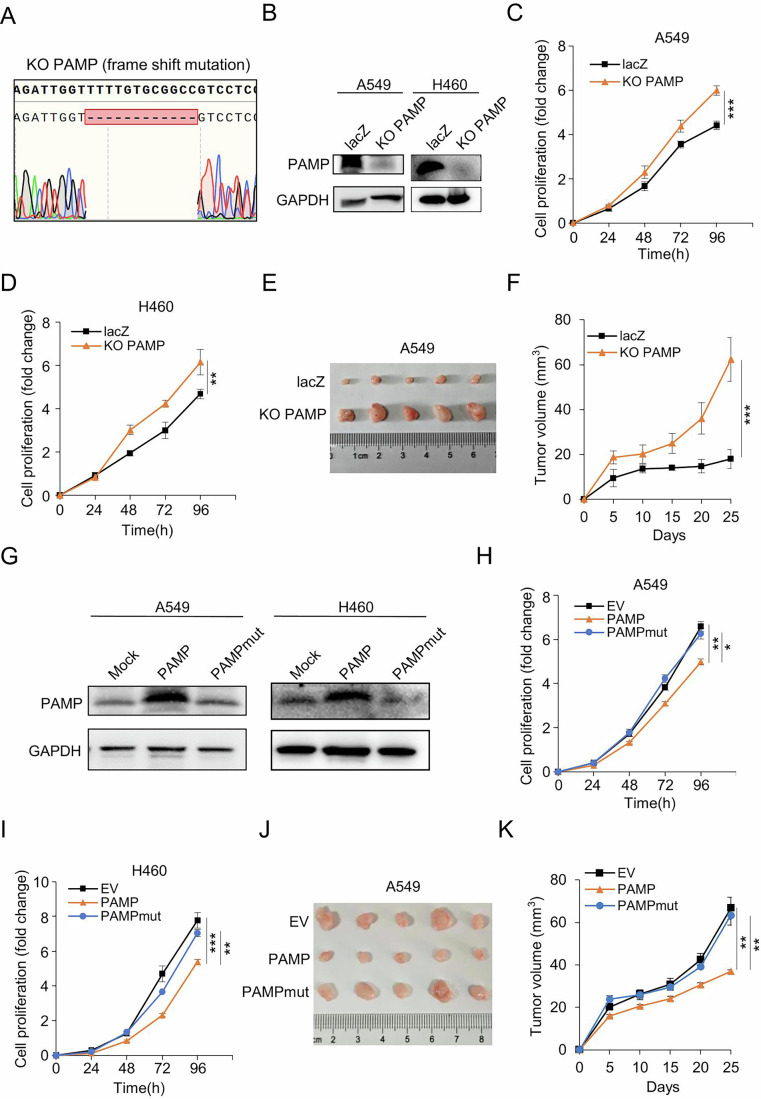
PAMP suppresses LUAD cell proliferation. (**A**) Sanger sequencing confirming CRISPR/Cas9-induced frameshift mutation in the PAMP genomic region. (**B**) Western blot analysis of PAMP protein levels in PAMP-knockout A549 and H460 cells. (**C**,** D**) CCK-8 assays assessing cell proliferation in control and PAMP knockout LUAD cell lines (*n* = 5, **C**: *P* = 1.84E-06; **D**: *P* = 0.003). Cell proliferation is presented as fold change relative to the 0 h time point, which served as the reference condition. (**E**,** F**) Nude mice were subcutaneously injected with control or PAMP knockout A549 cells. Representative tumors from at least five independent experiments were surgically excised. Tumor growth was monitored every five days to generate a growth curve (*n* = 5, *P* = 1.448E-05). (**G**) Western blot analysis of PAMP expression in LUAD cells overexpressing wild-type PAMP or PAMPmut using anti-PAMP antibody. (**H**,** I**) CCK-8 assays assessing cell proliferation in LUAD cells overexpressing wild-type PAMP or PAMPmut (*n* = 5, **H**: *P* (EV vs PAMP) = 0.004; *P* (PAMP vs PAMPmut) = 0.015; **I**: *P* (EV vs PAMP) = 0.003; *P* (PAMP vs PAMPmut) = 2.39E-04). Cell proliferation is presented as fold change relative to the 0 h time point, which served as the reference condition. (**J**,** K**) Nude mice were subcutaneously injected with empty vector (EV), wild-type PAMP or PAMPmut A549 cells. Representative tumors are shown, and tumor growth was monitored every 5 days to generate growth curves (*n* = 5, *P* (EV vs PAMP) = 0.004; *P* (PAMP vs PAMPmut) = 0.004). Data were shown as mean ± SEM in (**C**,** D**,** F**,** H**,** I**,** K**). ^*^P < 0.05; ^**^P < 0.01; ^***^P < 0.001; two-tailed unpaired *t*-test. Source data are available online for this figure.

**Figure EV3:**
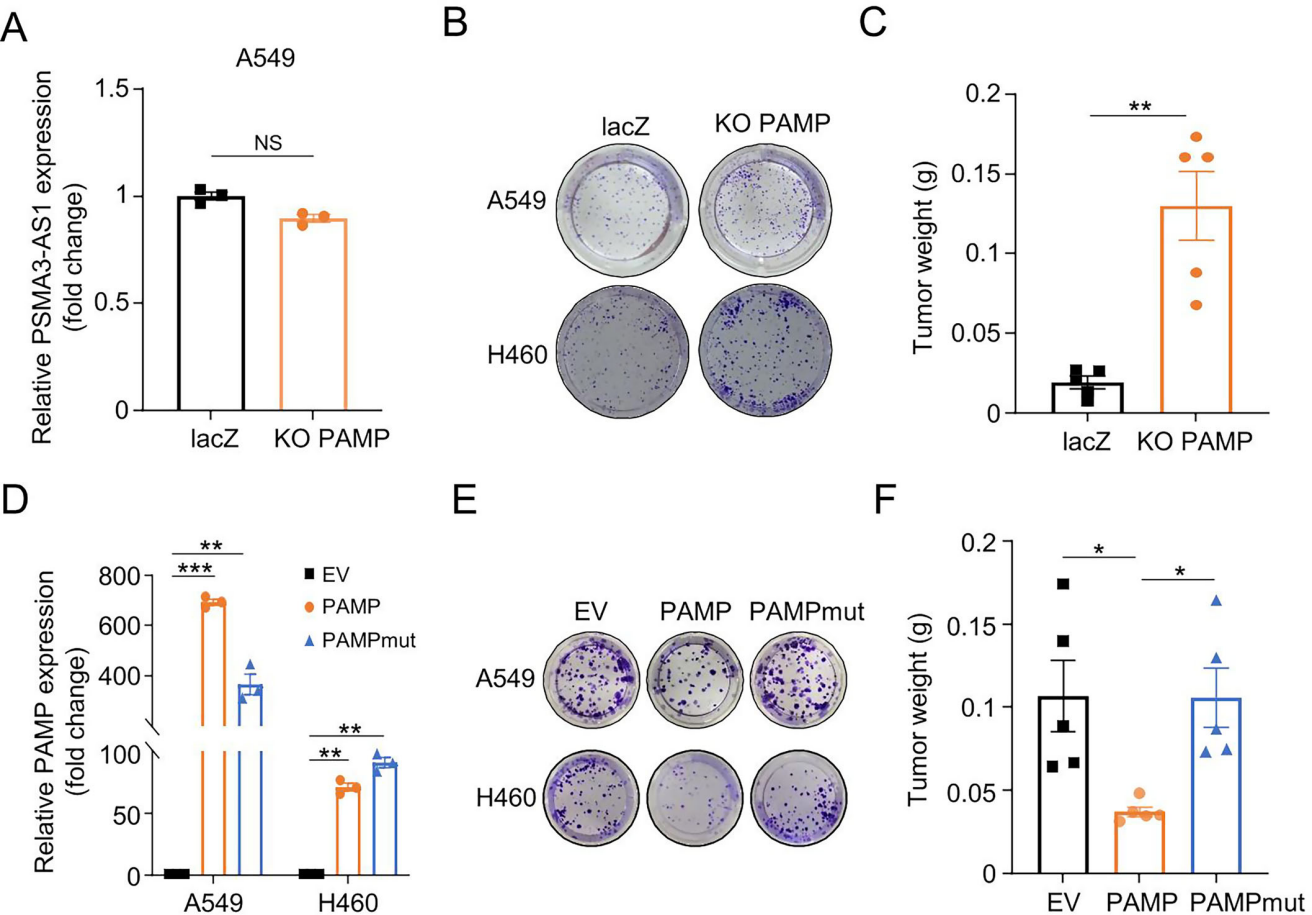
PAMP functions as a tumor suppressor. (**A**) qPCR analysis of PSAMA3-AS1 expression in A549 cells following PAMP knockout (*n* = 3). Primers were designed outside the PAMP region. Proline accumulation is presented as fold change relative to the LacZ control group, which was used as the reference condition and set to 1. (**B**) Colony formation assays assessing the impact of PAMP knockout on LUAD cell viability. (**C**) Quantification of tumor weight in xenograft mice from PAMP knockout and control mice (*n* = 5, *P* = 0.001). (**D**) qPCR analysis of PAMP RNA expression in LUAD cells overexpressing PAMP or PAMPmut constructs (*n* = 3, A549: *P* (EV vs PAMP) = 3.23E-04; *P* (PAMP vs PAMPmut) = 0.01; H460: *P* (EV vs PAMP) = 0.001; *P* (PAMP vs PAMPmut) = 0.002). Proline accumulation is presented as fold change relative to the EV control group, which was used as the reference condition and set to 1. (**E**) Colony formation assays to assess cell proliferation in LUAD cells overexpressing wild-type PAMP or PAMPmut. (**F**) Quantification of tumor weight in xenograft mice from EV, wild-type PAMP and PAMPmut mice (*n* = 5, *P* (EV vs PAMP) = 0.031; *P* (PAMP vs PAMPmut) = 0.017). Data were shown as mean ± SEM in (**C**,** D**,** F**). ^*^P < 0.05; ^**^P < 0.01; ^***^P < 0.001; two-tailed unpaired *t*-test. Source data are available online for this figure.

Having established that PSMA3-AS1-214 encodes a functional micropeptide, we next investigated PAMP’s role in LUAD progression. Using CRISPR/Cas9-mediated genome editing, we efficiently knocked down PAMP in LUAD cells while preserving the RNA profile of PSMA3-AS1 (Figs. 3A,B and EV3A). CCK-8 and colony formation assays revealed that A549 and H460 cells expressing KO-PAMP sgRNAs exhibited enhanced proliferation capacity (Figs. 3C,D and EV3B). To further investigate PAMP’s role in LUAD progression in vivo, KO-PAMP A549 cells and control cells (lacZ) were injected subcutaneously into 5 paired nude mice to compare tumor growth rates. Knockdown of the PAMP significantly accelerated the LUAD tumor development in vivo (Figs. 3E,F and EV3C).

To distinguish whether PSMA3-AS1 or its encoded PAMP peptide plays the primary role in tumor progression, we transfected LUAD cells with PAMP and PAMPmut plasmids (Figs. 3G and EV3D). Overexpression of PAMP significantly suppressed LUAD cell proliferation, whereas mutation of the PAMP start codon abolished this inhibitory effect (Figs. 3H,I and EV3E). Consistent results were obtained in a subcutaneous tumor xenograft model, where mutation of the PAMP start codon eliminated its suppressive effect on tumor growth in vivo (Figs. 3J,K and EV3F). Collectively, these findings suggested that PSMA3-AS1-214-encoded micropeptide PAMP functions as a tumor suppressor, inhibiting LUAD cell proliferation.

### PAMP interacts with PYCR1 and alters proline metabolism

**Figure 4:**
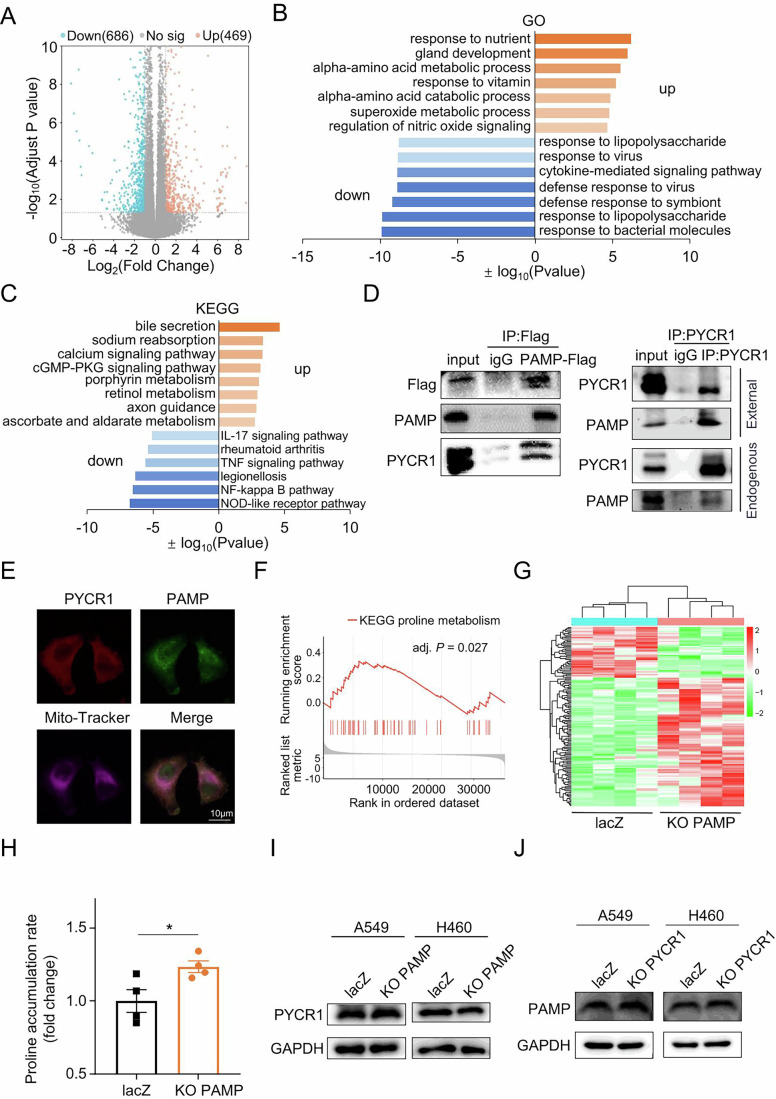
PAMP interacts with PYCR1 and modulates proline metabolism. (**A**) Volcano plot illustrating differential gene expression following PAMP knockout, (*n* = 3, |log_2_ FC|>1, and adjust *P* value <0.05, differentially expressed genes were identified using DESeq2). (**B**,** C**) GO and KEGG enrichment analysis of biological processes and signaling pathways associated with differentially expressed genes upon PAMP knockout. (**D**) Co-IP of whole-cell lysates from A549 cells transfected with PAMP-Flag plasmid using anti-Flag antibody, followed by immunoblot analysis with an anti-PYCR1 antibody to verify PYCR1-PAMP interaction (left). Reciprocally, Co-IP using an anti-PYCR1 antibody was performed to capture either exogenously expressed (transfected PAMP) or endogenous PAMP proteins. Whole-cell lysates from transfected (external) or untransfected (endogenous) cells were subjected to IP with the PYCR1 antibody or control IgG. Input lysates and Co-IP samples were analyzed by western blot using the indicated antibodies (right). (**E**) Immunostaining showing the co-localization of PAMP and PYCR1 in mitochondria. (**F**) GSEA enrichment plot analyzing proline metabolism pathway using RNA-seq data from PAMP-knockout A549 cells compared to lacZ control. (**G**,** H**) Non-targeted metabolomics analysis identifying differential metabolites between PAMP knockout and control groups. Heatmap illustrating all differential metabolites (fold change >1.2, and *P* < 0.05, *n* = 4). Proline accumulation rate was significantly elevated following PAMP knockout (*P* = 0.036). Fold change is shown relative to the LacZ control group, which was used as the reference condition and set to 1. (**I**,** J**) Western blot analysis examining the regulatory relationship between PAMP and PYCR1. Data were shown as mean ± SEM in (**H**). ^*^P < 0.05; ^**^P < 0.01; ^***^P < 0.001; two-tailed unpaired *t*-test. Source data are available online for this figure.

Given PAMP’s significant inhibitory effect on LUAD cell proliferation, we conducted RNA-seq analysis to further investigate its impact on LUAD by comparing gene expression profiles between PAMP knockout and control groups. Differential expression analysis revealed substantial changes, with 469 genes upregulated and 686 genes downregulated (|log_2_FC|>1, FDR <0.05) (Fig. 4A). Gene ontology (GO) analysis of upregulated genes highlighted biological processes associated with metabolism, particularly amino acid metabolism (Fig. 4B). Further kyoto encyclopedia of genes and genomes (KEGG) pathway analysis revealed significant enrichment of several cancer metabolic pathways (Fig. 4C).

**Figure EV4:**
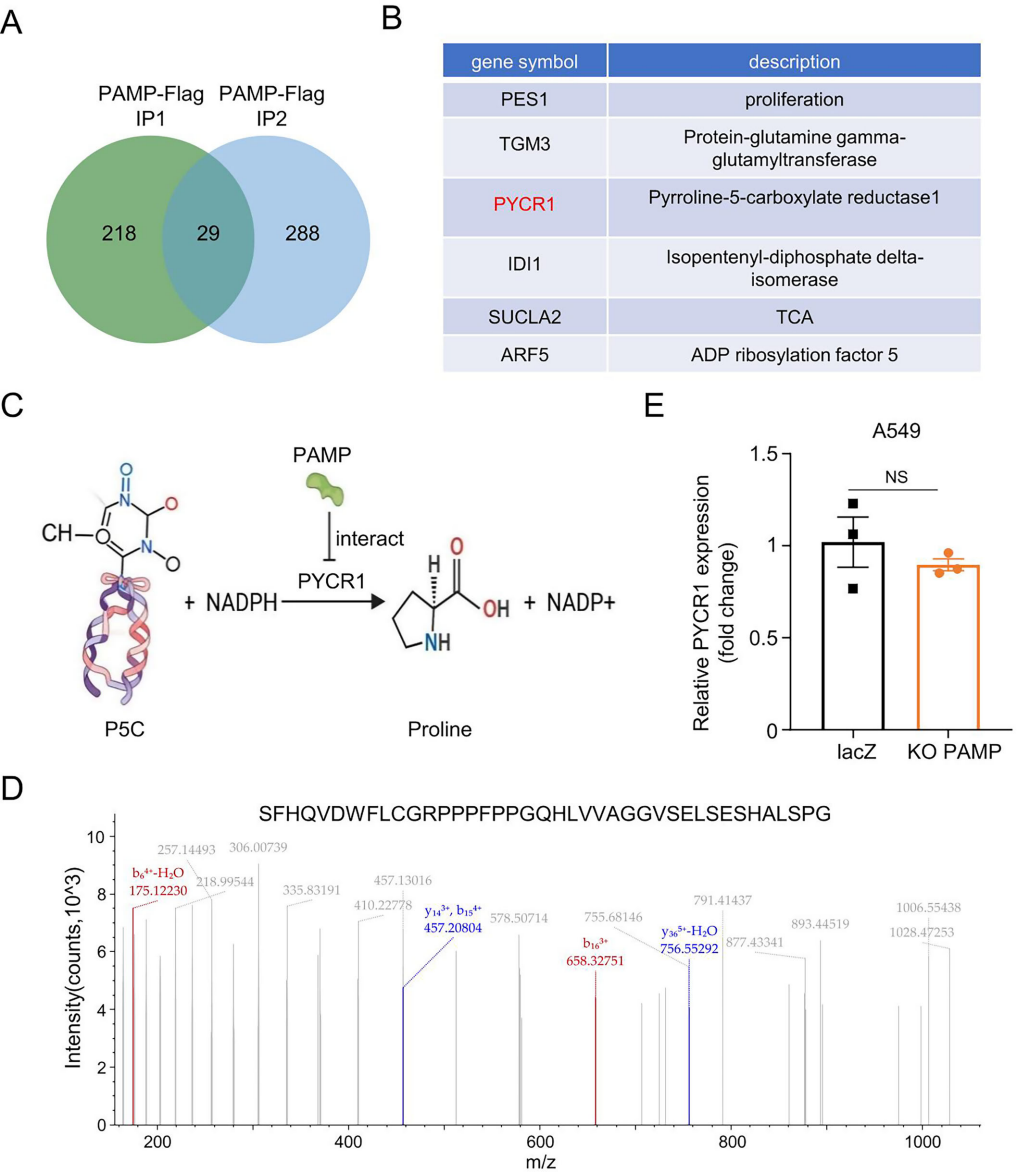
PYCR1 is a key interacting protein of PAMP. (**A**) Venn diagram comparing results from two independent Co-IP experiments. (**B**) Proteins identified by mass spectrometry analysis of Co-IP fractions. (**C**) Schematic illustrating the PYCR1-catalyzed reduction of P5C to proline, which is coupled with the oxidation of NADPH to NADP⁺. The interaction of the regulatory factor PAMP with PYCR1 is depicted. (**D**) Endogenous PAMP-specific peptide segments enriched by Co-IP identified via mass spectrometry analysis. (**E**) qPCR analysis of PYCR1 expression in A549 cells following PAMP knockout (*n* = 3). Proline accumulation is presented as fold change relative to the LacZ control group, which was used as the reference condition and set to 1. Data were shown as mean ± SEM in (**E**). A two-tailed unpaired *t*-test was performed, with *P* < 0.05 considered statistically significant. Source data are available online for this figure.

To elucidate PAMP’s molecular mechanisms in LUAD, we performed co-immunoprecipitation (Co-IP) using an anti-Flag antibody following transfection with a PAMP-3xFlag construct. Mass spectrometry analysis from two independent experiments identified 29 candidate interacting proteins (Fig. EV4A). Among metabolism-related proteins interacting with PAMP (Table EV2), PYCR1 emerged as a key interactor of interest, as it encodes an enzyme that catalyzes the NAD(P)H-dependent conversion of pyrroline-5-carboxylate to proline (Fig. EV4B,C) (Zheng et al, 2023). We confirmed that endogenous PYCR1 could be immunoprecipitated by the PAMP-Flag fusion protein. Reciprocally, endogenous PAMP was detected in PYCR1 immunoprecipitates from A549 cells by Co-IP coupled with LC-MS using an anti-PYCR1 antibody (Figs. 4D and EV4D). Additionally, the fluorescence-based mitochondrial co-localization further supports the physical interaction between PAMP and PYCR1 (Fig. 4E).

Given the observed increase in metabolic activity following PAMP knockout and PYCR1’s established role in proline synthesis, we performed gene set enrichment analysis (GSEA), which revealed a negative correlation between PAMP expression and proline metabolic pathways (Fig. 4F). Non-targeted metabolic profiling detected 143 metabolites, among which proline levels were significantly elevated in PAMP-deficient LUAD cells (Fig. 4G,H).

To further explore the regulatory relationship between PAMP and PYCR1, we examined their expression levels upon depletion. Interestingly, PAMP knockout did not significantly alter PYCR1 levels, nor did PYCR1 depletion affect PAMP expression (Figs. 4I,J and EV4E). These findings confirm that PAMP interacts with PYCR1 and is associated with proline metabolism, but does not regulate PYCR1 expression directly.

### PAMP-mediated inhibition of proline synthesis is dependent on PYCR1

**Figure 5:**
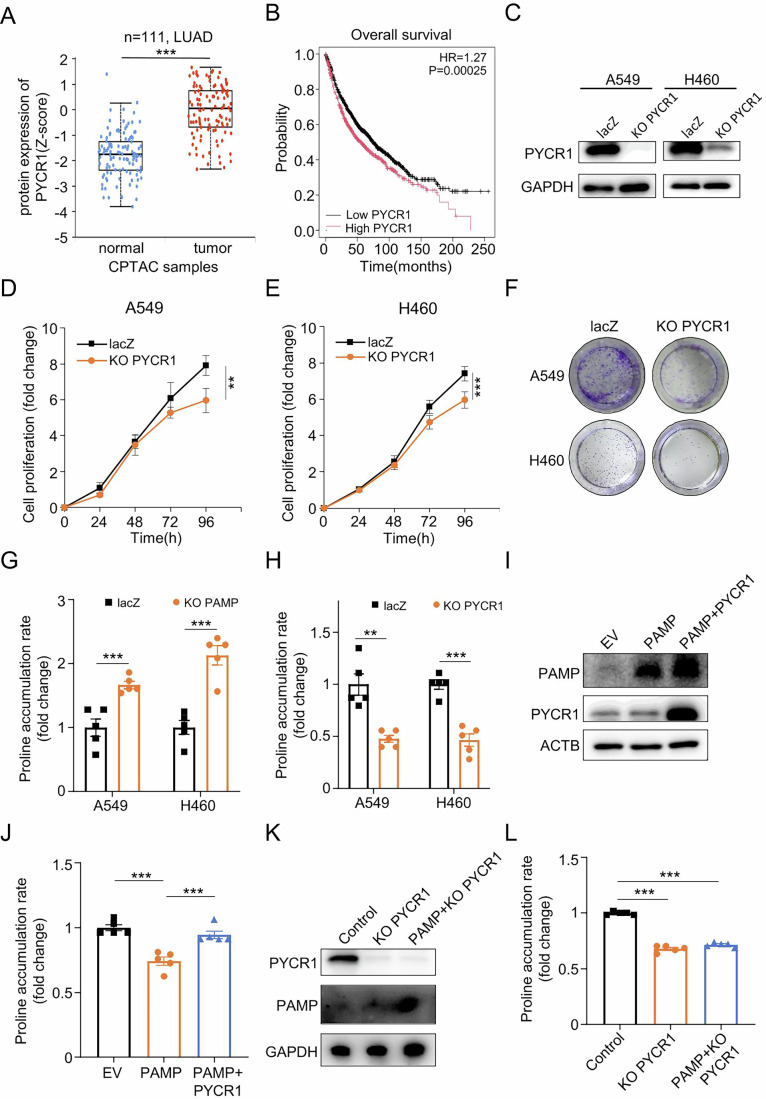
PAMP inhibits proline synthesis via PYCR1. (**A**) Protein expression analysis of PYCR1 in LUAD tissues and adjacent tissues based on CPTAC samples (*P* = 2.753E-30). (**B**) Kaplan–Meier survival curves comparing overall survival in LUAD patients with high and low PYCR1 expression. (**C**) Western blot analysis of PYCR1 protein levels in control and PYCR1 knockout LUAD cells. (**D**,** E**) CCK-8 assays assessing cell proliferation in control and PYCR1-knockout LUAD cells (*n* = 5, **D**: *P* = 0.001; **E**: *P* = 5.61E-4). Cell proliferation is presented as fold change relative to the 0 h time point, which served as the reference condition. (**F**) Colony formation assays evaluating the impact of PYCR1 knockout on LUAD cell viability. (**G**) Proline levels measured in control and PAMP-knockout LUAD cells (*n* = 5, A549: *P* = 0.005; H460: *P* = 4.21E-4). Proline accumulation is presented as fold change relative to the LacZ control group, which was used as the reference condition and set to 1. (**H**) Proline levels measured in control and PYCR1-knockout LUAD cells (*n* = 5, A549: *P* = 0.004; H460: *P* = 1.45E-4). Proline accumulation is presented as fold change relative to the LacZ control group, which was used as the reference condition and set to 1. (**I**,** J**) Western blot analysis of indicated proteins in A549 cells co-transfected with PAMP and PYCR1 plasmids, followed by proline level quantification (*n* = 5, *P* (EV vs PAMP) = 2.35E-4; *P* (PAMP vs PAMP + PYCR1) = 0.001). Proline accumulation is presented as fold change relative to the EV group, which was used as the reference condition and set to 1. (**K**) Western blot analysis of PYCR1 and PAMP protein levels under the indicated conditions. GAPDH serves as a loading control. (**L**) Quantitative analysis of intracellular proline levels in the indicated experimental groups (*n* = 5, *P* (Control vs KO PYCR1) = 0.001; *P* (Control vs PAMP + KO PYCR1) = 7.038E-05). Proline accumulation is presented as fold change relative to the control group, which was used as the reference condition and set to 1. Data were shown as mean ± SEM in (**D**,** E**,** G**,** H**,** J**,** L**). ^*^P < 0.05; ^**^P < 0.01; ^***^P < 0.001; two-tailed unpaired *t*-test. Source data are available online for this figure.

**Figure EV5:**
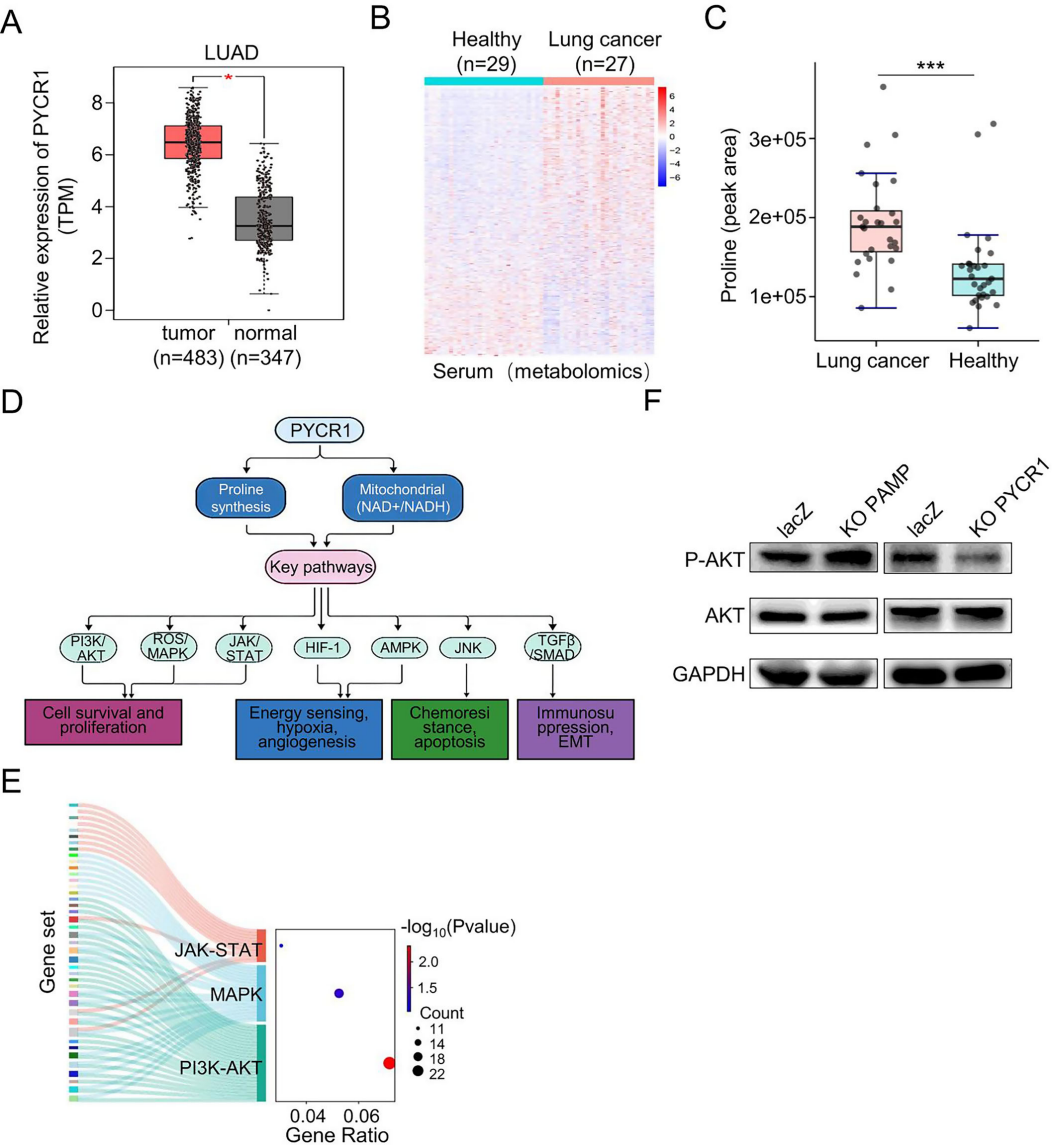
PAMP-PYCR1 axis regulates AKT phosphorylation. (**A**) Relative expression of PYCR1 in LUAD tissues and adjacent normal tissues from the TCGA cohort (*P* = 8.7E-36). (**B**) Metabolic heatmap showing alterations in plasma samples from lung cancer patients compared with healthy individuals. The color bar indicates log2 fold change. (**C**) Comparison of plasma proline levels between lung cancer patients and healthy individuals (*P* = 8.72E-4). (**D**) Schematic of PYCR1-centered metabolic and functional network. PYCR1 integrates signals from serum metabolomic features and regulates proline synthesis as well as mitochondrial NAD+/NADH homeostasis. These core functions converge on key pathways, subsequently driving diverse downstream biological processes including cell survival, proliferation, stress responses, and immunosuppression. (**E**) GSEA indicates that PAMP is significantly associated with the PI3K-Akt, JAK-STAT, and MAPK pathways, which are known regulators of cell proliferation as annotated in the SMPDB shown in (**D**). (**F**) Western blot analysis of phosphorylated AKT in lacZ, KO-PAMP, and KO PYCR1 cells. GAPDH serves as the loading control. Data are shown as mean ± SEM in (**C**). ^***^P < 0.001; two-tailed unpaired *t*-test. Box plots (**A**, **C**) show the median as the center line, the interquartile range (IQR; 25th–75th percentiles) as the box bounds, and whiskers extending to the minimum and maximum values within 1.5 × IQR. Source data are available online for this figure.

Given the interaction between PAMP and PYCR1, along with the inhibitory effect of PAMP on LUAD proliferation, we next investigated the role of PYCR1 in LUAD. GEPIA and CPTAC analyses revealed that PYCR1 mRNA and protein levels were significantly upregulated in LUAD (Figs. 5A and EV5A). Kaplan–Meier survival analysis showed that high PYCR1 expression was associated with shorter survival in lung cancer patients (Fig. 5B). To determine whether PYCR1 contributes to LUAD proliferation, we conducted CCK-8 and colony formation assays using LUAD cells with PYCR1 knockout. PYCR1 depletion significantly reduced LUAD cell proliferative capacity (Fig. 5C–F).

Proline, a key amino acid in tumor metabolism catalyzed by PYCR1 (Westbrook et al, 2022), was found to be significantly elevated in the stroma of lung cancer, as demonstrated by metabolomics analysis (Fig. EV5B,C). To investigate whether PAMP regulates PYCR1-mediated proline synthesis, we measured intracellular proline accumulation. PAMP depletion significantly increased proline levels, whereas PYCR1 knockout reduced proline accumulation (Fig. 5G,H). Notably, the suppression of proline synthesis by PAMP was rescued by PYCR1 overexpression (Fig. 5I,J). To further confirm that PAMP’s effect on proline metabolism depends on PYCR1, we overexpressed PAMP in PYCR1-deficient LUAD cells. Although PYCR1 depletion significantly reduced proline accumulation, PAMP overexpression no longer altered proline levels in the absence of PYCR1 (Fig. 5K,L). Together, these results indicate that the regulatory effect of PAMP on proline metabolism is PYCR1-dependent.

Given that PAMP interacts with PYCR1 and inhibits proline synthesis, we next investigated downstream signaling pathways potentially involved. The small-molecule pathway database (SMPDB) analysis revealed that PYCR1 and proline are linked to several key signaling pathways, including JNK, HIF-1, TGF-β, PI3K-Akt, JAK-STAT, AMPK, and MAPK signaling (Fig. EV5D). GSEA further showed that PAMP expression was significantly correlated with the PI3K-Akt, JAK-STAT, and MAPK pathways, all of which are well documented to be involved in cell proliferation according to the SMPDB analysis (Fig. EV5E). We therefore examined these pathways in both PAMP-KO and PYCR1-KO A549 cell lines. Notably, loss of PAMP led to increased p-AKT levels, whereas loss of PYCR1 resulted in decreased p-AKT levels, suggesting that AKT is a key downstream effector of the PAMP-PYCR1-proline axis (Fig. EV5F).

### PAMP-F16 and PYCR1-N123 mediate critical interactions

**Figure 6:**
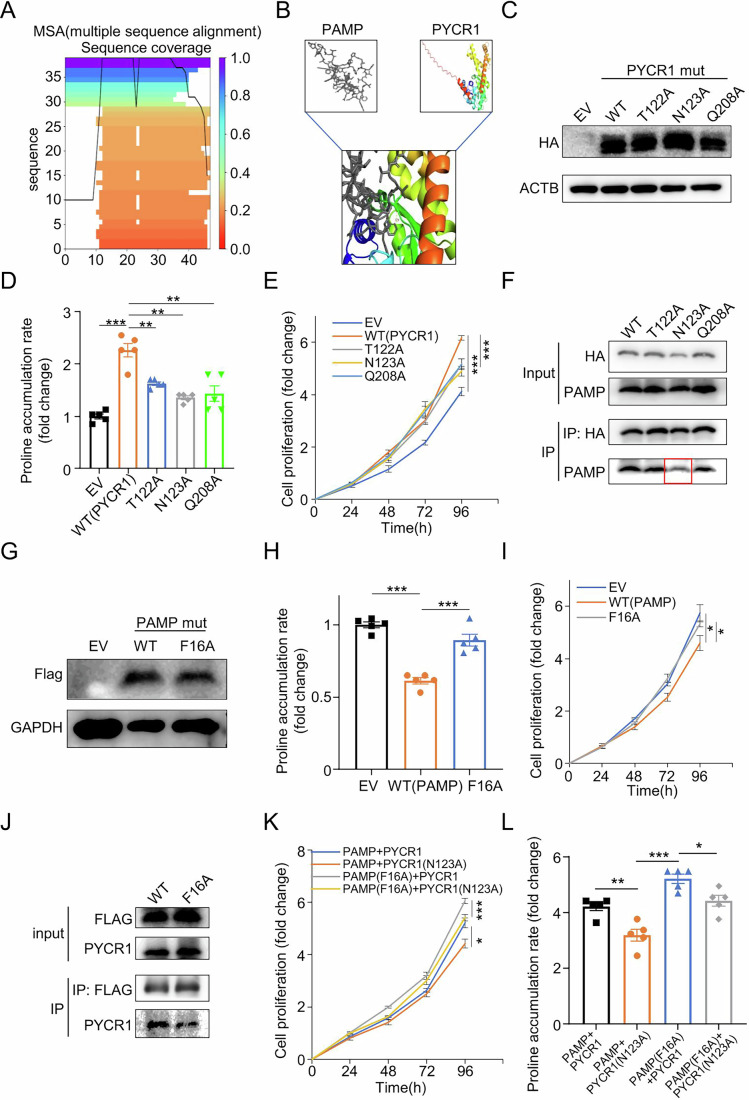
PAMP-F16 and PYCR1-N123 mediate critical interactions. (**A**) Heatmap representation of multiple sequence alignment (MSA) showing sequence identity scores. Sequences are ordered from highest to lowest identity, with white regions indicating uncovered areas due to sub-sequence entries in the database. The black line represents the relative sequence coverage. (**B**) Graphical representation of three-dimensional structures of PAMP and PYCR1 docking models, with a magnified view highlighting the interaction between N123 in PYCR1 and F16 in PAMP. (**C**) Western blot analysis of protein expression in A549 cells transfected with wild-type (WT) and mutant PYCR1, coupled with an HA tag. (**D**) Proline levels were measured in A549 cells transfected with WT and mutant PYCR1 (*n* = 5, *P* (EV vs WT) = 1.68E-05; *P* (WT vs T122A) = 0.001; *P* (WT vs N123A) = 0.001; *P* (WT vs Q208A) = 0.003). Proline accumulation is presented as fold change relative to the EV group, which was used as the reference condition and set to 1. (**E**) CCK-8 assays assessing cell proliferation in A549 cells transfected with WT and mutant PYCR1 (*n* = 5, *P* (EV vs WT) = 3.61E-06; *P* (WT vs T122A) = 0.001; *P* (WT vs N123A) = 2.88E-04; *P* (WT vs Q208A) = 0.001). Cell proliferation is presented as fold change relative to the 0 h time point, which served as the reference condition. (**F**) Co-IP of A549 cells transfected with WT and mutant PYCR1 coupled with HA tag plasmids, followed by western blot analysis of the indicated proteins. (**G**) Western blot analysis of protein expression in A549 cells transfected with WT and mutant PAMP, coupled with a Flag tag. (**H**) Proline levels measured in A549 cells transfected with WT and mutant PAMP (*n* = 5, *P* (EV vs WT) = 1.28E-06; *P* (WT vs F16A) = 3.25E-04). Proline accumulation is presented as fold change relative to the EV group, which was used as the reference condition and set to 1. (**I**) CCK-8 assays assessing cell proliferation in A549 cells transfected with WT and mutant PAMP (*n* = 5, *P* (EV vs WT) = 0.028; *P* (WT vs F16A) = 0.041). Cell proliferation is presented as fold change relative to the 0 h time point, which served as the reference condition. (**J**) Co-IP of A549 cells transfected with WT and mutant PAMP coupled with Flag tag plasmids, followed by western blot analysis of the indicated proteins. (**K**) Cell proliferation was assessed by CCK-8 assay in cells co-expressing wild-type PAMP or binding-defective PAMP(F16A) with either wild-type PYCR1 or catalytically inactive PYCR1(N123A) (*n* = 5, *P* (PAMP + PYCR1 vs PAMP + PYCR1(N123A)) = 0.015; *P* (PAMP + PYCR1(N123A) vs PAMP(F16A) + PYCR1 = 1.33E-04; *P* (PAMP(F16A) + PYCR1 vs PAMP(F16A) + PYCR1(N123A) = 0.004). Cell proliferation is presented as fold change relative to the 0 h time point, which served as the reference condition. (**L**) Proline accumulation rates were quantitatively analyzed under the same experimental conditions as in (**K**) (*n* = 5, *P* (PAMP + PYCR1 vs PAMP + PYCR1(N123A)) = 0.005; *P* (PAMP + PYCR1(N123A) vs PAMP(F16A) + PYCR1 = 9.79E-05; *P* (PAMP(F16A) + PYCR1 vs PAMP(F16A) + PYCR1(N123A) = 0.015). Proline accumulation is presented as fold change relative to the PAMP + PYCR1 group, which was used as the reference condition and set to 1. Data were shown as mean ± SEM in (**D**,** E**,** H**,** I**,** K**,** L**). ^*^P < 0.05; ^**^P < 0.01; ^***^P < 0.001; two-tailed unpaired *t*-test. Source data are available online for this figure.

**Figure EV6:**
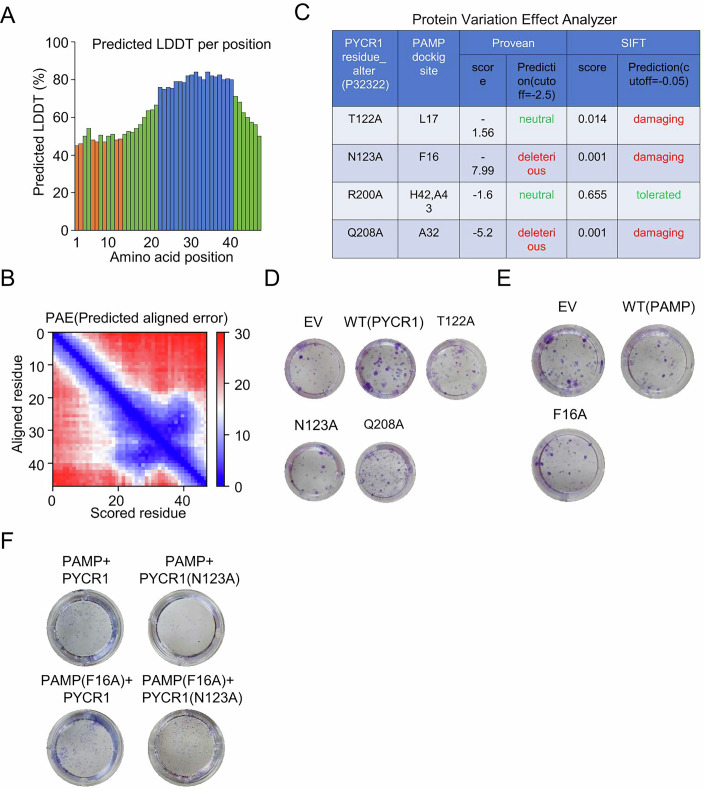
PAMP-F16 and PYCR1-N123 binding sites are critical for LUAD proliferation. (**A**) Residue-based pLDDT profile for the top AlphaFold2 models of PAMP conformations, derived from AlphaFold2 predictions. The structural model assessment distributions for the PAMP conformational ensemble come from PAMP-MSA shallow subsampling predictions. pLDDT, a local prediction score by AlphaFold2, assesses the reliability of residue geometry predictions. Scores range from 0 to 100, with higher values indicating more confidence: 90–100 (high precision), 70–90 (credible), 50–70 (low precision), and <50 (error). (**B**) Heatmaps illustrating the predicted alignment error (PAE) between residue pairs in the top-ranked model. The color scale highlights contrast between high-confidence and low-confidence regions. (**C**) Evaluation of amino acid variations at PYCR1 docking sites using Provean and SIFT. (**D**) Colony formation assays assessing cell proliferation in PAMP-mutated A549 cells. (**E**) Colony formation assays evaluating cell proliferation in PYCR1-mutated A549 cells. (**F**) Colony formation assays evaluating cell proliferation in cells co-expressing wild-type PAMP or binding-defective PAMP(F16A) with either wild-type PYCR1 or catalytically inactive PYCR1(N123A). Source data are available online for this figure.

Utilizing AlphaFold, an advanced algorithm based on multiple sequence alignment (MSA) (Yang et al, 2023), we predicted the 3D structure of PAMP (Fig. 6A). The reliability of the predicted structure was supported by favorable pLDDT (Predicted Local Distance Difference Test) and PAE (Predicted Aligned Error) scores (Fig. EV6A,B). Given PAMP’s role in proline metabolism via PYCR1 interaction, we obtained the PYCR1 structure from UniProt and performed molecular docking simulations using HDOCK and AlphaFold (Fig. 6B). Four most potential hydrogen bond interactions were identified within binding site residues. Alanine mutagenesis was performed at each of these four positions to assess their functional significance. Provean and SIFT (Sorts Intolerant From Tolerant) analyses further demonstrated that mutations at T122A, N123A, and Q208A significantly impair PYCR1 function (Fig. EV6C).

We generated PYCR1 residue mutants tagged with HA (Fig. 6C). Proline quantification assays revealed that mutations at T122, N123, and Q208 markedly suppressed proline accumulation, while CCK-8 and colony formation assays further confirmed that these mutations impaired LUAD cell proliferation (Figs. 6D,E and EV6D). Importantly, Co-IP assays showed that the PYCR1-N123A mutant largely lost its interaction with PAMP, indicating PYCR1-N123 is a major interaction site for PAMP (Fig. 6F).

In parallel, we constructed a PAMP-F16 mutant plasmid tagged with a Flag, corresponding to the PYCR1-N123 binding site (Fig. 6G). Mutation of PAMP-F16A significantly inhibited proline accumulation and LUAD cell proliferation (Figs. 6H,I and EV6E). Co-IP further confirmed that PAMP-F16 is a key site for PYCR1 interaction (Fig. 6J). To further investigate the role of these major interaction sites in cell proliferation and proline accumulation, rescue experiments were performed. Co-expression of PAMP(F16A) with wild-type PYCR1 significantly enhanced cell proliferation and proline accumulation, whereas these effects were markedly attenuated when PAMP(F16A) was combined with the catalytically impaired PYCR1(N123A) mutant. The double-mutant combination, PAMP(F16A) plus PYCR1(N123A), produced only partial restoration of both phenotypes, further supporting the coordinated role of PAMP and PYCR1 in regulating proline metabolism and cell growth (Figs. 6K,L and EV6F). Taken together, these findings demonstrate that the PYCR1-T122, PYCR1-N123, PYCR1-Q208, and PAMP-F16 are crucial for proline accumulation and LUAD cell proliferation, with PYCR1-N123 and PAMP-F16 being the primary interaction sites.

### Synthetic PAMP inhibits proline accumulation and LUAD progression

Polypeptide-based therapeutics have garnered significant attention due to their simple spatial structure, low immunogenicity, minimal toxicity, and high product purity (Wang et al, 2022). Given PAMP’s small molecular weight and potent antitumor activity, we investigated the therapeutic potential of synthetically produced PAMP peptides in LUAD.

**Figure EV7:**
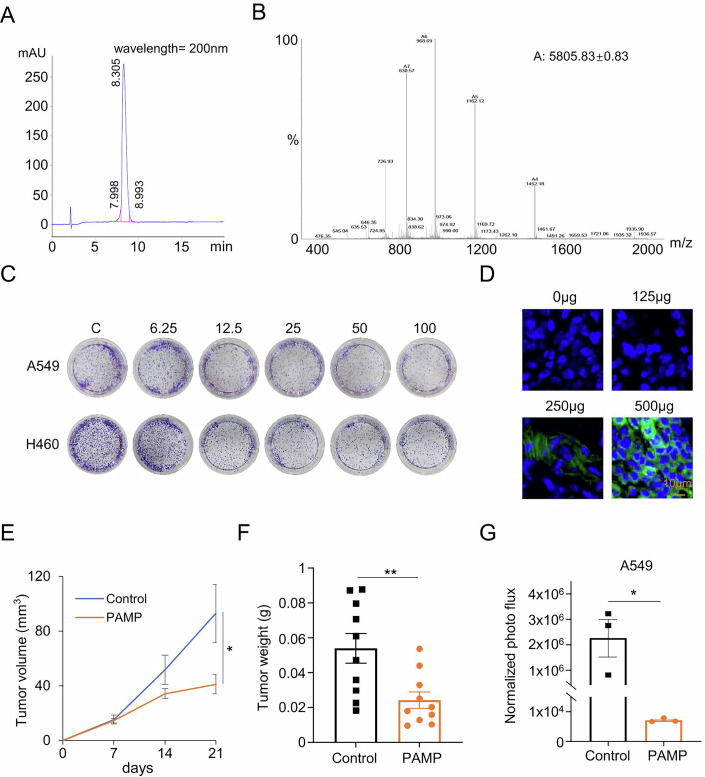
Synthetic PAMP inhibits LUAD growth. (**A**,** B**) Purity and molecular weight analysis of chemically synthesized PAMP peptide using HPLC (**A**) and LC-MS (**B**). (**C**) Colony formation assays evaluating cell proliferation in LUAD cells treated with synthetic PAMP. (**D**) FITC-labeled synthetic PAMP was injected into the peritoneal cavity of mice, and then FITC fluorescence in the lung tissue was detected using a fluorescence microscope; nuclei were stained with DAPI (blue). (**E**,** F**) Tumor growth was monitored every 7 days to generate a growth curve, and tumor weight was measured after surgical excision (*n* = 5, **E**: *P* = 0.048; **F**: *P* = 0.036). (**G**) Bioluminescence imaging was used to quantify lung colonization in PAMP-treated and control nude mice (*n* = 3, *P* = 0.038). Data were shown as mean ± SEM in (**E**–**G**). *P < 0.05; two-tailed unpaired *t*-test. Source data are available online for this figure.

**Figure 7:**
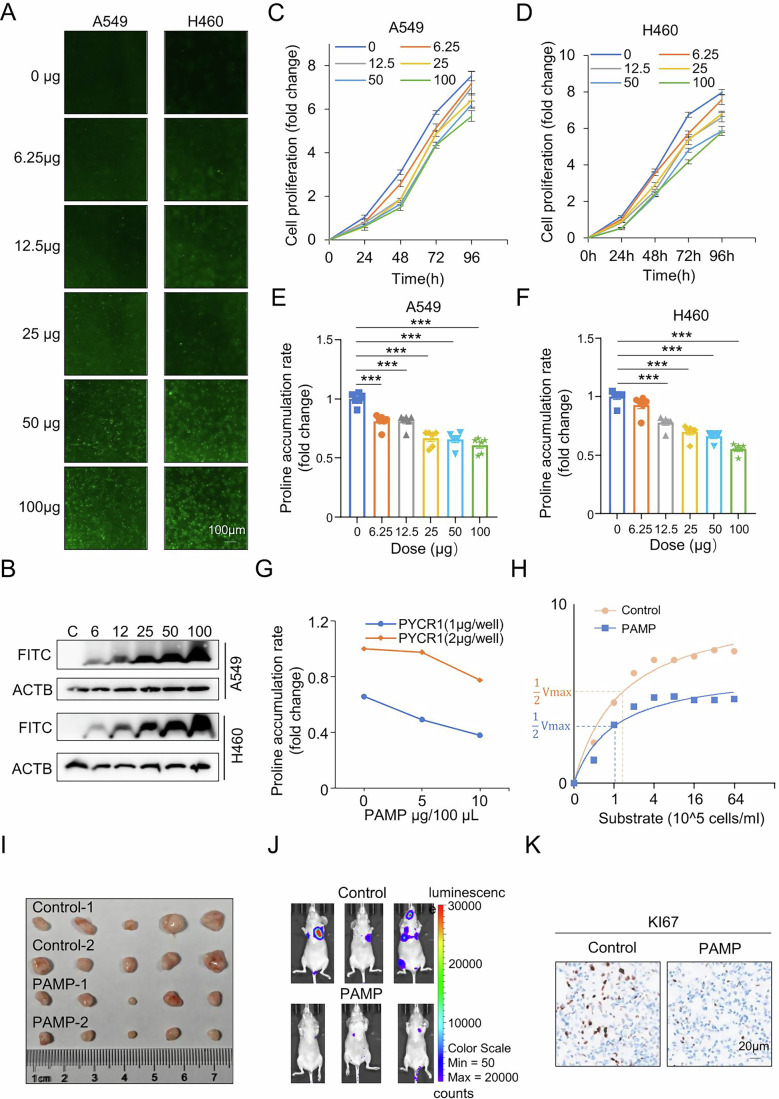
Synthetic PAMP inhibits proline accumulation and LUAD cell proliferation in vitro and in vivo. (**A**) FITC-labeled synthetic PAMP was added to the cell culture medium for 24 h, and FITC fluorescence was detected using a fluorescence microscope. (**B**) Western blot analysis of FITC-labeled PAMP fusion protein levels using anti-FITC antibody. (**C**,** D**) CCK-8 assays assessing cell proliferation in LUAD cells treated with synthetic PAMP. Significant differences were observed at PAMP concentrations >25 μg in A549 cells and >12.5 μg in H460 cells (*n* = 5, *P* (25) = 0.018; *P* (50) = 0.001; *P* (100) = 5E-04; H460: *P* (12.5) = 0.001; *P* (25) = 4.43E-04; *P* (50) = 8.65E-05; *P* (100) = 8.77E-07). Cell proliferation is presented as fold change relative to the 0 h time point, which served as the reference condition. (**E**,** F**) Proline levels measured in LUAD cells treated with synthetic PAMP (*n* = 5, A549: *P* (6.25) = 1.41E-04; *P* (12.5) = 1.31E-04; *P* (25) = 2.11E-06; *P* (50) = 1.21E-06; *P* (100) = 3.82E-07; H460: *P* (6.25) = 0.1; *P* (12.5) = 5.04E-05; *P* (25) = 5.75E-06; *P* (50) = 4.57E-07; *P* (100) = 3.81E-08). Proline accumulation is presented as fold change relative to the condition without PAMP treatment, 0 μg dose, which was used as the reference condition and set to 1. (**G**) In vitro enzymatic assay demonstrating the inhibitory effect of synthetic PAMP on PYCR1-mediated proline synthesis (*n* = 6). Proline accumulation is presented as fold change relative to the condition without PAMP treatment, 0 μg/100 μL, which was used as the reference condition and set to 1. (**H**) Enzyme kinetic assays evaluating PYCR1 enzymatic activity. Following PAMP treatment, the ${V}_{\max }$ decreased from 10.12 to 6.394, and the ${K}_{m}$ decreased from 2.401 to 2.042. PYCR1 activity was measured indirectly by quantifying proline levels (*n* = 5). (**I**) Nude mice were subcutaneously injected with wild-type A549 cells and randomly divided into PAMP-treated and control groups. Synthetic PAMP (500 μg) was administered intraperitoneally every 2 days. Representative tumors from at least five independent experiments were surgically excised (*n* = 5). (**J**) Bioluminescence imaging of lung colonization in nude mice intravenously injected with A549 cells via the lateral tail vein. Imaging of the PAMP-treated and control groups was captured after 28 days. (**K**) Representative images of Ki67 immunohistochemical staining in lung tissue of PAMP-treated and control nude mice. Data were shown as mean ± SEM in (**C**–**H**). ^*^P < 0.05; ^**^P < 0.01; ^***^P < 0.001; two-tailed unpaired *t*-test. Source data are available online for this figure.

High-performance liquid chromatography (HPLC) and liquid chromatography-mass spectrometry (LC-MS) confirmed the purity and molecular weight of the synthesized PAMP peptides (Fig. EV7A,B). FITC labeling demonstrated efficient uptake of synthetic PAMP peptides by LUAD cells within 24 h (Fig. 7A,B). In vitro functional assays showed that PAMP peptides dose-dependently inhibited LUAD cell proliferation and proline accumulation (Figs. 7C–F and EV7C). Additionally, in vitro enzymatic assays revealed that the synthesized PAMP significantly inhibited the PYCR1-catalyzed enzymatic reaction involved in proline synthesis (Fig. 7G). Enzyme kinetic analysis further indicated that PAMP treatment reduced the Vmax and Km of PYCR1, suggesting that PAMP acts as a mixed-type inhibitor that impairs both substrate binding and catalytic efficiency, thereby suppressing proline synthesis (Fig. 7H).

To evaluate PAMP’s therapeutic potential in vivo, we established LUAD tumor models in nude mice and administered synthetic PAMP. Immunofluorescence analysis confirmed successful delivery of synthetic PAMP to the mouse lung at a dose of 500 μg (Fig. EV7D). Subcutaneous tumor formation assays showed that synthetic PAMP significantly inhibits LUAD tumor growth (Figs. 7I and EV7E,F). In the lung cancer model, PAMP-treated mice exhibited significantly reduced fluorescence signal compared to untreated controls, accompanied by decreased Ki67 expression (Figs. 7J,K and EV7G). Collectively, these findings suggest that synthetic PAMP effectively suppresses proline metabolism and tumor progression, highlighting its potential as a promising therapeutic strategy for LUAD.

## Discussion

Advances in bioinformatics and next-generation sequencing have revealed that lncRNAs can encode functional micropeptides, typically fewer than 100 amino acids, through sORFs (Choi et al, 2019). Increasing evidence suggests that these lncRNA-derived peptides play crucial roles in cancer biology, influencing tumor progression, metabolic regulation, and therapeutic resistance (Kesner et al, 2023; Yi et al, 2024). In this study, we identified and functionally characterized PAMP (proline-associated micropeptide), a 48-amino acid micropeptide encoded by PSMA3-AS1, which acts as a tumor suppressor in LUAD. Of note, previous studies have reported that the PSMA3-AS1 functions as an oncogenic lncRNA, promoting the proliferation of A549 and H460 cells (Kan et al, 2023). This seemingly contradictory observation merits careful consideration. In our study, we employed two distinct siRNAs in loss-of-function experiments to minimize off-target effects, and integrated analyses of multiple independent clinical cohorts consistently demonstrated that PSMA3-AS1 is downregulated in LUAD tissues, supporting its tumor-suppressive role in this disease context. More importantly, our findings reveal that PSMA3-AS1 encodes a functional micropeptide, PAMP. PAMP expression was significantly downregulated in LUAD tissues and positively correlated with a favorable patient prognosis. Functional assays further demonstrated that PAMP inhibits LUAD cell proliferation in vitro and suppresses tumor growth in vivo.

Existing studies on lncRNA-encoded micropeptides primarily focus on their protein-binding interaction, which mediates their functional roles (Wu et al, 2020). For instance, MIAC, a micropeptide encoded by LINC00473, suppresses renal cell carcinoma progression by interacting with AQP2 and inhibiting EREG/EGFR signaling (Li et al, 2022). Similarly, MTLN, a mitochondrial peptide encoded by LINC00116, modulates mitochondrial function via interaction with NADH-dependent cytochrome b5 reductase (Chugunova et al, 2019). Expanding on this paradigm, our study identified PYCR1, a key enzyme in proline biosynthesis, as a direct binding partner of PAMP. Co-IP assays confirmed that PAMP binds to PYCR1, thereby inhibiting proline synthesis in a PYCR1-dependent manner. Molecular docking simulations and affinity experiments further revealed that the PAMP-F16 residue and the PYCR1-N123 residue are critical for this interaction. Because N123 resides within the Rossmann fold domain of PYCR1, PAMP binding at this site may induce conformational perturbations that compromise catalytic function without affecting protein abundance. Future structural studies, such as X-ray crystallography, NMR, or cryo-EM, will be needed to define the underlying mechanism more precisely. Together, these findings suggest that PAMP disrupts proline accumulation and impairs LUAD cell proliferation by functionally restraining PYCR1.

The management of LUAD remains a significant challenge due to the scarcity of effective therapeutic targets, underscoring the urgency for innovative treatment strategies (Gillette et al, 2020). Antitumor peptides have emerged as a promising avenue for cancer therapy owing to their unique physicochemical properties, including high specificity, low toxicity, and favorable pharmacokinetics (Liu et al, 2023; Muttenthaler et al, 2021). Recent studies have identified several ncRNA-derived antitumor peptides with promising therapeutic potential in colorectal cancer and TNBC (Wang et al, 2020). Consistent with this emerging concept, our findings demonstrate that PAMP inhibits proline synthesis, suppresses LUAD cell proliferation, and reduces tumor growth in nude mouse models, highlighting its therapeutic relevance. Future translational efforts may further improve the utility of PAMP through optimized delivery approaches. For example, lipid nanoparticle (LNP)-based systems may provide an effective platform for PAMP administration. Surface modification of LNPs with cyclic RGD peptides could enhance targeting to tumor vasculature and αvβ3 integrin-expressing tumor cells, whereas incorporation of cell-penetrating peptides such as TAT may improve intracellular uptake (Luo et al, 2025; Ren et al, 2025). Together, these strategies may increase the delivery efficiency and translational potential of PAMP-based therapy.

Metabolic interventions targeting cancer-specific pathways are gaining traction, with glycolytic enzyme inhibitors, glutaminase inhibitors, and mitochondrial disruptors being actively developed (Li et al, 2023). Although several lncRNA-encoded peptides have been reported to regulate cellular metabolism, their mechanisms generally involve modulation of alternative splicing (e.g., HOXB-AS3), activation of enzyme complexes (e.g., ASAP), or regulation of upstream signaling pathways (e.g., XBP1SBM, LncRIM) (Ge et al, 2021; Huang et al, 2017; Ma et al, 2025; Wu et al, 2022). Proline metabolism, a critical axis in tumor metabolism, is well-established to promote tumorigenesis and cancer progression (Mayneris-Perxachs et al, 2022). By contrast, our study identifies PAMP as a direct and specific inhibitor of the metabolic-enzyme PYCR1. PAMP physically associates with PYCR1 and suppresses its catalytic activity, thereby reducing proline biosynthesis.

This mechanism distinguishes PAMP from conventional small-molecule inhibitors of proline metabolism, such as PYCR1 or PRODH inhibitors, which generally target enzyme active sites and may be limited by suboptimal selectivity, off-target effects, or the emergence of resistance (Chen et al, 2024). As an endogenous micropeptide, PAMP may engage regulatory mechanisms that are less accessible to small molecules, including modulation of protein-protein interactions or allosteric interference with PYCR1 function. In addition, because PAMP is a naturally occurring peptide, it may offer potential advantages in biocompatibility and safety. Thus, PAMP represents a distinct and potentially valuable form of metabolic intervention that suppresses LUAD growth by targeting proline metabolism.

In summary, we identified PAMP as a previously uncharacterized lncRNA-encoded micropeptide that is significantly downregulated in LUAD and associated with favorable patient prognosis. Mechanistically, PAMP directly binds PYCR1 and suppresses proline biosynthesis, thereby inhibiting LUAD cell proliferation and tumor progression in vitro and in vivo (Fig. 7J). These findings advance our understanding of lncRNA-encoded micropeptides in cancer metabolism and nominate the PAMP-PYCR1 axis as a promising candidate for therapeutic intervention and biomarker development in LUAD.

## Methods

**Table:** Reagents and tools table

Reagent/resource	Reference or source	Identifier or catalog number
**Experimental mouse models and cell lines**		
BALB/c-Nude	GemPharmatech	Strain NO. D000521
A549	ATCC	TCHu150
H460	ATCC	SCSP-584
293 T	ATCC	SCSP-502
**Recombinant DNA**		
PLVX-CMV-PAMP-3x FLAG-Puro	Miaoling	NA
PLVX-CMV-PAMP-GFP-Puro	Miaoling	NA
lentiCRISPRv2-PAMP sgRNA	Miaoling	NA
lentiCRISPRv2-PYCR1 sgRNA	Miaoling	NA
**Antibodies**		
GAPDH	Proteintech	60004-1-Ig
Flag tag	Sigma-Aldrich	F1804
GFP tag	Invitrogen	MA5-15256
ACTB	Abclonal	AC026
PYCR1	Proteintech	13108-1-AP
HA tag	Cell Signaling Technology	3724
FITC	Abclonal	A22444
KI67	Cell Signaling Technology	9129
PAMP	This study	NA
**Oligonucleotides and other sequence-based reagents**		
PSMA3-AS1 siRNA1	RiboBio	GGUUAAGAGCAAUCAGCAATT
PSMA3-AS1 siRNA2	RiboBio	GCCUAGUUAUAAUGAUAUATT
GAPDH-F	Tsingke	AGAAGGCTGGGGCTCATTTG
GAPDH-R	Tsingke	AGGGGCCATCCACAGTCTTC
Actin-F	Tsingke	GGACTTCGAGCAAGAGATGG
Actin-R	Tsingke	AGCACTGTGTTGGCGTACAG
PSMA3-AS1-F	Tsingke	TTGTGATGTGCGAGAAAAAGT
PSMA3-AS1-R	Tsingke	GTCGTAGTCAATGGAGAGAGG
NEAT1-F	Tsingke	CCAGTTTTCCGAGAACCAAA
NEAT1-R	Tsingke	ATGCTGATCTGCTGCGTATG
PYCR1-F	Tsingke	ACGCACGCCCAGGTGGAGGACG
PYCR1-R	Tsingke	TGATGAGCAGGGAGCGGAAGCC
**Chemicals, enzymes and other reagents**		
Puromycin	Invivogen	ant-pr-1
D-Luciferin	Beyotime	ST196-500mg
PYCR1 protein (enzyme)	CUSABIO	CSB-EP019115
Proline Content Assay Kit	Sangon Biotech	D799575
PARIS™ Kit	Life Technologies	AM1921
synthesized PAMP	Yuan-peptide	NA
**Software**		
Hisat2	Steven Salzberg lab	v2.1.0
DEseq2	Bioconductor	v1.42.1
Stringtie2	Steven Salzberg lab	v2.2.1
AlphaFold2	DeepMind	v2.2.0
KF Slide OS	KFBIO	v1.0.8

### Tissue specimens

Tissue microarrays comprising 74 paired LUAD samples and corresponding adjacent tissues for immunohistochemistry experiments were procured from Shanghai ZhuoLi Biotechnology Company. Additionally, five paired LUAD and adjacent tissues were collected from the tissue bank of Sir Run Run Shaw Hospital of Zhejiang University for Western blot analysis. All tissue specimens were collected with informed consent at the time of diagnosis, prior to any treatment. The study protocol was approved by the Institutional Review Boards of Sir Run Run Shaw Hospital, Zhejiang University. The study involving human tissue specimens was conducted in accordance with the ethical principles of the World Medical Association Declaration of Helsinki and the Department of Health and Human Services Belmont Report.

### Cell lines and cell culture

LUAD cell lines A549, H460, along with 293T, were purchased from ATCC (American Type Culture Collection, Manassas, VA, USA). A549 and H460 were authenticated by STR profiling, while 293T was not; none of the cell lines were tested for mycoplasma contamination. Cells were cultured with DMEM or RPMI 1640 (Gibco, Carlsbad, CA, USA) supplemented with 10% fetal bovine serum (FBS, Thermo Fisher Scientific, Rockford, IL, USA) under standard conditions.

### Reagents and antibodies

Details regarding the reagents and antibodies used in this study are provided in the reagents and tools table. The anti-PAMP antibody (1 mg/ml) was generated by immunizing New Zealand rabbits with a synthetic peptide (sequence: C-FLCGRPPPFPPGQ, corresponding to amino acids 16–28 of human PAMP; length: 13 amino acids) conjugated to Keyhole Limpet Hemocyanin (KLH) via an N-terminal cysteine residue. Antisera were purified by immuno-affinity purification (DeTai, Shanghai, China). For immunoprecipitation, the anti-PAMP antibody was used at a concentration of 2 µg per 500 µL of cell lysate (or at a 1:200 dilution). For western blotting, the antibody was diluted 1:1000–1:2000 in dilution buffer.

### siRNA and transfection

siRNAs were obtained from GenePharma (Shanghai, China) and transfected using GenMute^TM^ reagent (SignaGen™ Laboratories, Rockville, MD, USA) according to the manufacturer’s protocol. The specific siRNA sequences are listed in the reagents and tools table.

### RNA isolation and quantitative real-time PCR analysis

Total RNA was extracted using TRIzol RNA Isolation Reagents (Invitrogen, Carlsbad, CA, USA). Extracted RNA was reverse transcribed into cDNA using HiScript® II Reverse Transcriptase (Vazyme, Nanjing, China) following the manufacturer’s instructions. Quantitative real-time PCR (qRT-PCR) was performed using an SYBR Green PCR Mix Kit (Vazyme). The primers used in this study are listed in the reagents and tools table.

### Cytoplasmic and nuclear RNA isolation

Cytoplasmic and nuclear fractions were isolated using the PARIS Kit (Life Technologies, CA, USA) following the manufacturer’s protocol. Briefly, cells were lysed using a cell fractionation buffer and incubated on ice for 10 min. After centrifugation at 500×*g* for 5 min, the supernatant was collected as the cytoplasmic fraction, while the remaining nuclear pellets were lysed with a cell disruption buffer to obtain the nuclear fraction. RNA from both fractions was extracted and analyzed via qRT-PCR.

### Plasmid construction and cell transfection

For PAMP overexpression, the full-length human PAMP sequence was synthesized by PCR and cloned into a PLVX-CMV-FLAG-Puro vector (Miaoling, Wuhan, China). To construct stably PAMP and PYCR1 knockout LUAD cell lines, sgRNAs were synthesized and cloned into the lentiCRISPRv2 vector (Addgene, Watertown, MA, USA). For lentivirus production, 293T cells were co-transfected with recombinant plasmids along with packaging plasmids pVSVG (Addgene) and psPAX2 (Addgene). After 48 h, lentiviruses were harvested and concentrated. A549 and H460 cells were then transfected with the concentrated lentiviruses to establish stable knockout cell lines. The sgRNA sequences used in this study are listed in the reagents and tools table.

### Western blot

Total protein was extracted from cells using RIPA buffer (Beyotime) supplemented with a proteinase inhibitor cocktail (PMSF, Beyotime). Protein concentrations were quantified using a PierceTM BCA Protein Assay Kit (Thermo Scientific). Equal amounts of protein were separated by 10% SDS–PAGE or 15.5% Tricine gel and transferred onto PVDF membranes. Membranes were blocked with 5% skimmed milk, incubated with primary antibodies at 4 °C overnight, and subsequently incubated with secondary antibodies at room temperature for 2 h. Protein bands were detected using Pierce ECL Western Blotting Substrate reagent (Thermo Scientific).

### Immunofluorescence staining

Cultured cells were fixed with 4% formaldehyde for 10−15 min and then blocked with 3% BSA and 0.1% Triton X-100 in PBS for 20 min at room temperature. Immunostaining was performed using anti-PAMP (this study), and anti-FLAG antibodies. Nuclei were counterstained with DAPI. Images were captured using a Zeiss fluorescence photomicroscope.

### Immunohistochemistry

For immunohistochemical (IHC) staining, tissue sections were deparaffinized in xylene and rehydrated through a graded ethanol series. Antigen retrieval was conducted using Tris-EDTA buffer (pH 9.0) in a microwave for 20 min. Sections were then incubated with 3% hydrogen peroxide in distilled water to quench endogenous peroxidase activity and blocked with 1% bovine serum albumin (BSA) to prevent non-specific binding. The sections were incubated overnight at 4 °C with an anti-PAMP antibody (1:100 dilution). After several washes, the sections were treated with horseradish peroxidase (HRP)-conjugated secondary antibody for 30 min at room temperature, followed by staining with 3,3’-diaminobenzidine tetrahydrochloride (DAB). Images were captured using a Zeiss AXIO Observer A1 microscope, and PAMP expression intensity was quantified using KFSlideOS software.

### Cell proliferation and colony assays

To assess cell variability, cells were seeded into 96-well plates (800–1000 cells/well), and proliferation was measured using the Cell Counting Kit-8 (MCE, Monmouth Junction, NJ, USA). For colony formation assays, 1000 transfected cells were seeded in 12-well plates and cultured for ~10 days. Colonies were fixed with methanol, stained with 0.1% crystal violet, and washed with PBS.

### Proline measurement

Relative proline levels were measured using the Proline Content Assay Kit (Sangon Biotech, D799575). In brief, cells were homogenized in extraction buffer, and the supernatant was mixed with corresponding reagents, followed by incubation at 100 °C for 30 min. Absorbance values were measured at 520 nm using a microplate reader.

### In vitro enzyme assay

The inhibitory effect of synthesized PAMP on PYCR1-catalyzed proline synthesis was evaluated using an in vitro enzyme activity assay. The assay was performed in 96-well plates with a 50 µL reaction volume per well. Cell lysates, prepared by sonicating cells in ice-cold PBS or Tris-HCl buffer, followed by centrifugation (12,000 × *g*, 15 min, 4 °C) and dilution, served as the substrate source at a concentration equivalent to 1.5 × 10⁶ cells per well. Recombinant PYCR1 enzyme was added at 1 or 2 µg per well, and PAMP was tested at concentrations of 0, 5, or 10 µg per well as the inhibitor. Reaction mixtures were incubated at 37 °C for 60 min and terminated by adding an equal-volume mixture of glacial acetic acid and chromogen reagent, followed by boiling for 30 min. After cooling to room temperature, absorbance at 520 nm was measured, and proline content was quantified based on a standard curve.

### Co-immunoprecipitation and mass spectrometry

Whole-cell lysates were prepared using lysis buffer for immunoprecipitation (IP). Lysates were incubated with 2 mg of the specified antibody or normal rabbit IgG at room temperature for 2 h. Antigen-antibody complexes were then incubated with Protein A/G magnetic beads (Bimake, Houston, TX, USA) for an additional 2 h at room temperature. Beads were washed three times with wash buffer (50 mM Tris-HCl, 150 mM NaCl, 0.5% Tween-20, pH 7.5). Finally, the immunoprecipitates were resuspended in SDS loading buffer, boiled, and analyzed by Western blotting.

### RNA-seq and data analysis

Total RNAs was extracted from three independent PAMP-knockout and control A549 cell groups for RNA-seq analysis. RNA libraries were prepared for sequencing using the HiSeq X10 sequencer (Mingma, Shanghai, China). RNA-seq data processing followed previously published protocols (Qian et al, 2021). In brief, raw sequence reads were trimmed to remove adapter sequence, and then aligned to the human reference genome (hg38) using Hisat2. Transcript assembly and gene expression quantification in Fragments Per Kilobase of transcript per Million mapped reads (FPKM) were performed using Stringtie2. Differentially expressed genes were identified using DEseq2, with a false discovery rate (FDR) threshold of <0.05.

### Metabolomics analysis via UPLC-MS

Cells were extracted using 800 mL of 80% methanol, followed by vortexing for 1 min and ultrasonication for 30 min at 4 °C. Extracts were incubated at −40 °C for 1 h, vortexed for 30 s, and incubated at 4 °C for 30 min. Samples were centrifuged at 12,000 rpm for 15 min at 4 °C. Supernatants were concentrated to remove organic solvents and water, and reconstituted in 100 mL of 80% methanol for ultra-performance liquid chromatography-mass spectrometry (UPLC-MS) analysis. UPLC-MS analysis was performed using an ACQUITY UPLC system (Waters) coupled with a Triple Quad 5500 MS (SCIEX, Applied Biosystems).

### Animal experiments

To establish a xenograft tumor mouse model, A549 cells were subcutaneously inoculated into the flanks of 5-week-old female BALB/c-nude mice. The mice were purchased from GemPharmatech Co., Ltd. (Nanjing, China). All animals were housed under specific pathogen-free conditions in a temperature-controlled (22 ± 2 °C) and humidity-controlled (50 ± 10%) environment with a 12 h light/dark cycle, and had free access to sterile food and water. Tumor growth was monitored over time, and tumor volume was measured and calculated using the formula (length × width²)/2 at predetermined intervals following inoculation. For the lung colonization model, 1 × 10^6^ A549 cells were injected intravenously into the tail vein of female BALB/c-nude mice. To evaluate the effect of synthetic PAMP on tumor proliferation, synthetic PAMP peptides were administered intraperitoneally at a dose of 500 μg every 2 days. All animal experiments were approved by the Animal Care Committee of Zhejiang University, Hangzhou, China (No. ZJU20250416) and were performed in strict accordance with the guidelines for the care and use of laboratory animals.

### Ethics approval

The Ethics Committee of Sir Run Run Shaw Hospital of Zhejiang University School of Medicine (Hangzhou, China) reviewed and approved this study. Human subjects involved in this study were conducted according to the International Ethical Guidelines for Biomedical Research. All participants enrolled at Sir Run Run Shaw Hospital provided informed consent to take part in the research (Approval No.: Sir Run Run Shaw Hospital Ethics Approval 2025 Research No. 0015).

### Statistical analysis

Data were presented as mean ± SEM of at least three independent experiments. Sample sizes (*n*) for each experiment are indicated in the corresponding figure legends. For animal studies, mice were randomly assigned to experimental groups, and investigators were blinded to group allocation during tumor measurement and data analysis. No exclusion criteria were predefined; all animals that completed the study were included in the final analysis, except for those that died prematurely due to causes unrelated to the experimental treatment (e.g., injection accidents), which were excluded from the analysis. Statistical analyses were performed with the student’s *t*-test, with *P* value <0.05 considered significant. Statistical significance is represented in figures as follows: 0.01 < *P <  0.05; 0.05 < **P < 0.01; 0.01 < ***P < 0.001.

## Supplementary information

Table EV1

Table EV2

Peer Review File

Source data Fig. 1

Source data Fig. 2

Source data Fig. 3

Source data Fig. 4

Source data Fig. 5

Source data Fig. 6

Source data Fig. 7

Figure EV1 Source Data

Figure EV2 Source Data

Figure EV3 Source Data

Figure EV4 Source Data

Figure EV5 Source Data

Figure EV6 Source Data

Figure EV7 Source Data

Expanded View Figures

## Data Availability

The RNA-seq data generated in this study have been deposited in the GSA-Human database under accession number HRA017134 (BioProject: PRJCA039014) (Shared URL: https://ngdc.cncb.ac.cn/gsa-human/s/yg0RL9n2). The metabolomics data have been deposited in the OMIX database of the National Genomics Data Center (NGDC) under accession number OMIX016869 (BioProject: PRJCA064275) (Shared URL: https://download.cncb.ac.cn/OMIX/OMIX016869/). The source data of this paper are collected in the following database record: biostudies:S-SCDT-10_1038-S44321-026-00460-2.
