# Supplementary figures and images for: PAMP orchestrates proline metabolic rewiring to suppress LUAD via PYCR1 inhibition (part 1 of 2)
Source: EMBO Mol Med. 2026 Jun 9;18(7):2867–95. doi: 10.1038/s44321-026-00460-2 (PMC13365495; doi:10.1038/s44321-026-00460-2)

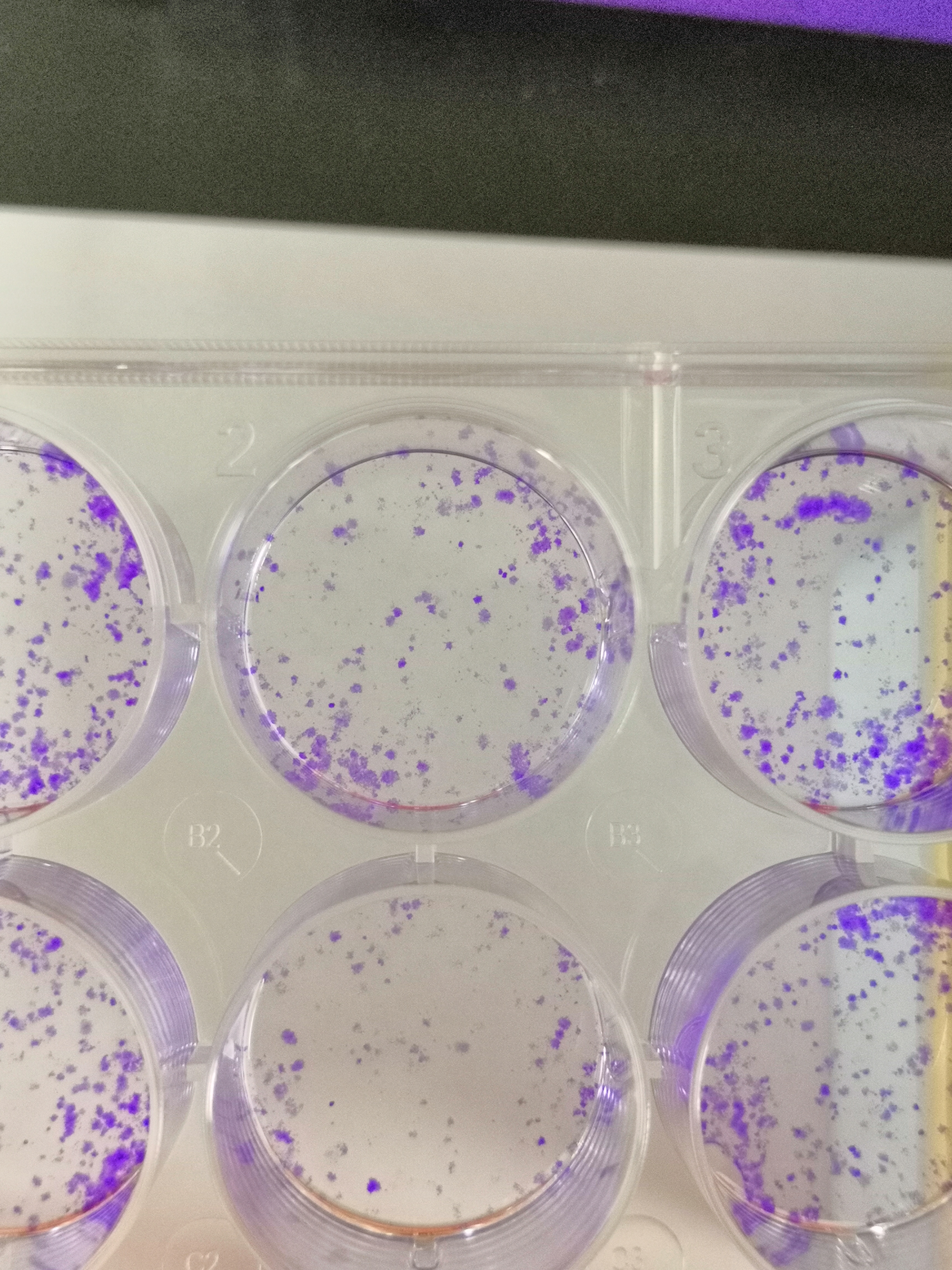

Supplement: Supplementary file 4 — Source data Fig. 1 [file 44321_2026_460_MOESM4_ESM.zip › Source data Figure1/FIG 1I/A549-NC.jpg]

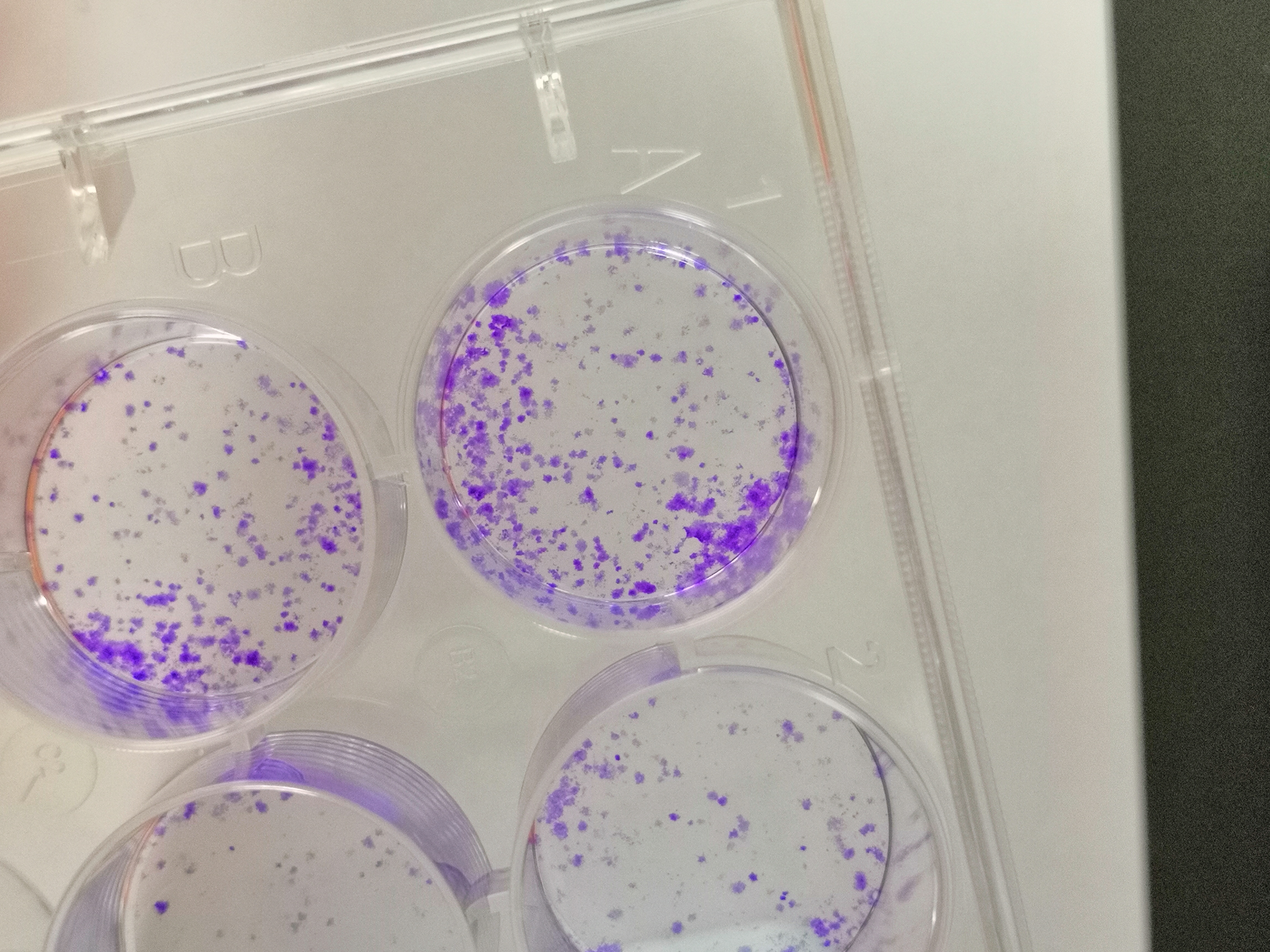

Supplement: Supplementary file 4 — Source data Fig. 1 [file 44321_2026_460_MOESM4_ESM.zip › Source data Figure1/FIG 1I/A549-SIPSMA-1.jpg]

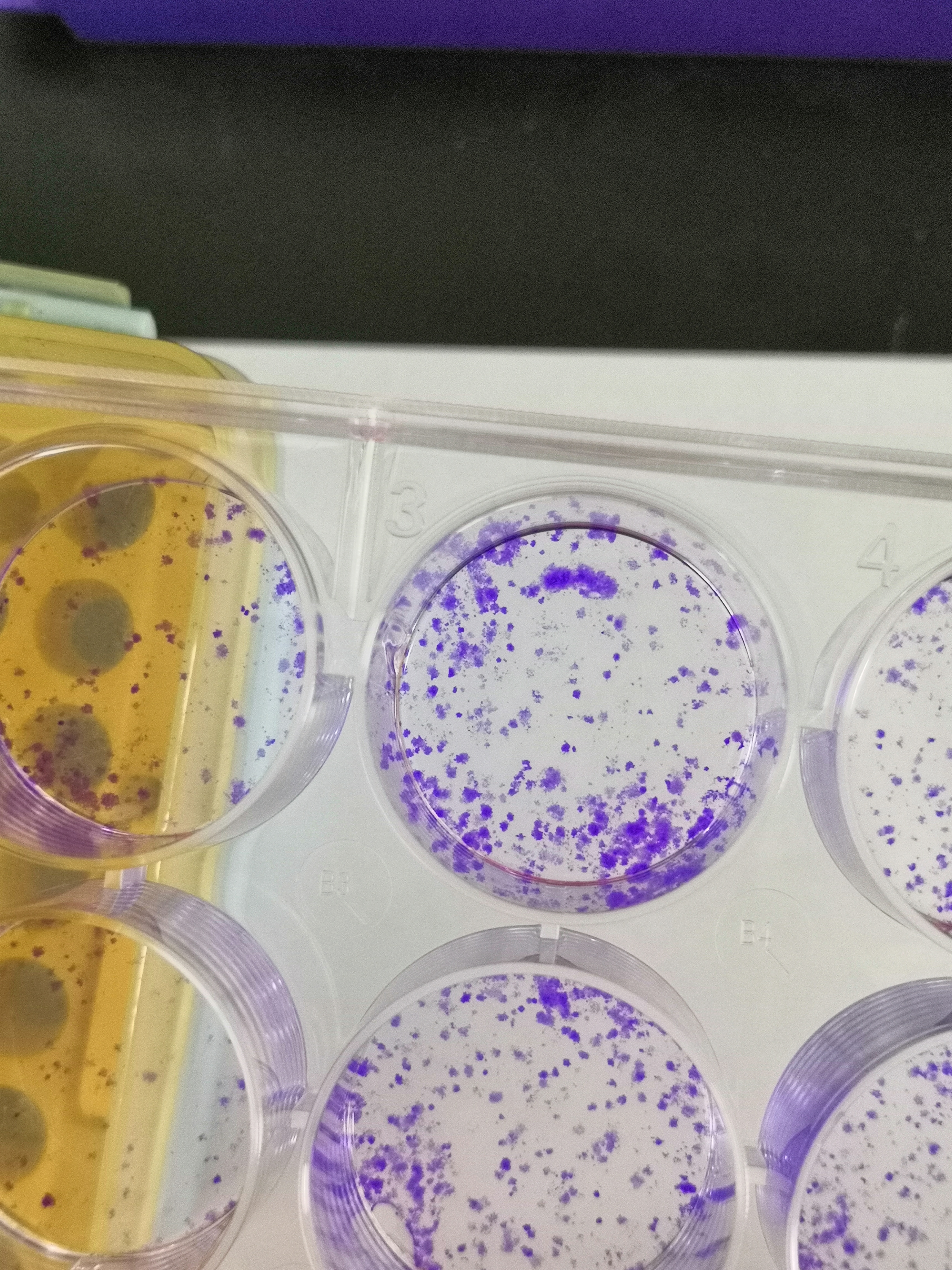

Supplement: Supplementary file 4 — Source data Fig. 1 [file 44321_2026_460_MOESM4_ESM.zip › Source data Figure1/FIG 1I/A549-SIPSMA-2.jpg]

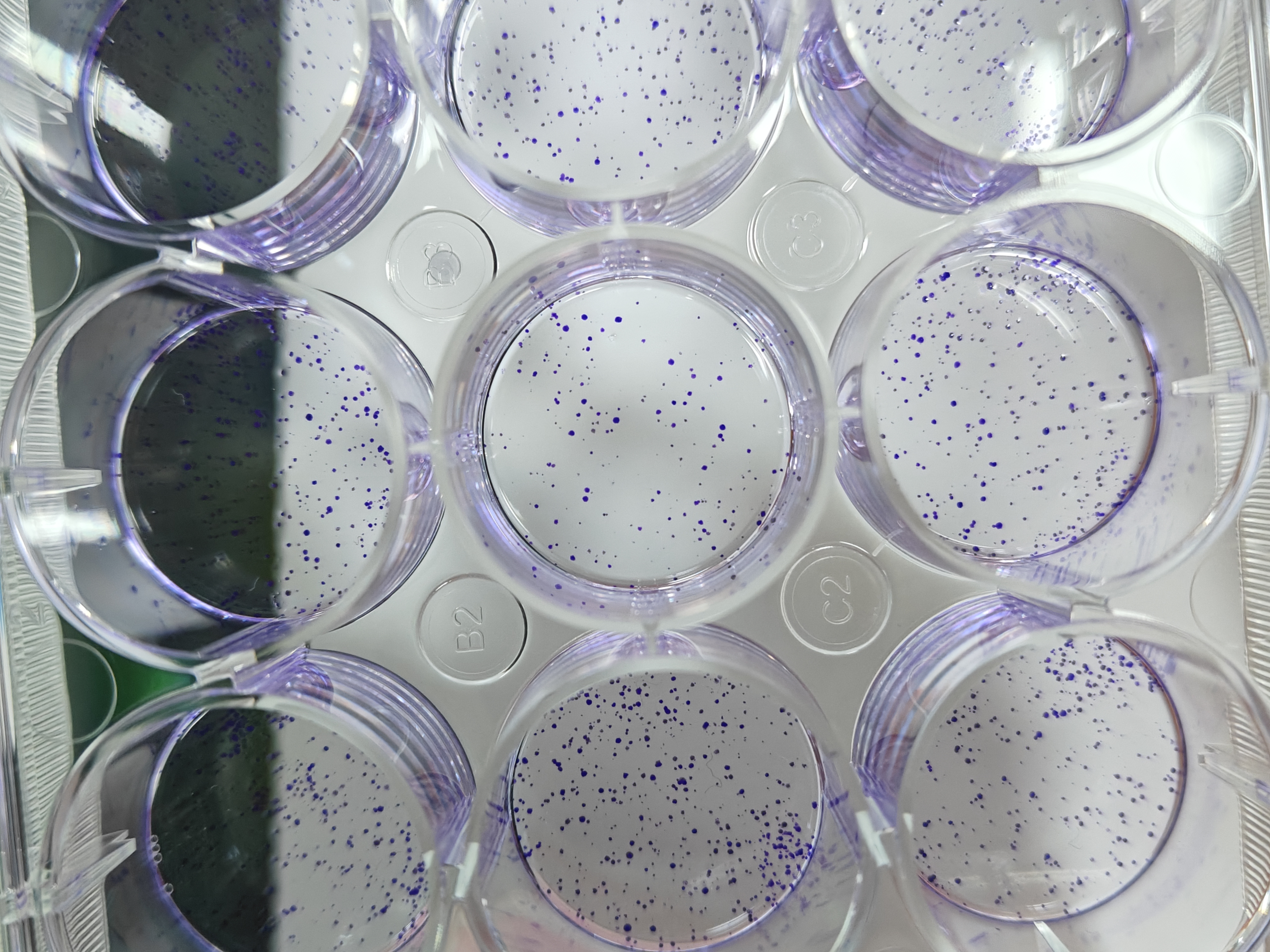

Supplement: Supplementary file 4 — Source data Fig. 1 [file 44321_2026_460_MOESM4_ESM.zip › Source data Figure1/FIG 1I/H460-NC.jpg]

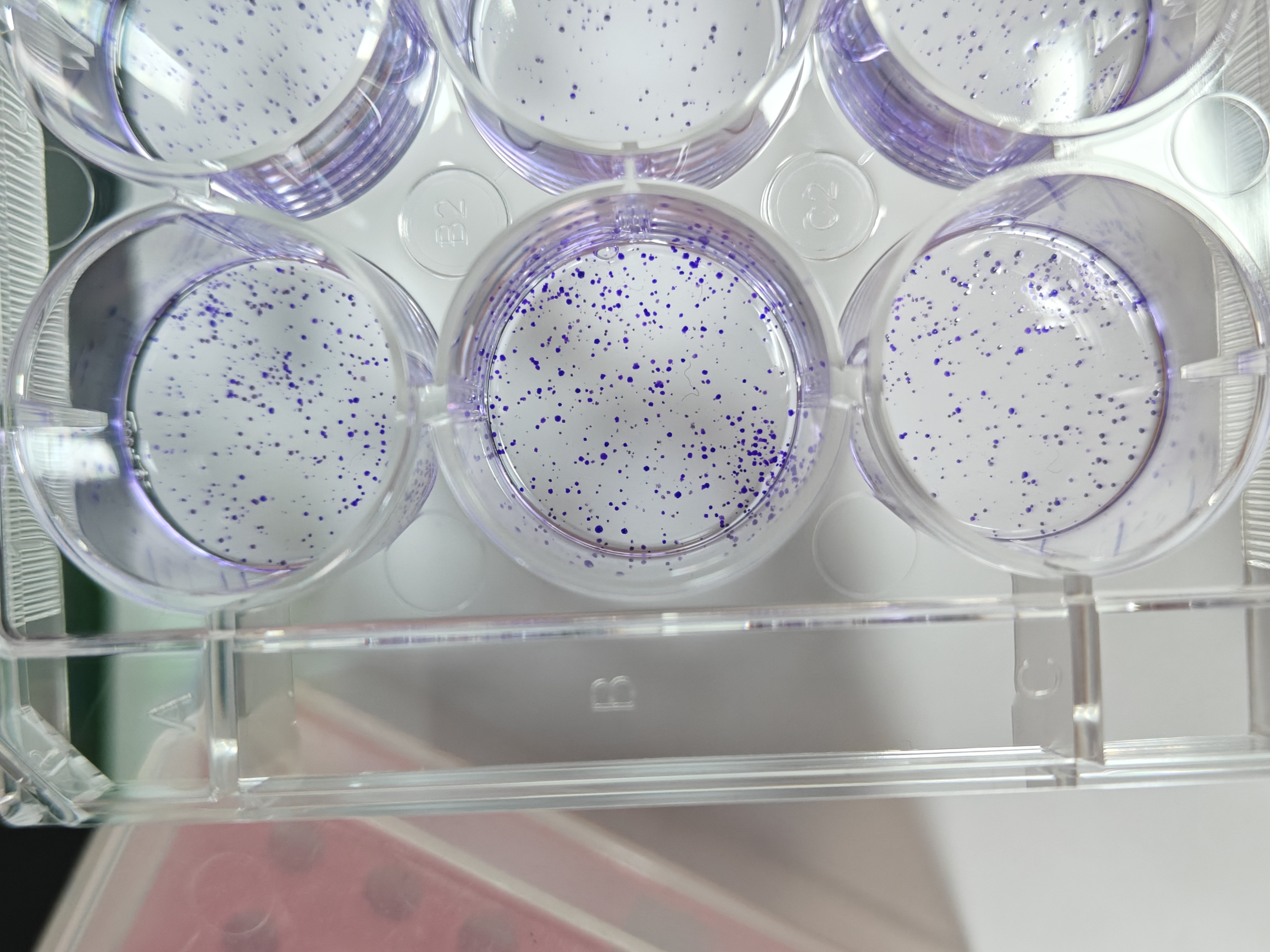

Supplement: Supplementary file 4 — Source data Fig. 1 [file 44321_2026_460_MOESM4_ESM.zip › Source data Figure1/FIG 1I/H460-SIPSMA-1.jpg]

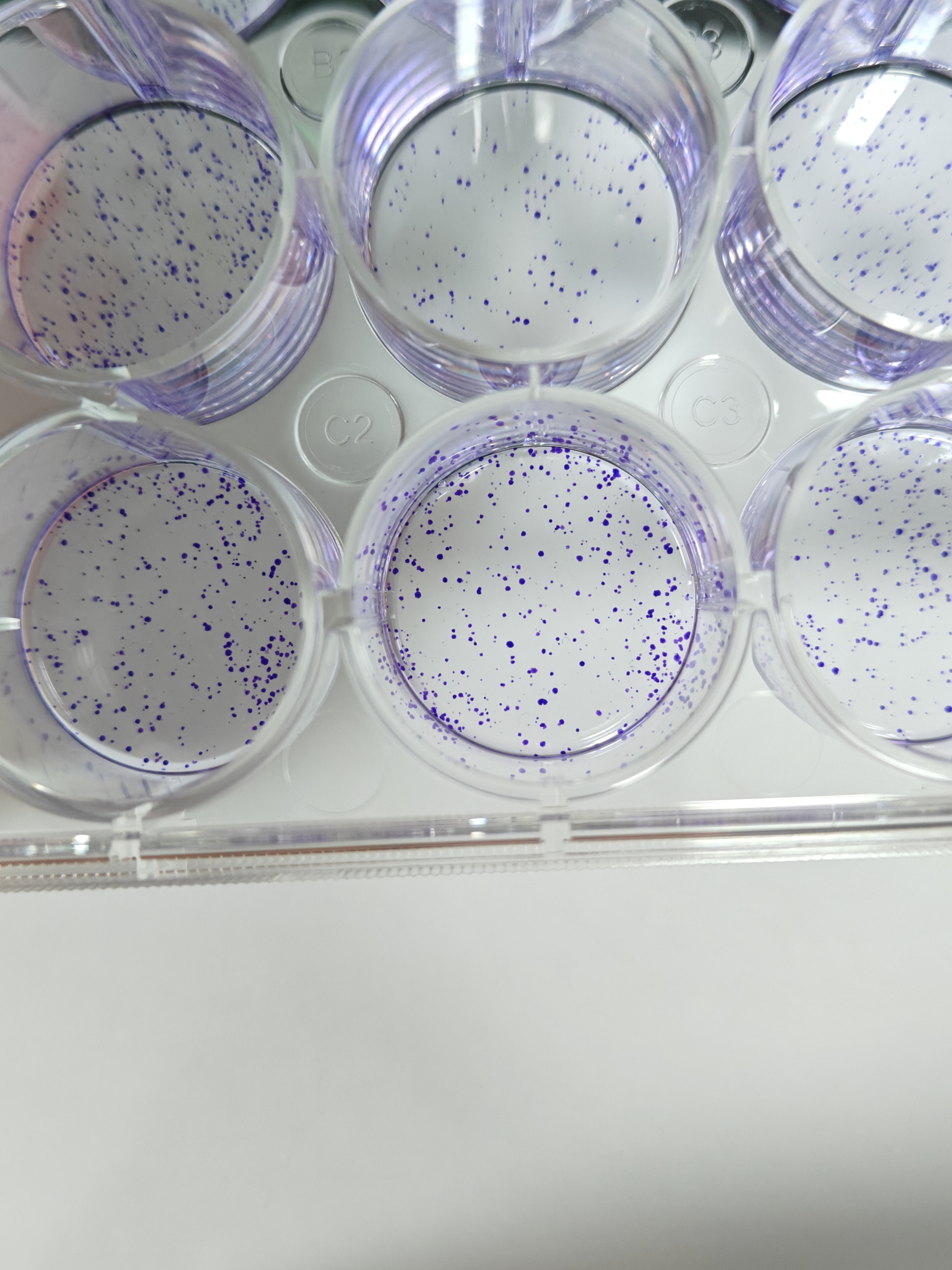

Supplement: Supplementary file 4 — Source data Fig. 1 [file 44321_2026_460_MOESM4_ESM.zip › Source data Figure1/FIG 1I/H460-SIPSMA-2.jpg]

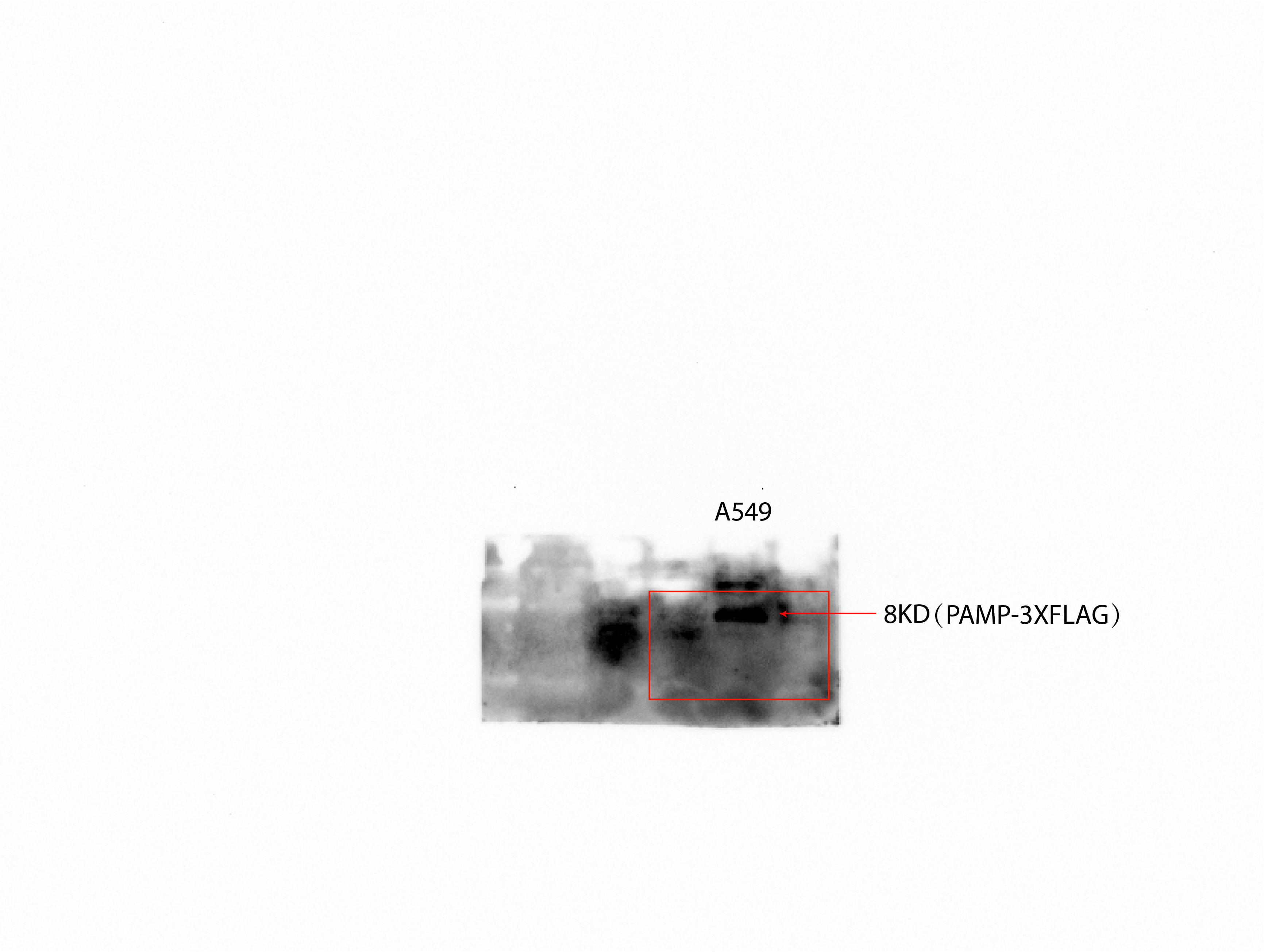

Supplement: Supplementary file 5 — Source data Fig. 2 [file 44321_2026_460_MOESM5_ESM.zip › Source data Figure2/FIG 2C/flag.png]

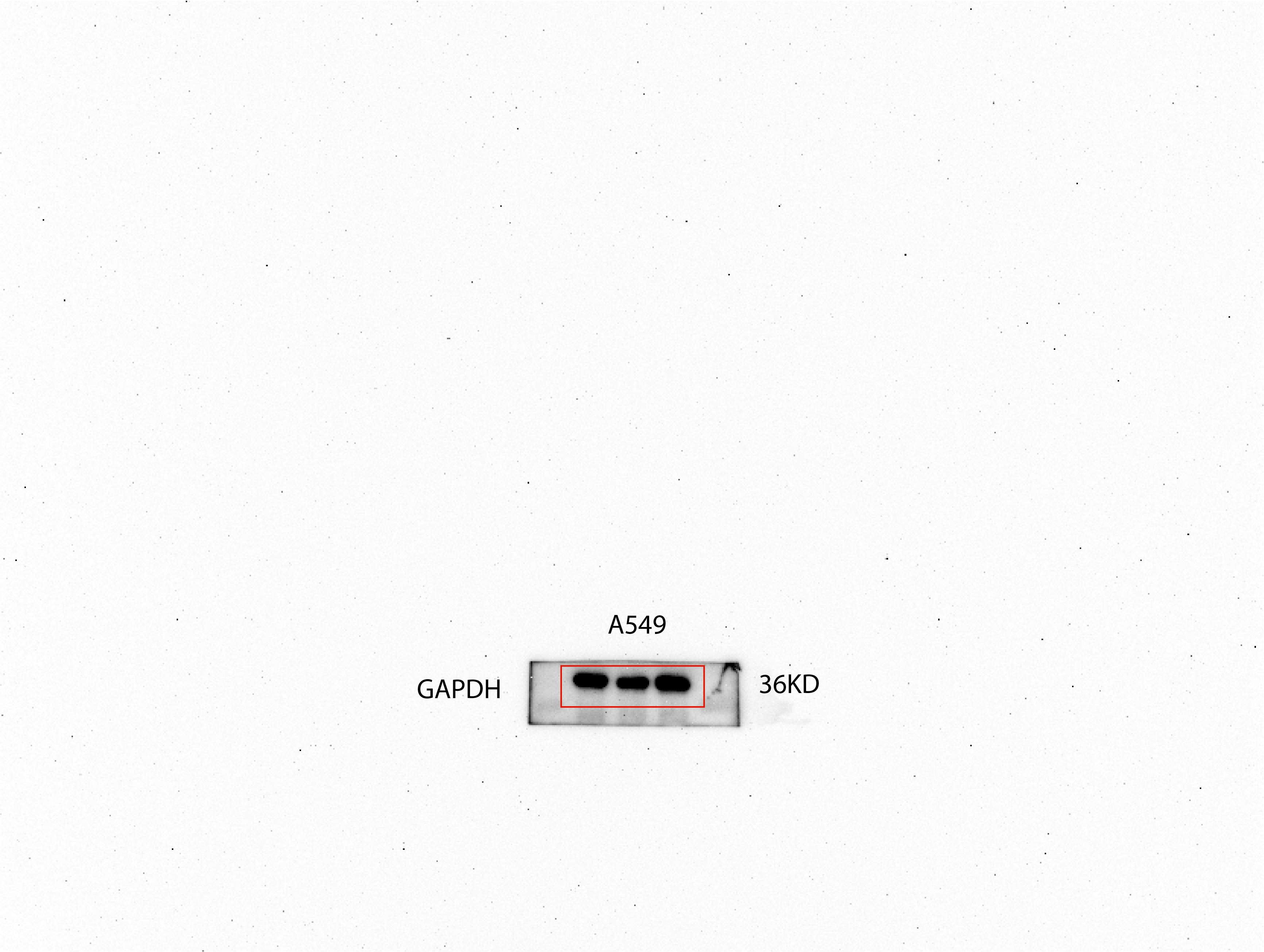

Supplement: Supplementary file 5 — Source data Fig. 2 [file 44321_2026_460_MOESM5_ESM.zip › Source data Figure2/FIG 2C/gapdh.png]

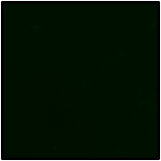

Supplement: Supplementary file 5 — Source data Fig. 2 [file 44321_2026_460_MOESM5_ESM.zip › Source data Figure2/FIG 2D/GFPmut.png]

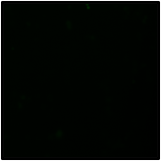

Supplement: Supplementary file 5 — Source data Fig. 2 [file 44321_2026_460_MOESM5_ESM.zip › Source data Figure2/FIG 2D/PAMPmut-GFPmut.png]

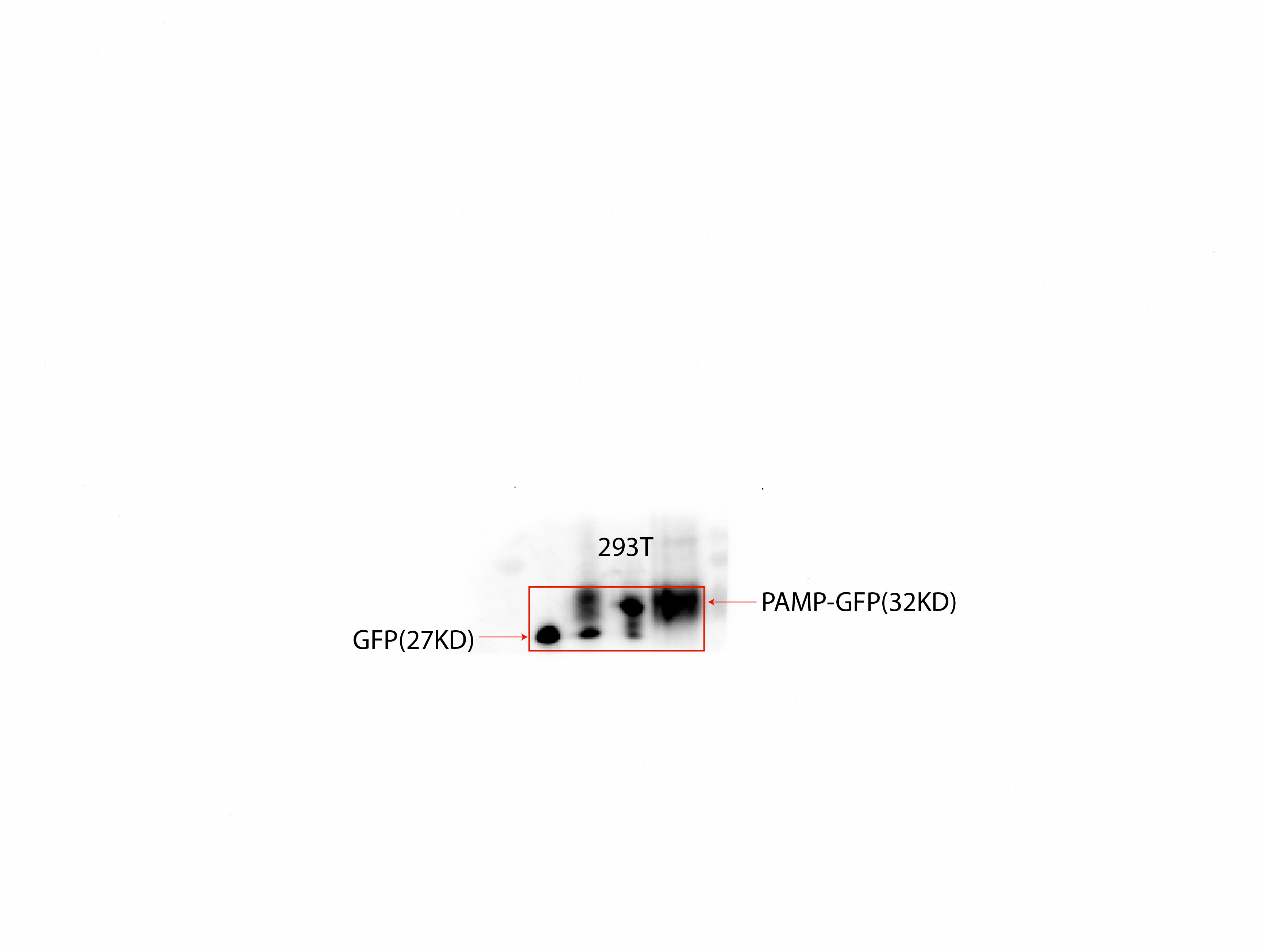

Supplement: Supplementary file 5 — Source data Fig. 2 [file 44321_2026_460_MOESM5_ESM.zip › Source data Figure2/FIG 2E.png]

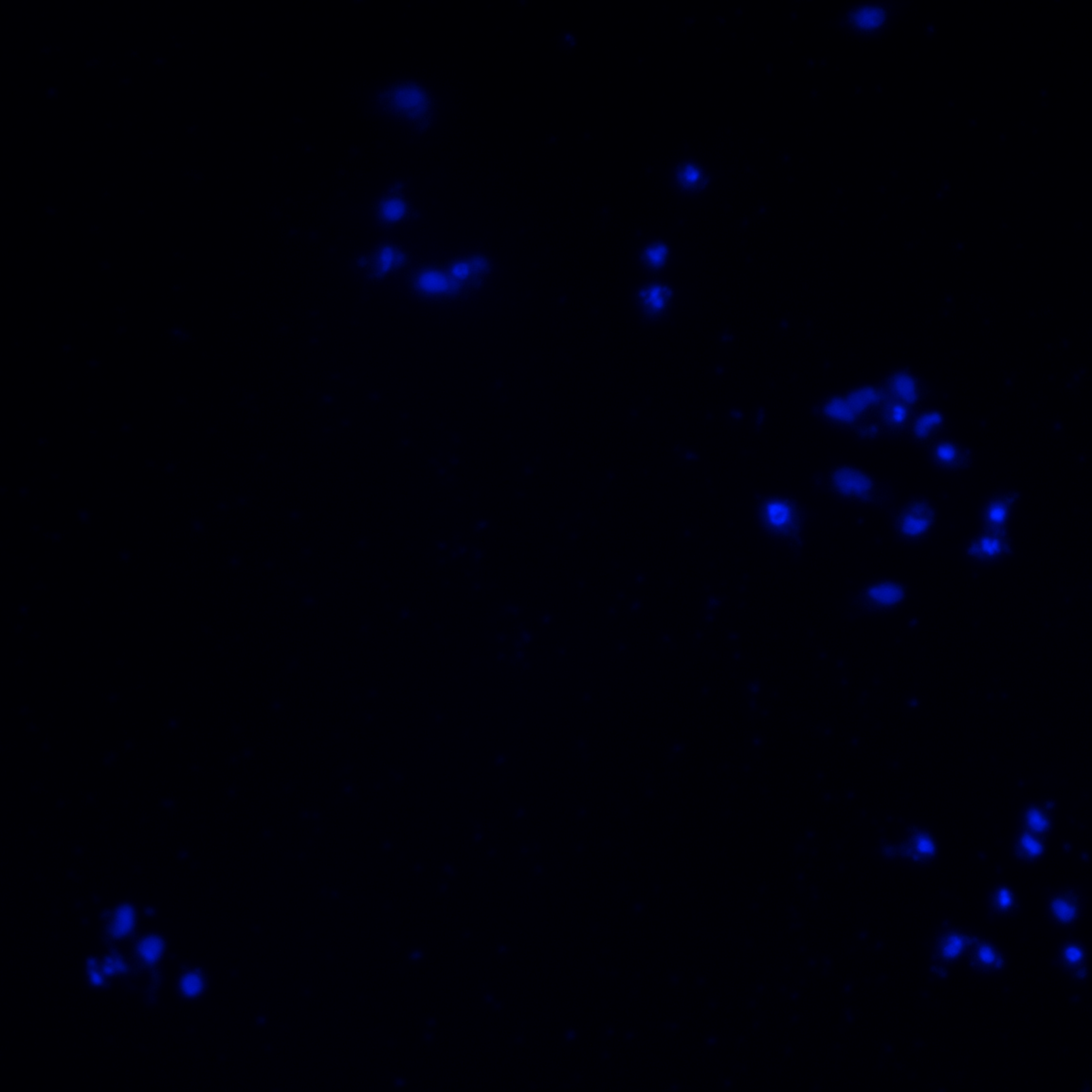

Supplement: Supplementary file 5 — Source data Fig. 2 [file 44321_2026_460_MOESM5_ESM.zip › Source data Figure2/FIG 2G/EV-Dapi.tif]

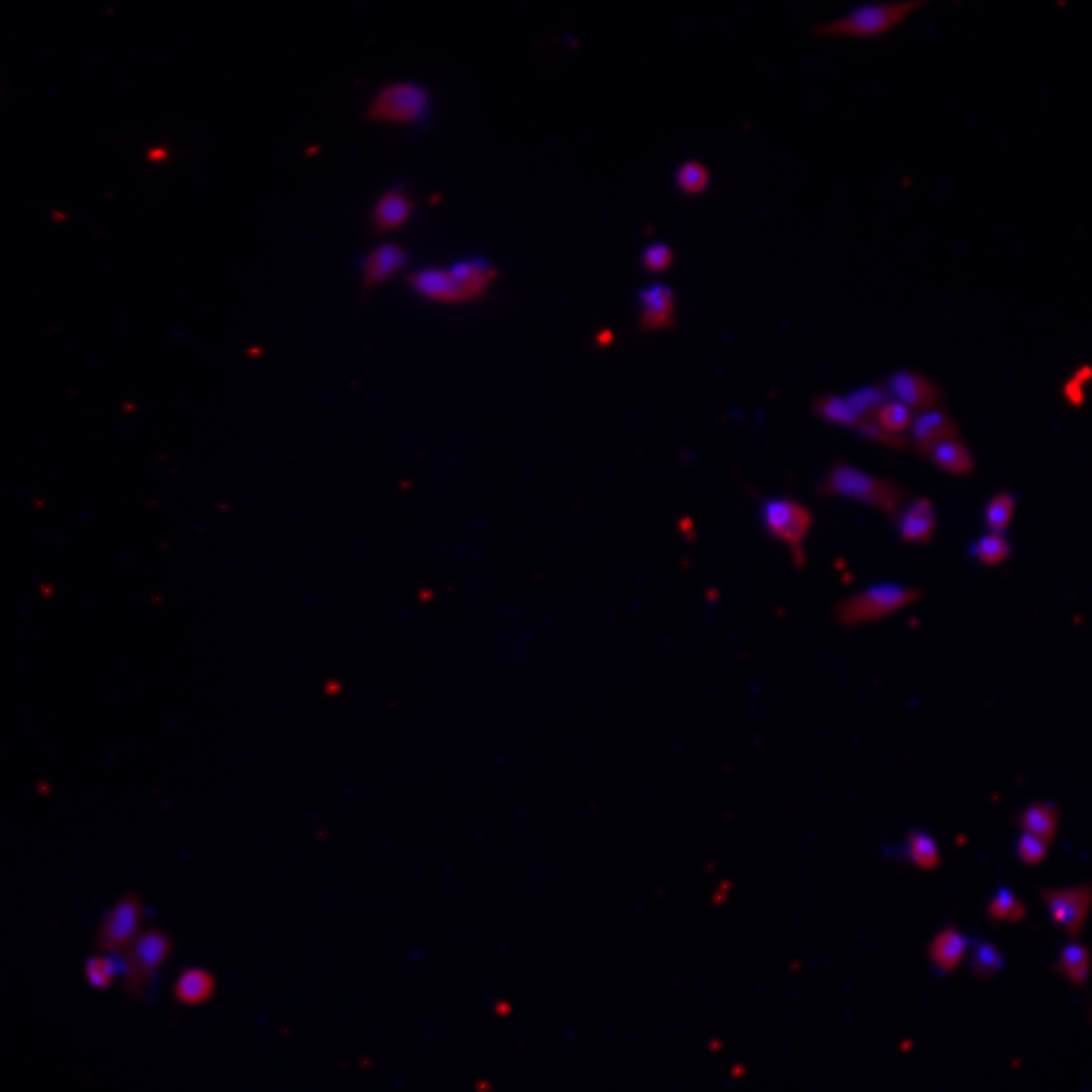

Supplement: Supplementary file 5 — Source data Fig. 2 [file 44321_2026_460_MOESM5_ESM.zip › Source data Figure2/FIG 2G/EV-Merged.tif]

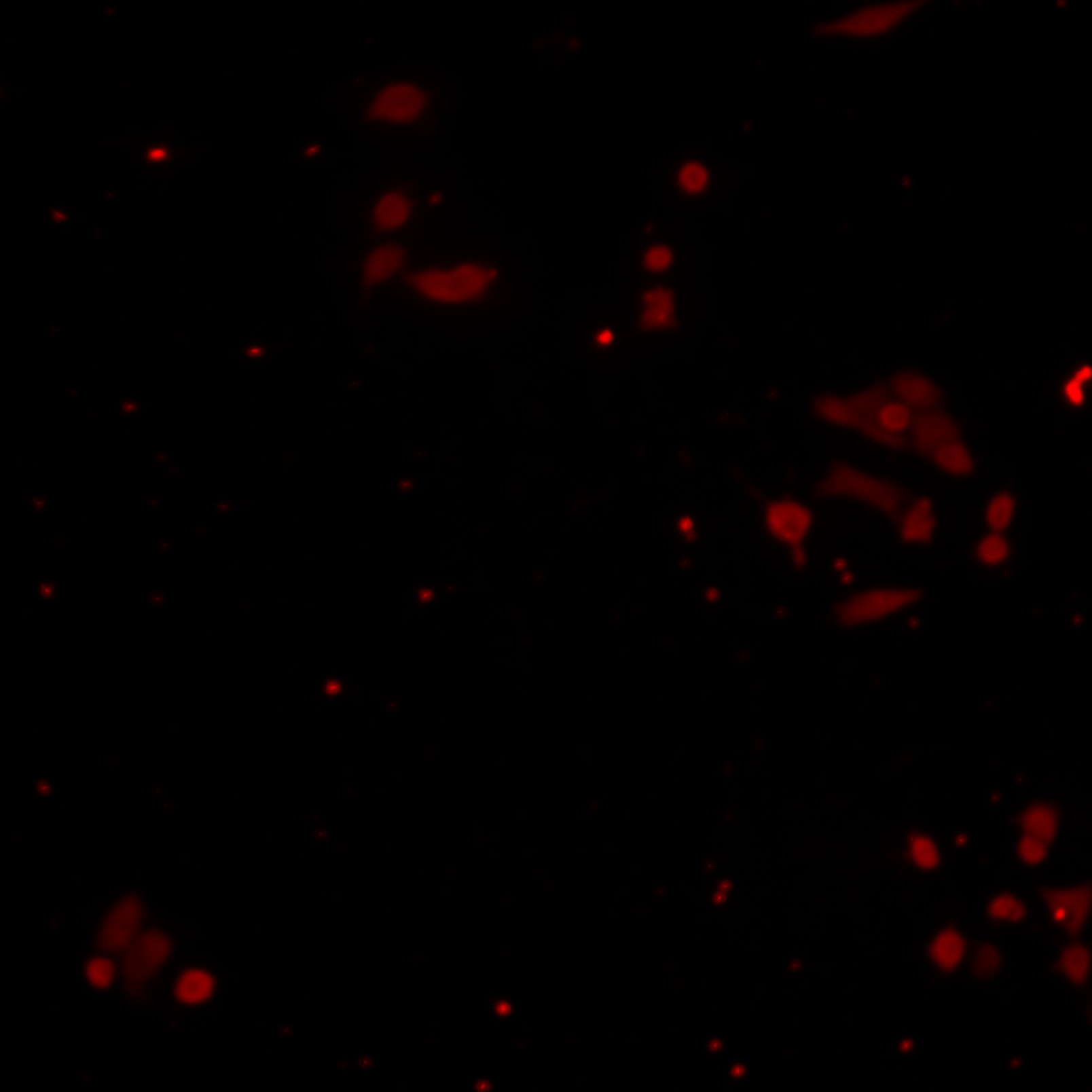

Supplement: Supplementary file 5 — Source data Fig. 2 [file 44321_2026_460_MOESM5_ESM.zip › Source data Figure2/FIG 2G/EV-PAMP.tif]

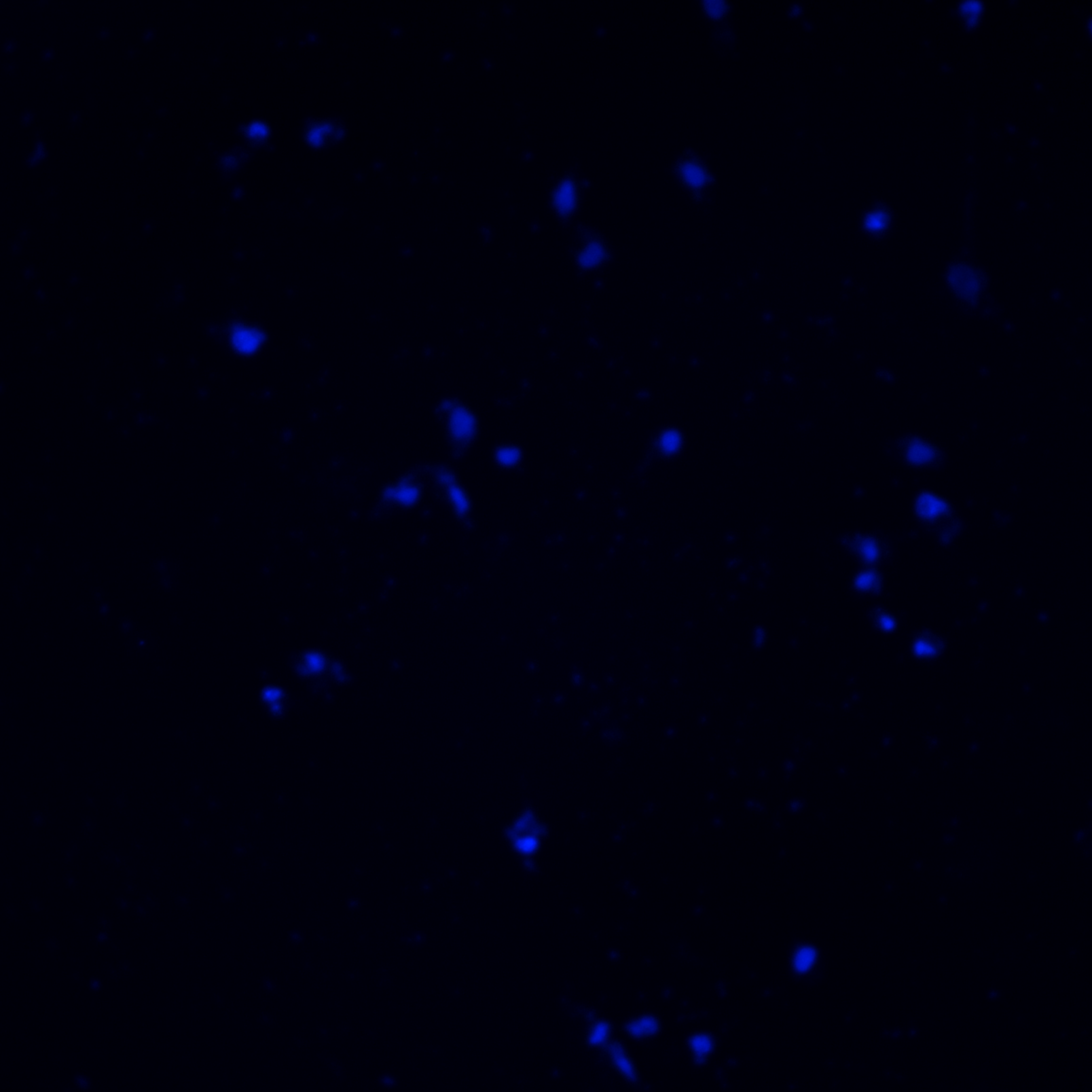

Supplement: Supplementary file 5 — Source data Fig. 2 [file 44321_2026_460_MOESM5_ESM.zip › Source data Figure2/FIG 2G/PAMP-Dapi.tif]

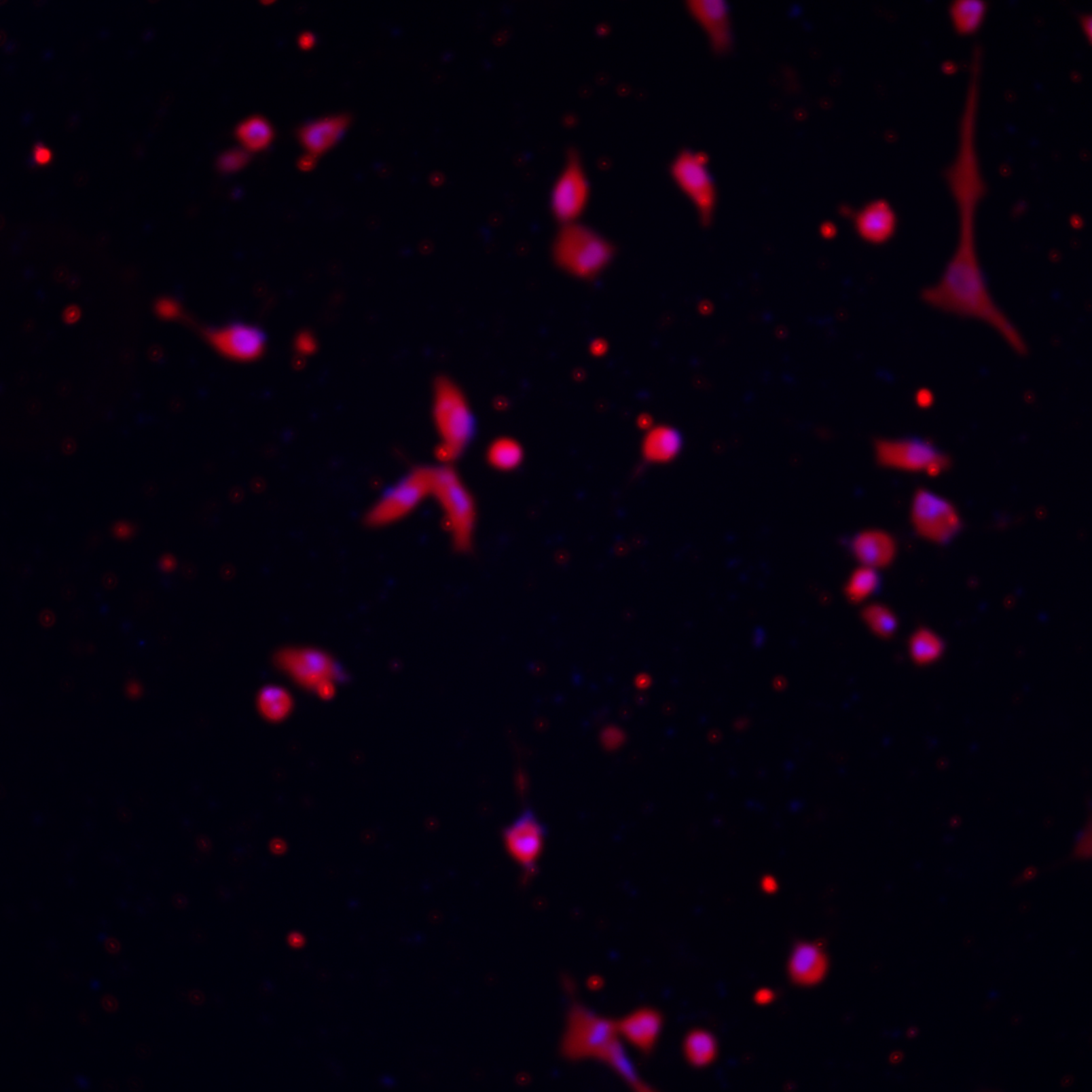

Supplement: Supplementary file 5 — Source data Fig. 2 [file 44321_2026_460_MOESM5_ESM.zip › Source data Figure2/FIG 2G/PAMP-Merged.tif]

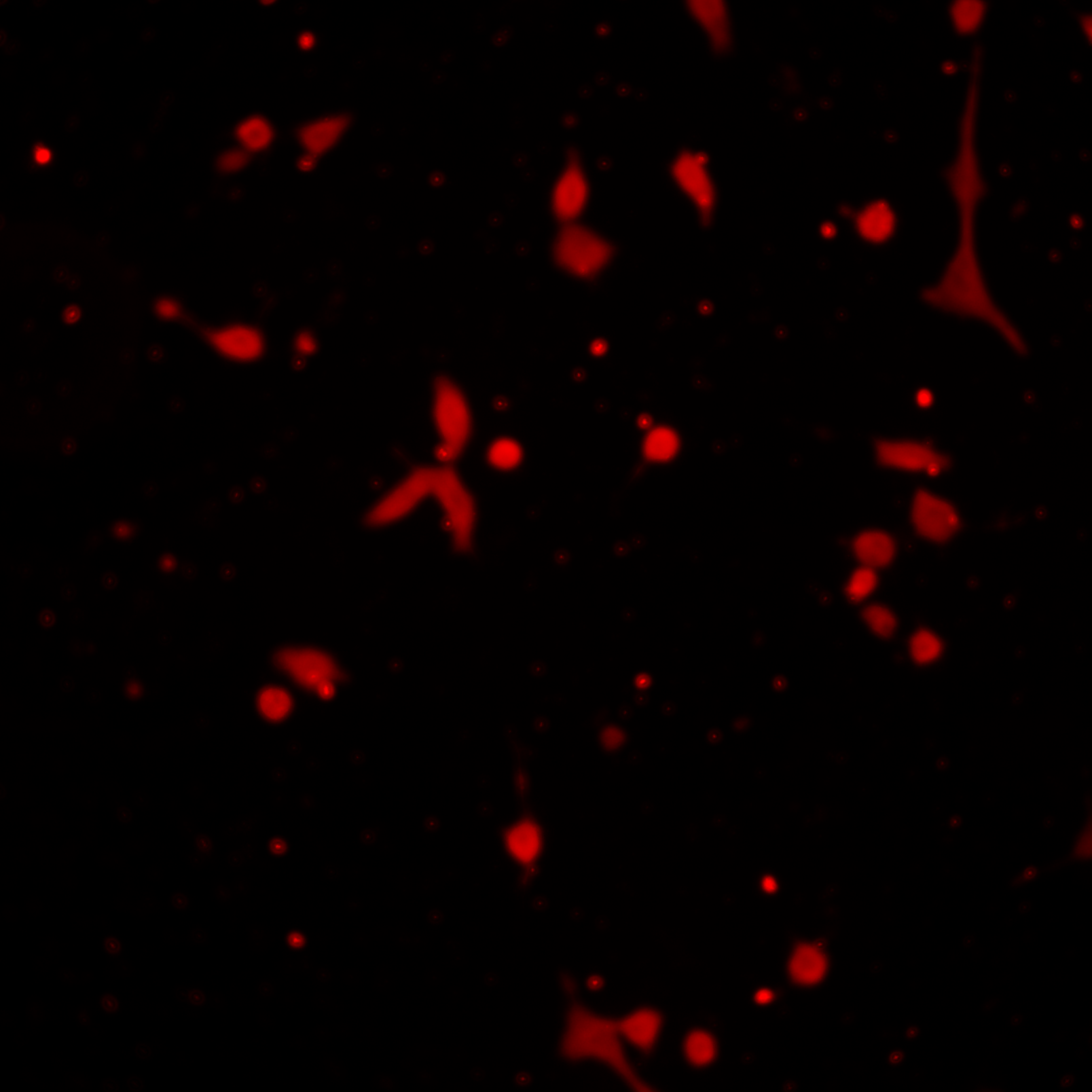

Supplement: Supplementary file 5 — Source data Fig. 2 [file 44321_2026_460_MOESM5_ESM.zip › Source data Figure2/FIG 2G/PAMP-PAMP.tif]

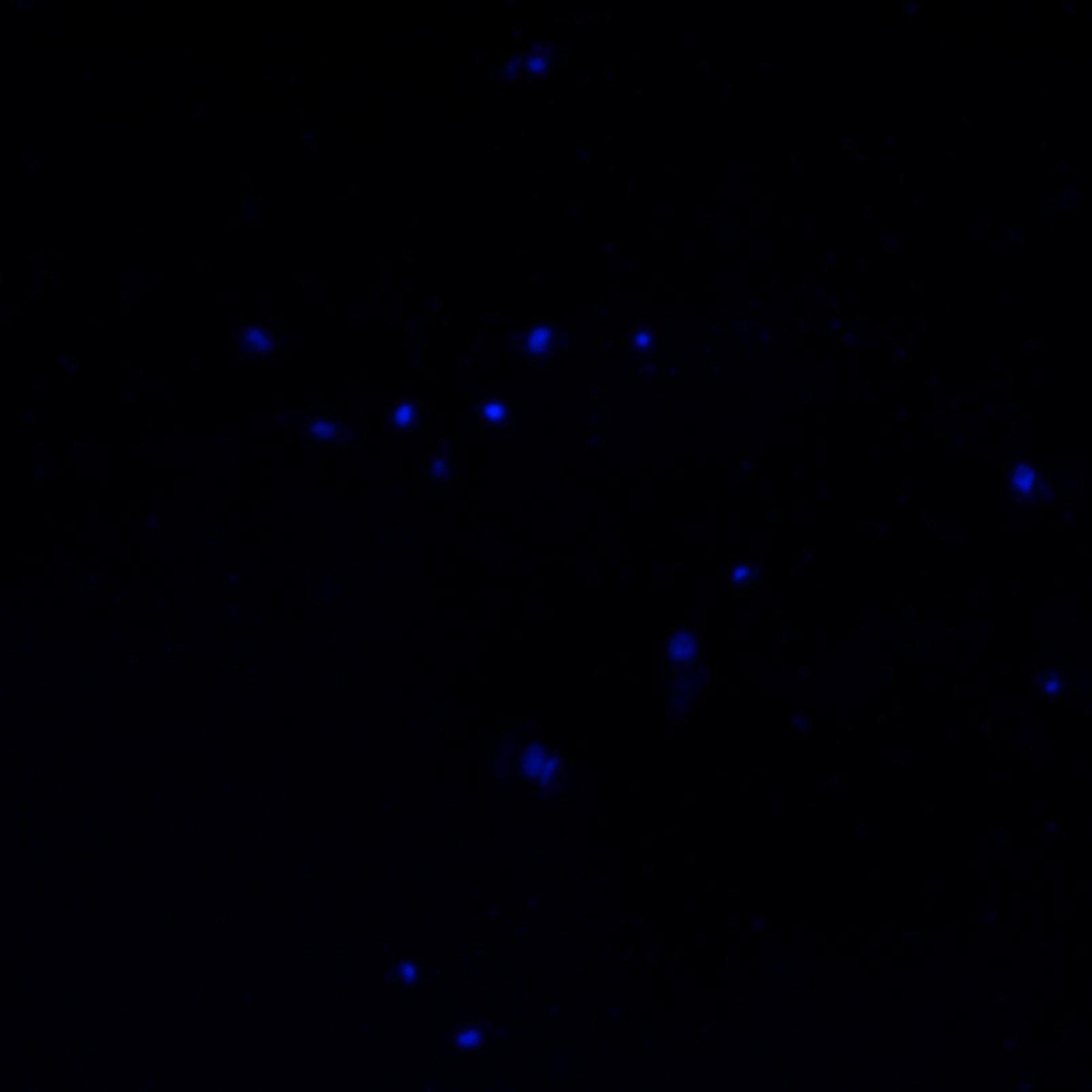

Supplement: Supplementary file 5 — Source data Fig. 2 [file 44321_2026_460_MOESM5_ESM.zip › Source data Figure2/FIG 2G/PAMPmut-Dapi.tif]

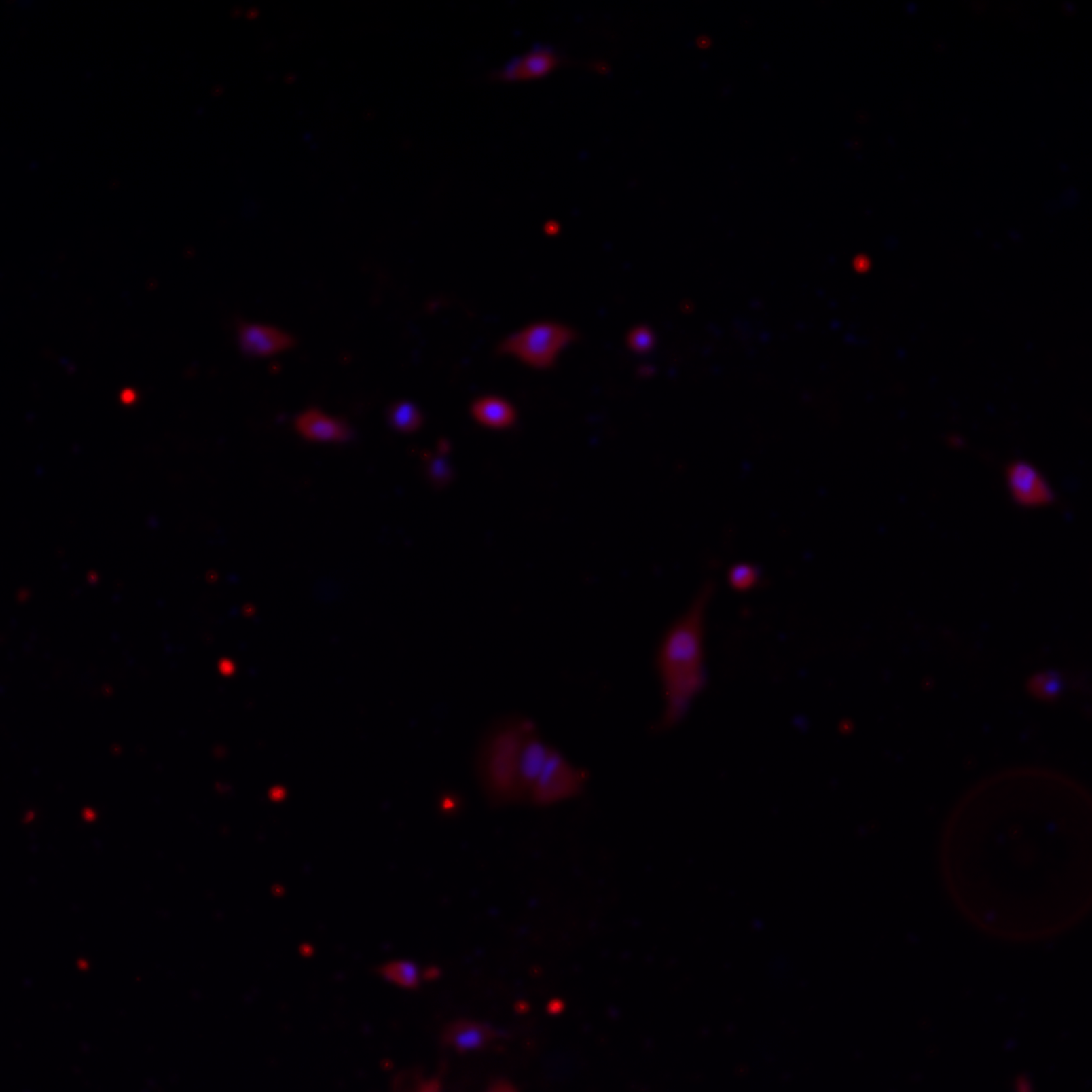

Supplement: Supplementary file 5 — Source data Fig. 2 [file 44321_2026_460_MOESM5_ESM.zip › Source data Figure2/FIG 2G/PAMPmut-Merged.tif]

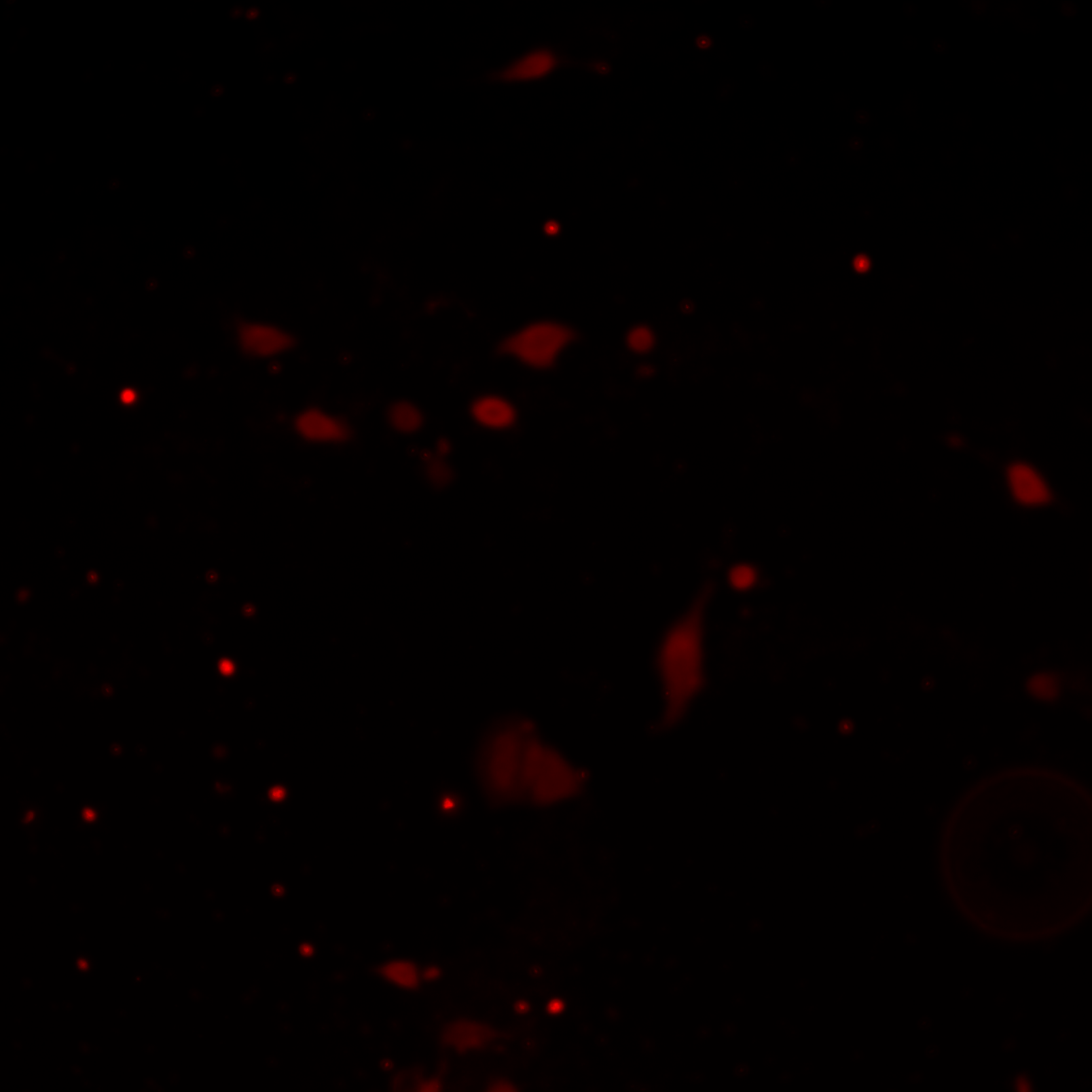

Supplement: Supplementary file 5 — Source data Fig. 2 [file 44321_2026_460_MOESM5_ESM.zip › Source data Figure2/FIG 2G/PAMPmut-PAMP.tif]

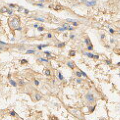

Supplement: Supplementary file 5 — Source data Fig. 2 [file 44321_2026_460_MOESM5_ESM.zip › Source data Figure2/FIG 2H/normal1.png]

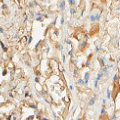

Supplement: Supplementary file 5 — Source data Fig. 2 [file 44321_2026_460_MOESM5_ESM.zip › Source data Figure2/FIG 2H/normal2.png]

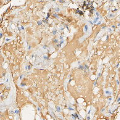

Supplement: Supplementary file 5 — Source data Fig. 2 [file 44321_2026_460_MOESM5_ESM.zip › Source data Figure2/FIG 2H/normal3.png]

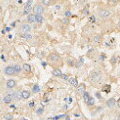

Supplement: Supplementary file 5 — Source data Fig. 2 [file 44321_2026_460_MOESM5_ESM.zip › Source data Figure2/FIG 2H/tumor1.png]

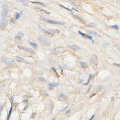

Supplement: Supplementary file 5 — Source data Fig. 2 [file 44321_2026_460_MOESM5_ESM.zip › Source data Figure2/FIG 2H/tumor2.png]

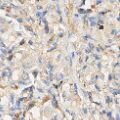

Supplement: Supplementary file 5 — Source data Fig. 2 [file 44321_2026_460_MOESM5_ESM.zip › Source data Figure2/FIG 2H/tumor3.png]

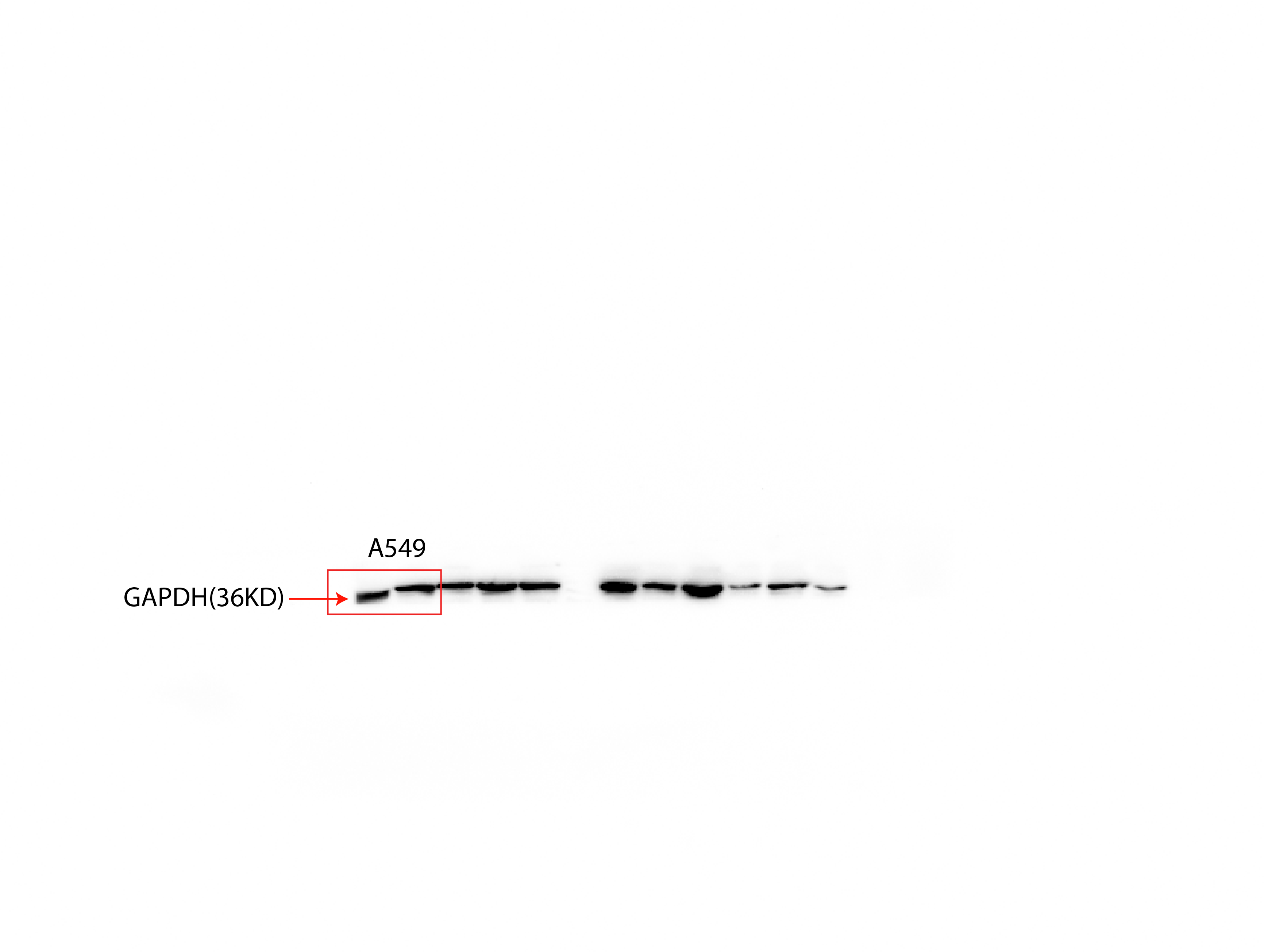

Supplement: Supplementary file 6 — Source data Fig. 3 [file 44321_2026_460_MOESM6_ESM.zip › Source data Figure3/FIG 3B/GAPDH-1.png]

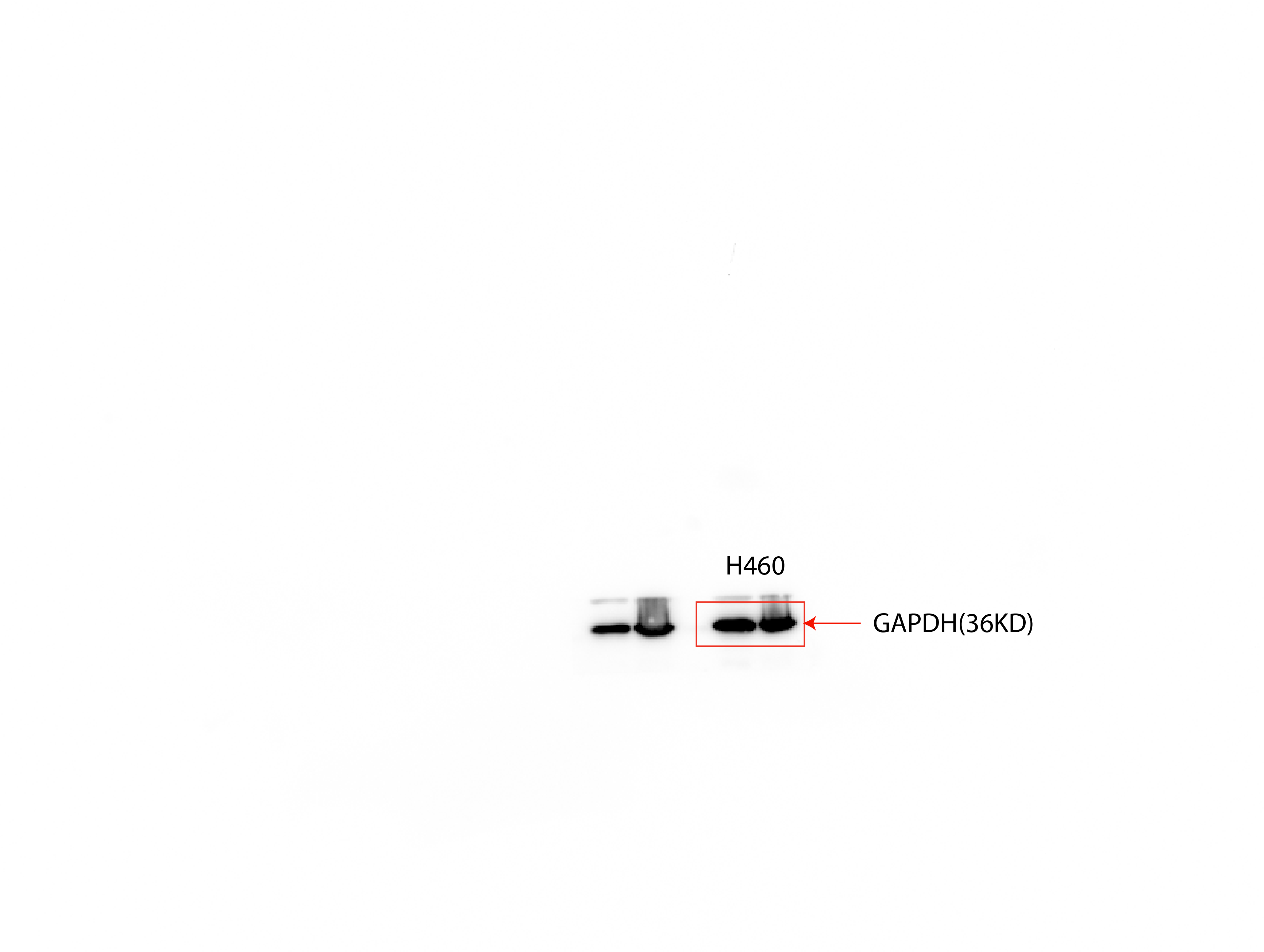

Supplement: Supplementary file 6 — Source data Fig. 3 [file 44321_2026_460_MOESM6_ESM.zip › Source data Figure3/FIG 3B/GAPDH-2.png]

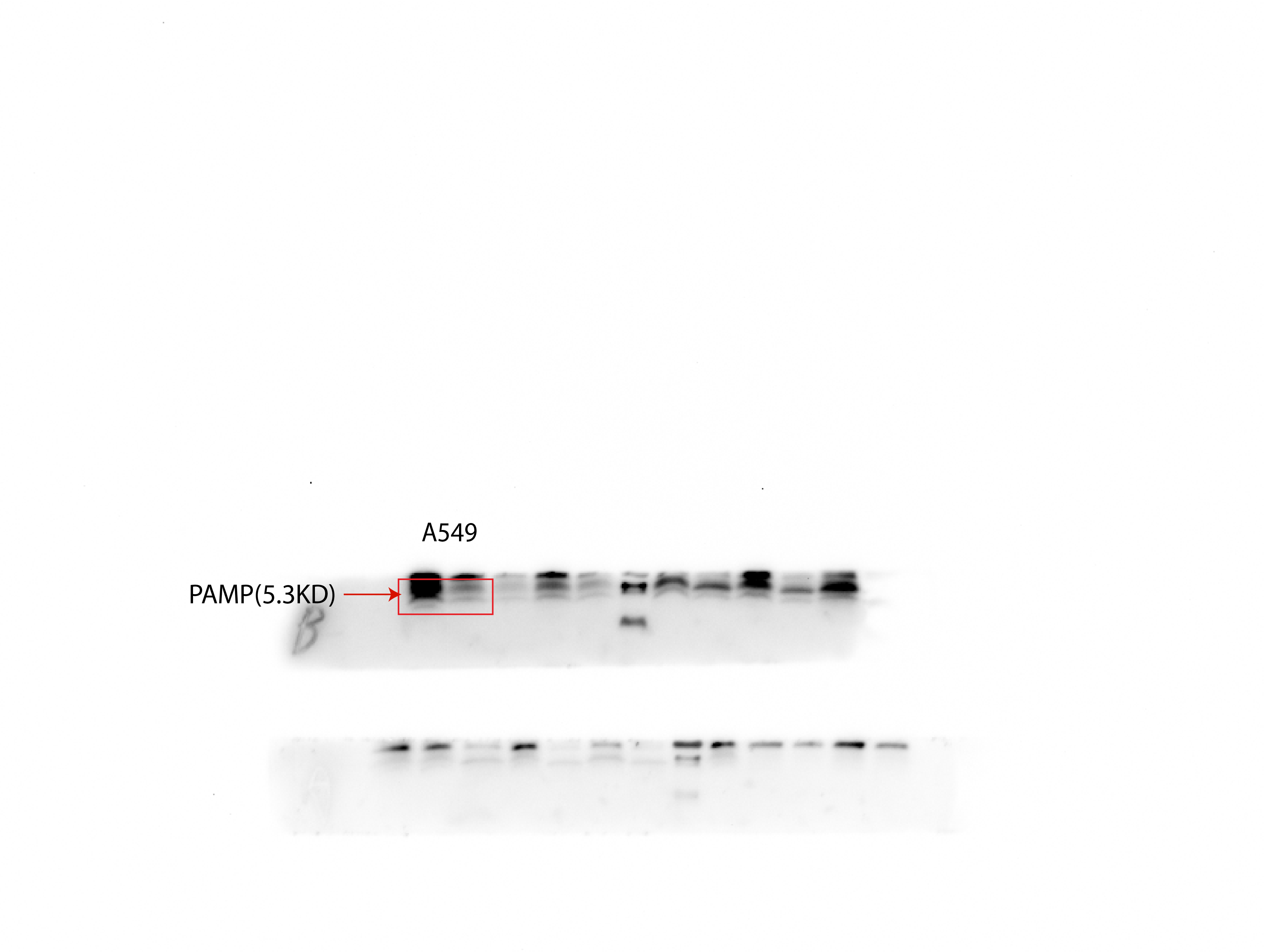

Supplement: Supplementary file 6 — Source data Fig. 3 [file 44321_2026_460_MOESM6_ESM.zip › Source data Figure3/FIG 3B/PAMP-1.png]

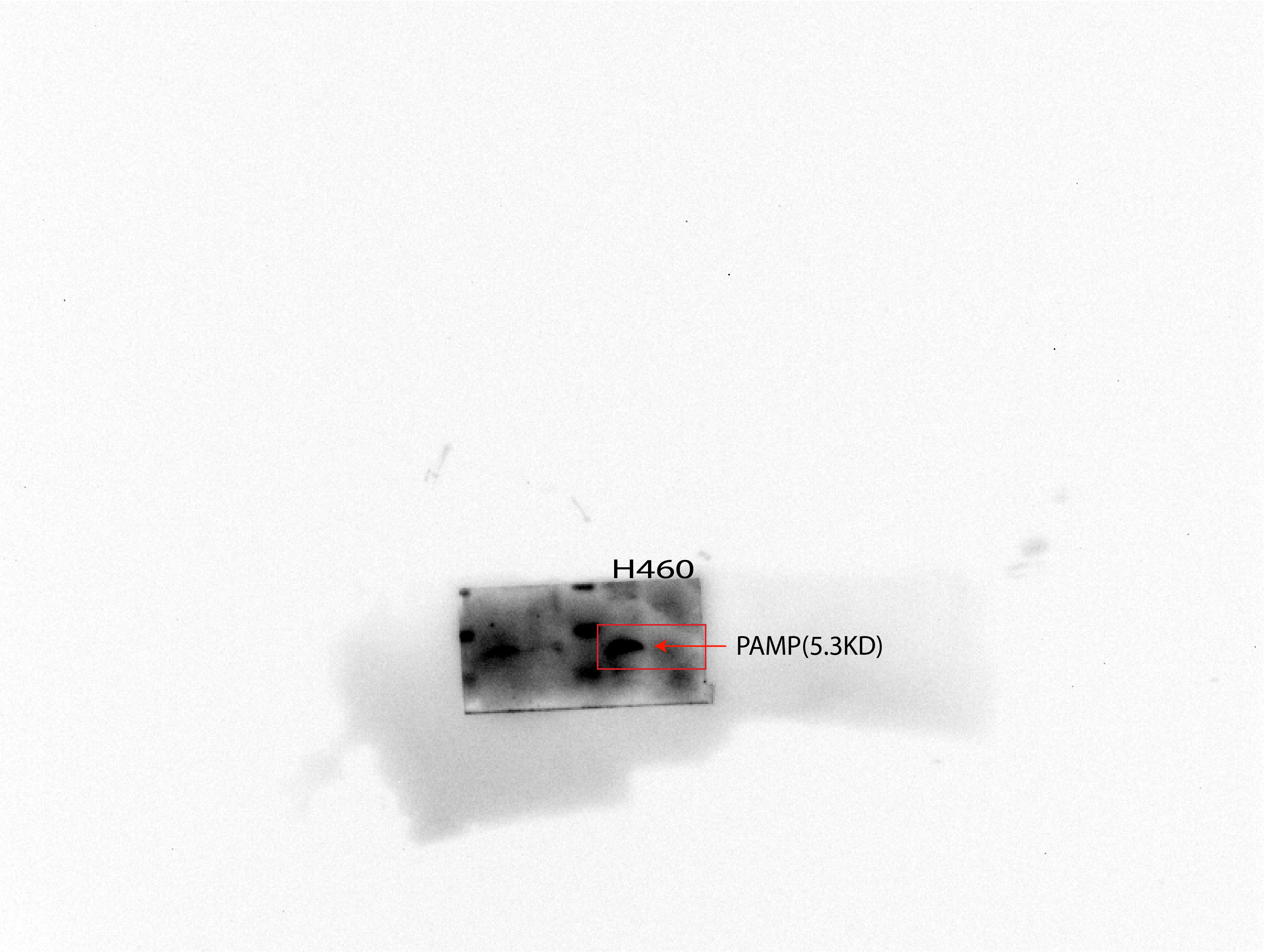

Supplement: Supplementary file 6 — Source data Fig. 3 [file 44321_2026_460_MOESM6_ESM.zip › Source data Figure3/FIG 3B/PAMP-2.png]

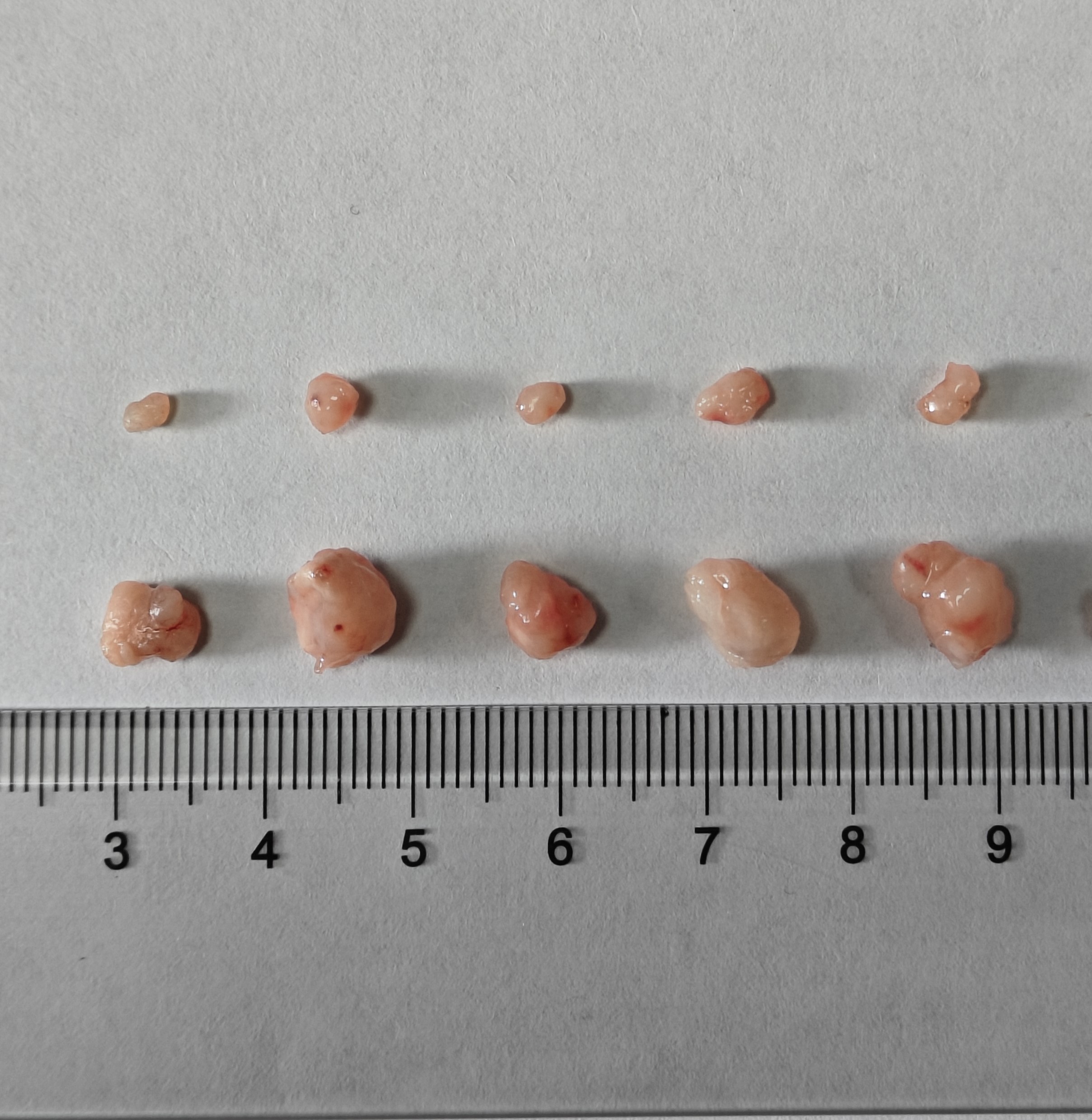

Supplement: Supplementary file 6 — Source data Fig. 3 [file 44321_2026_460_MOESM6_ESM.zip › Source data Figure3/FIG 3E.jpg]

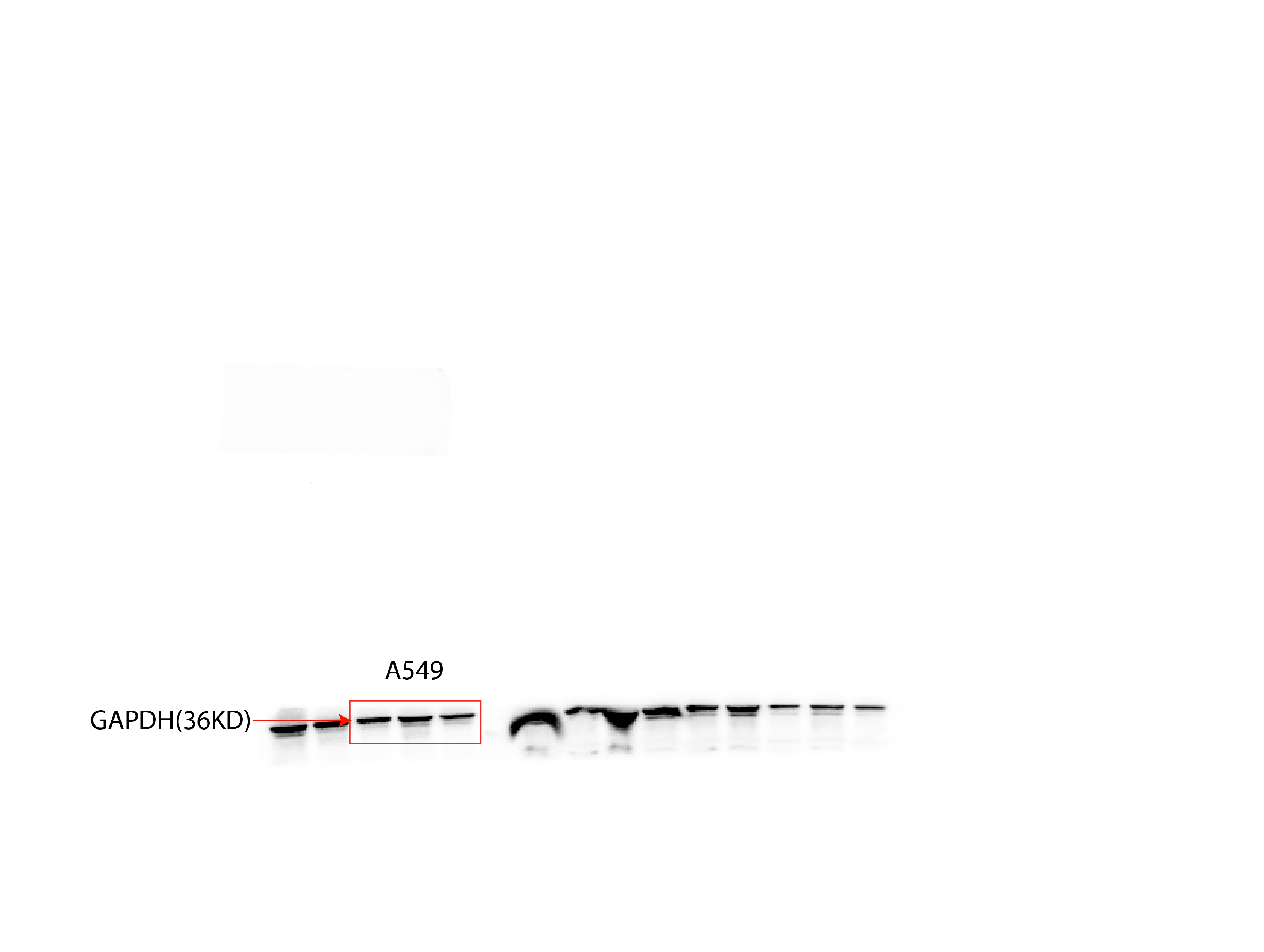

Supplement: Supplementary file 6 — Source data Fig. 3 [file 44321_2026_460_MOESM6_ESM.zip › Source data Figure3/FIG 3G/GAPDH-1.png]

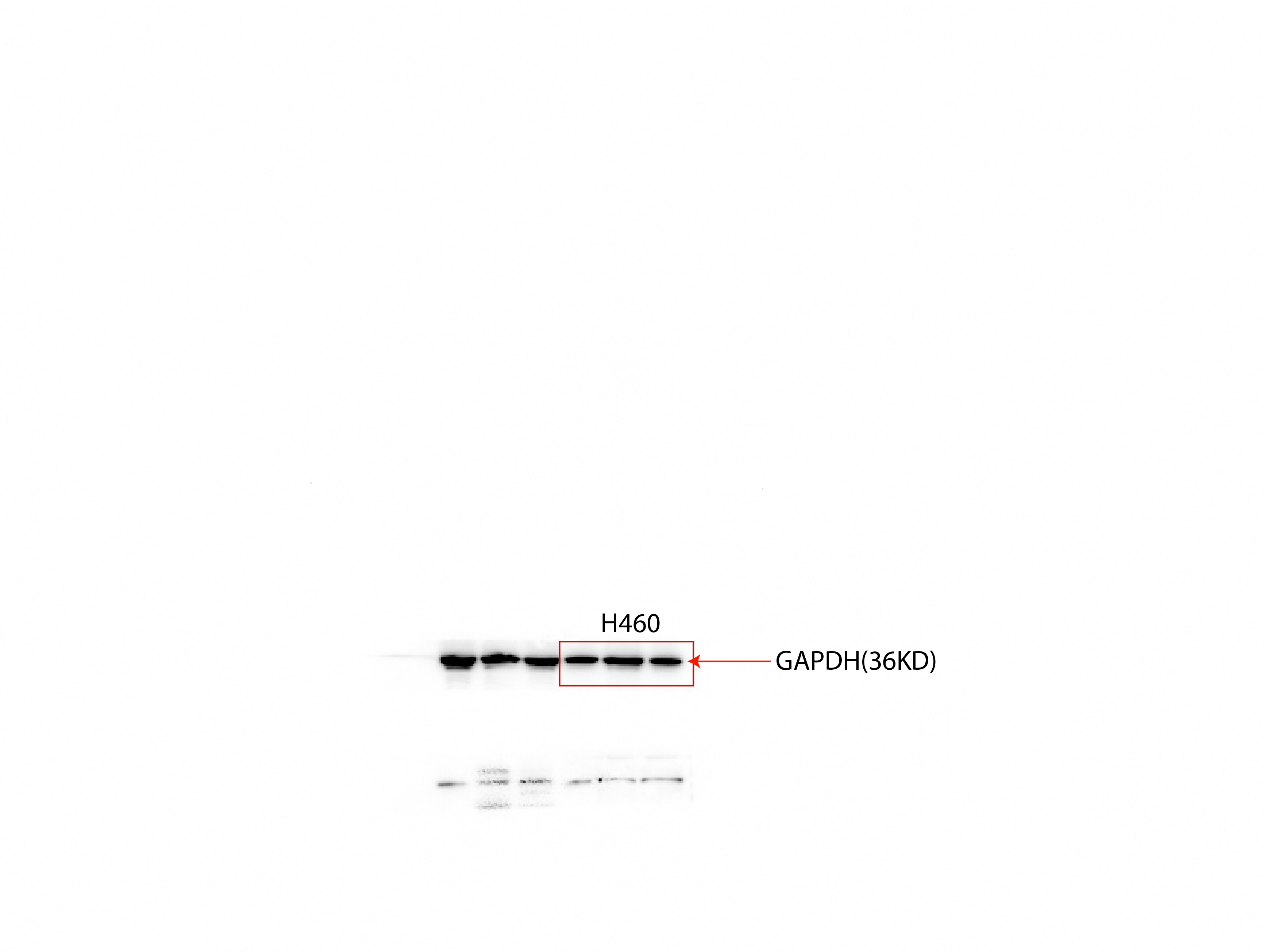

Supplement: Supplementary file 6 — Source data Fig. 3 [file 44321_2026_460_MOESM6_ESM.zip › Source data Figure3/FIG 3G/GAPDH-2.png]

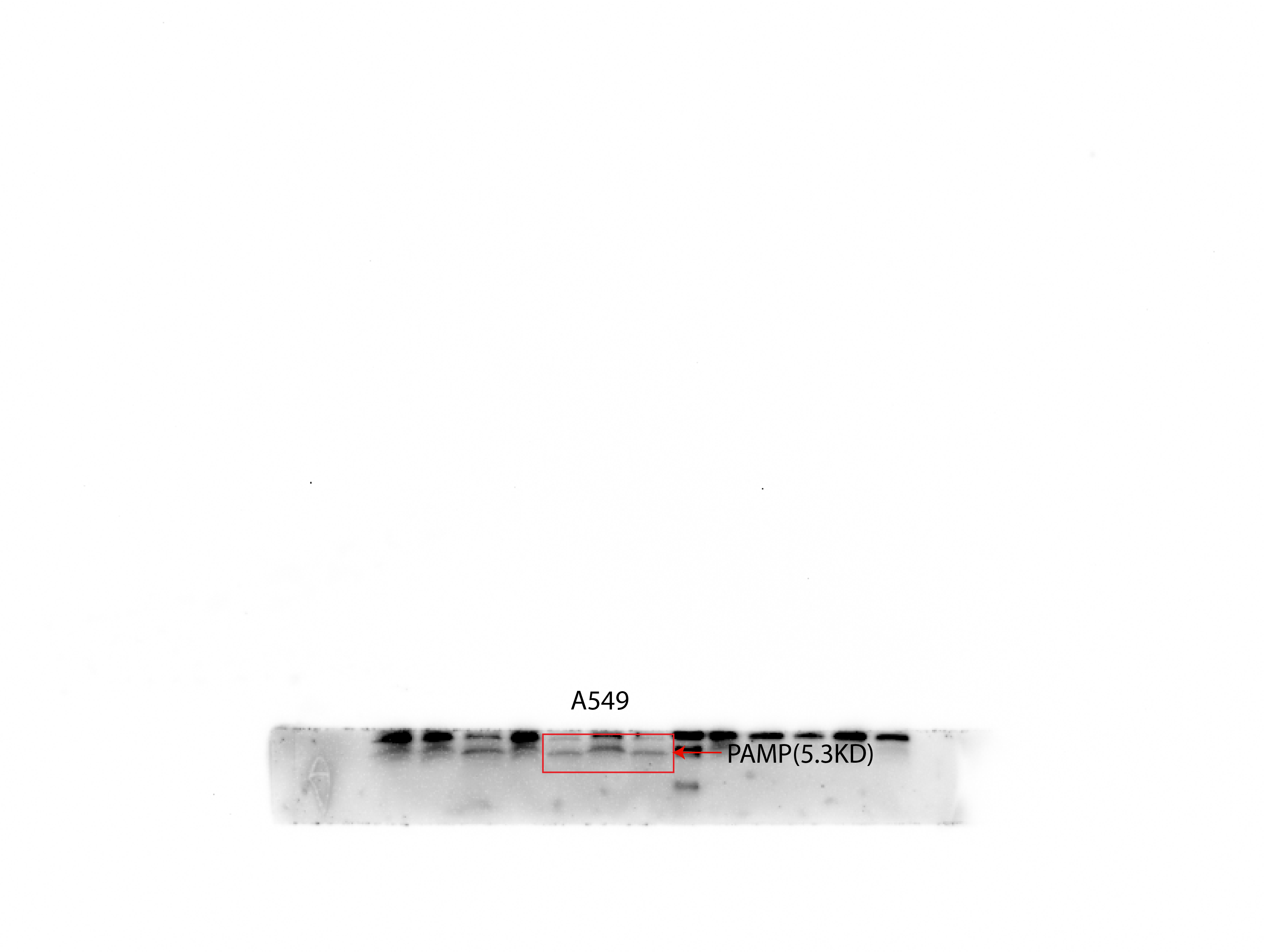

Supplement: Supplementary file 6 — Source data Fig. 3 [file 44321_2026_460_MOESM6_ESM.zip › Source data Figure3/FIG 3G/PAMP-1.png]

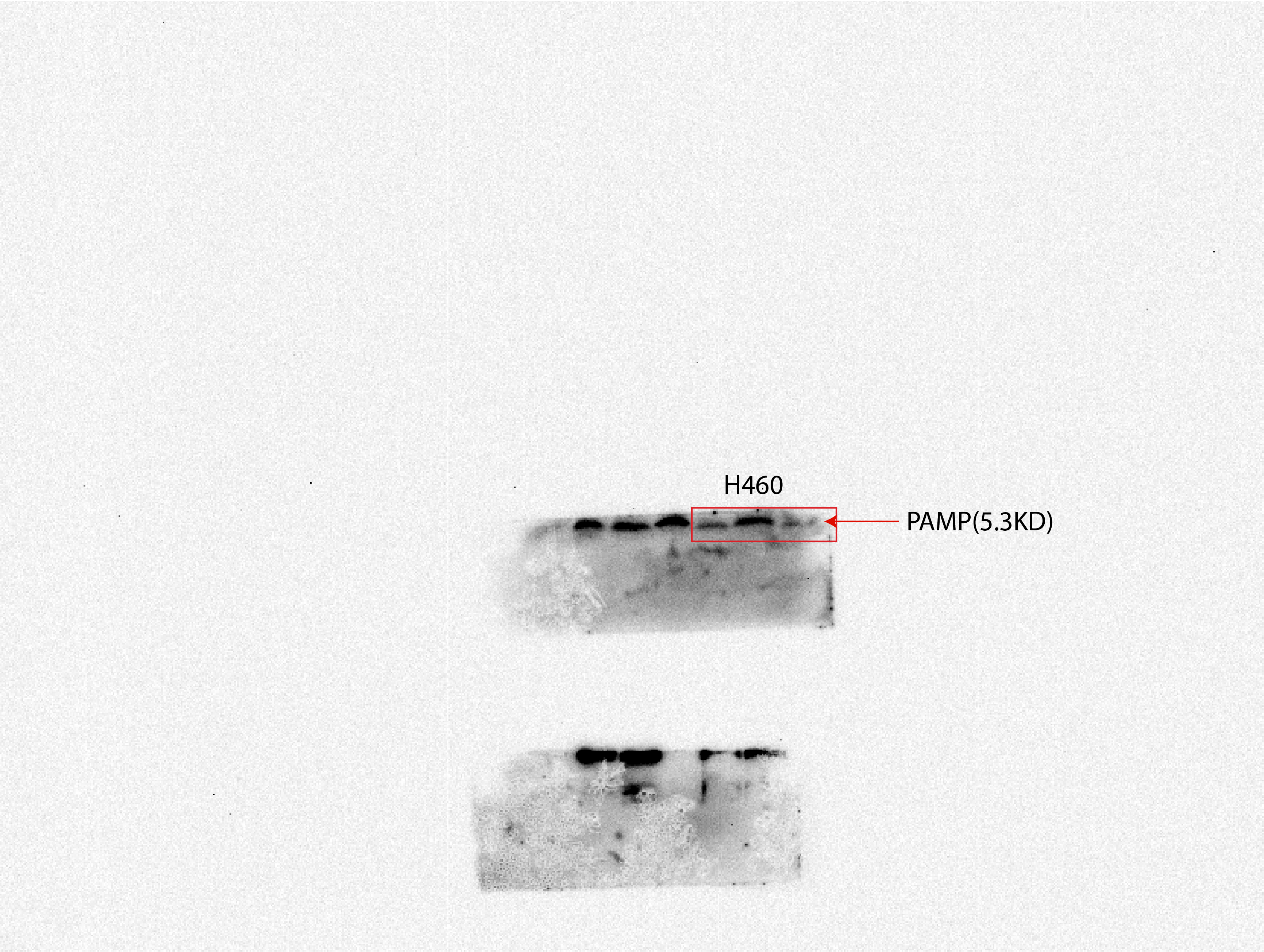

Supplement: Supplementary file 6 — Source data Fig. 3 [file 44321_2026_460_MOESM6_ESM.zip › Source data Figure3/FIG 3G/PAMP-2.png]

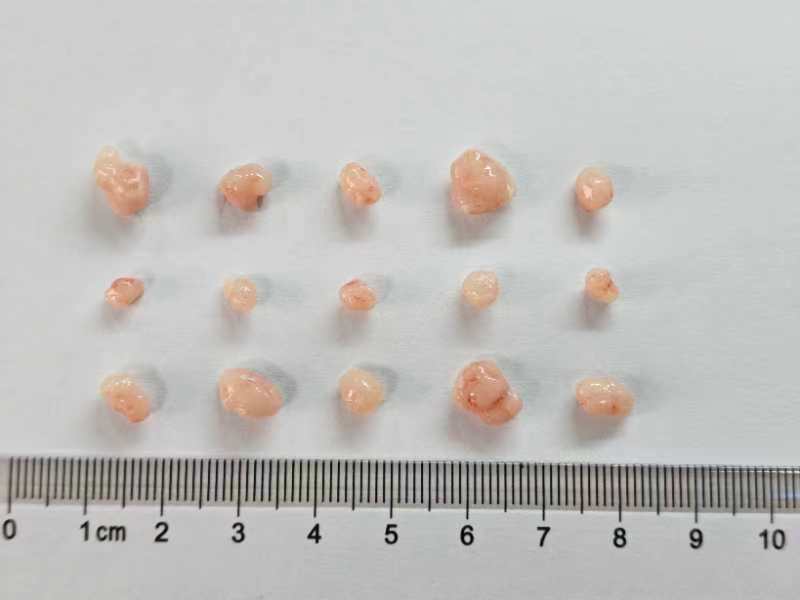

Supplement: Supplementary file 6 — Source data Fig. 3 [file 44321_2026_460_MOESM6_ESM.zip › Source data Figure3/FIG 3J.jpg]

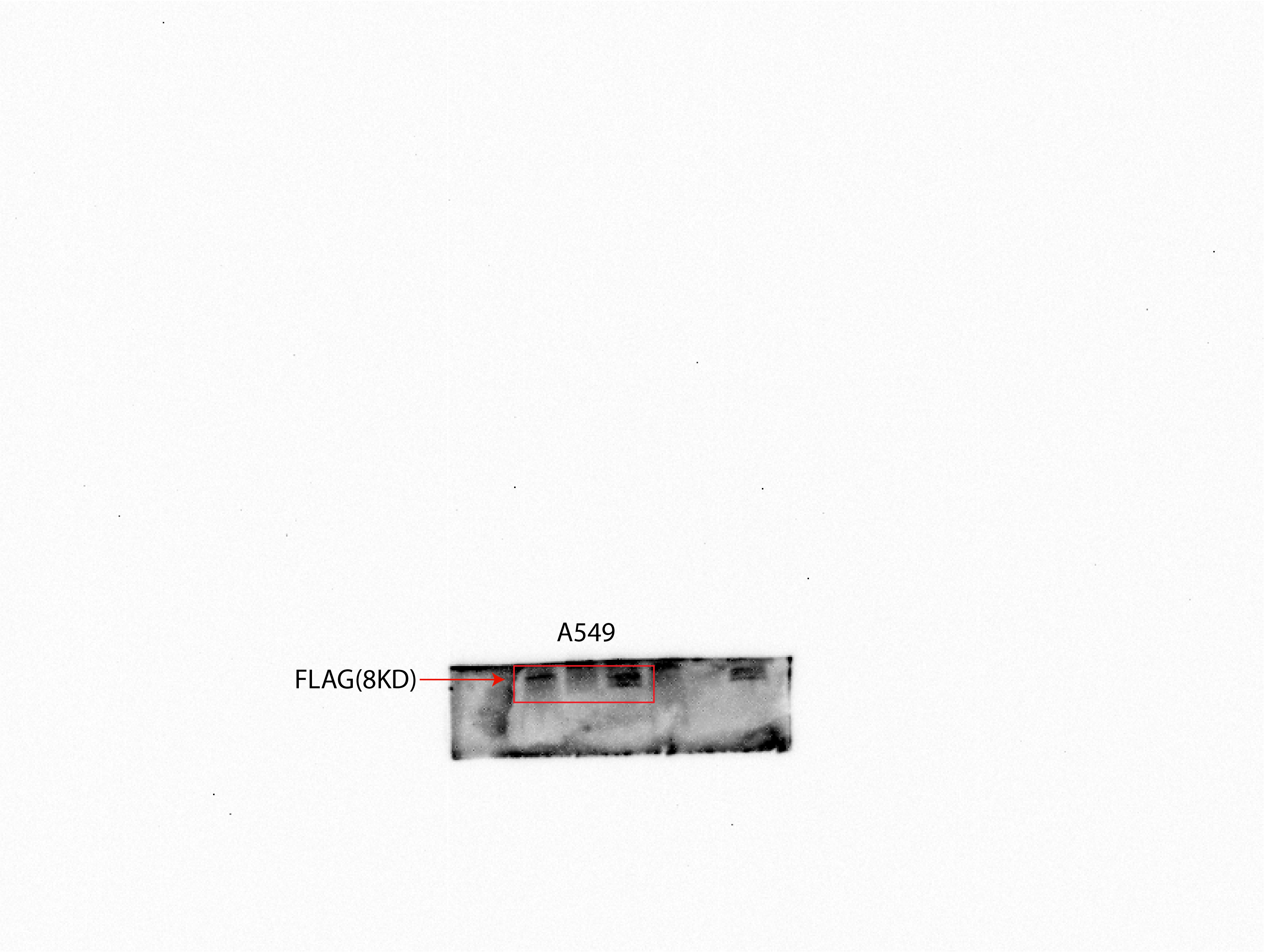

Supplement: Supplementary file 7 — Source data Fig. 4 [file 44321_2026_460_MOESM7_ESM.zip › Source data Figure4/FIG 4D/flag.png]

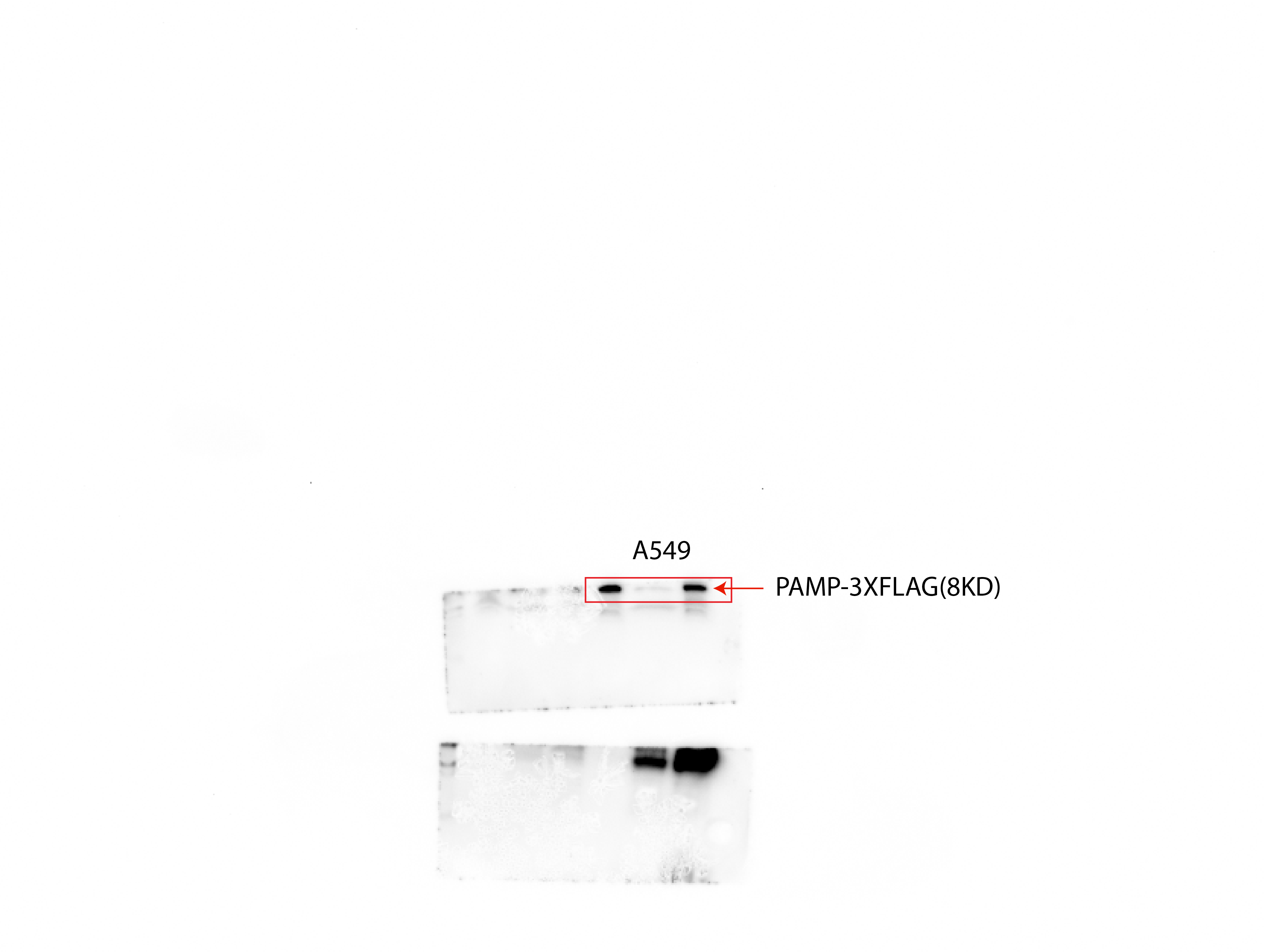

Supplement: Supplementary file 7 — Source data Fig. 4 [file 44321_2026_460_MOESM7_ESM.zip › Source data Figure4/FIG 4D/left-PAMP.png]

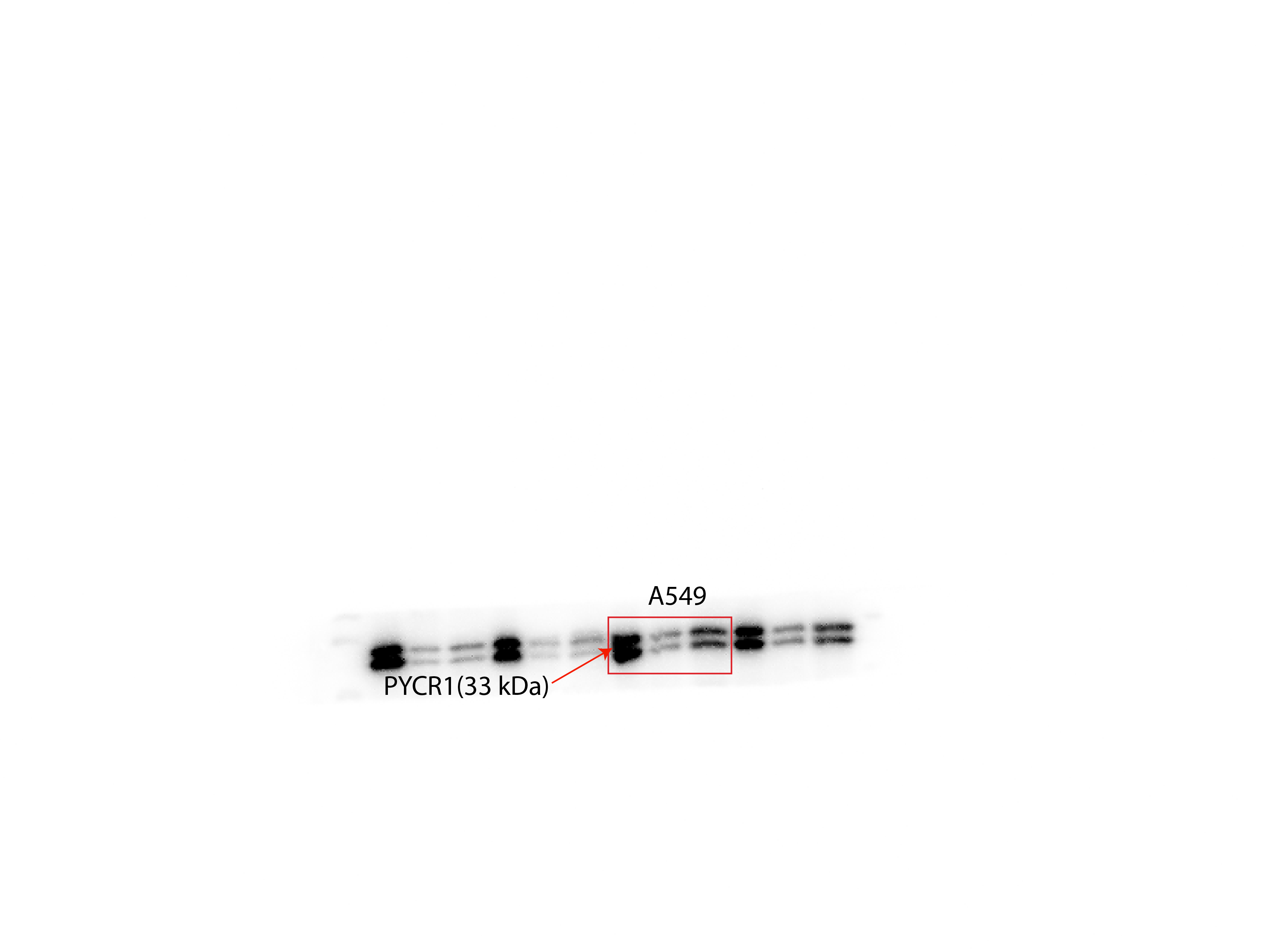

Supplement: Supplementary file 7 — Source data Fig. 4 [file 44321_2026_460_MOESM7_ESM.zip › Source data Figure4/FIG 4D/left-pycr1.png]

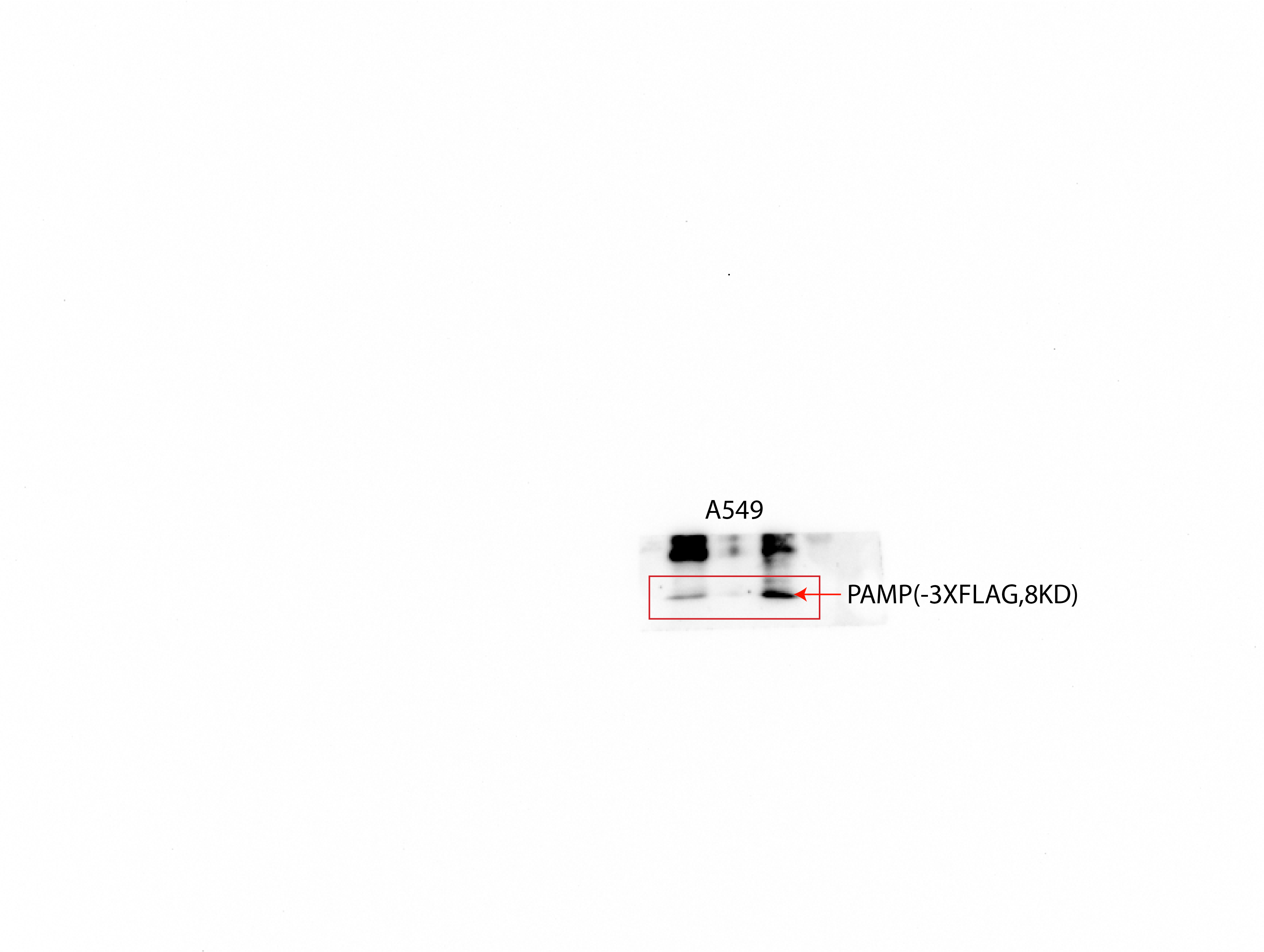

Supplement: Supplementary file 7 — Source data Fig. 4 [file 44321_2026_460_MOESM7_ESM.zip › Source data Figure4/FIG 4D/right-PAMP1.png]

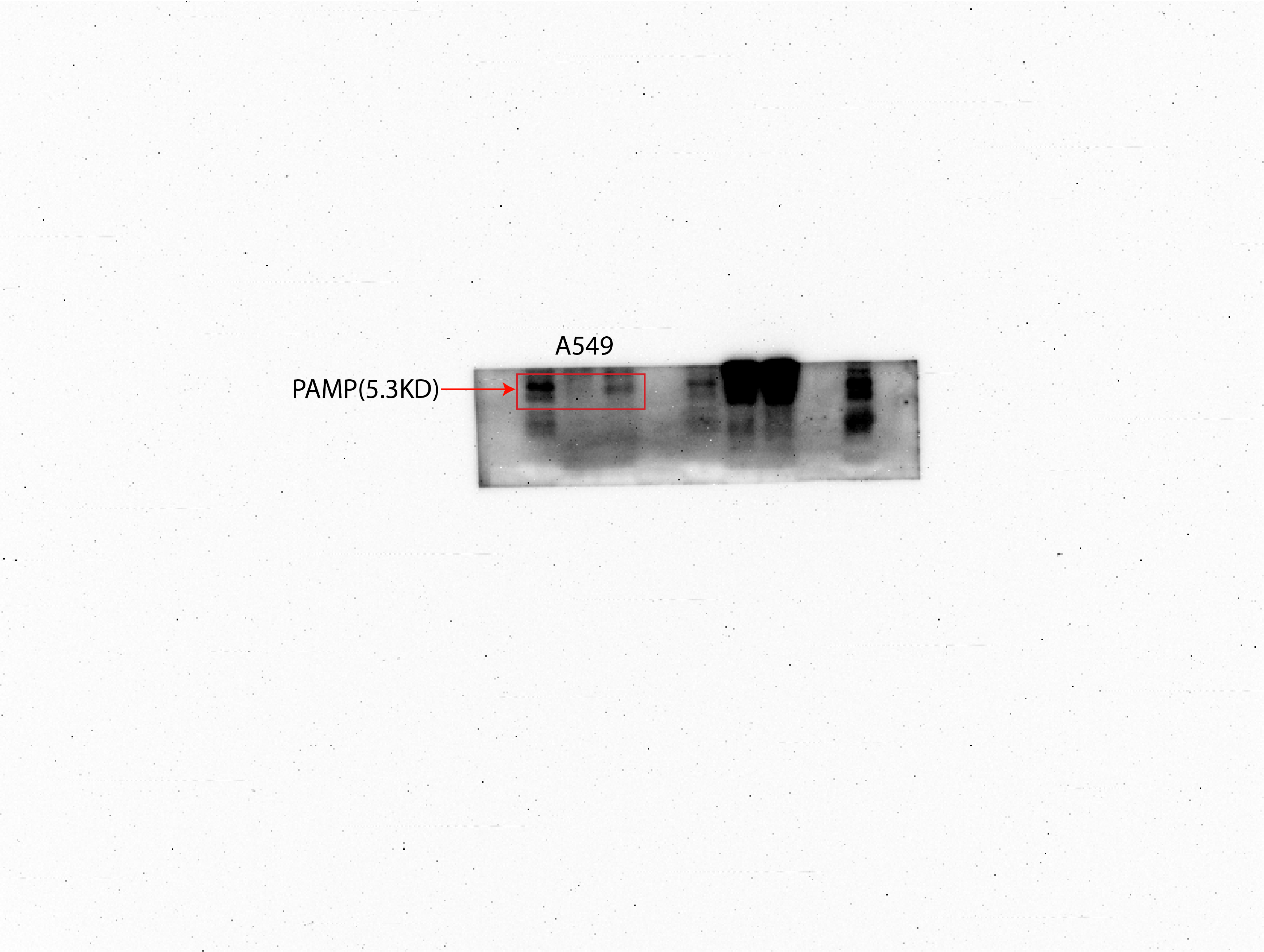

Supplement: Supplementary file 7 — Source data Fig. 4 [file 44321_2026_460_MOESM7_ESM.zip › Source data Figure4/FIG 4D/right-PAMP2.png]

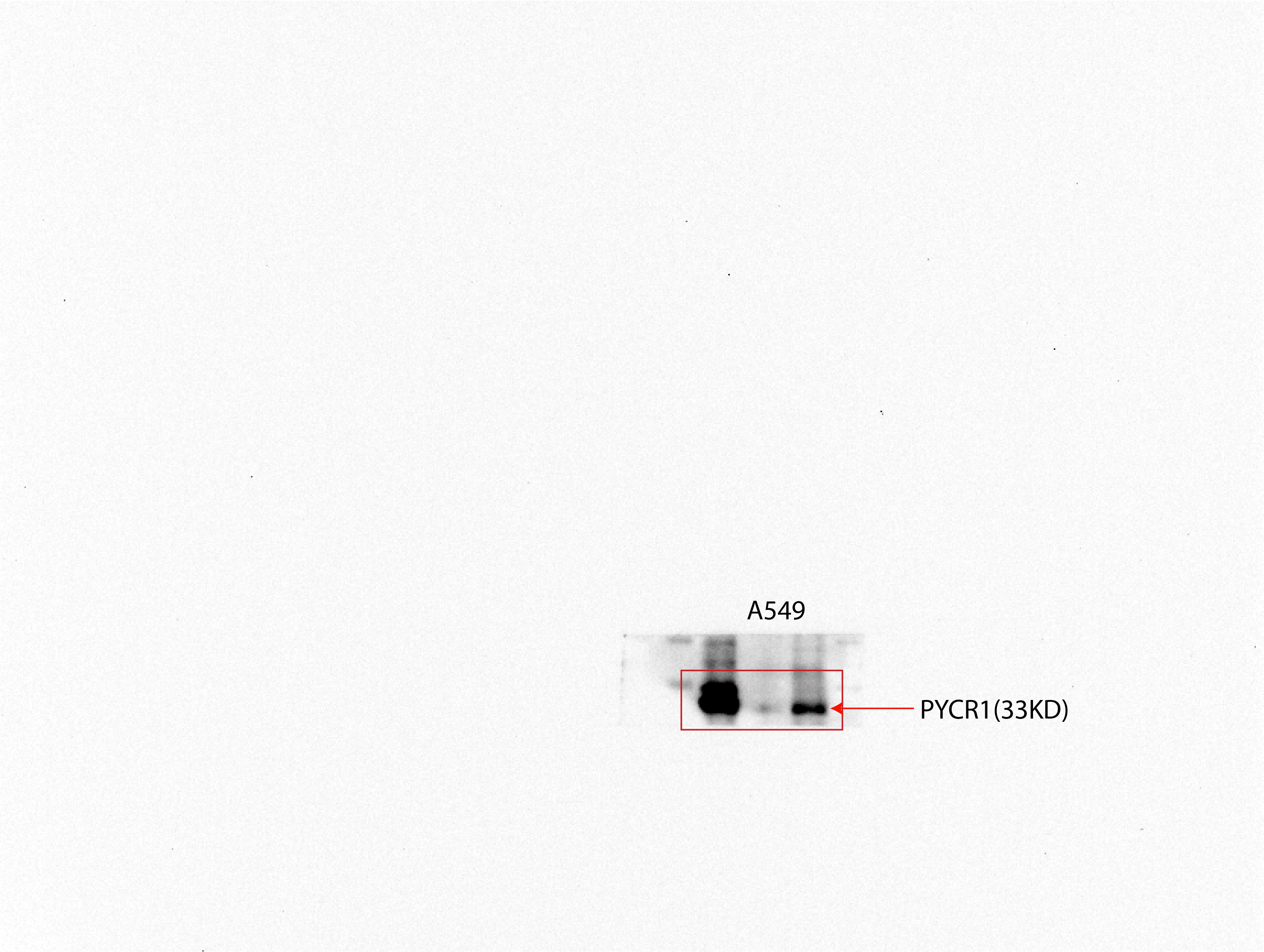

Supplement: Supplementary file 7 — Source data Fig. 4 [file 44321_2026_460_MOESM7_ESM.zip › Source data Figure4/FIG 4D/right-pycr1-1.png]

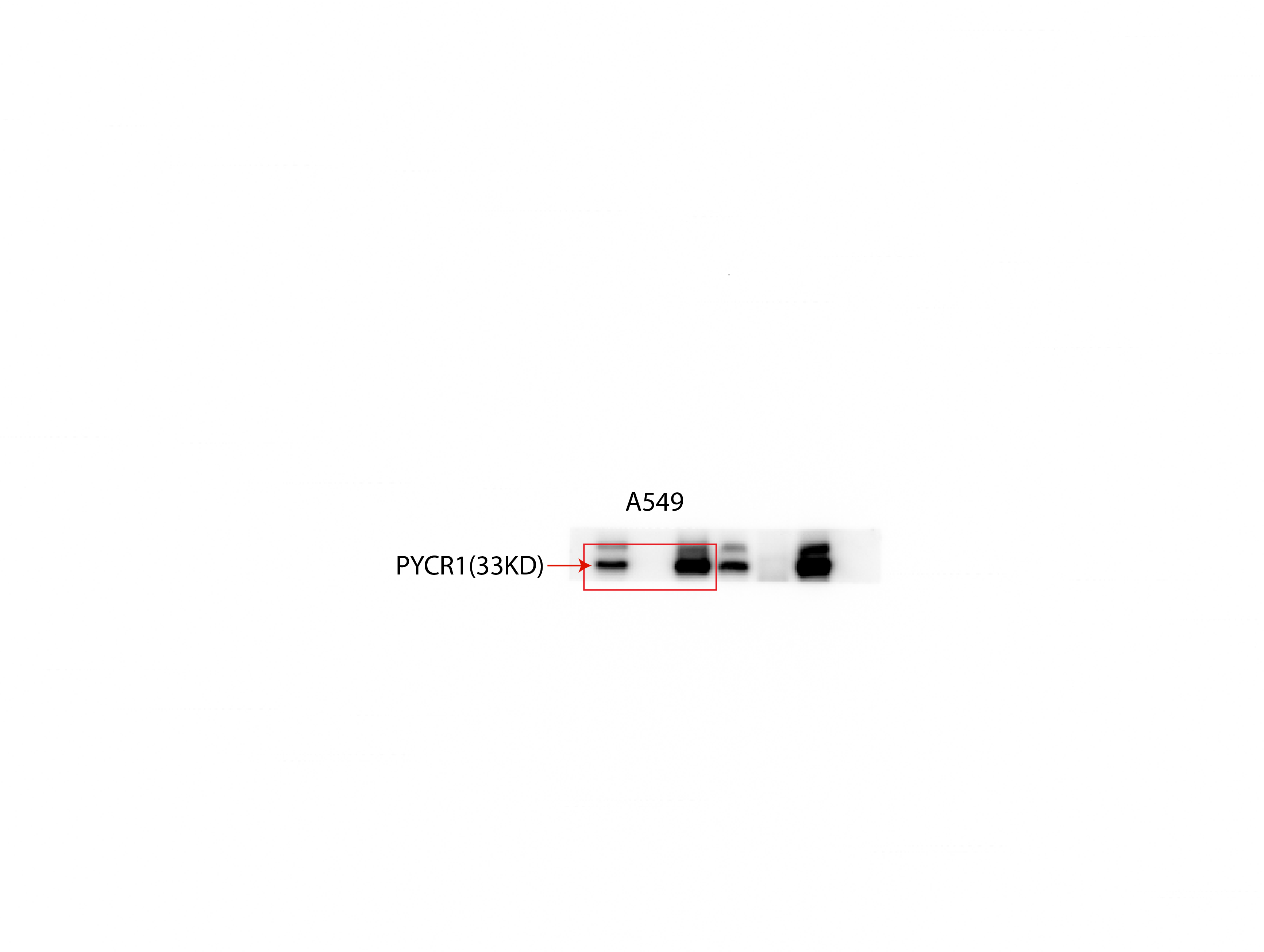

Supplement: Supplementary file 7 — Source data Fig. 4 [file 44321_2026_460_MOESM7_ESM.zip › Source data Figure4/FIG 4D/right-pycr1-2.png]

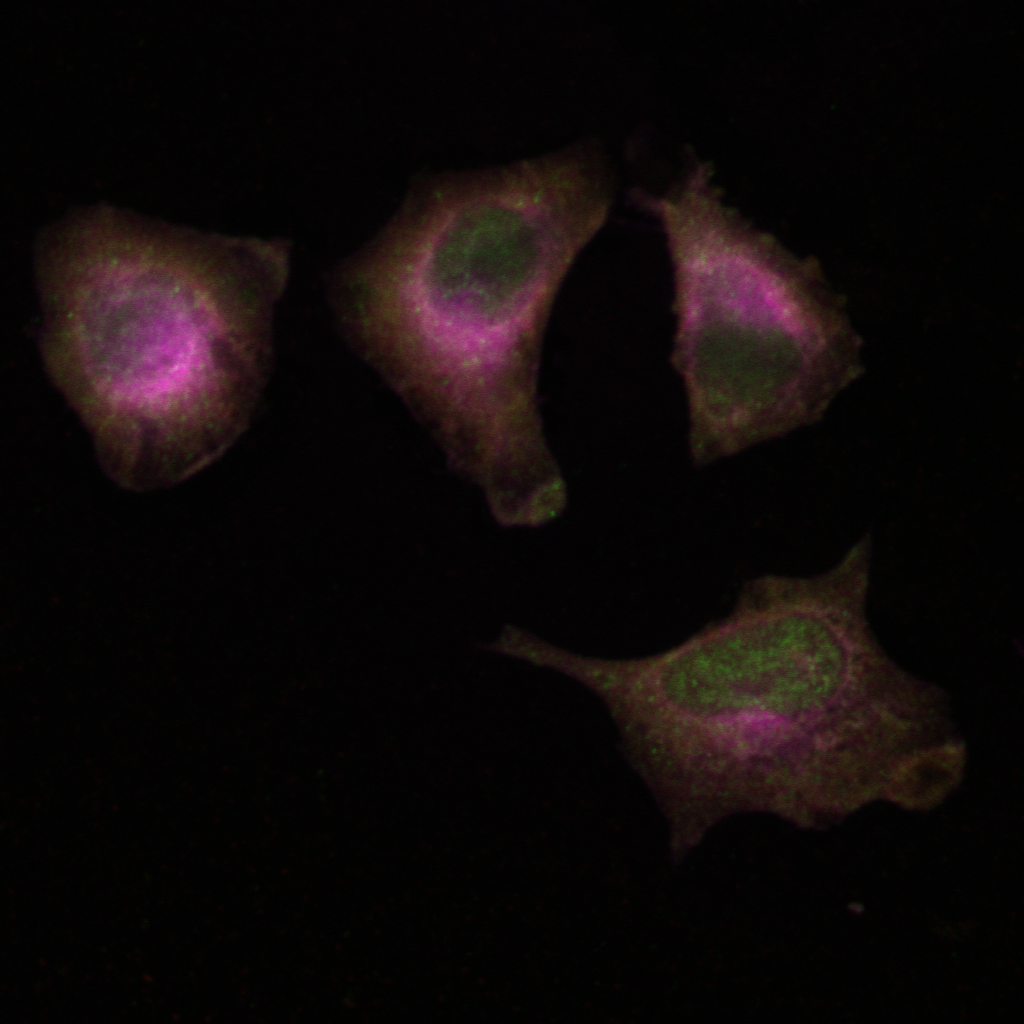

Supplement: Supplementary file 7 — Source data Fig. 4 [file 44321_2026_460_MOESM7_ESM.zip › Source data Figure4/FIG 4E/Merge.tif]

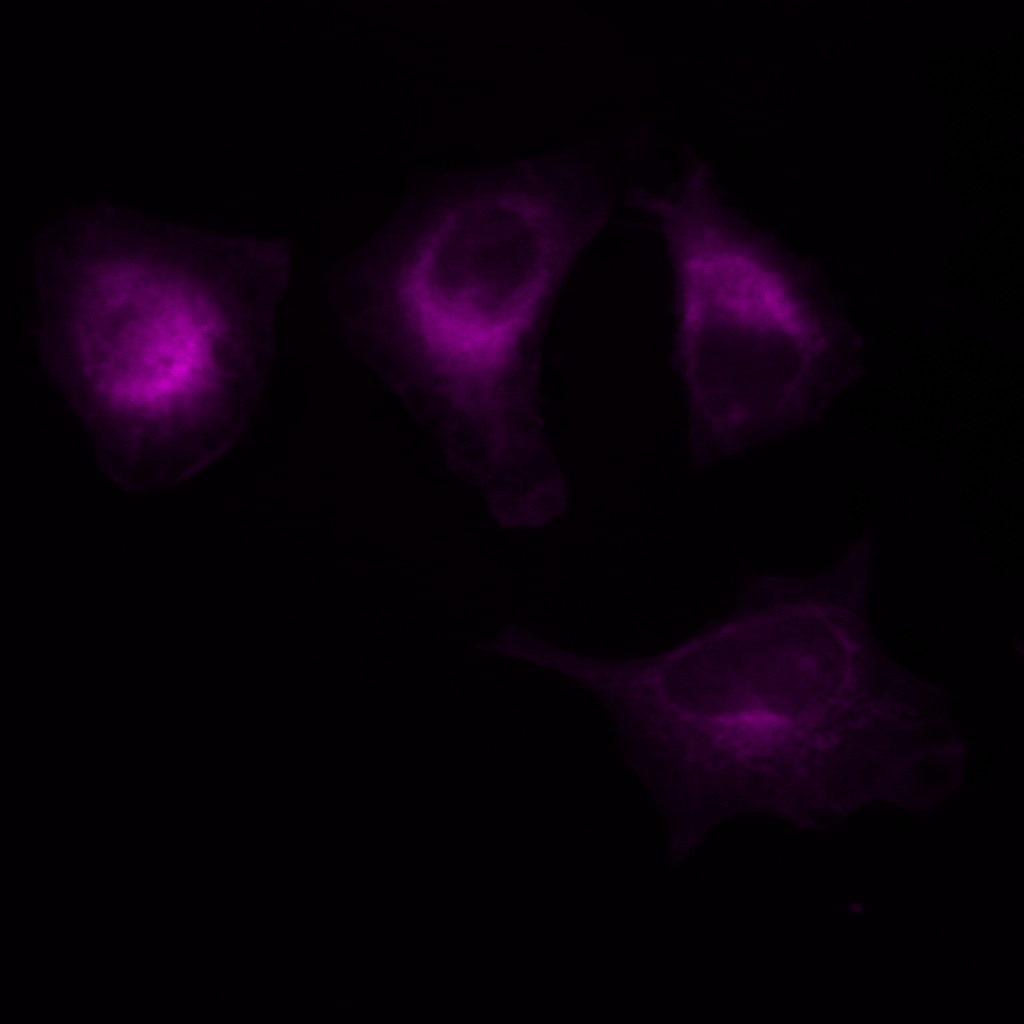

Supplement: Supplementary file 7 — Source data Fig. 4 [file 44321_2026_460_MOESM7_ESM.zip › Source data Figure4/FIG 4E/Mito-Tracker.tif]

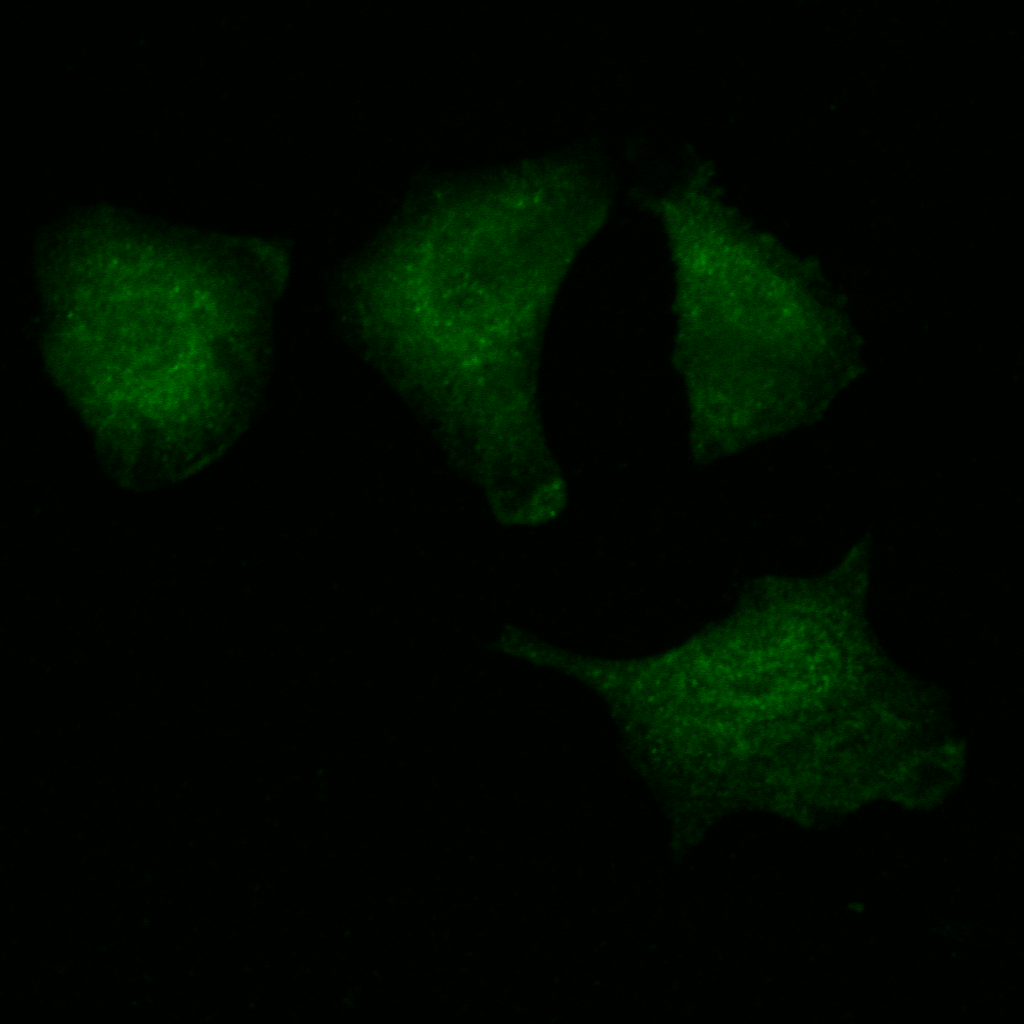

Supplement: Supplementary file 7 — Source data Fig. 4 [file 44321_2026_460_MOESM7_ESM.zip › Source data Figure4/FIG 4E/PAMP.tif]

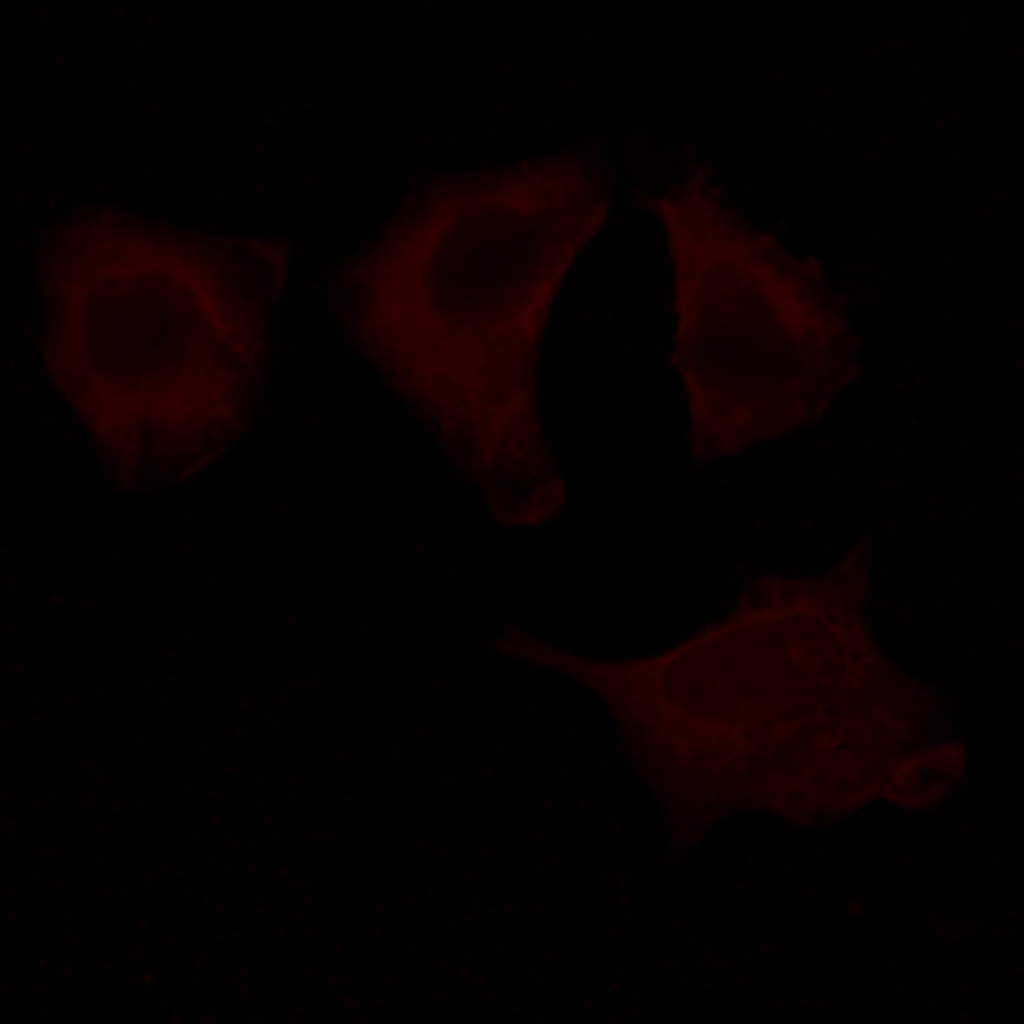

Supplement: Supplementary file 7 — Source data Fig. 4 [file 44321_2026_460_MOESM7_ESM.zip › Source data Figure4/FIG 4E/PYCR1.tif]

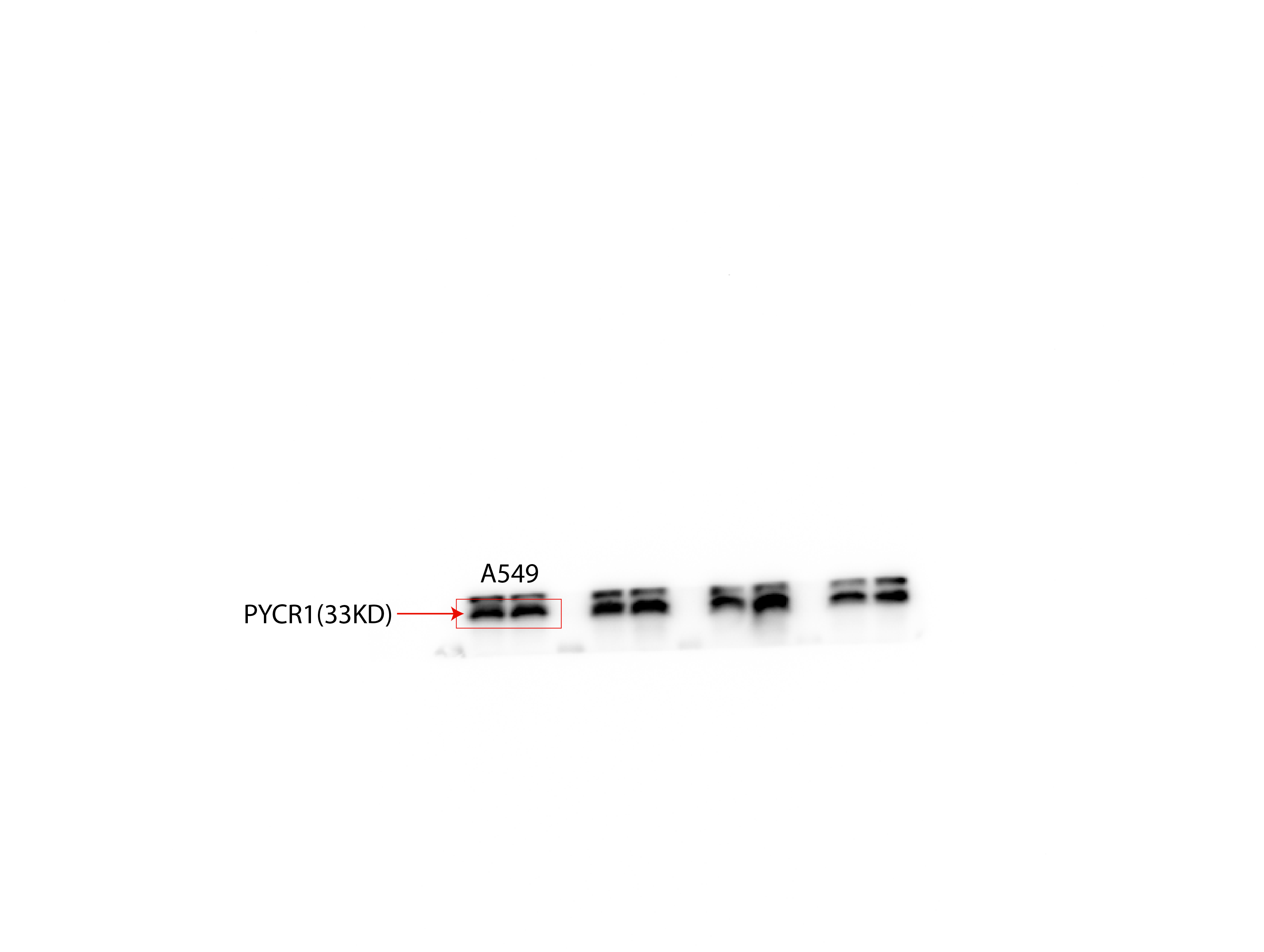

Supplement: Supplementary file 7 — Source data Fig. 4 [file 44321_2026_460_MOESM7_ESM.zip › Source data Figure4/FIG 4I/2024-05-11_Pycr3_8bit.png]

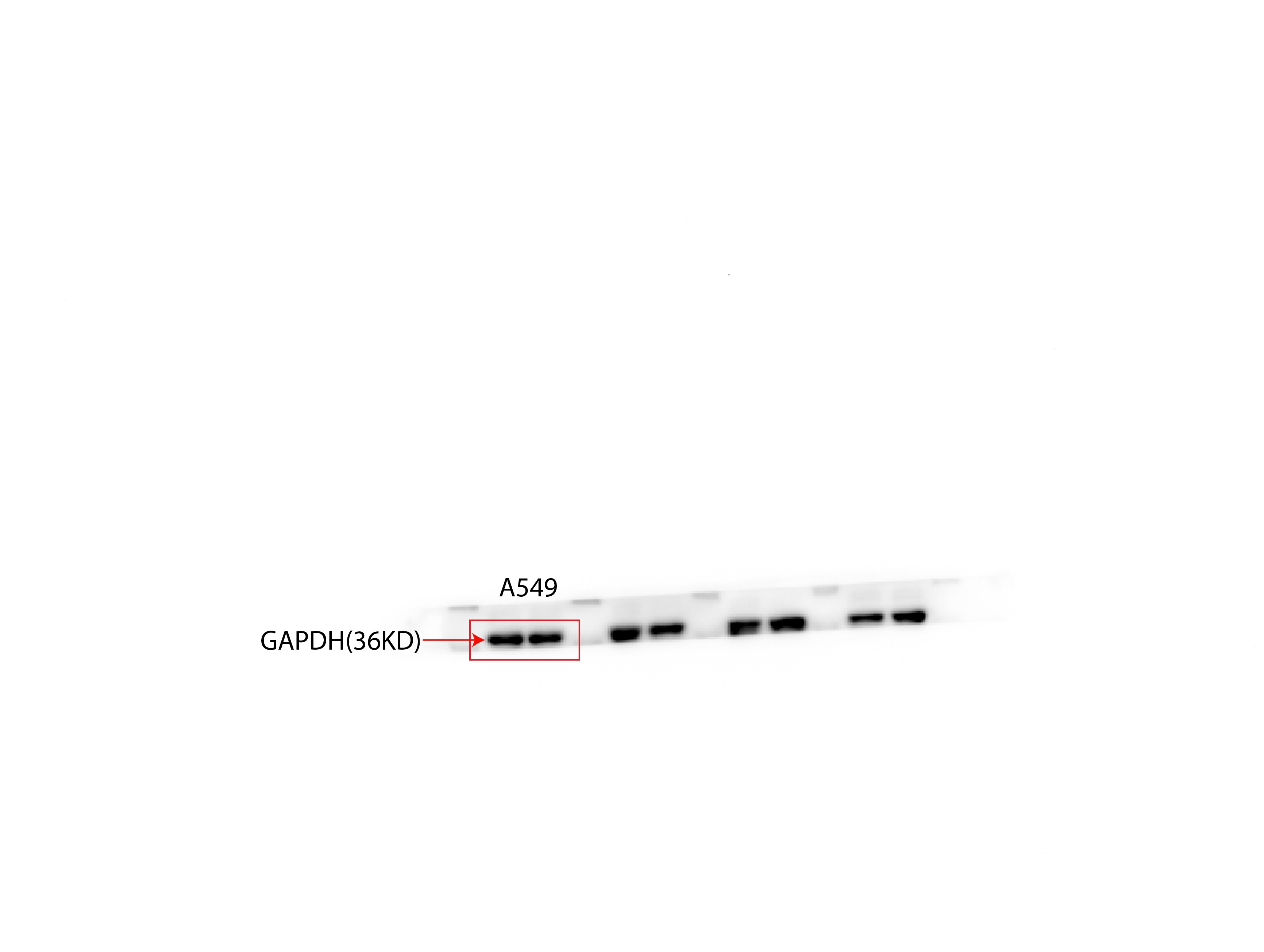

Supplement: Supplementary file 7 — Source data Fig. 4 [file 44321_2026_460_MOESM7_ESM.zip › Source data Figure4/FIG 4I/A549-GAPDH.png]

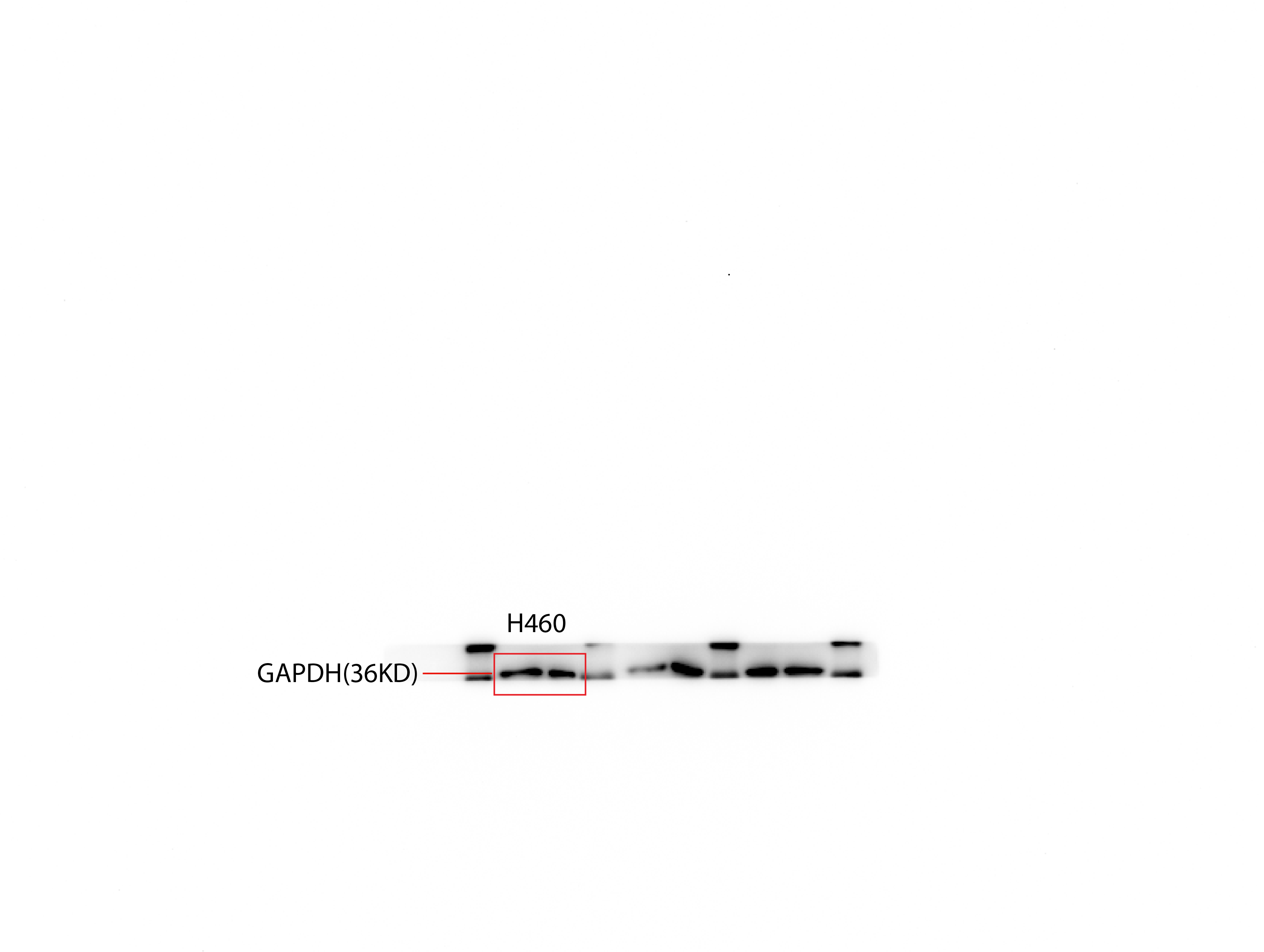

Supplement: Supplementary file 7 — Source data Fig. 4 [file 44321_2026_460_MOESM7_ESM.zip › Source data Figure4/FIG 4I/H460-GAPDH.png]

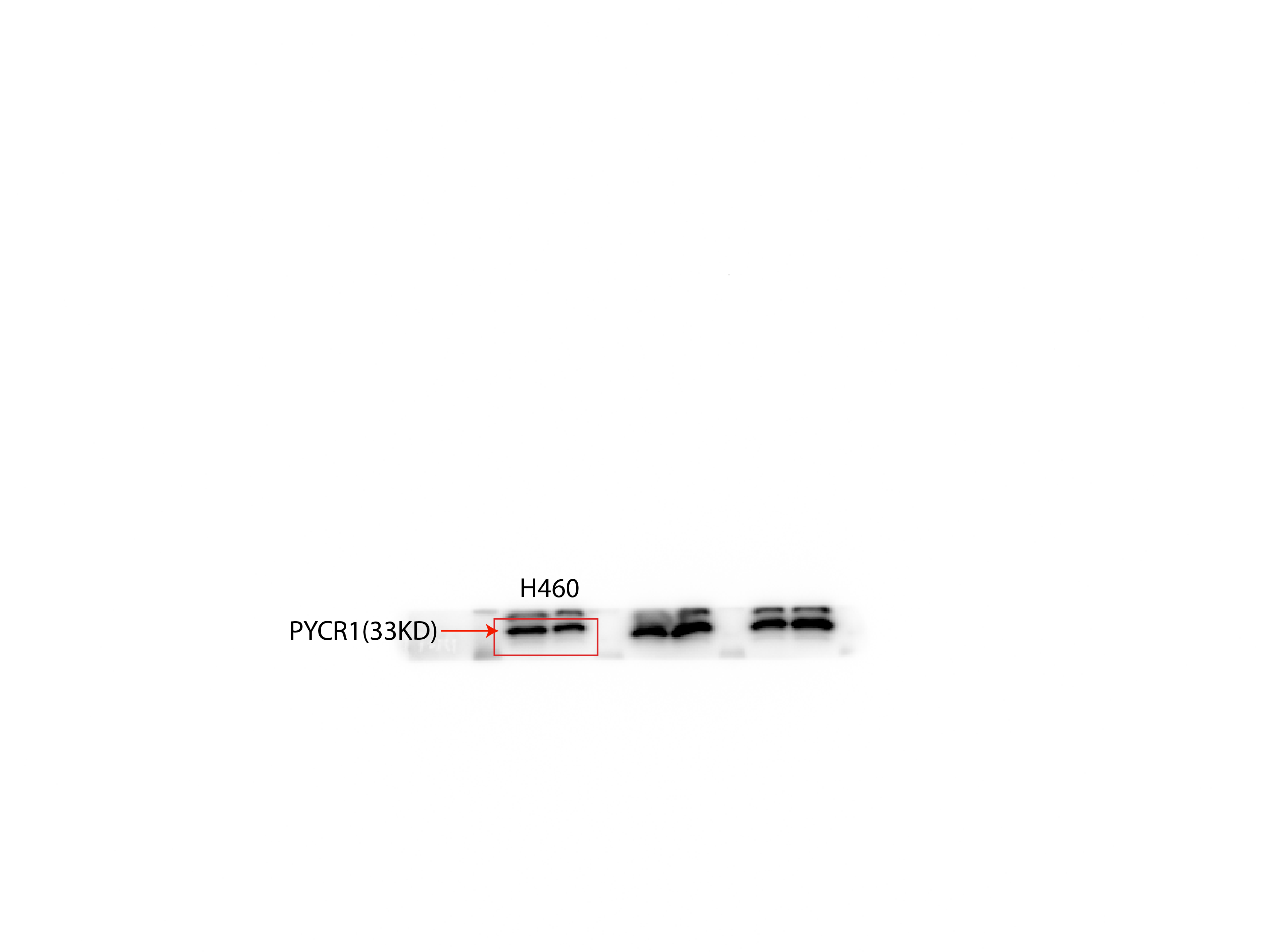

Supplement: Supplementary file 7 — Source data Fig. 4 [file 44321_2026_460_MOESM7_ESM.zip › Source data Figure4/FIG 4I/H460-PYCR1.png]

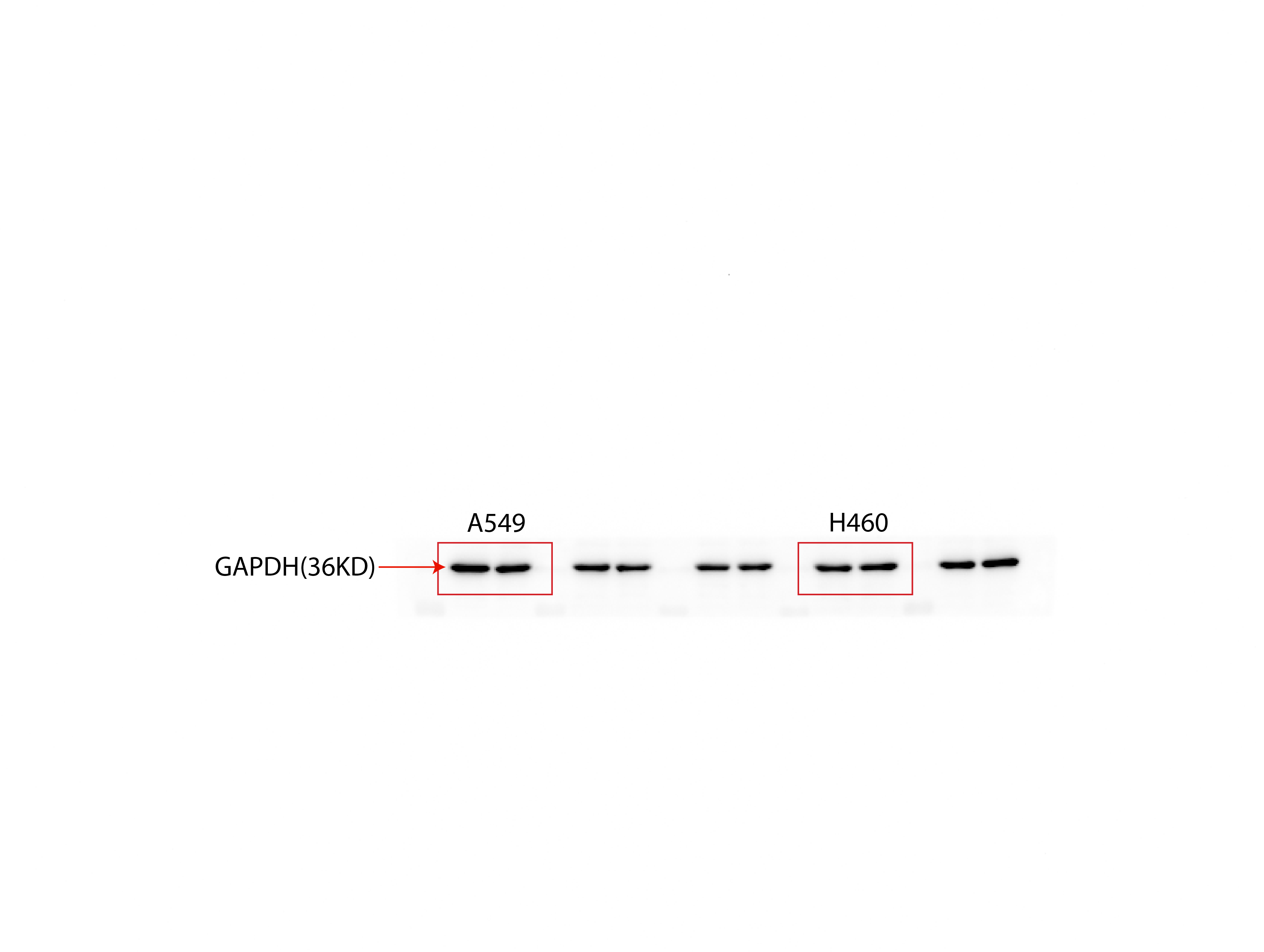

Supplement: Supplementary file 7 — Source data Fig. 4 [file 44321_2026_460_MOESM7_ESM.zip › Source data Figure4/FIG 4J/GAPDH.png]

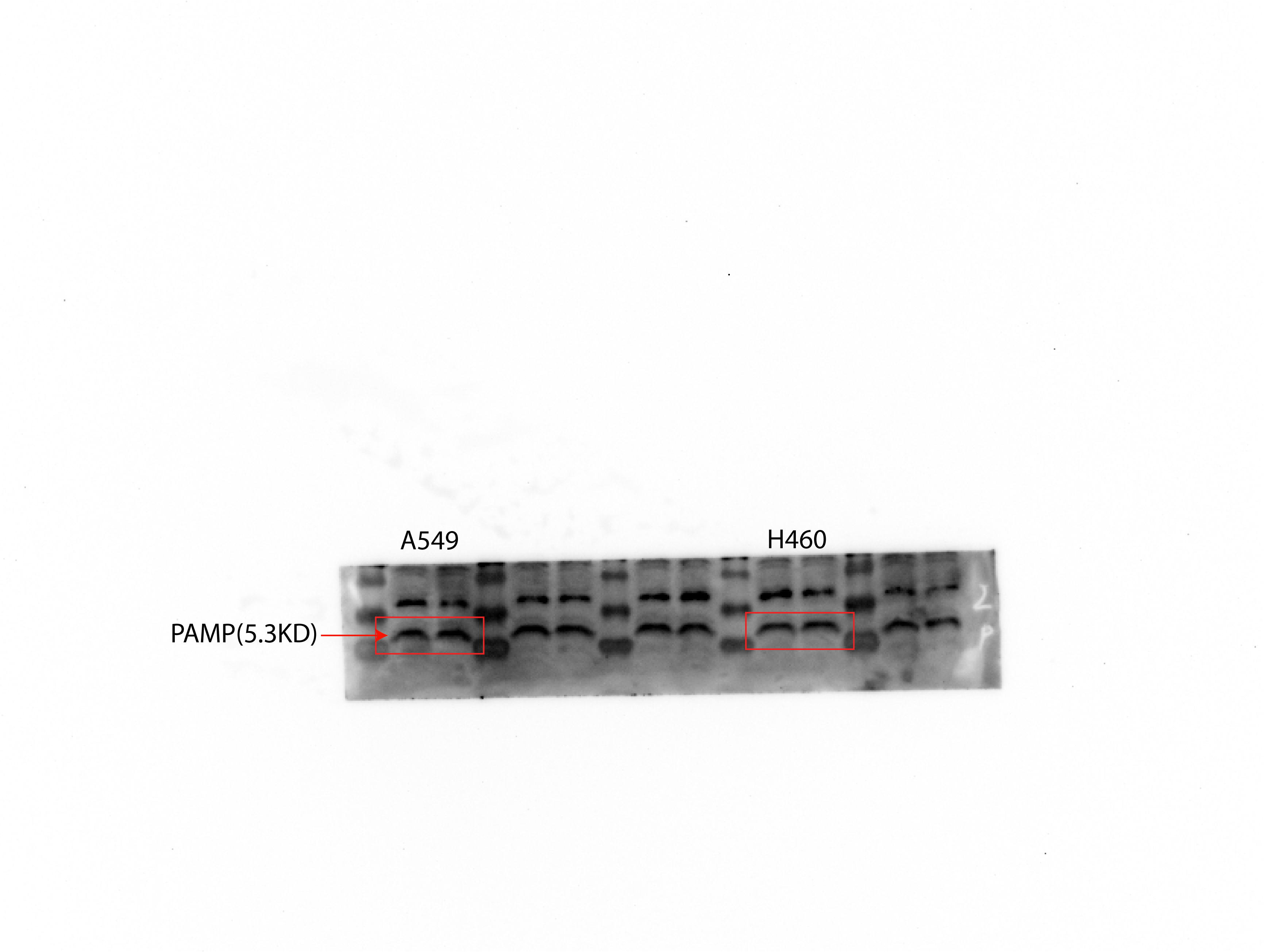

Supplement: Supplementary file 7 — Source data Fig. 4 [file 44321_2026_460_MOESM7_ESM.zip › Source data Figure4/FIG 4J/PAMP.png]

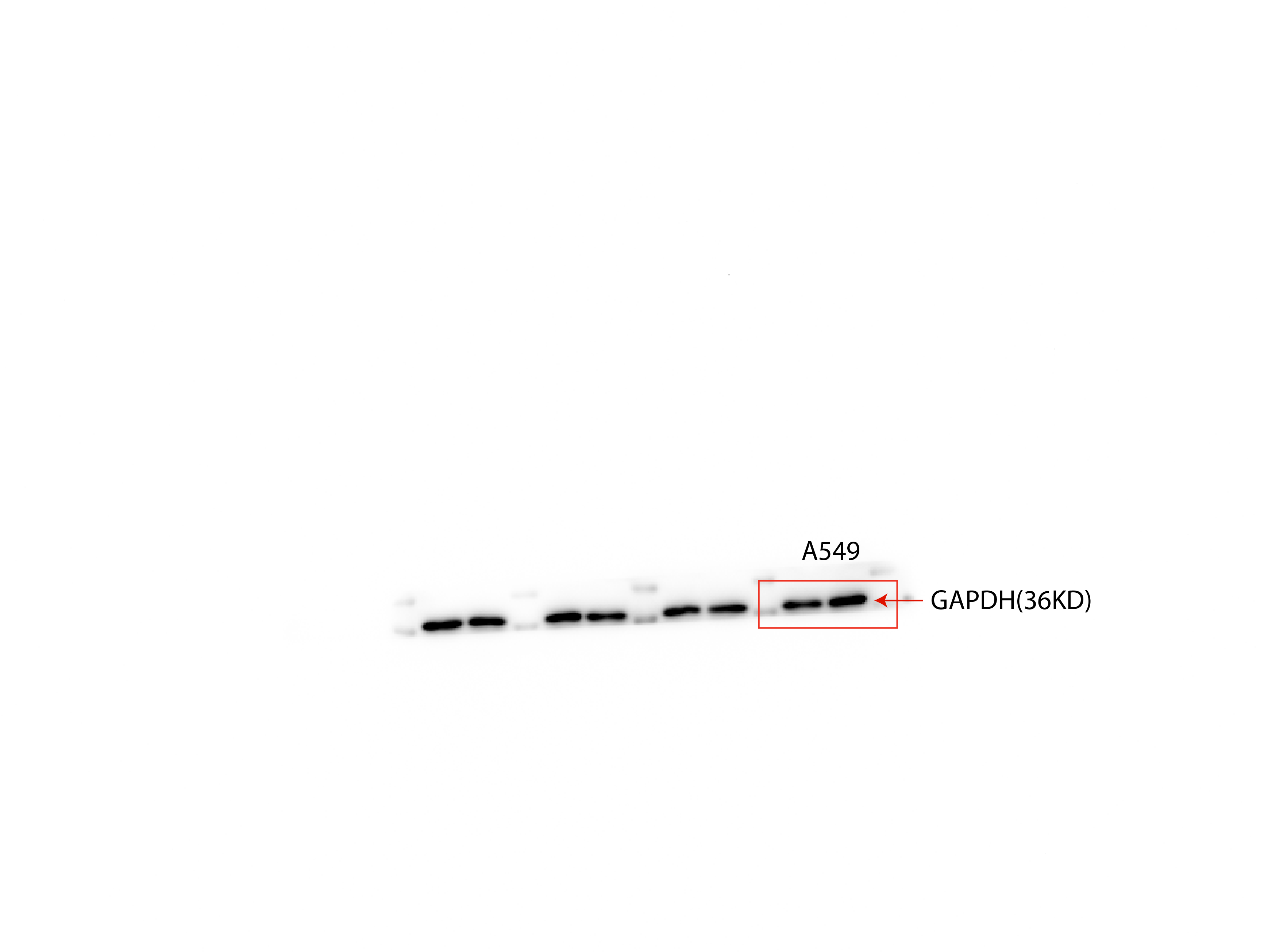

Supplement: Supplementary file 8 — Source data Fig. 5 [file 44321_2026_460_MOESM8_ESM.zip › Source data Figure5/FIG 5C/A549-GAPDH.png]

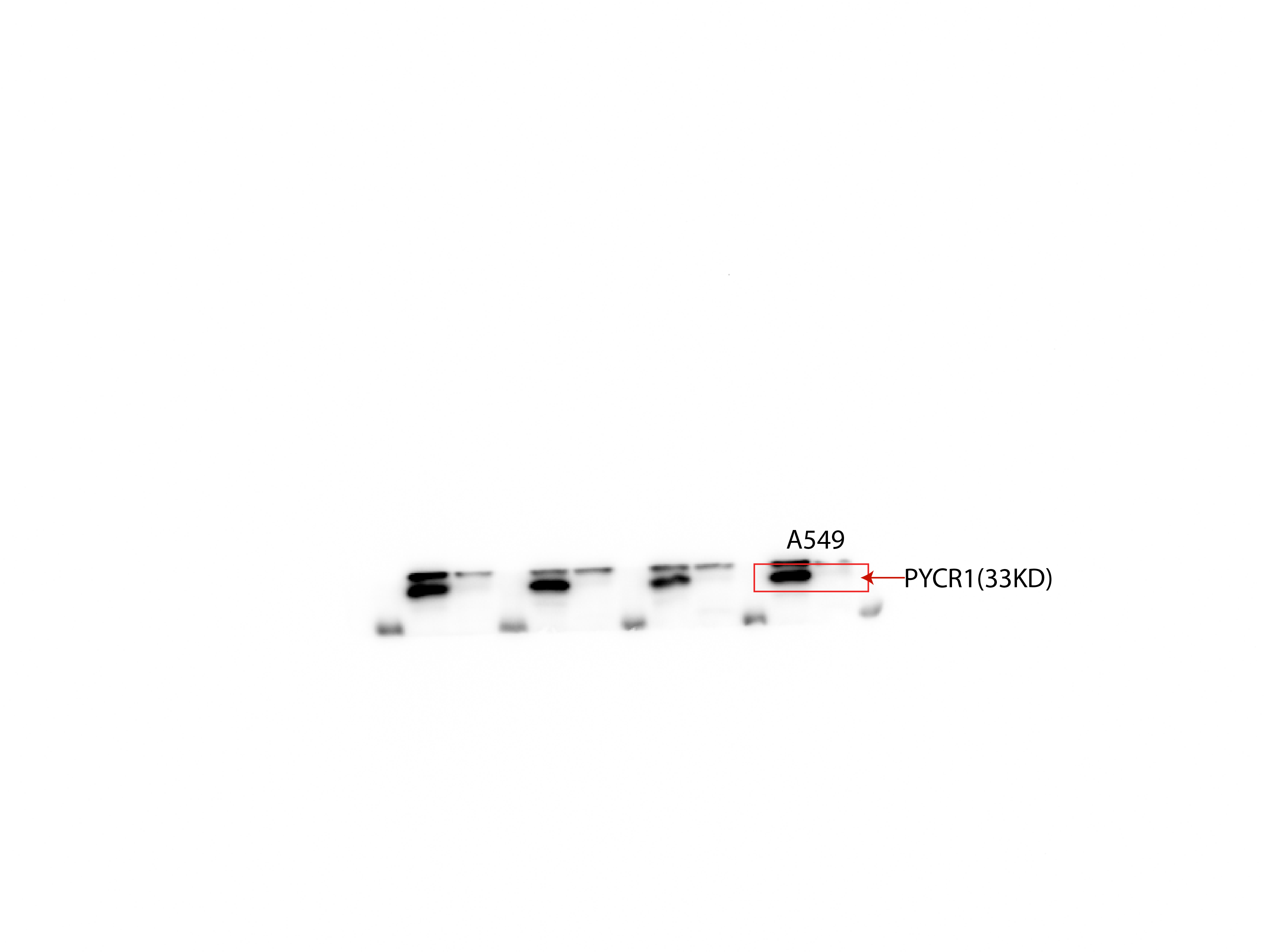

Supplement: Supplementary file 8 — Source data Fig. 5 [file 44321_2026_460_MOESM8_ESM.zip › Source data Figure5/FIG 5C/A549-PYCR1.png]

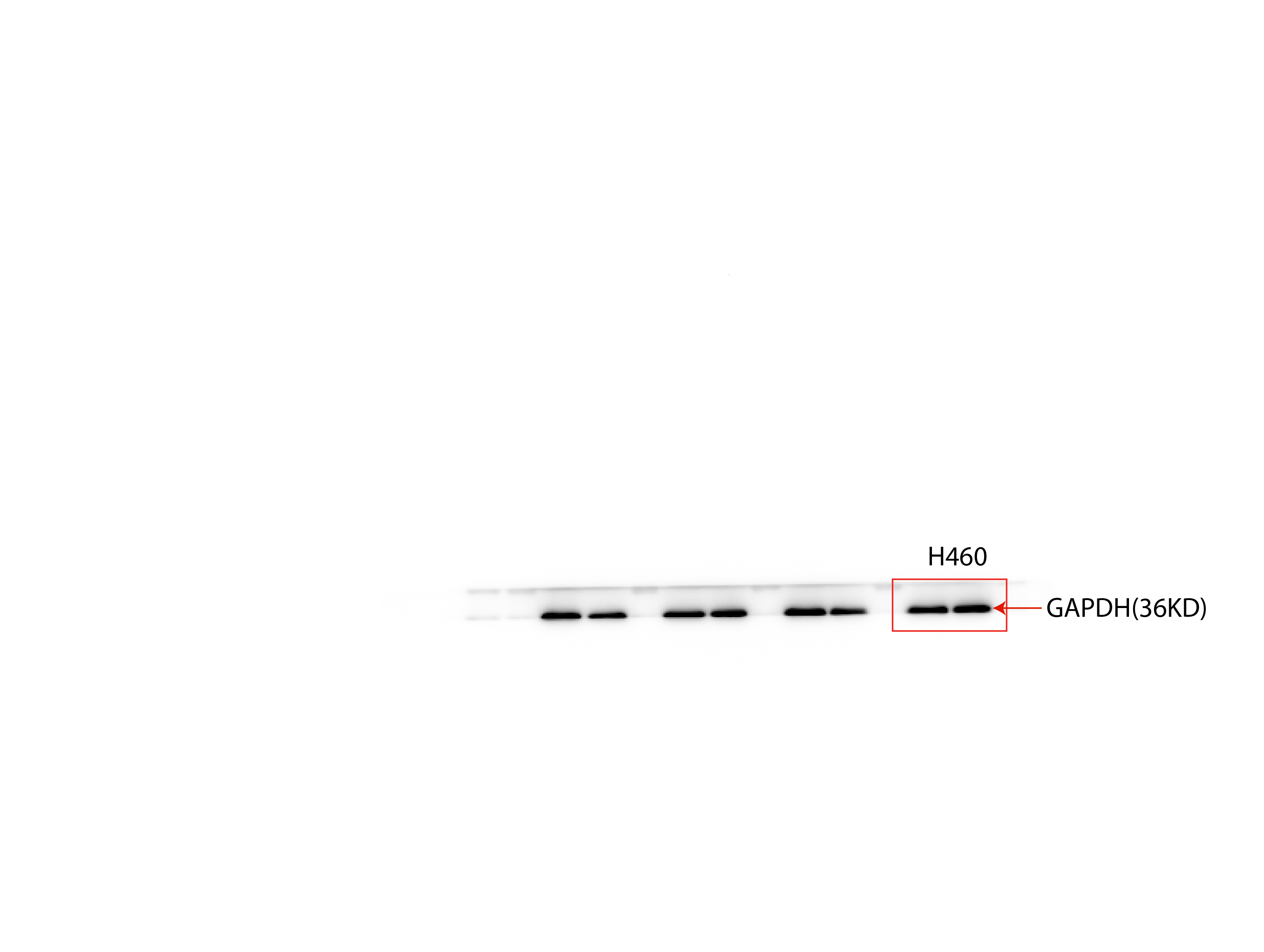

Supplement: Supplementary file 8 — Source data Fig. 5 [file 44321_2026_460_MOESM8_ESM.zip › Source data Figure5/FIG 5C/H460-GAPDH.png]

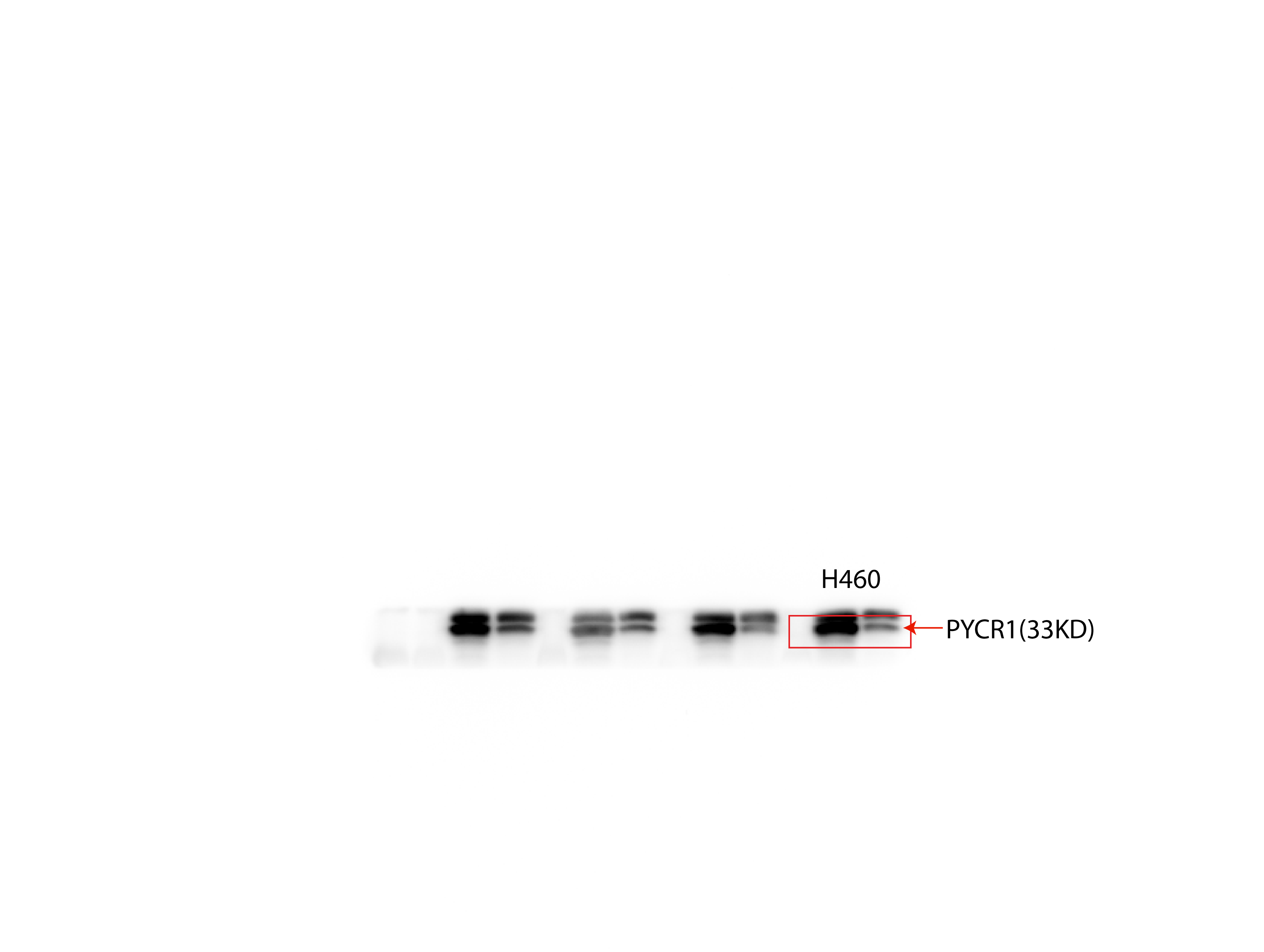

Supplement: Supplementary file 8 — Source data Fig. 5 [file 44321_2026_460_MOESM8_ESM.zip › Source data Figure5/FIG 5C/H460-PYCR1.png]

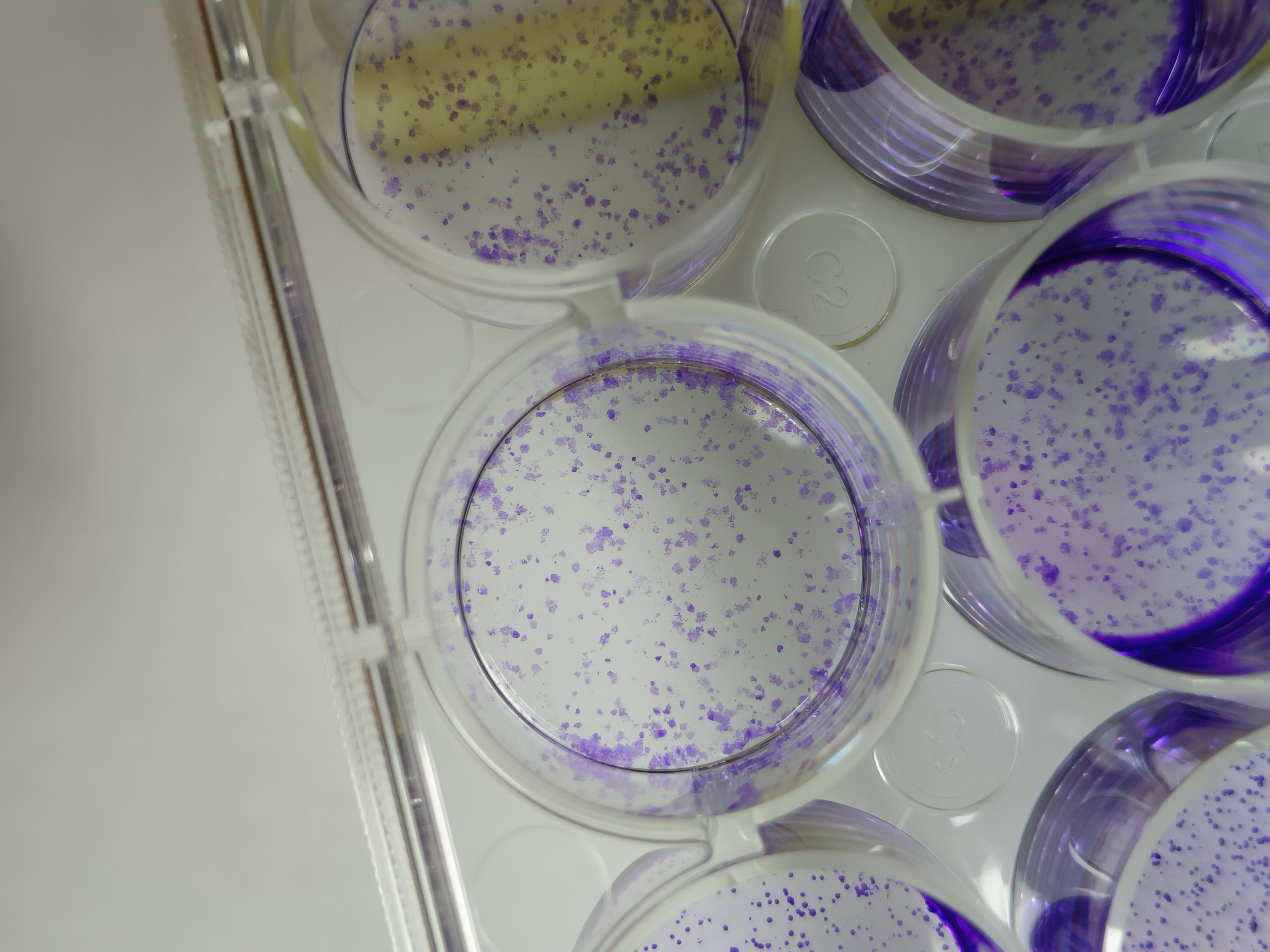

Supplement: Supplementary file 8 — Source data Fig. 5 [file 44321_2026_460_MOESM8_ESM.zip › Source data Figure5/FIG 5F/A549 KO PYCR1.jpg]

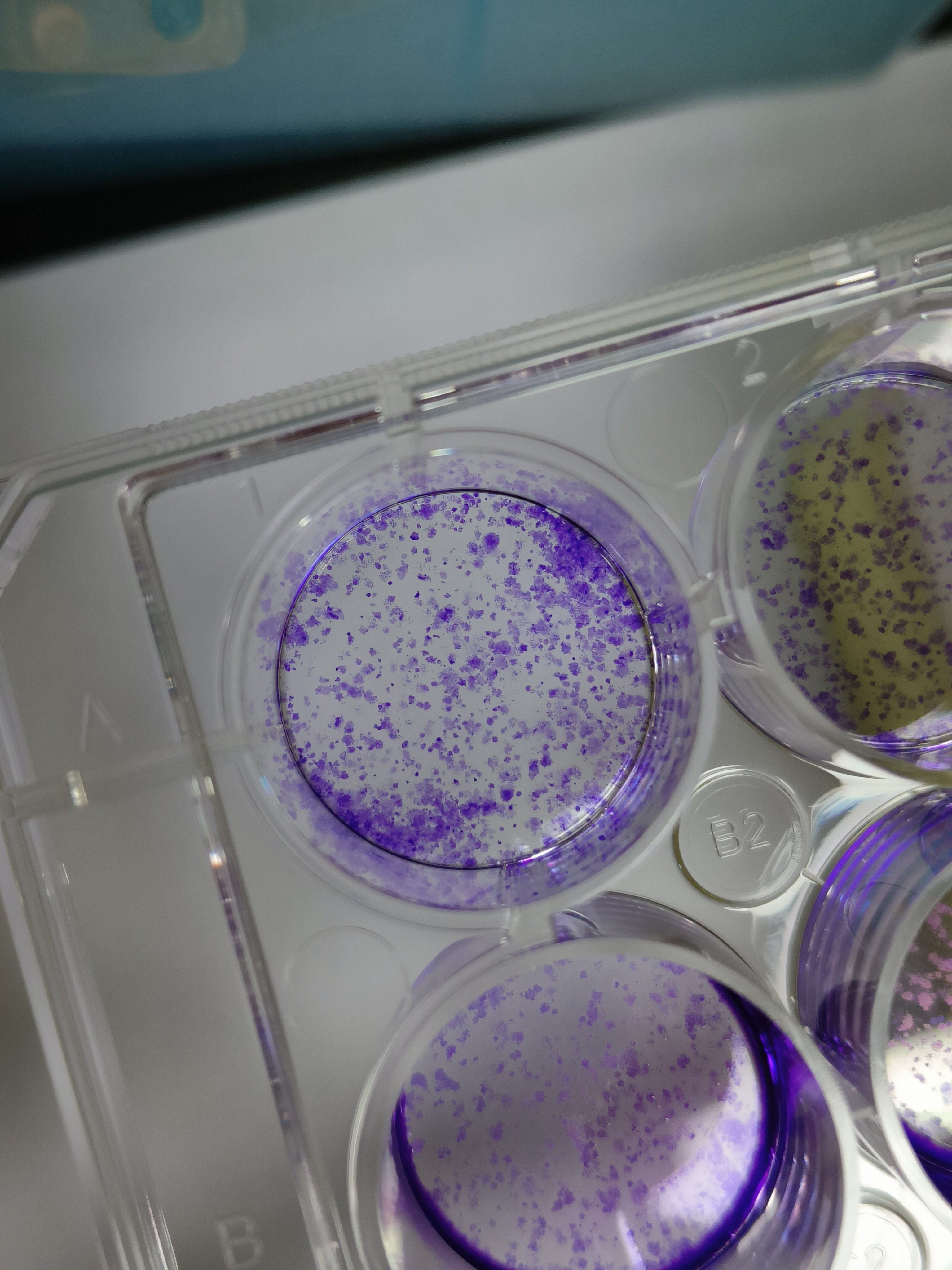

Supplement: Supplementary file 8 — Source data Fig. 5 [file 44321_2026_460_MOESM8_ESM.zip › Source data Figure5/FIG 5F/A549-lacZ.jpg]

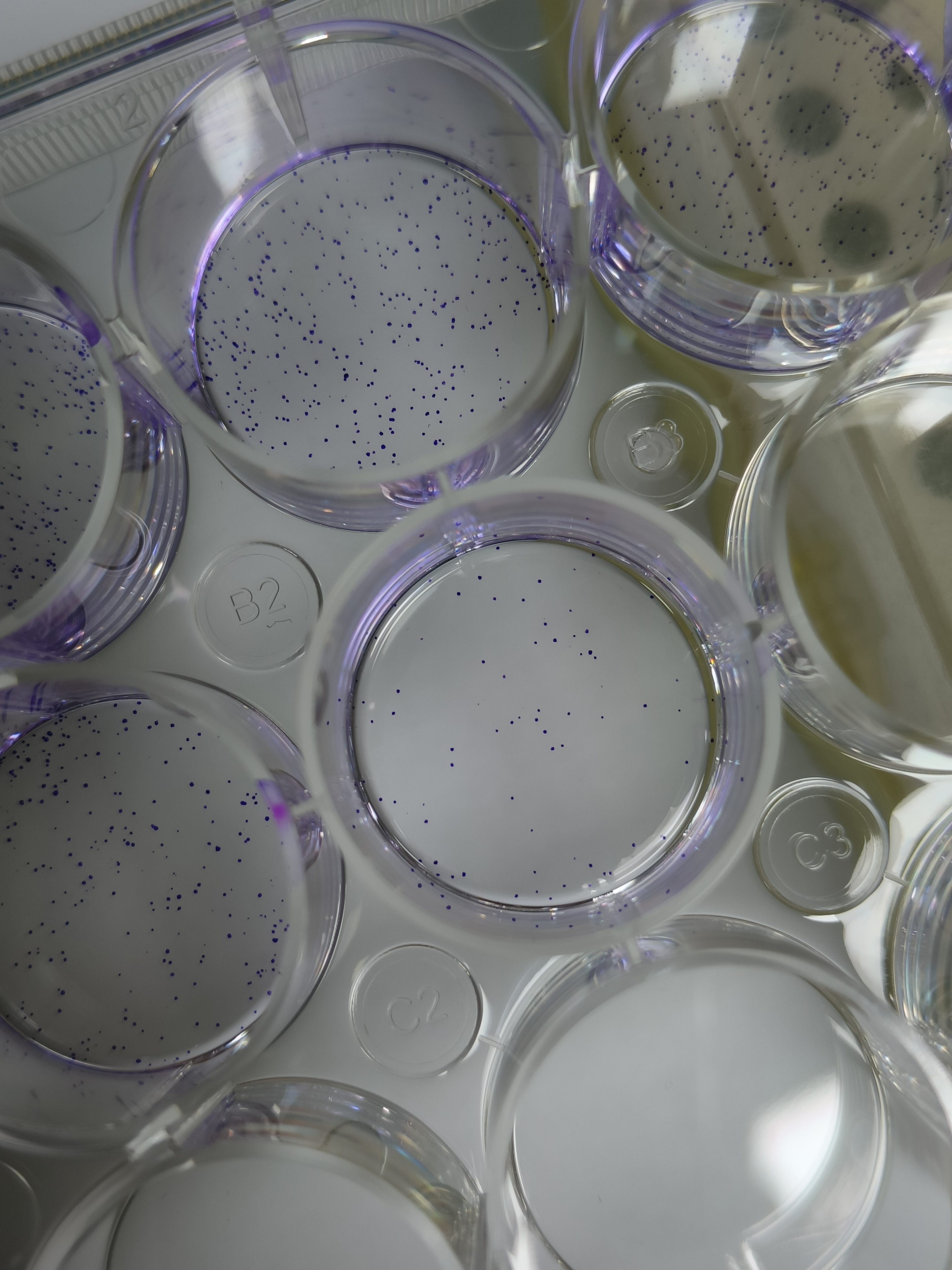

Supplement: Supplementary file 8 — Source data Fig. 5 [file 44321_2026_460_MOESM8_ESM.zip › Source data Figure5/FIG 5F/H460 KO PYCR1.jpg]

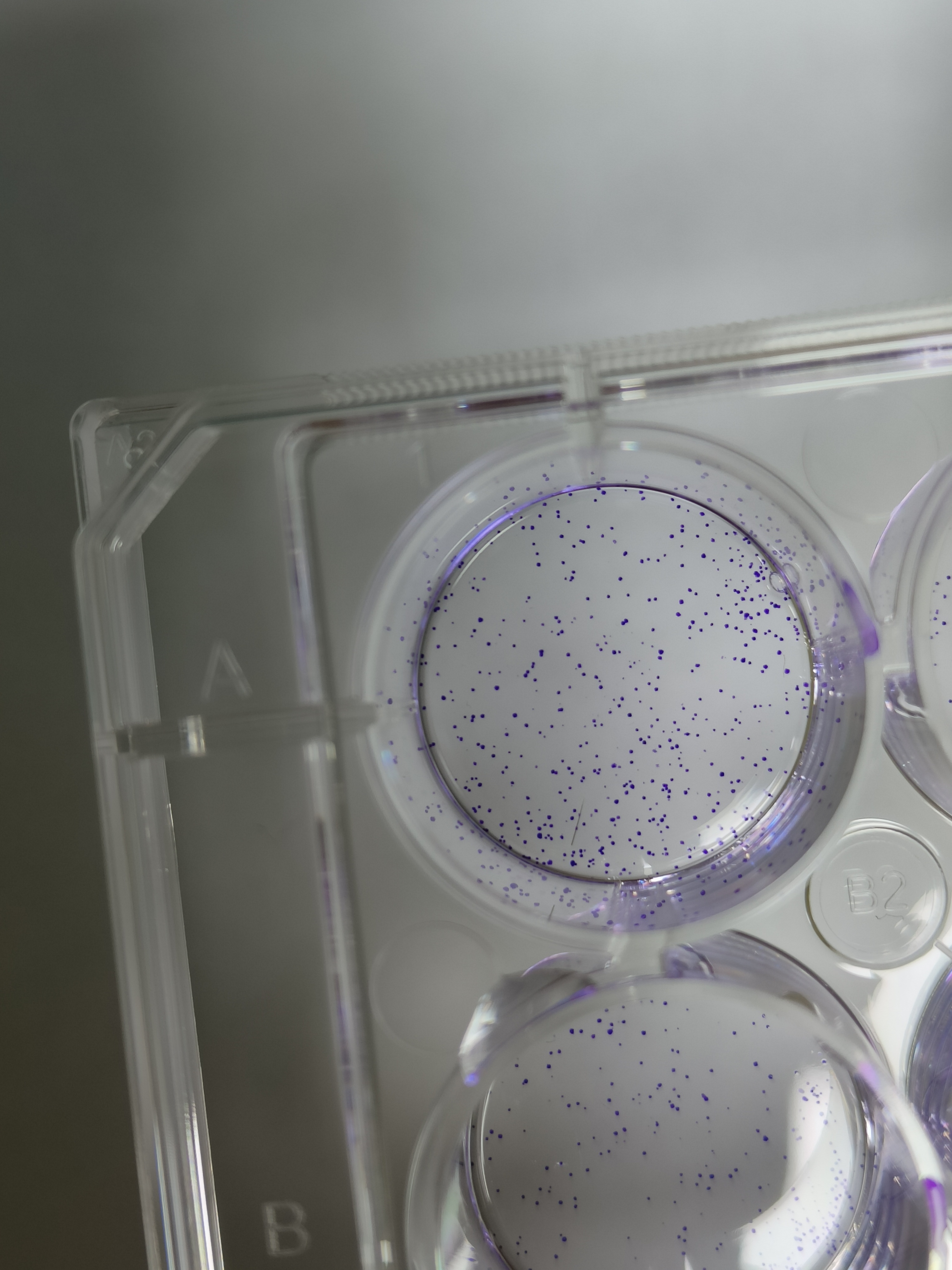

Supplement: Supplementary file 8 — Source data Fig. 5 [file 44321_2026_460_MOESM8_ESM.zip › Source data Figure5/FIG 5F/H460-lacZ.jpg]

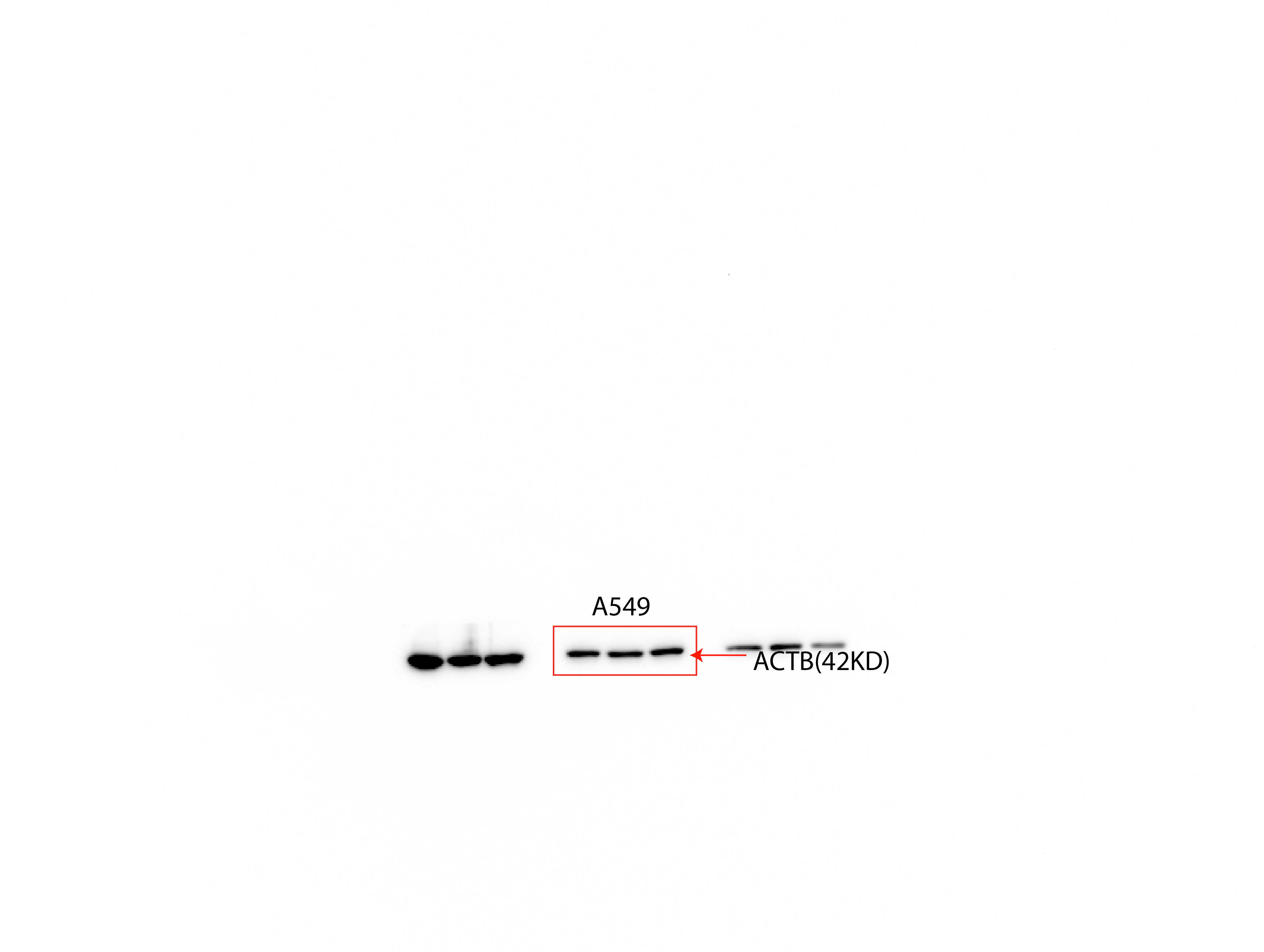

Supplement: Supplementary file 8 — Source data Fig. 5 [file 44321_2026_460_MOESM8_ESM.zip › Source data Figure5/FIG 5I/ACTB.png]

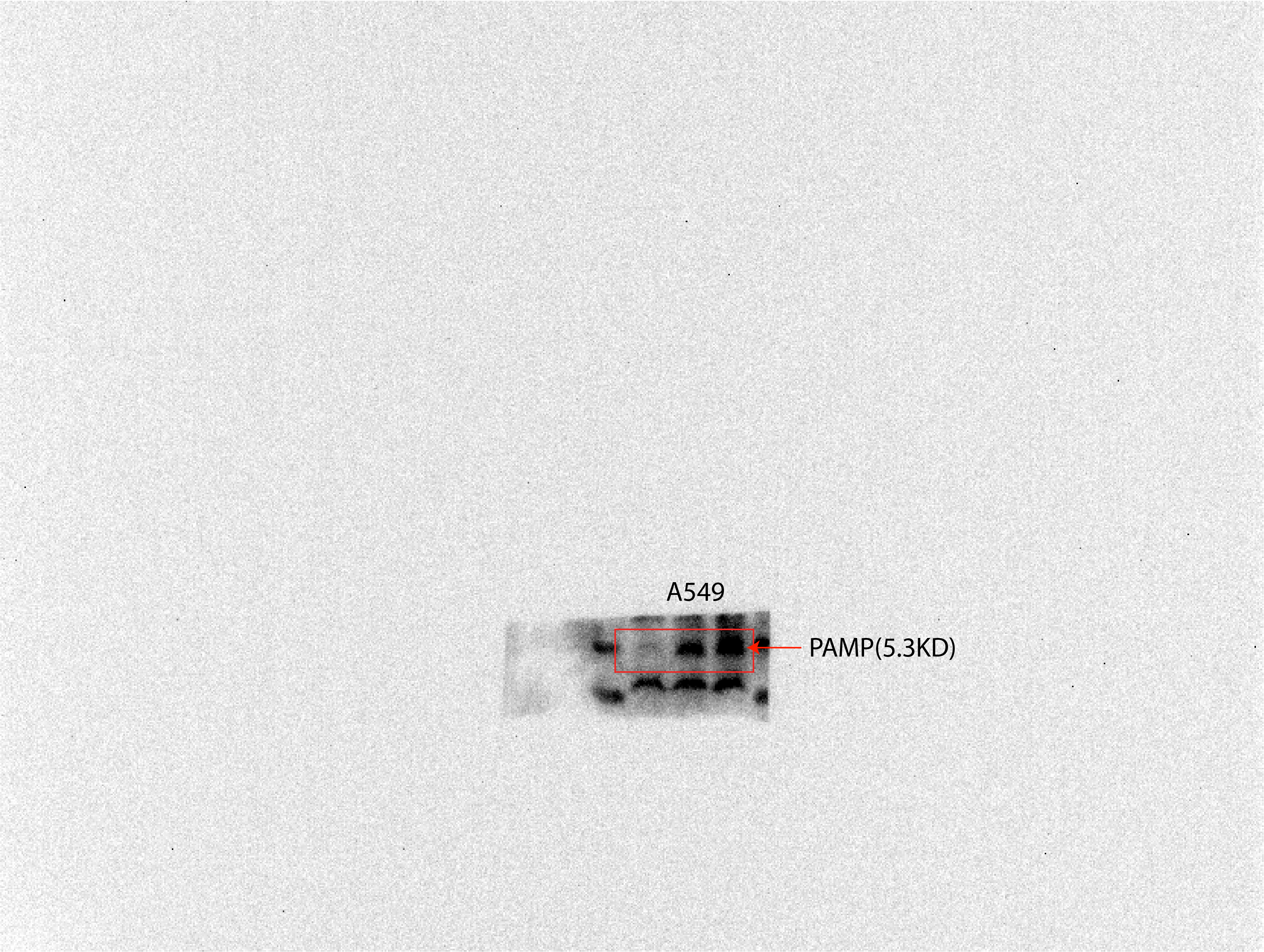

Supplement: Supplementary file 8 — Source data Fig. 5 [file 44321_2026_460_MOESM8_ESM.zip › Source data Figure5/FIG 5I/PAMP.png]

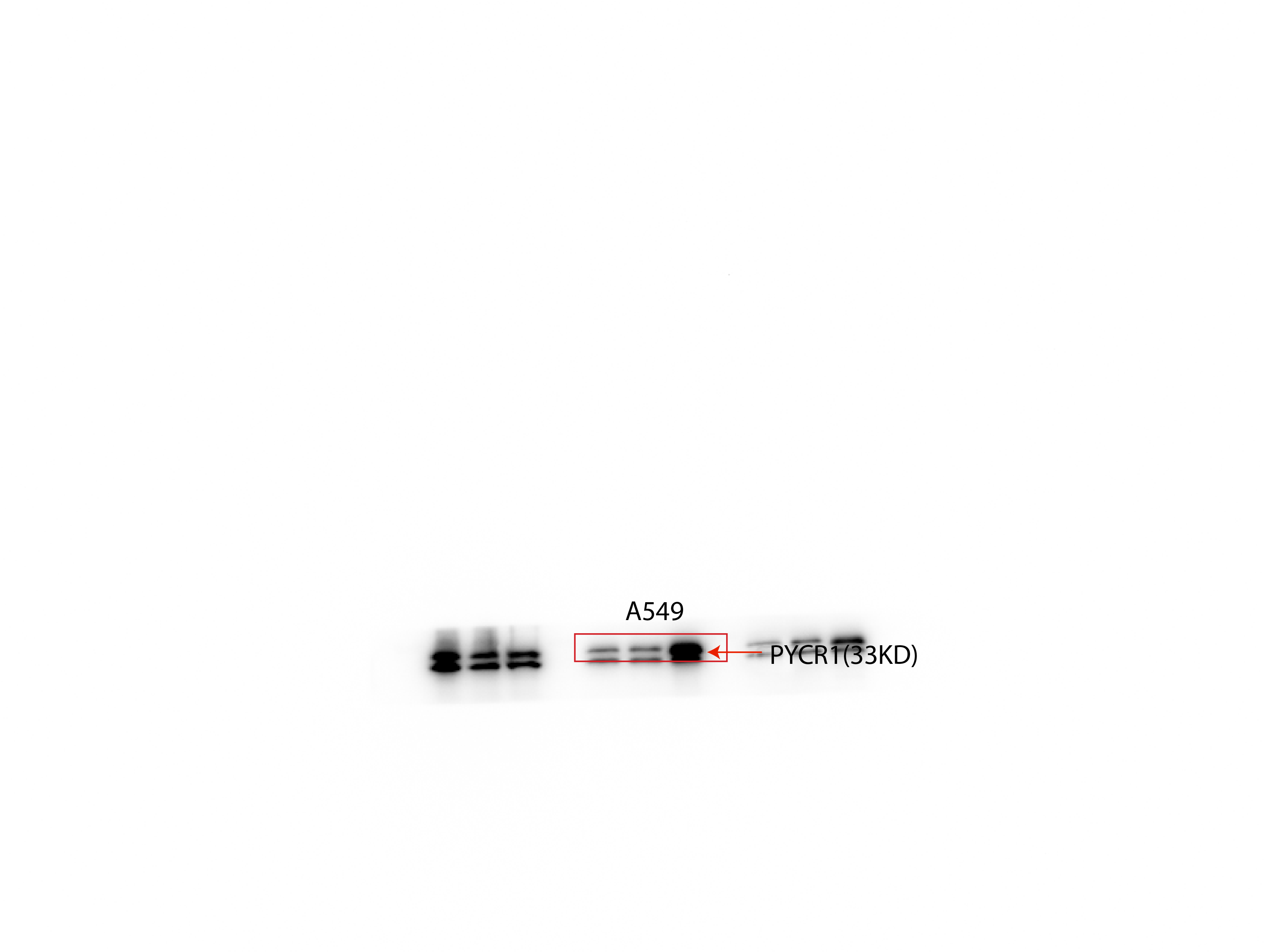

Supplement: Supplementary file 8 — Source data Fig. 5 [file 44321_2026_460_MOESM8_ESM.zip › Source data Figure5/FIG 5I/PYCR1.png]

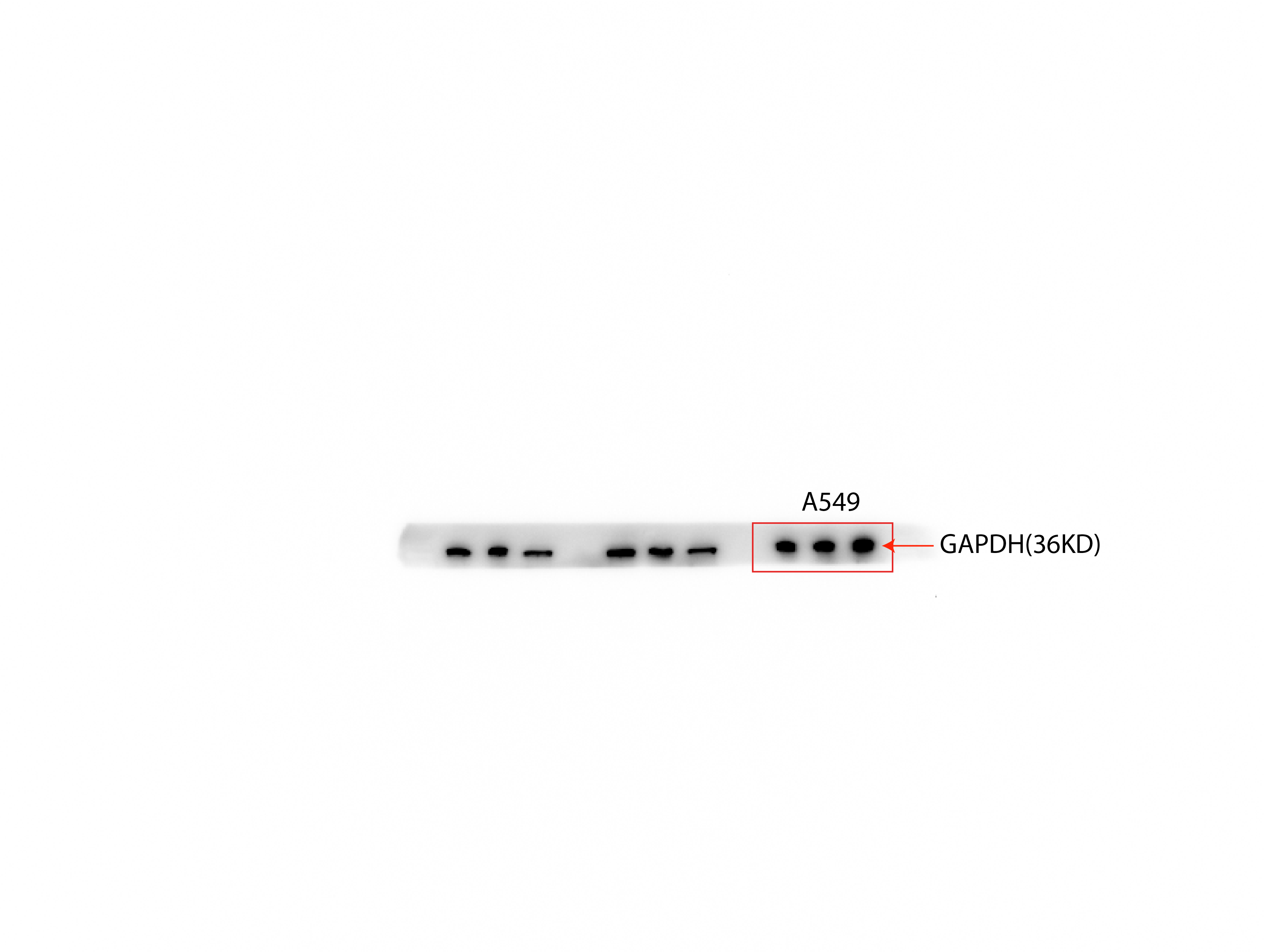

Supplement: Supplementary file 8 — Source data Fig. 5 [file 44321_2026_460_MOESM8_ESM.zip › Source data Figure5/FIG 5K/GAPDH.png]

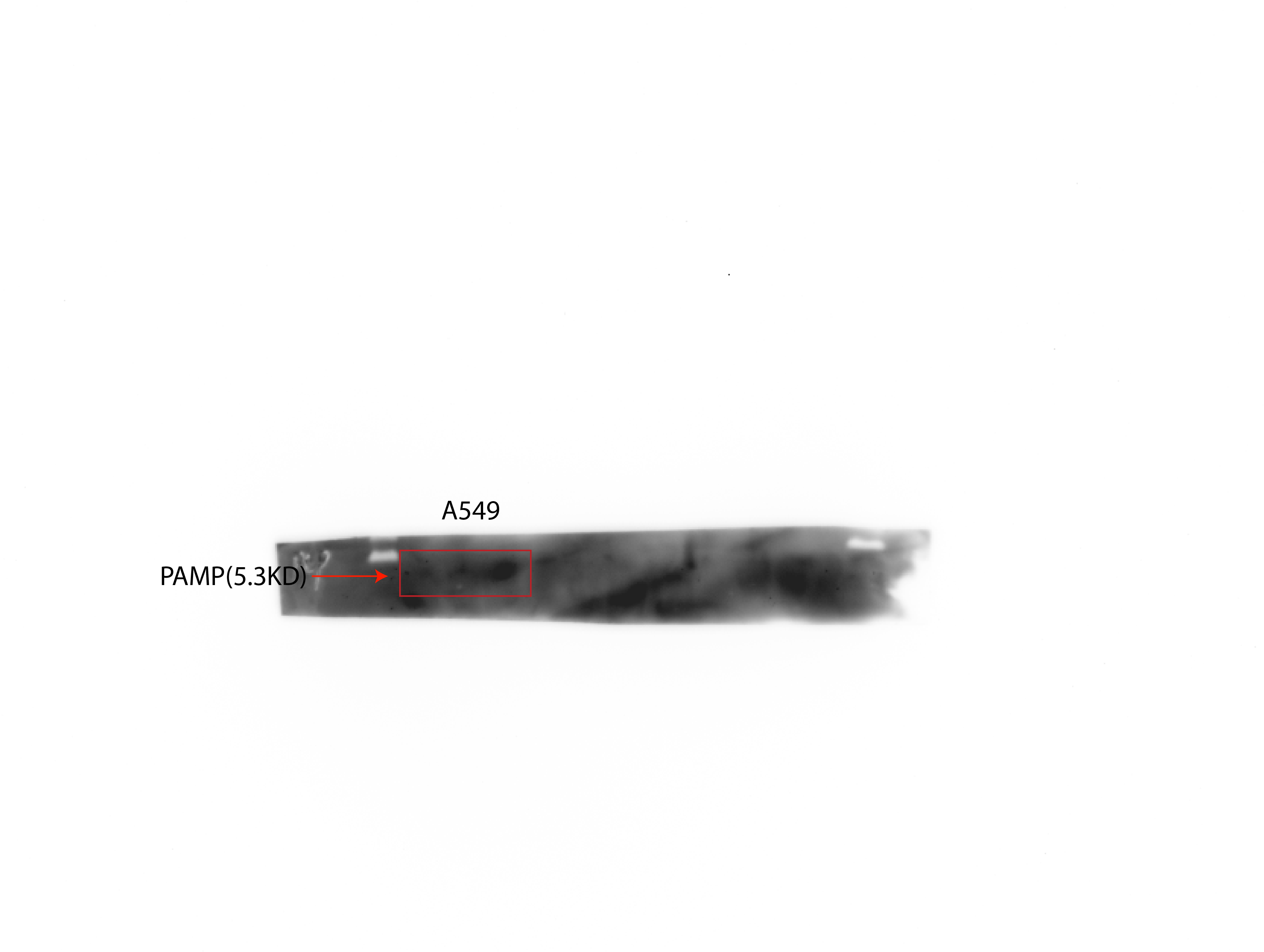

Supplement: Supplementary file 8 — Source data Fig. 5 [file 44321_2026_460_MOESM8_ESM.zip › Source data Figure5/FIG 5K/PAMP.png]

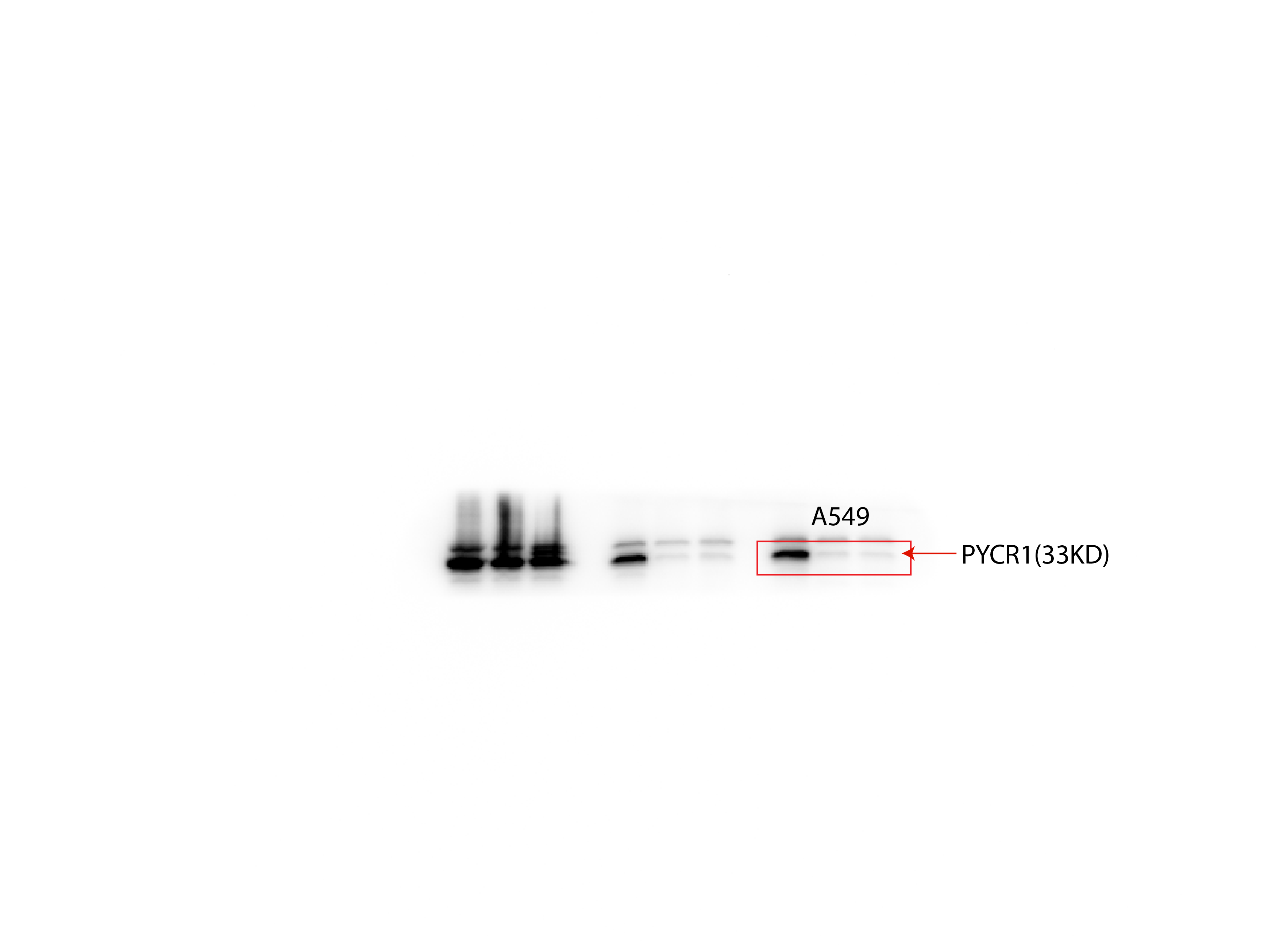

Supplement: Supplementary file 8 — Source data Fig. 5 [file 44321_2026_460_MOESM8_ESM.zip › Source data Figure5/FIG 5K/PYCR1.png]

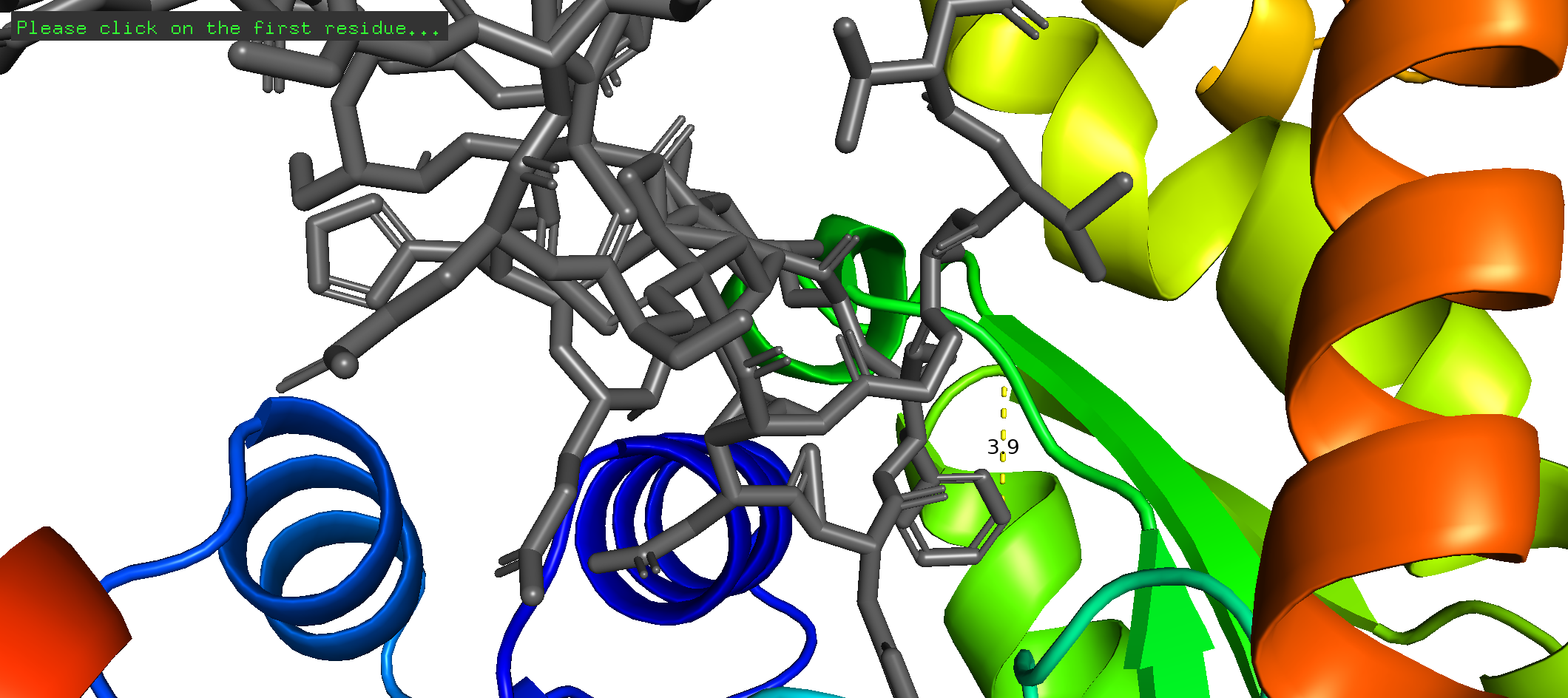

Supplement: Supplementary file 9 — Source data Fig. 6 [file 44321_2026_460_MOESM9_ESM.zip › Source data Figure6/FIG 6B/DOCK.png]

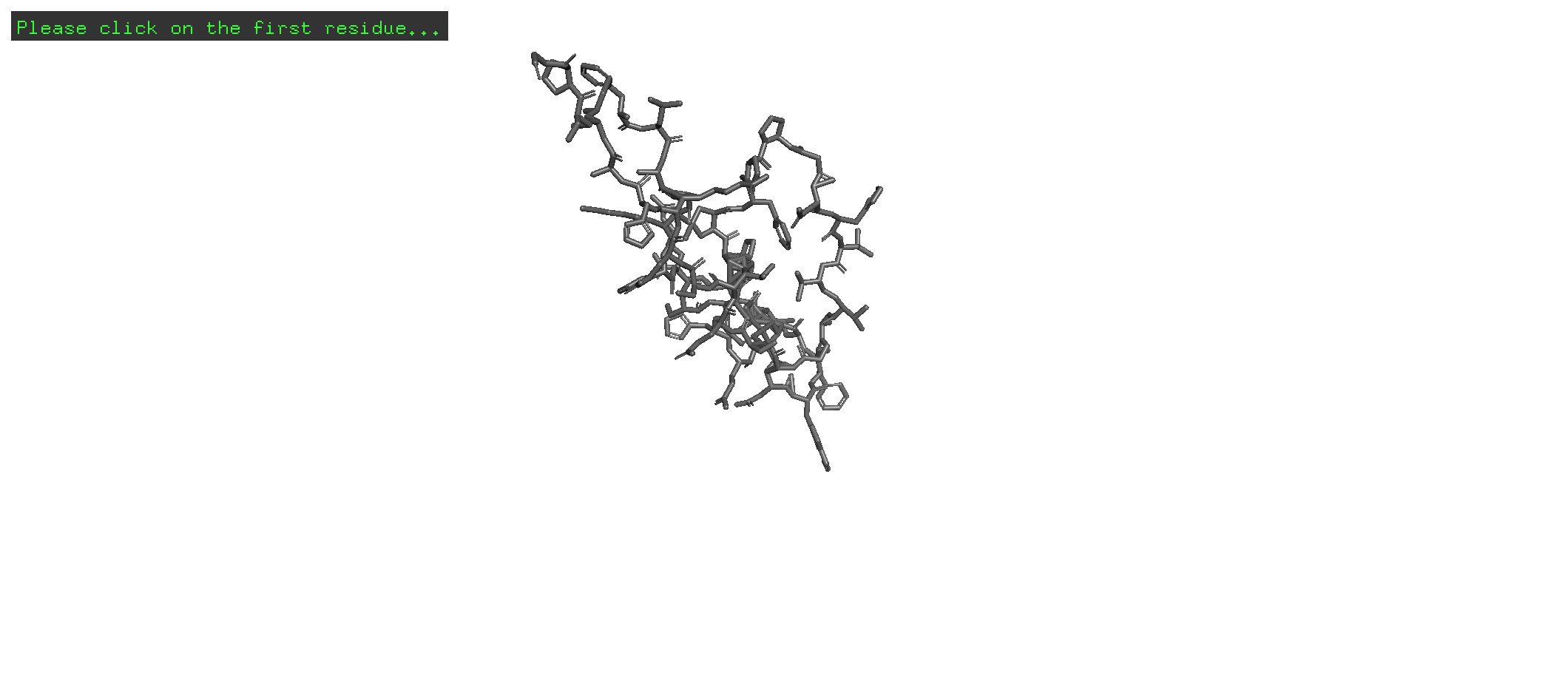

Supplement: Supplementary file 9 — Source data Fig. 6 [file 44321_2026_460_MOESM9_ESM.zip › Source data Figure6/FIG 6B/PAMP.png]

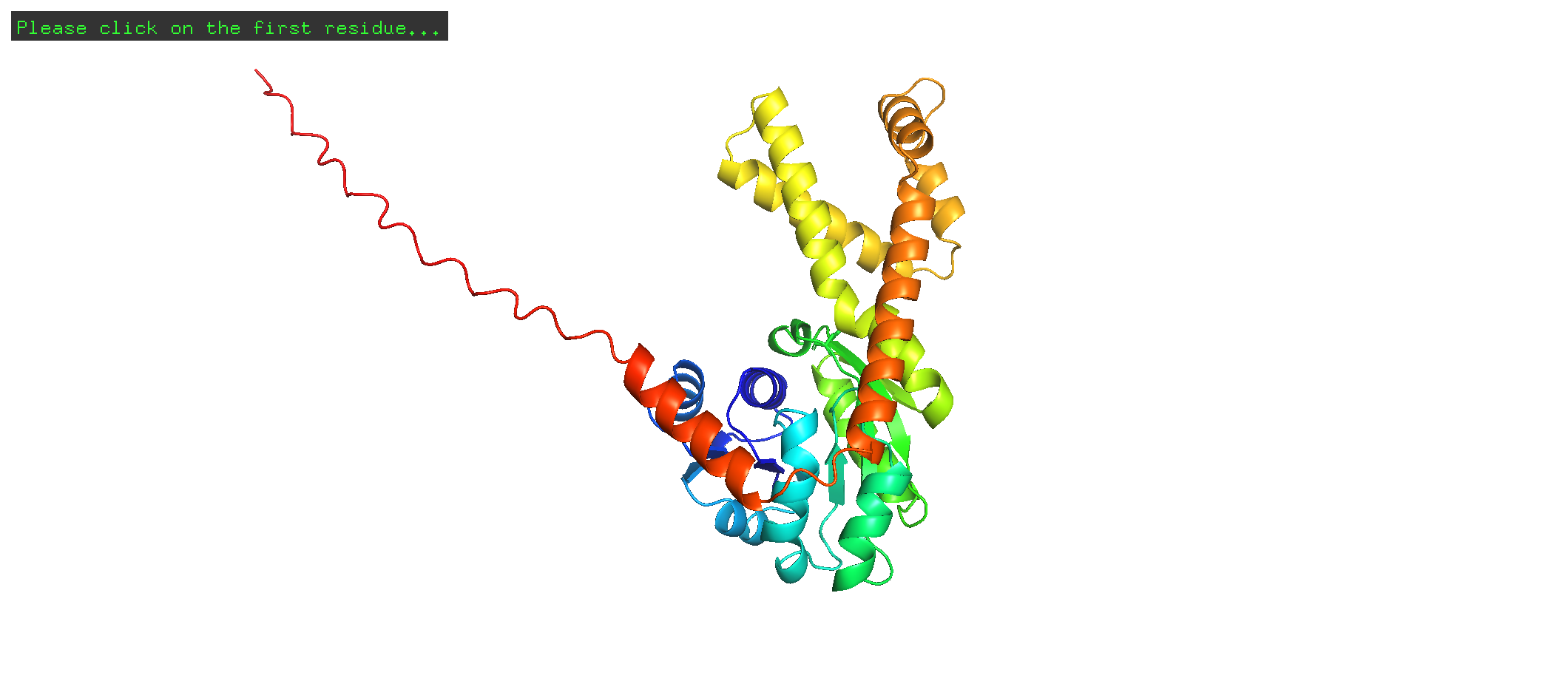

Supplement: Supplementary file 9 — Source data Fig. 6 [file 44321_2026_460_MOESM9_ESM.zip › Source data Figure6/FIG 6B/PYCR1.png]

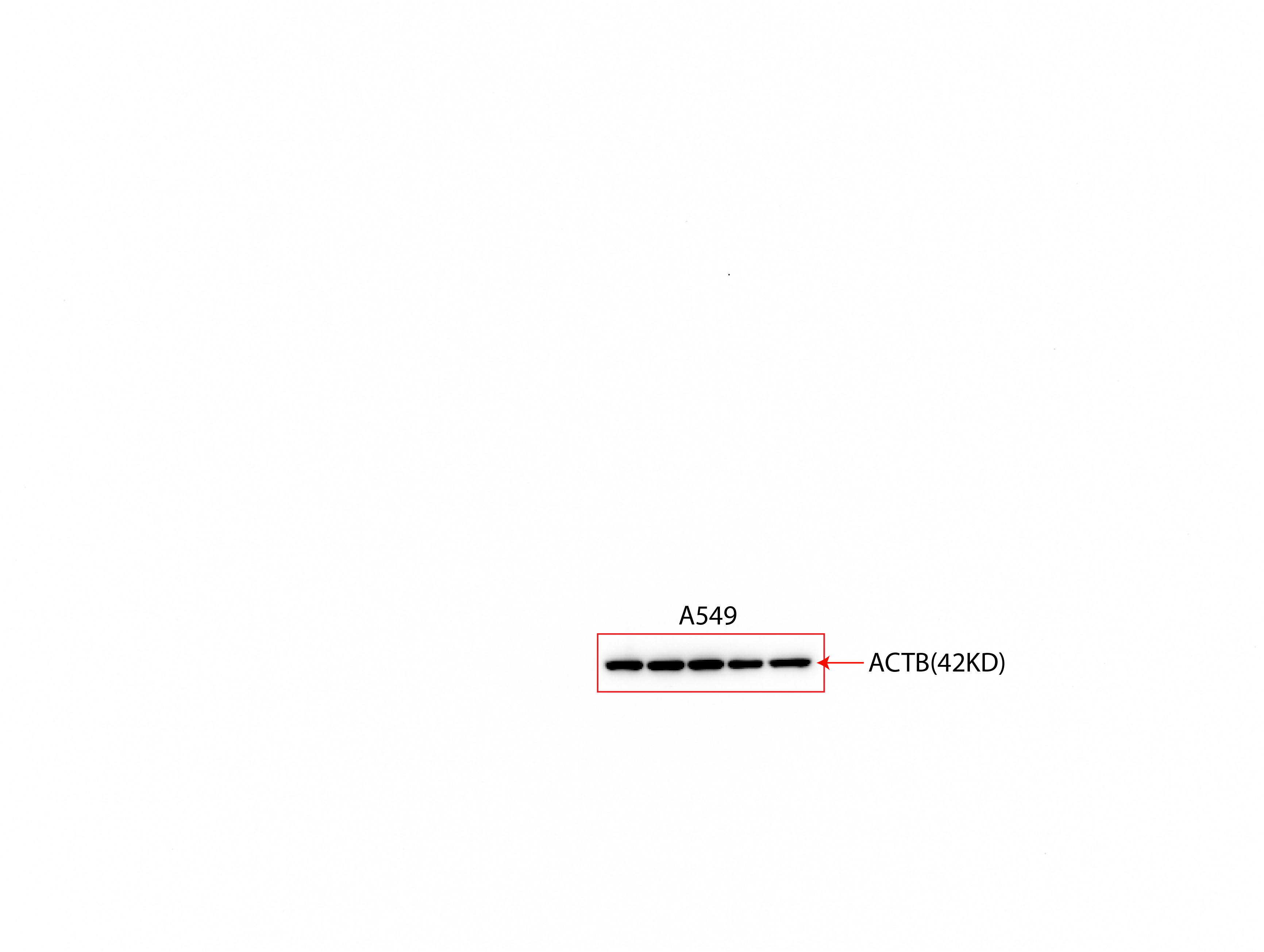

Supplement: Supplementary file 9 — Source data Fig. 6 [file 44321_2026_460_MOESM9_ESM.zip › Source data Figure6/FIG 6C/ACTB.png]

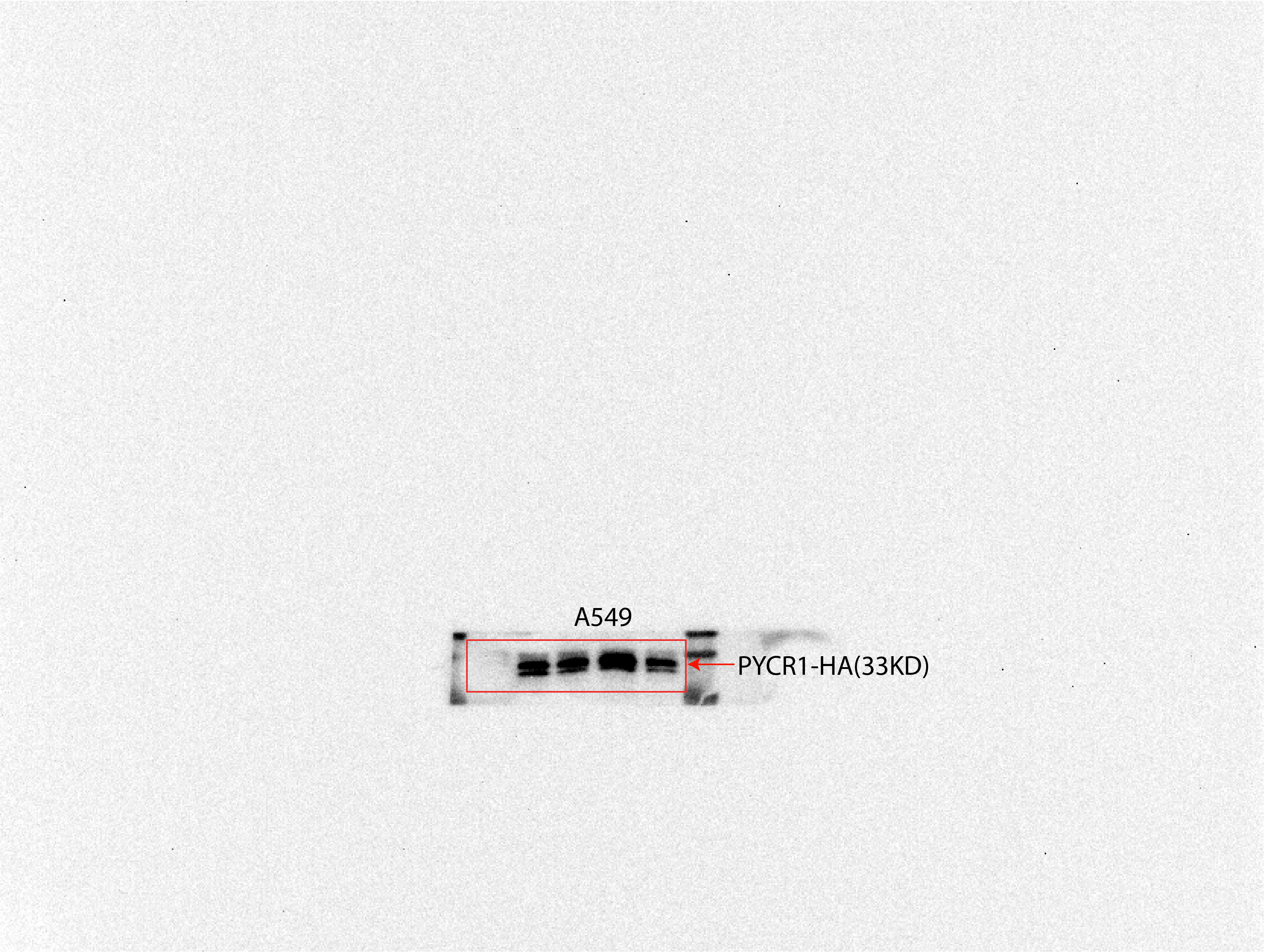

Supplement: Supplementary file 9 — Source data Fig. 6 [file 44321_2026_460_MOESM9_ESM.zip › Source data Figure6/FIG 6C/HA.png]

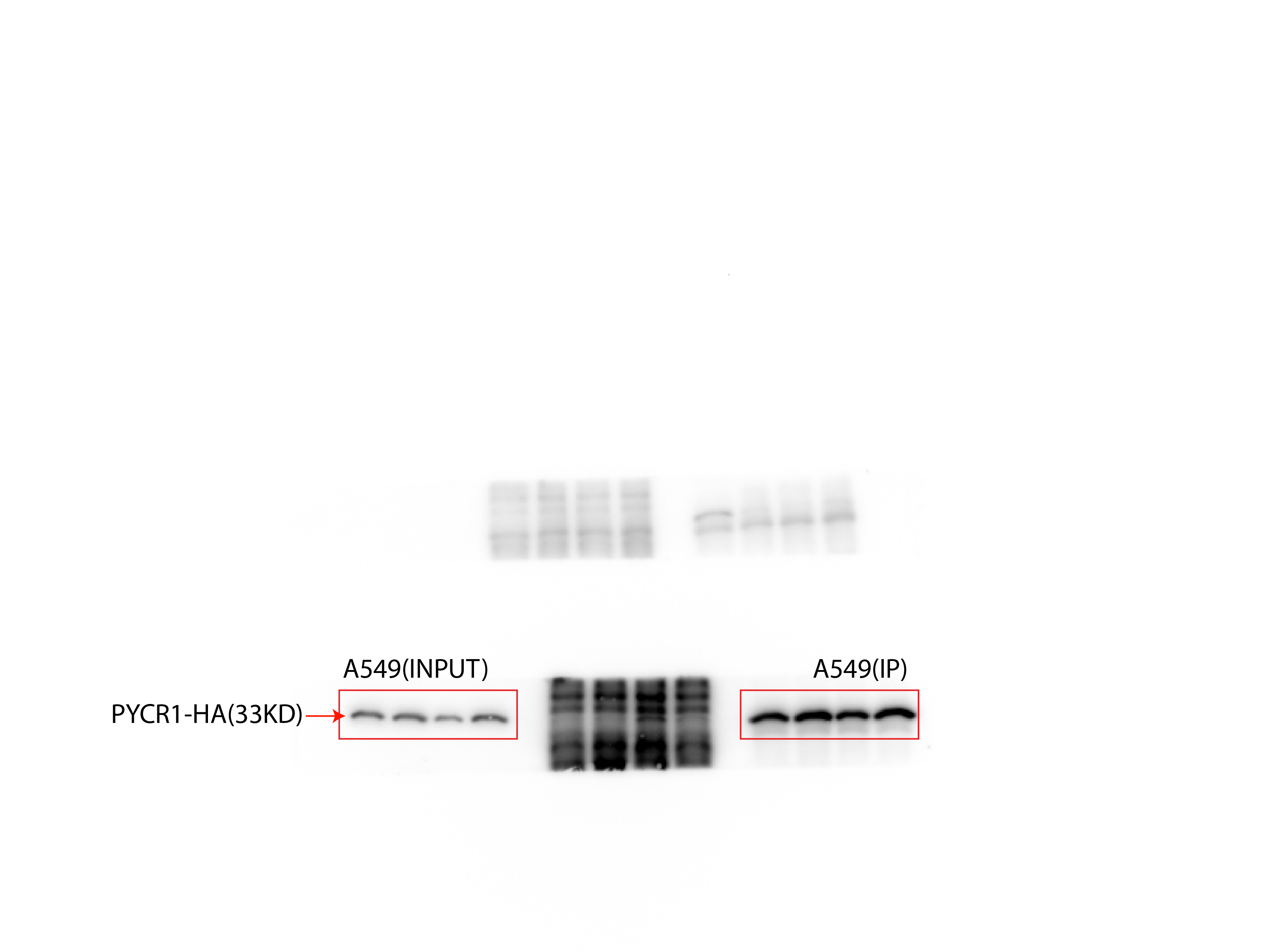

Supplement: Supplementary file 9 — Source data Fig. 6 [file 44321_2026_460_MOESM9_ESM.zip › Source data Figure6/FIG 6F/HA1+2.png]

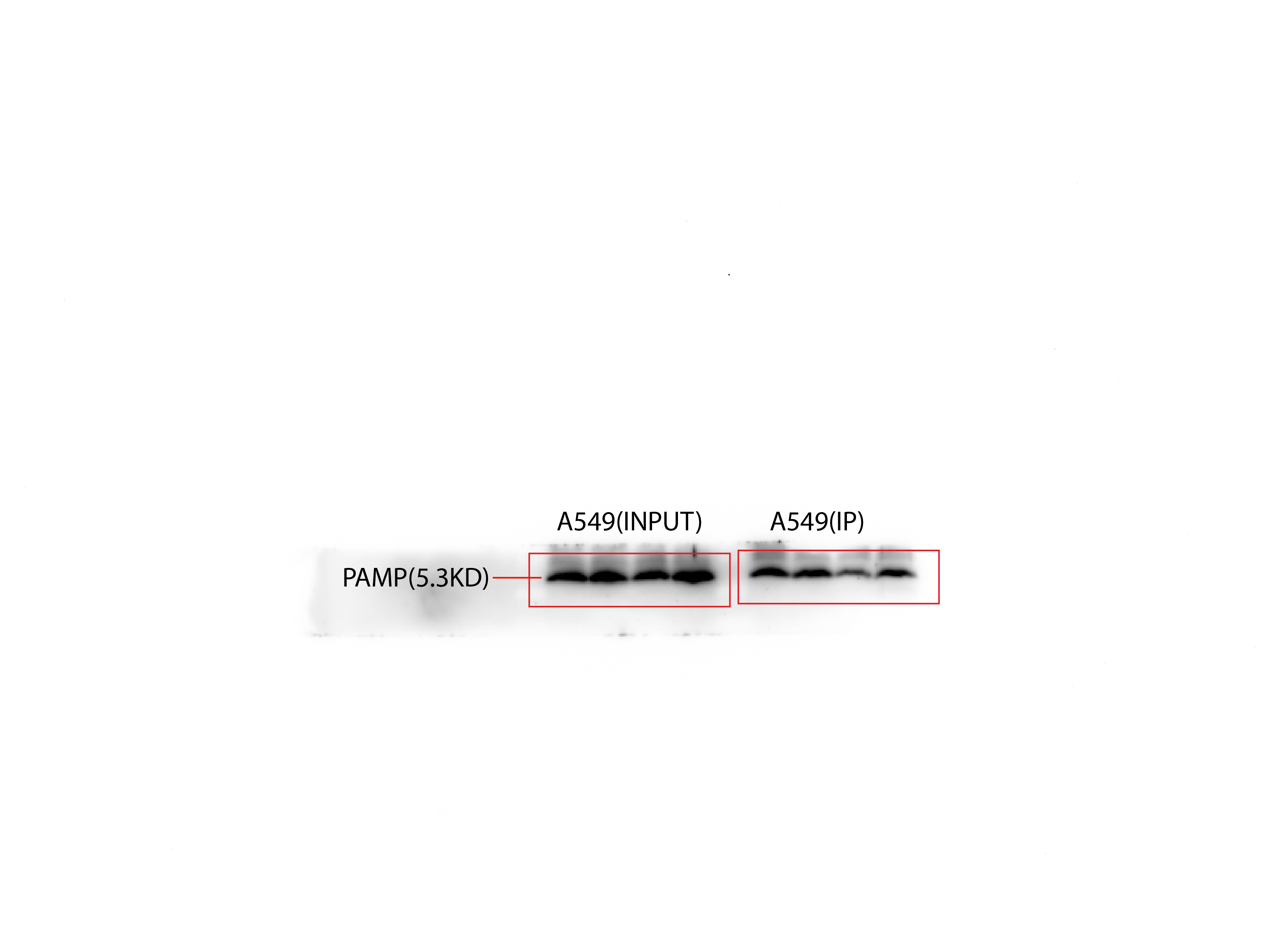

Supplement: Supplementary file 9 — Source data Fig. 6 [file 44321_2026_460_MOESM9_ESM.zip › Source data Figure6/FIG 6F/PAMP1+2.png]

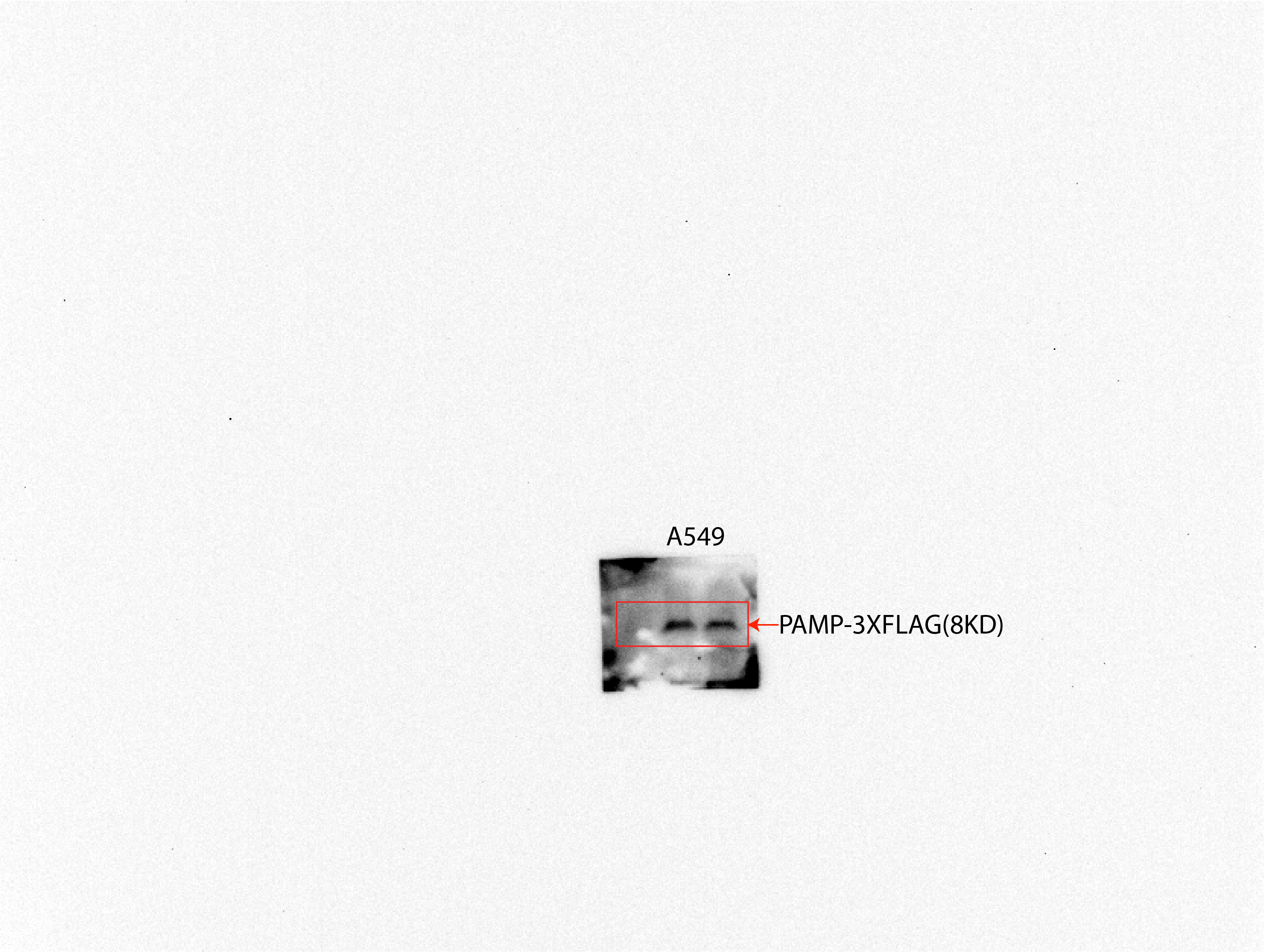

Supplement: Supplementary file 9 — Source data Fig. 6 [file 44321_2026_460_MOESM9_ESM.zip › Source data Figure6/FIG 6G/FLAG.png]

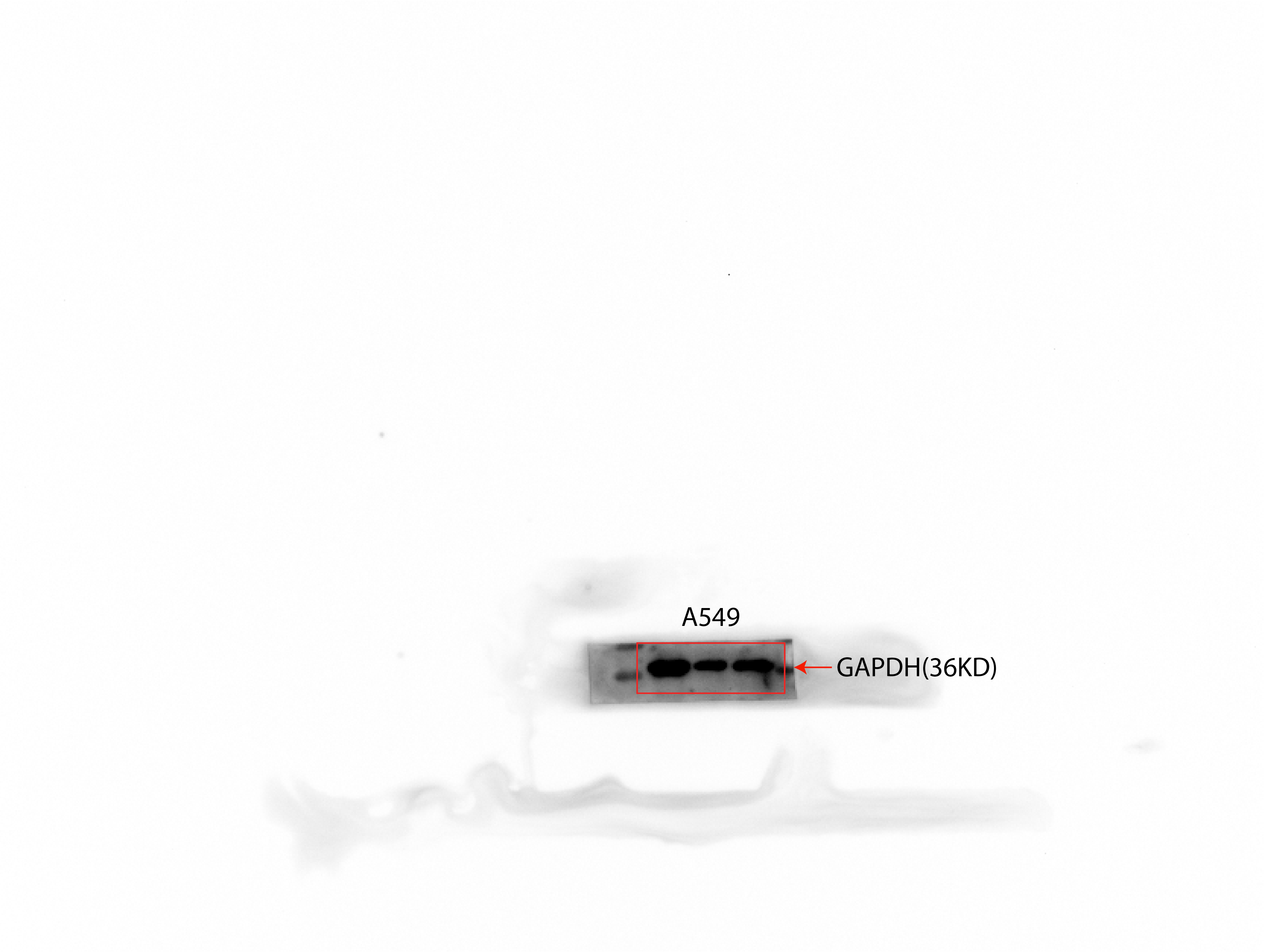

Supplement: Supplementary file 9 — Source data Fig. 6 [file 44321_2026_460_MOESM9_ESM.zip › Source data Figure6/FIG 6G/GAPDH.png]

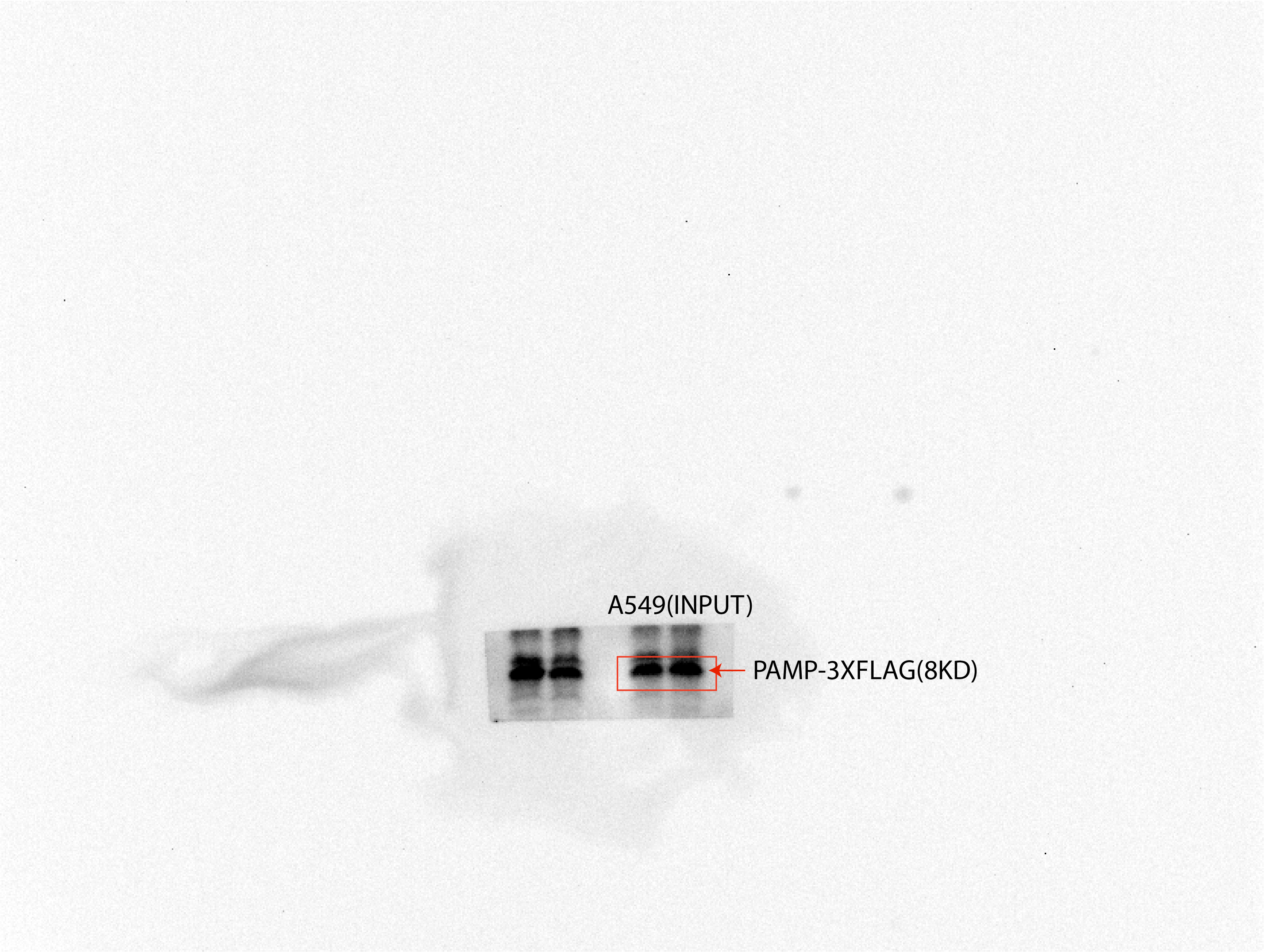

Supplement: Supplementary file 9 — Source data Fig. 6 [file 44321_2026_460_MOESM9_ESM.zip › Source data Figure6/FIG 6J/input-flag.png]

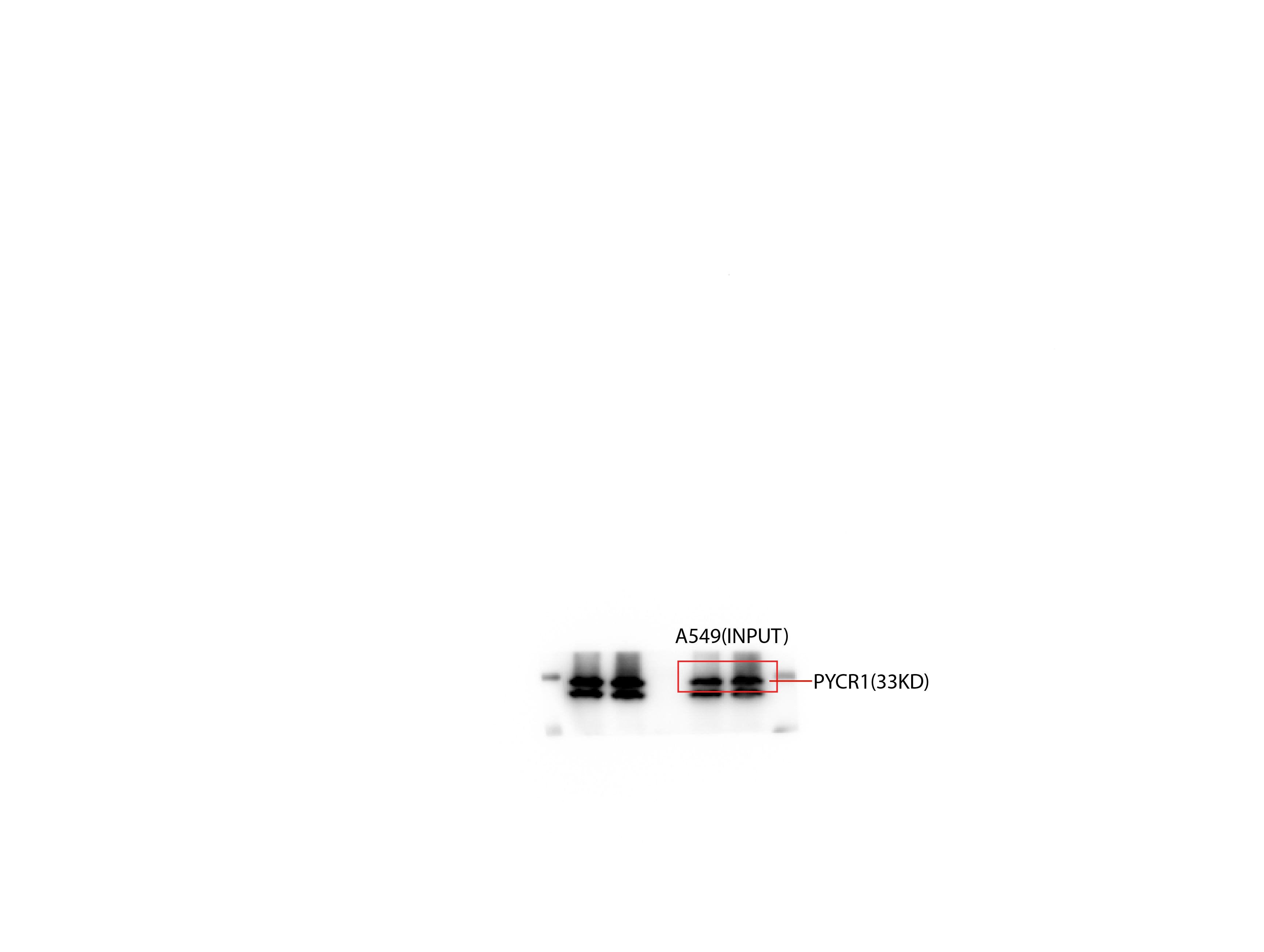

Supplement: Supplementary file 9 — Source data Fig. 6 [file 44321_2026_460_MOESM9_ESM.zip › Source data Figure6/FIG 6J/input-pycr1.png]

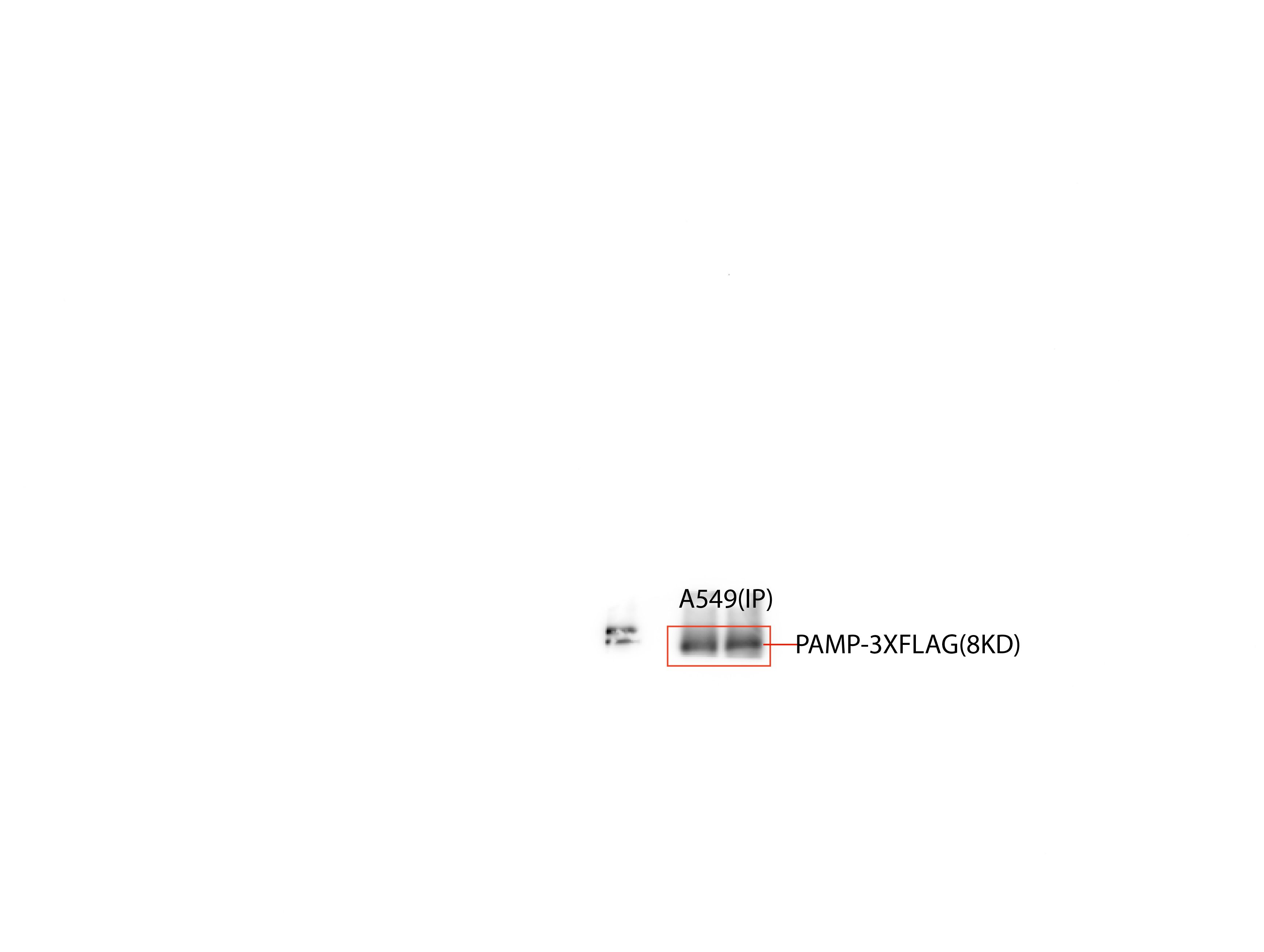

Supplement: Supplementary file 9 — Source data Fig. 6 [file 44321_2026_460_MOESM9_ESM.zip › Source data Figure6/FIG 6J/ip-flag.png]

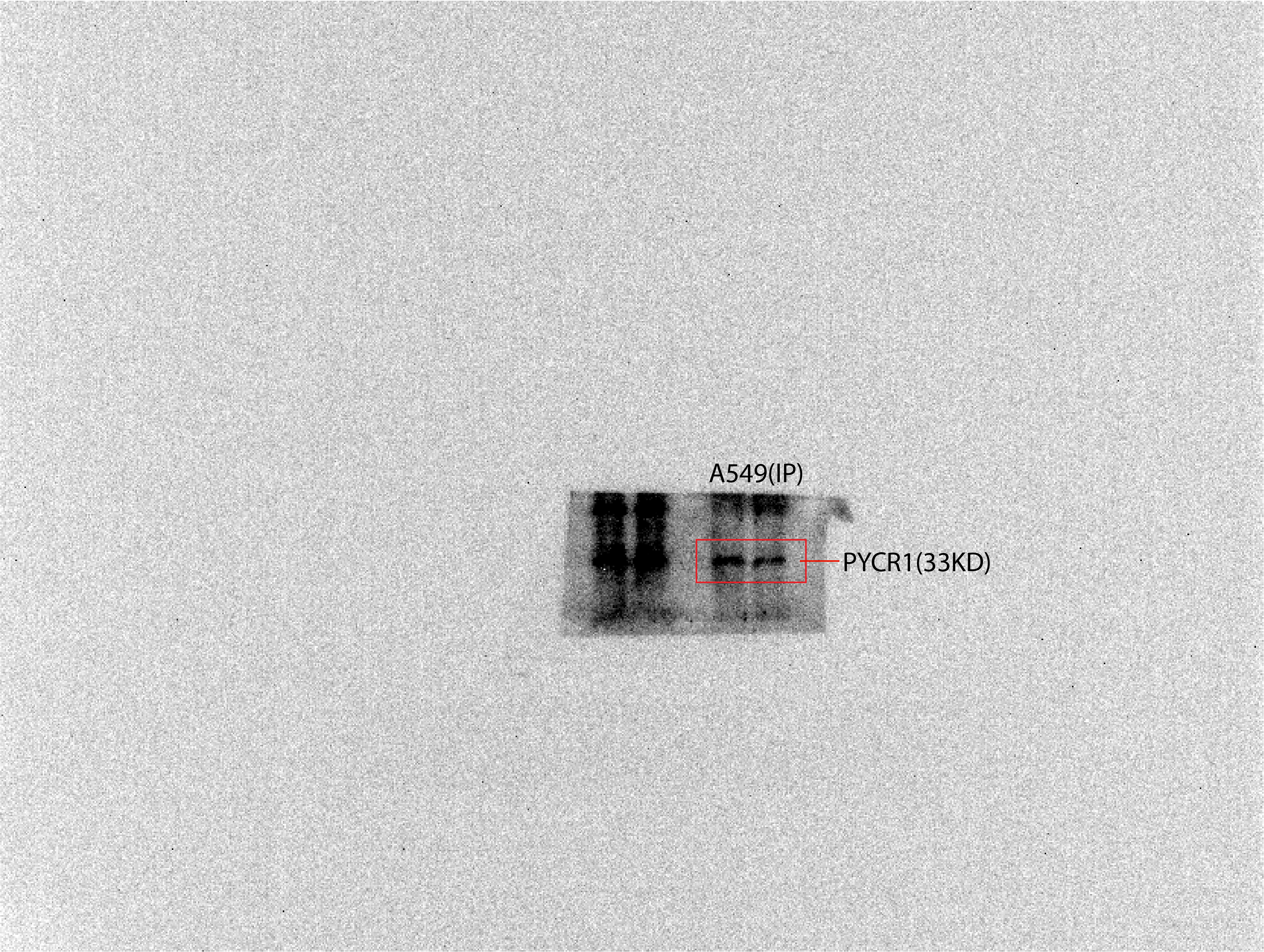

Supplement: Supplementary file 9 — Source data Fig. 6 [file 44321_2026_460_MOESM9_ESM.zip › Source data Figure6/FIG 6J/ip-pycr1.png]

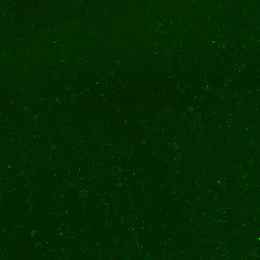

Supplement: Supplementary file 10 — Source data Fig. 7 [file 44321_2026_460_MOESM10_ESM.zip › Source data Figure7/FIG 7A/A549-0.png]

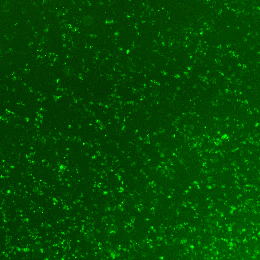

Supplement: Supplementary file 10 — Source data Fig. 7 [file 44321_2026_460_MOESM10_ESM.zip › Source data Figure7/FIG 7A/A549-100.png]

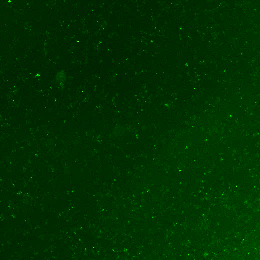

Supplement: Supplementary file 10 — Source data Fig. 7 [file 44321_2026_460_MOESM10_ESM.zip › Source data Figure7/FIG 7A/A549-12.5.png]

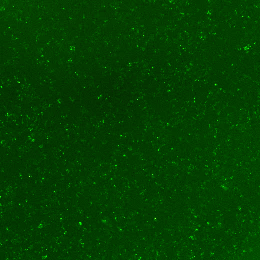

Supplement: Supplementary file 10 — Source data Fig. 7 [file 44321_2026_460_MOESM10_ESM.zip › Source data Figure7/FIG 7A/A549-25.png]

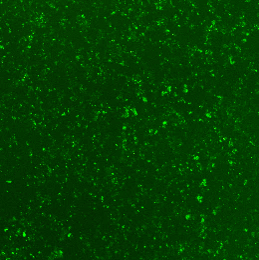

Supplement: Supplementary file 10 — Source data Fig. 7 [file 44321_2026_460_MOESM10_ESM.zip › Source data Figure7/FIG 7A/A549-50.png]

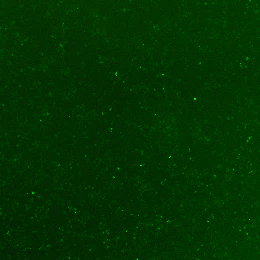

Supplement: Supplementary file 10 — Source data Fig. 7 [file 44321_2026_460_MOESM10_ESM.zip › Source data Figure7/FIG 7A/A549-6.25.png]

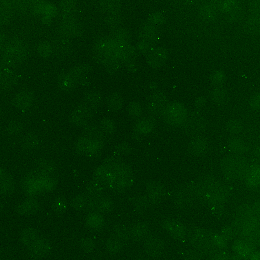

Supplement: Supplementary file 10 — Source data Fig. 7 [file 44321_2026_460_MOESM10_ESM.zip › Source data Figure7/FIG 7A/H460-0.png]

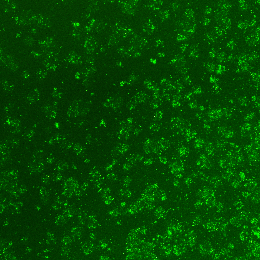

Supplement: Supplementary file 10 — Source data Fig. 7 [file 44321_2026_460_MOESM10_ESM.zip › Source data Figure7/FIG 7A/H460-100.png]

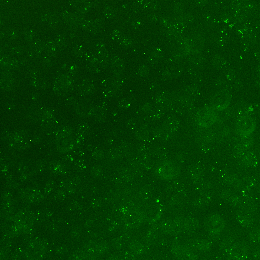

Supplement: Supplementary file 10 — Source data Fig. 7 [file 44321_2026_460_MOESM10_ESM.zip › Source data Figure7/FIG 7A/H460-12.5.png]

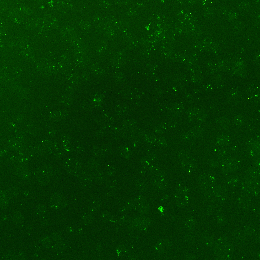

Supplement: Supplementary file 10 — Source data Fig. 7 [file 44321_2026_460_MOESM10_ESM.zip › Source data Figure7/FIG 7A/H460-25.png]

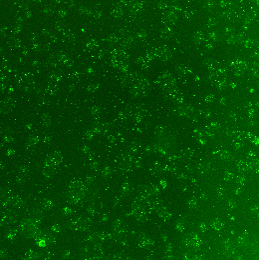

Supplement: Supplementary file 10 — Source data Fig. 7 [file 44321_2026_460_MOESM10_ESM.zip › Source data Figure7/FIG 7A/H460-50.png]

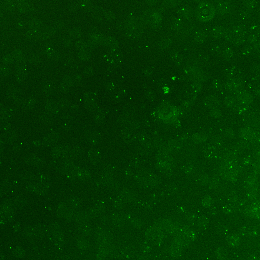

Supplement: Supplementary file 10 — Source data Fig. 7 [file 44321_2026_460_MOESM10_ESM.zip › Source data Figure7/FIG 7A/H460-6.25.png]

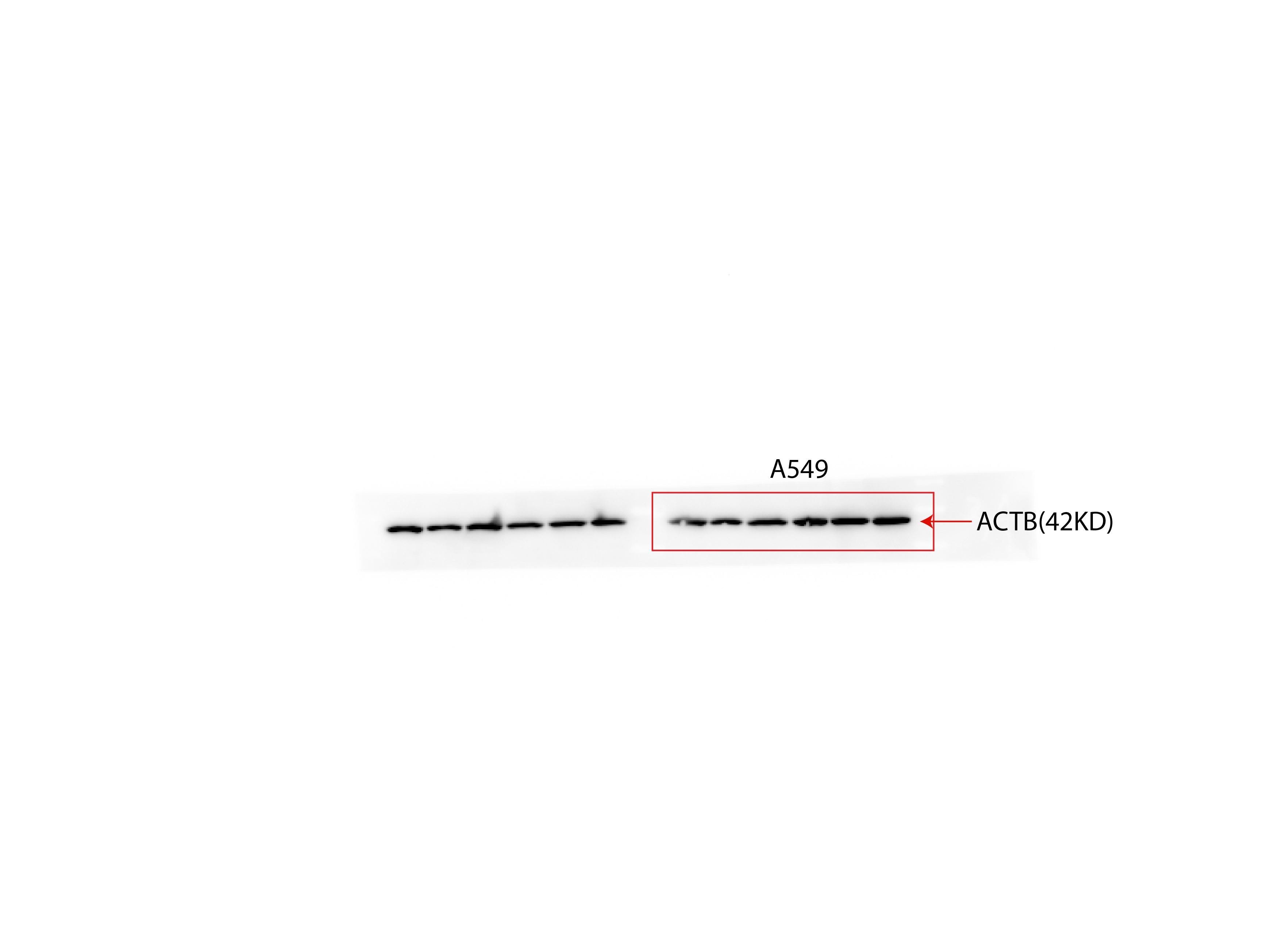

Supplement: Supplementary file 10 — Source data Fig. 7 [file 44321_2026_460_MOESM10_ESM.zip › Source data Figure7/FIG 7B/A549-ACTB.png]

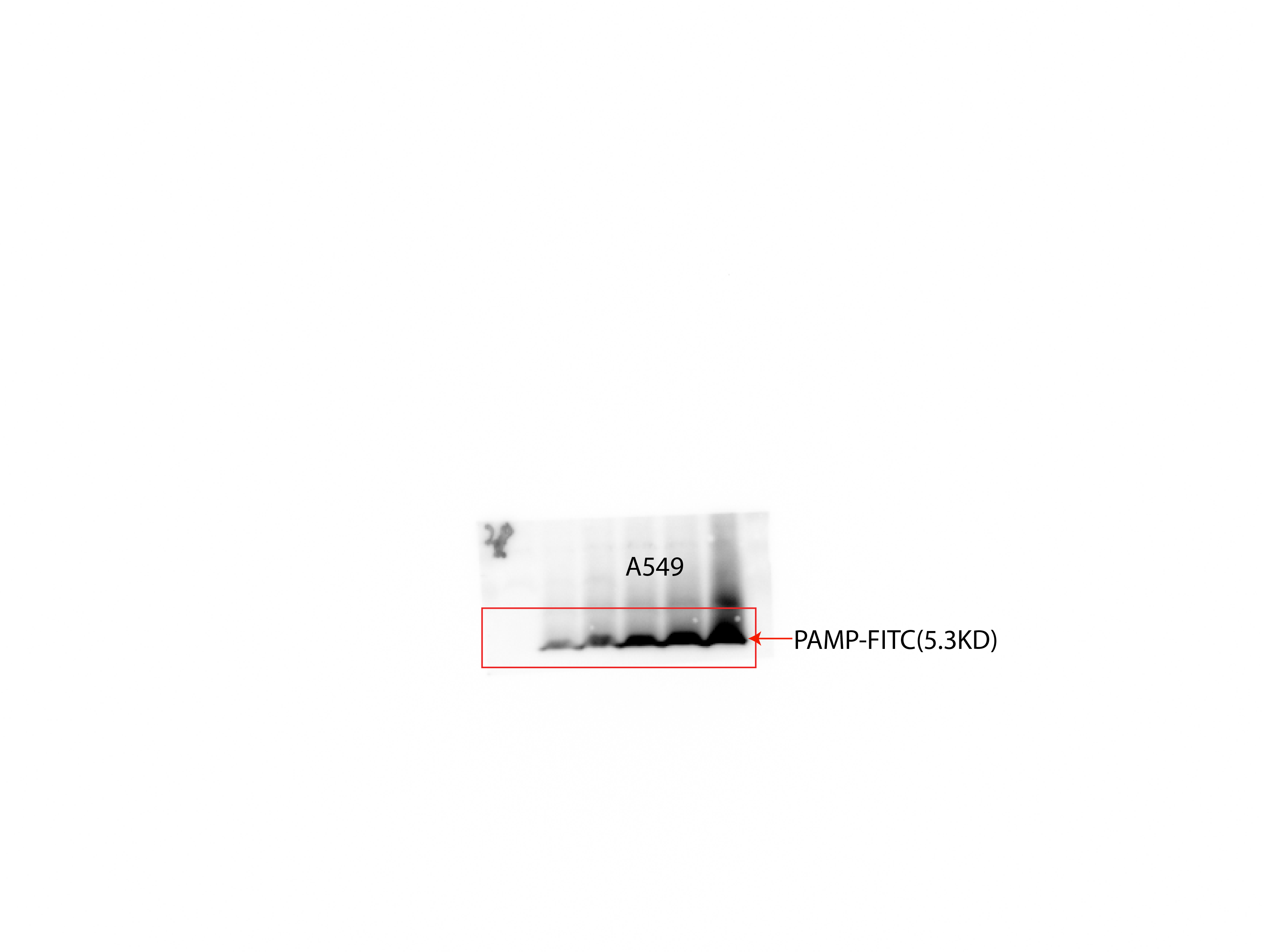

Supplement: Supplementary file 10 — Source data Fig. 7 [file 44321_2026_460_MOESM10_ESM.zip › Source data Figure7/FIG 7B/A549-FITC.png]

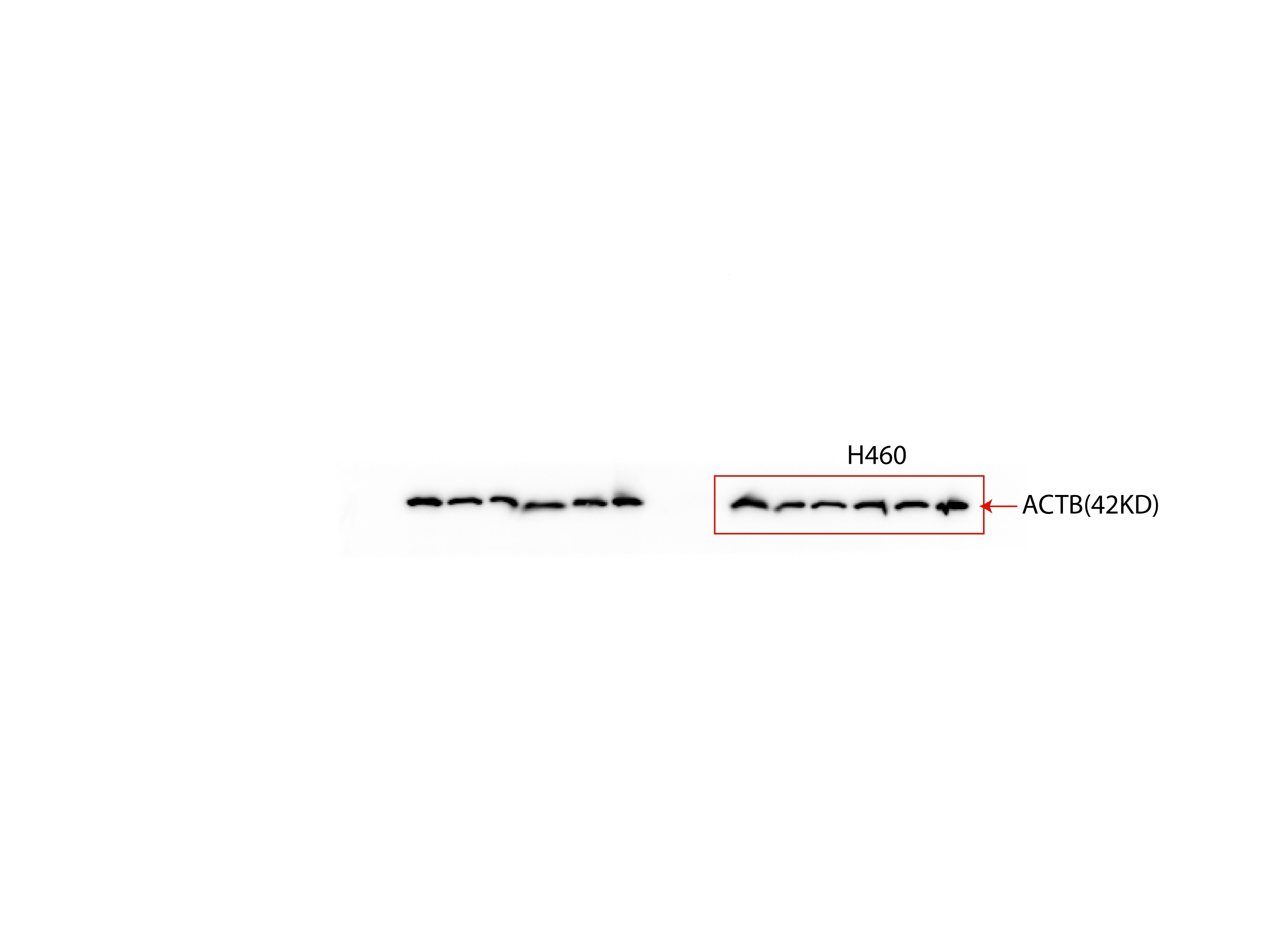

Supplement: Supplementary file 10 — Source data Fig. 7 [file 44321_2026_460_MOESM10_ESM.zip › Source data Figure7/FIG 7B/H460-ACTB.png]

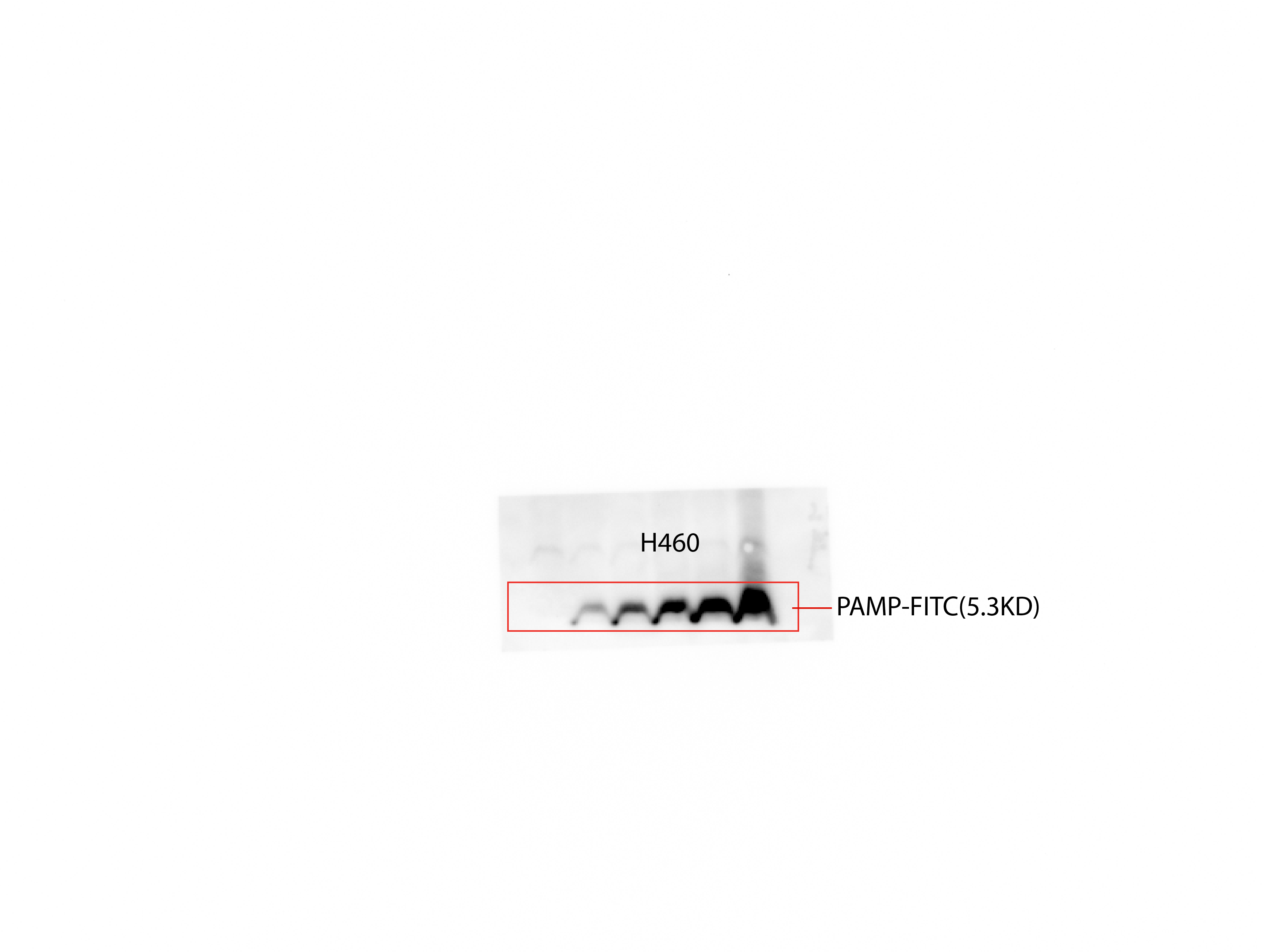

Supplement: Supplementary file 10 — Source data Fig. 7 [file 44321_2026_460_MOESM10_ESM.zip › Source data Figure7/FIG 7B/H460-FITC.png]

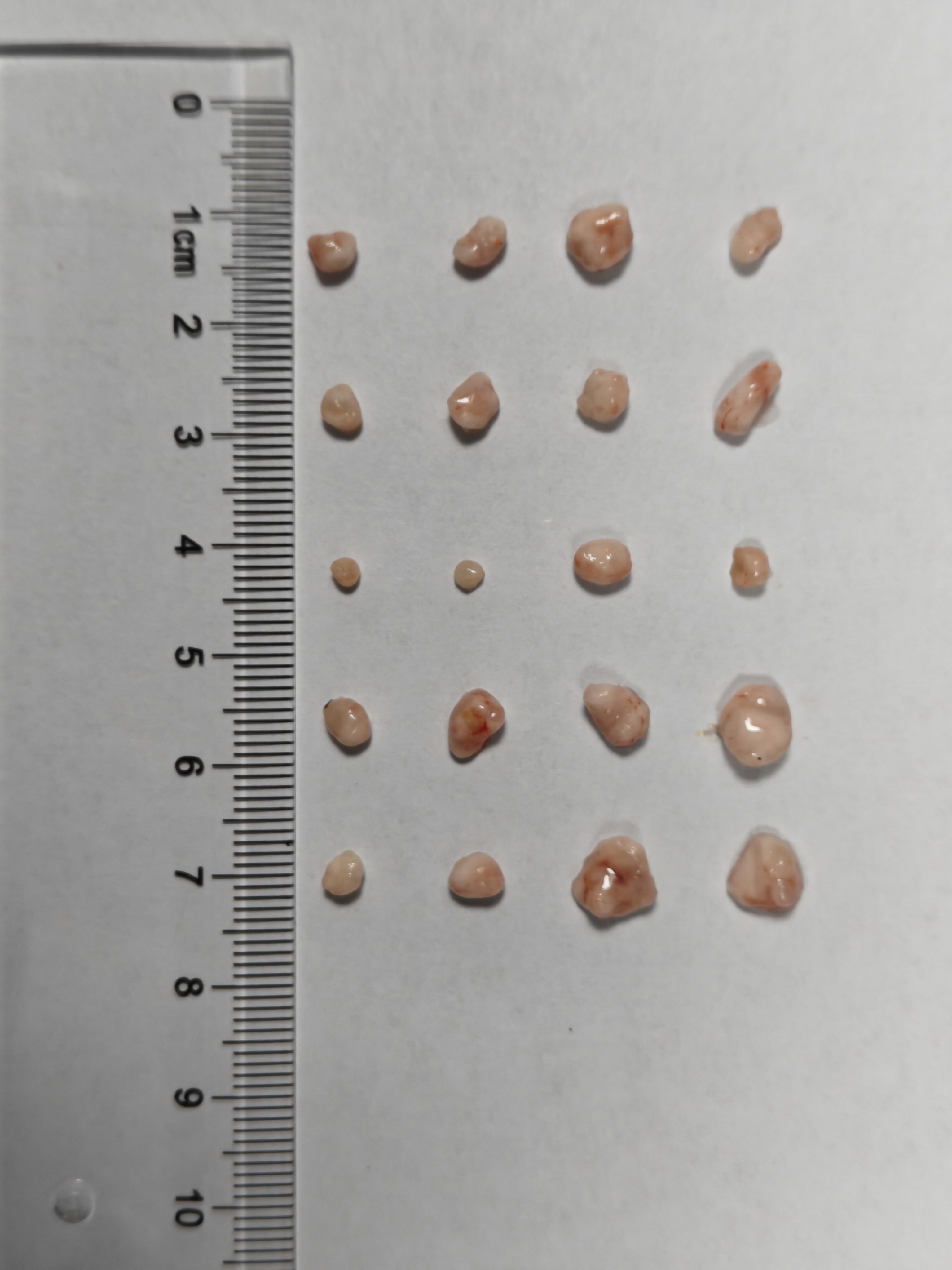

Supplement: Supplementary file 10 — Source data Fig. 7 [file 44321_2026_460_MOESM10_ESM.zip › Source data Figure7/FIG 7I.jpg]

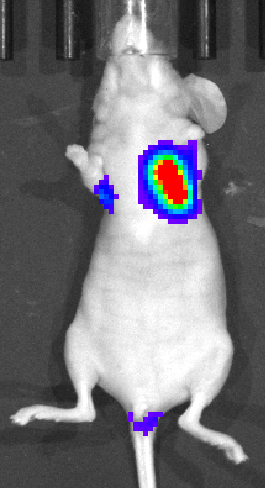

Supplement: Supplementary file 10 — Source data Fig. 7 [file 44321_2026_460_MOESM10_ESM.zip › Source data Figure7/FIG 7J/Control1.png]

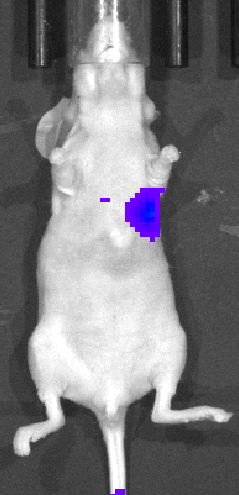

Supplement: Supplementary file 10 — Source data Fig. 7 [file 44321_2026_460_MOESM10_ESM.zip › Source data Figure7/FIG 7J/Control2.png]

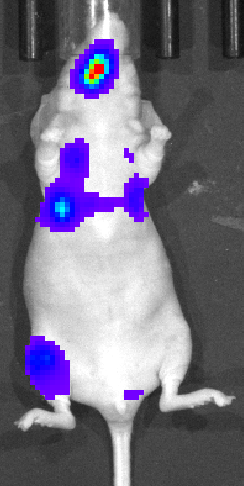

Supplement: Supplementary file 10 — Source data Fig. 7 [file 44321_2026_460_MOESM10_ESM.zip › Source data Figure7/FIG 7J/Control3.png]
