# Supplementary figures and images for: PAMP orchestrates proline metabolic rewiring to suppress LUAD via PYCR1 inhibition (part 2 of 2)
Source: EMBO Mol Med. 2026 Jun 9;18(7):2867–95. doi: 10.1038/s44321-026-00460-2 (PMC13365495; doi:10.1038/s44321-026-00460-2)

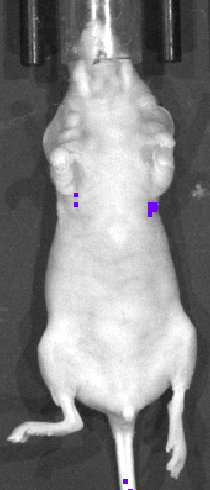

Supplement: Supplementary file 10 — Source data Fig. 7 [file 44321_2026_460_MOESM10_ESM.zip › Source data Figure7/FIG 7J/PAMP1.png]

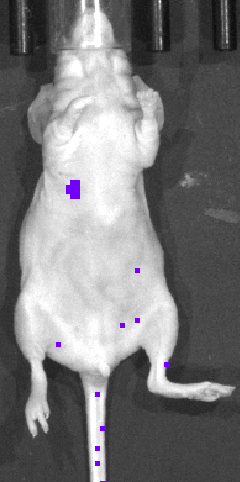

Supplement: Supplementary file 10 — Source data Fig. 7 [file 44321_2026_460_MOESM10_ESM.zip › Source data Figure7/FIG 7J/PAMP2.png]

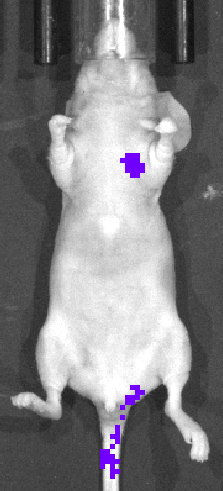

Supplement: Supplementary file 10 — Source data Fig. 7 [file 44321_2026_460_MOESM10_ESM.zip › Source data Figure7/FIG 7J/PAMP3.png]

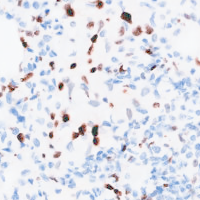

Supplement: Supplementary file 10 — Source data Fig. 7 [file 44321_2026_460_MOESM10_ESM.zip › Source data Figure7/FIG 7K/KI67-Control.png]

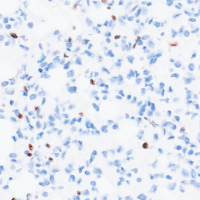

Supplement: Supplementary file 10 — Source data Fig. 7 [file 44321_2026_460_MOESM10_ESM.zip › Source data Figure7/FIG 7K/KI67-PAMP.png]

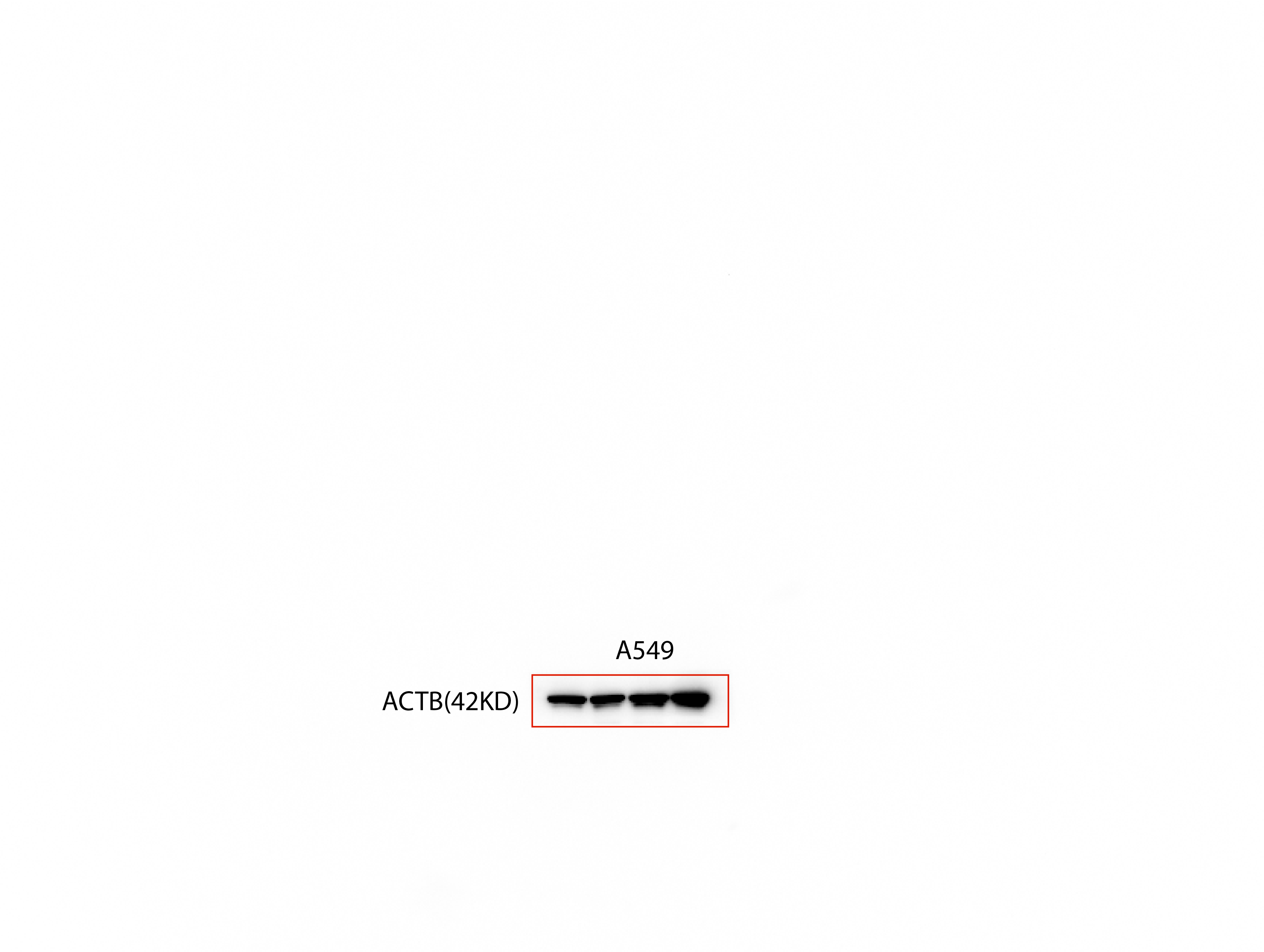

Supplement: Supplementary file 12 — Figure EV2 Source Data [file 44321_2026_460_MOESM12_ESM.zip › Source data Figure EV2/FIG EV2B/ACTB.png]

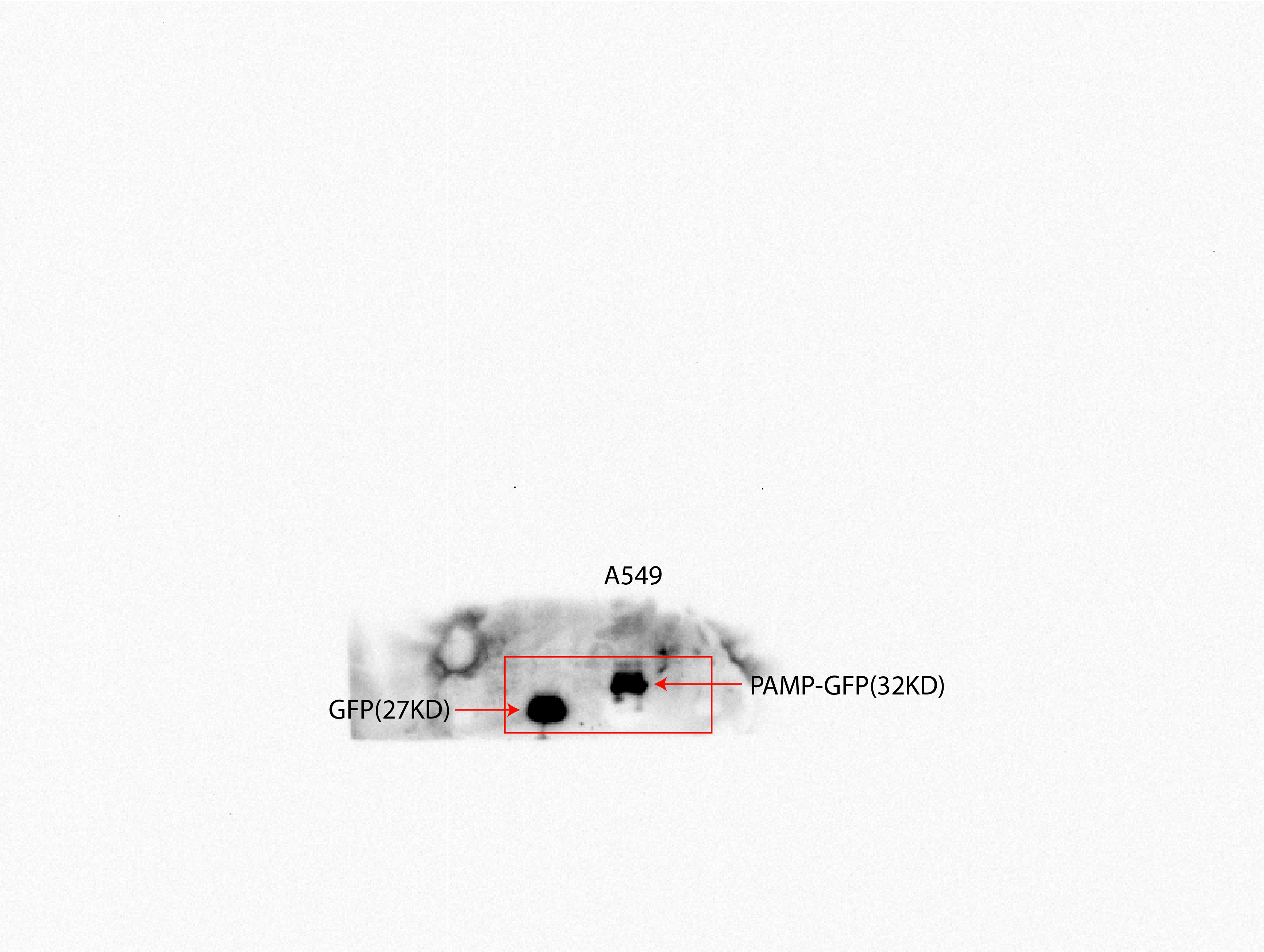

Supplement: Supplementary file 12 — Figure EV2 Source Data [file 44321_2026_460_MOESM12_ESM.zip › Source data Figure EV2/FIG EV2B/GFP.png]

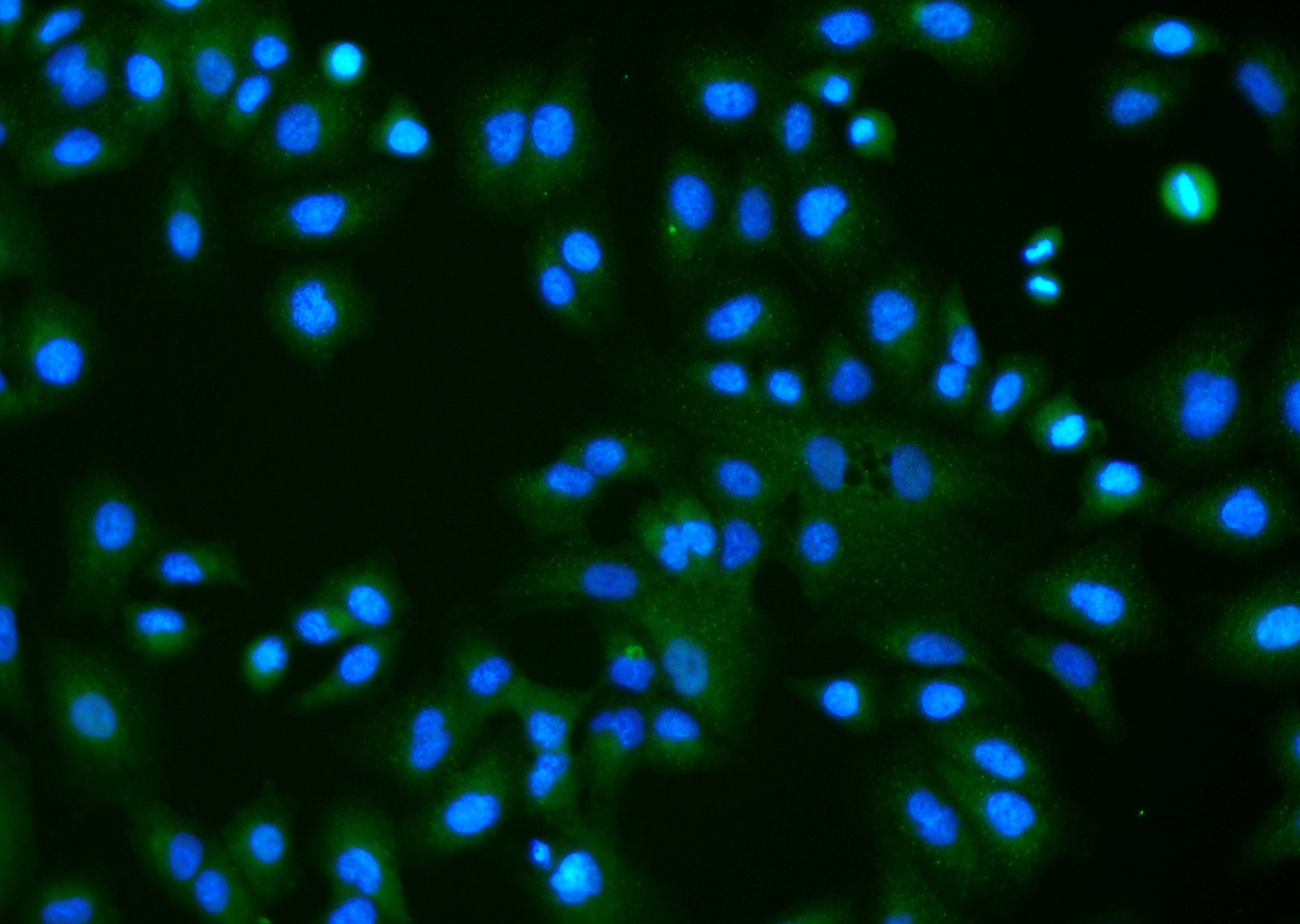

Supplement: Supplementary file 12 — Figure EV2 Source Data [file 44321_2026_460_MOESM12_ESM.zip › Source data Figure EV2/FIG EV2D/KO PAMP.tif]

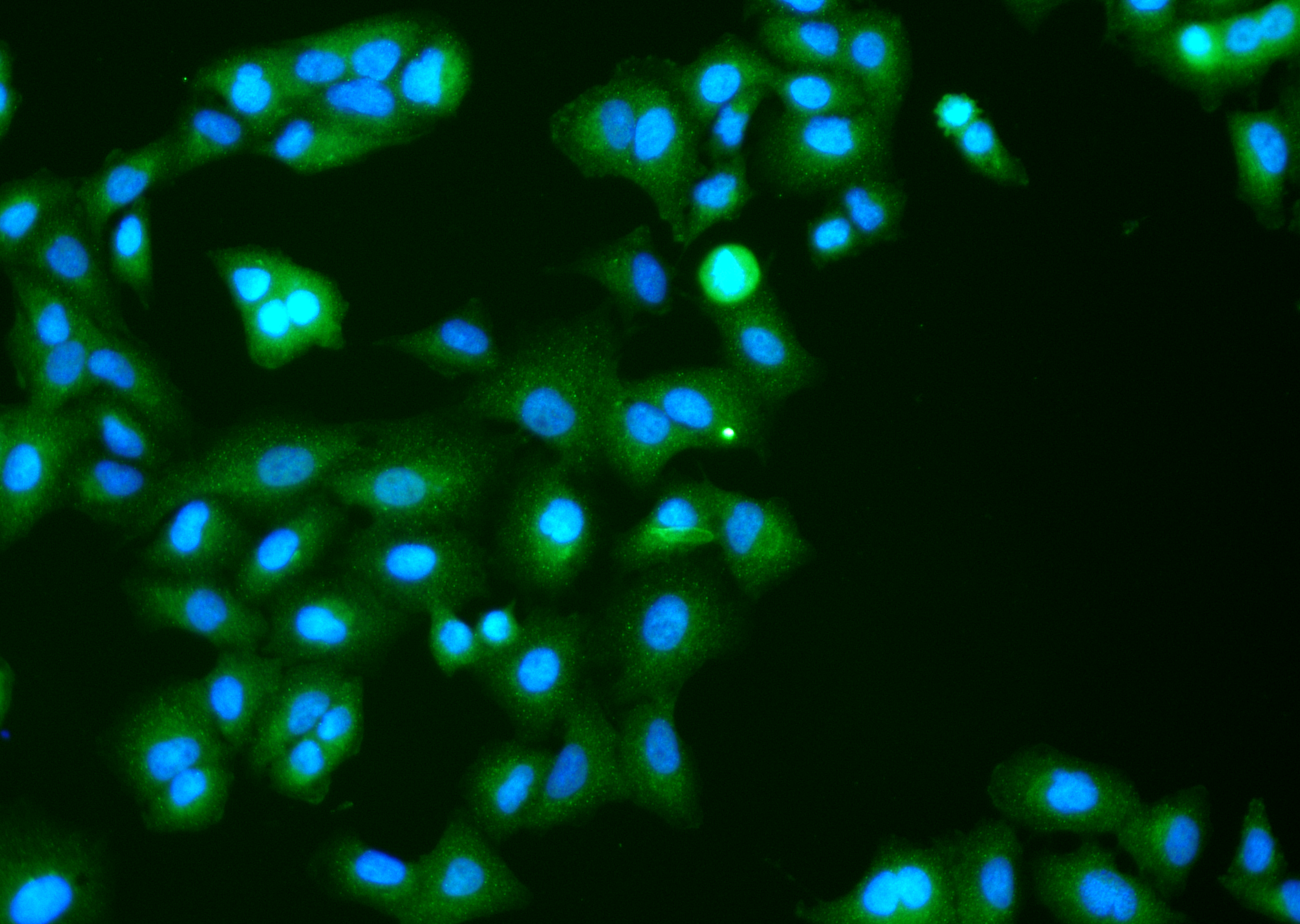

Supplement: Supplementary file 12 — Figure EV2 Source Data [file 44321_2026_460_MOESM12_ESM.zip › Source data Figure EV2/FIG EV2D/lacZ.tif]

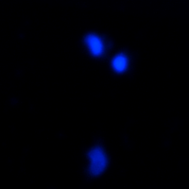

Supplement: Supplementary file 12 — Figure EV2 Source Data [file 44321_2026_460_MOESM12_ESM.zip › Source data Figure EV2/FIG EV2E/EV-Dapi.png]

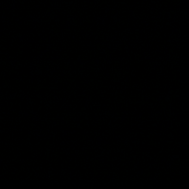

Supplement: Supplementary file 12 — Figure EV2 Source Data [file 44321_2026_460_MOESM12_ESM.zip › Source data Figure EV2/FIG EV2E/EV-GFP.png]

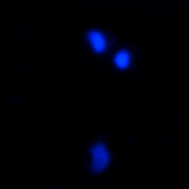

Supplement: Supplementary file 12 — Figure EV2 Source Data [file 44321_2026_460_MOESM12_ESM.zip › Source data Figure EV2/FIG EV2E/EV-Merge.png]

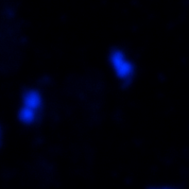

Supplement: Supplementary file 12 — Figure EV2 Source Data [file 44321_2026_460_MOESM12_ESM.zip › Source data Figure EV2/FIG EV2E/ORF-GFPmut-Dapi.png]

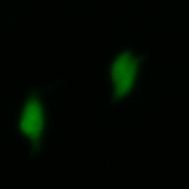

Supplement: Supplementary file 12 — Figure EV2 Source Data [file 44321_2026_460_MOESM12_ESM.zip › Source data Figure EV2/FIG EV2E/ORF-GFPmut-GFP.png]

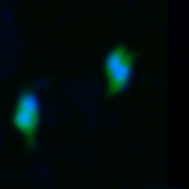

Supplement: Supplementary file 12 — Figure EV2 Source Data [file 44321_2026_460_MOESM12_ESM.zip › Source data Figure EV2/FIG EV2E/ORF-GFPmut-Merge.png]

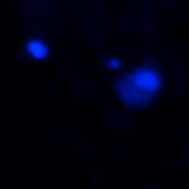

Supplement: Supplementary file 12 — Figure EV2 Source Data [file 44321_2026_460_MOESM12_ESM.zip › Source data Figure EV2/FIG EV2E/ORFmut-GFPmut-Dapi.png]

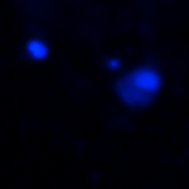

Supplement: Supplementary file 12 — Figure EV2 Source Data [file 44321_2026_460_MOESM12_ESM.zip › Source data Figure EV2/FIG EV2E/ORFmut-GFPmut-Merge.png]

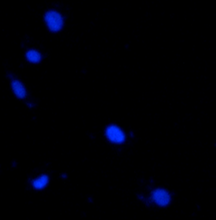

Supplement: Supplementary file 12 — Figure EV2 Source Data [file 44321_2026_460_MOESM12_ESM.zip › Source data Figure EV2/FIG EV2F/EV-Dapi.png]

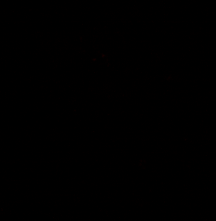

Supplement: Supplementary file 12 — Figure EV2 Source Data [file 44321_2026_460_MOESM12_ESM.zip › Source data Figure EV2/FIG EV2F/EV-FLAG.png]

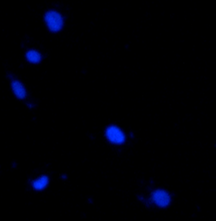

Supplement: Supplementary file 12 — Figure EV2 Source Data [file 44321_2026_460_MOESM12_ESM.zip › Source data Figure EV2/FIG EV2F/EV-Merge.png]

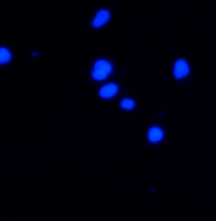

Supplement: Supplementary file 12 — Figure EV2 Source Data [file 44321_2026_460_MOESM12_ESM.zip › Source data Figure EV2/FIG EV2F/ORF-FLAG-Dapi.png]

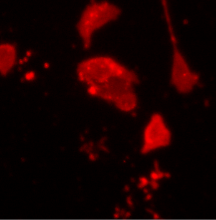

Supplement: Supplementary file 12 — Figure EV2 Source Data [file 44321_2026_460_MOESM12_ESM.zip › Source data Figure EV2/FIG EV2F/ORF-FLAG-FLAG.png]

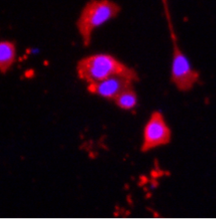

Supplement: Supplementary file 12 — Figure EV2 Source Data [file 44321_2026_460_MOESM12_ESM.zip › Source data Figure EV2/FIG EV2F/ORF-FLAG-Merge.png]

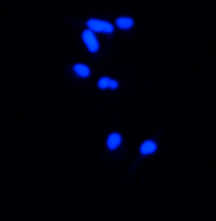

Supplement: Supplementary file 12 — Figure EV2 Source Data [file 44321_2026_460_MOESM12_ESM.zip › Source data Figure EV2/FIG EV2F/ORFmut-FLAG-Dapi.png]

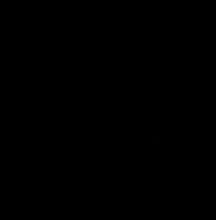

Supplement: Supplementary file 12 — Figure EV2 Source Data [file 44321_2026_460_MOESM12_ESM.zip › Source data Figure EV2/FIG EV2F/ORFmut-FLAG-FLAG.png]

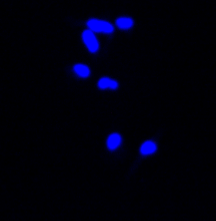

Supplement: Supplementary file 12 — Figure EV2 Source Data [file 44321_2026_460_MOESM12_ESM.zip › Source data Figure EV2/FIG EV2F/ORFmut-FLAG-Merge.png]

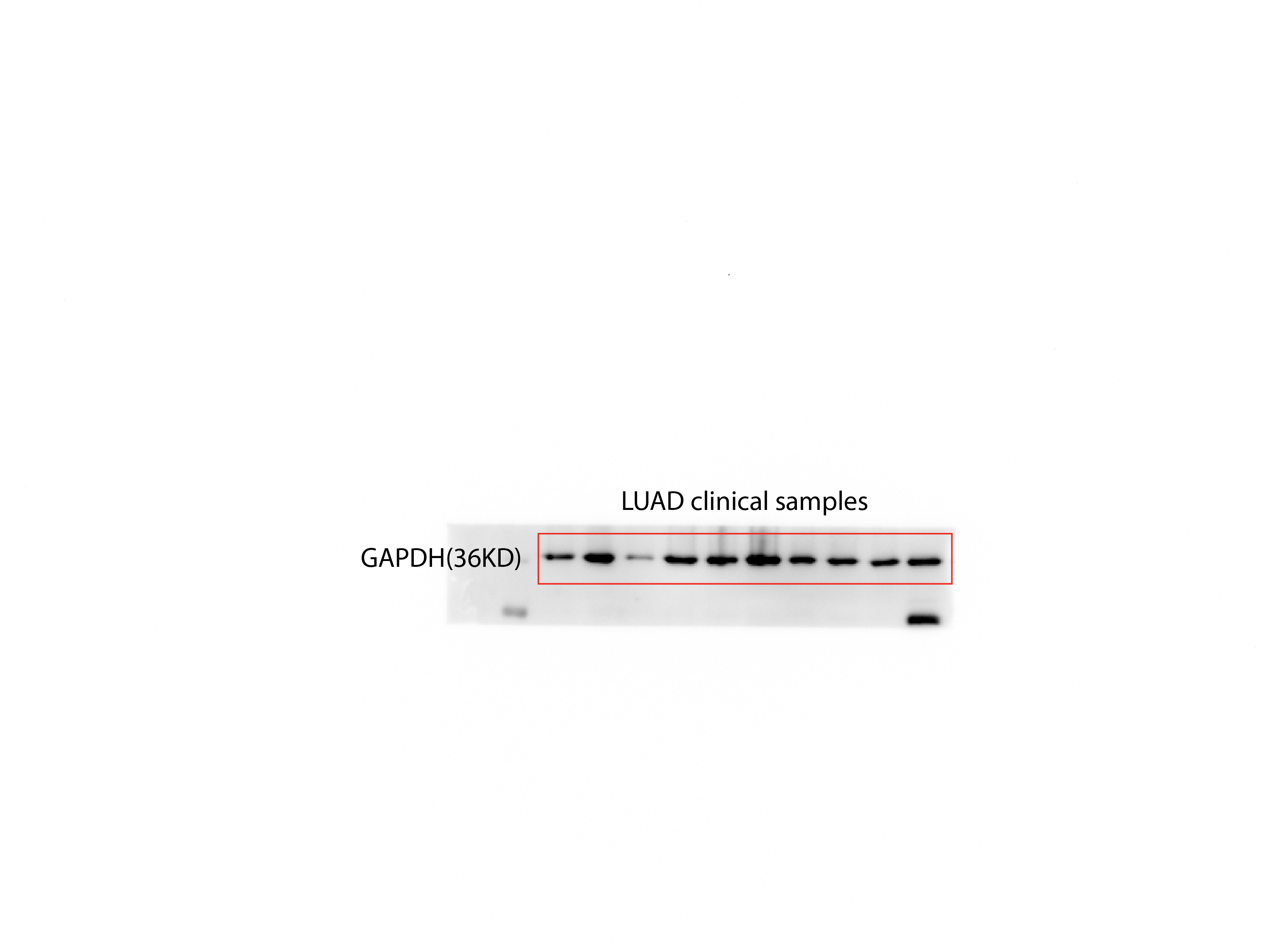

Supplement: Supplementary file 12 — Figure EV2 Source Data [file 44321_2026_460_MOESM12_ESM.zip › Source data Figure EV2/FIG EV2G/GAPDH.png]

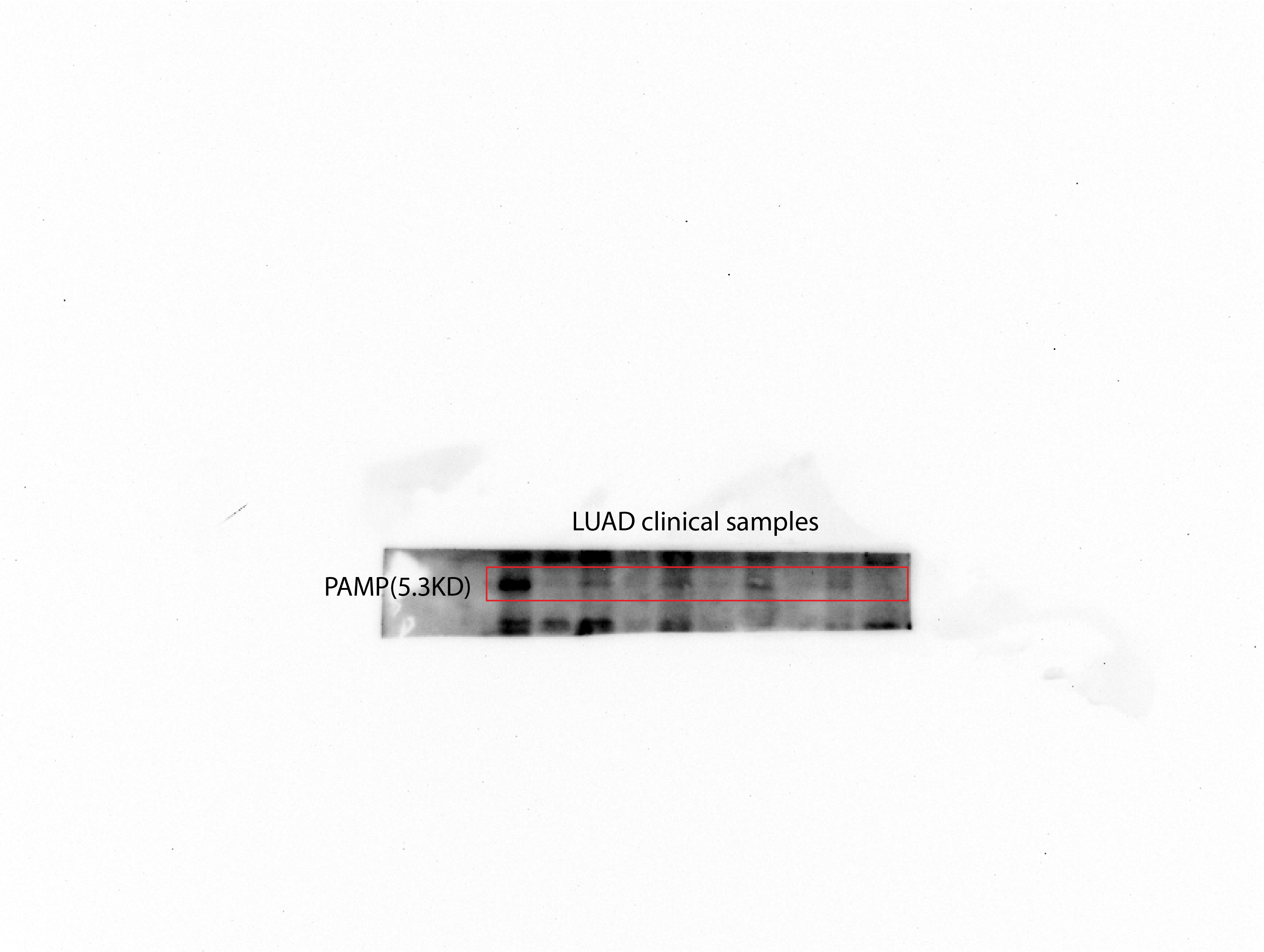

Supplement: Supplementary file 12 — Figure EV2 Source Data [file 44321_2026_460_MOESM12_ESM.zip › Source data Figure EV2/FIG EV2G/PAMP.png]

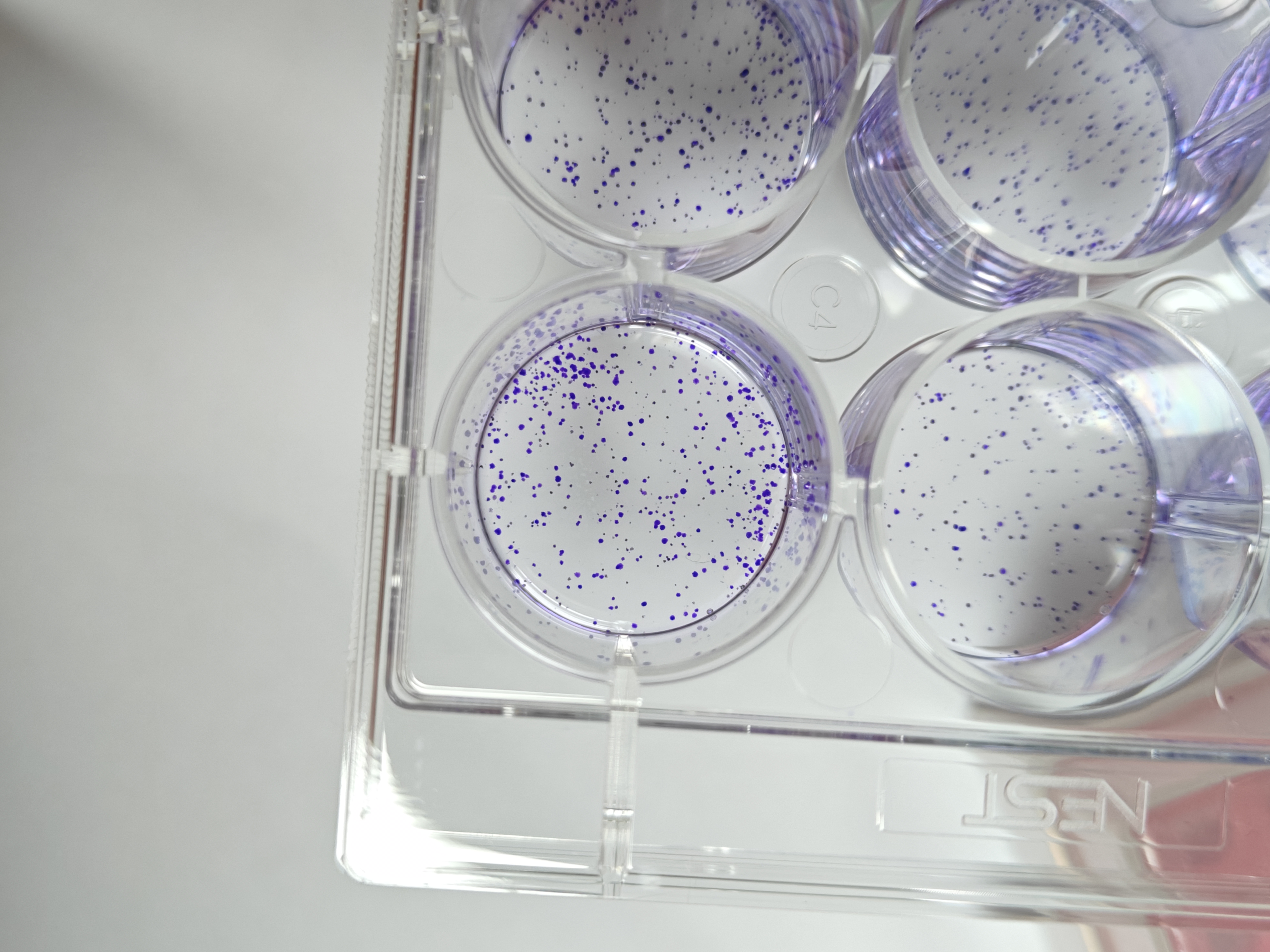

Supplement: Supplementary file 13 — Figure EV3 Source Data [file 44321_2026_460_MOESM13_ESM.zip › Source data Figure EV3/FIG EV3B/A549-ko pamp.jpg]

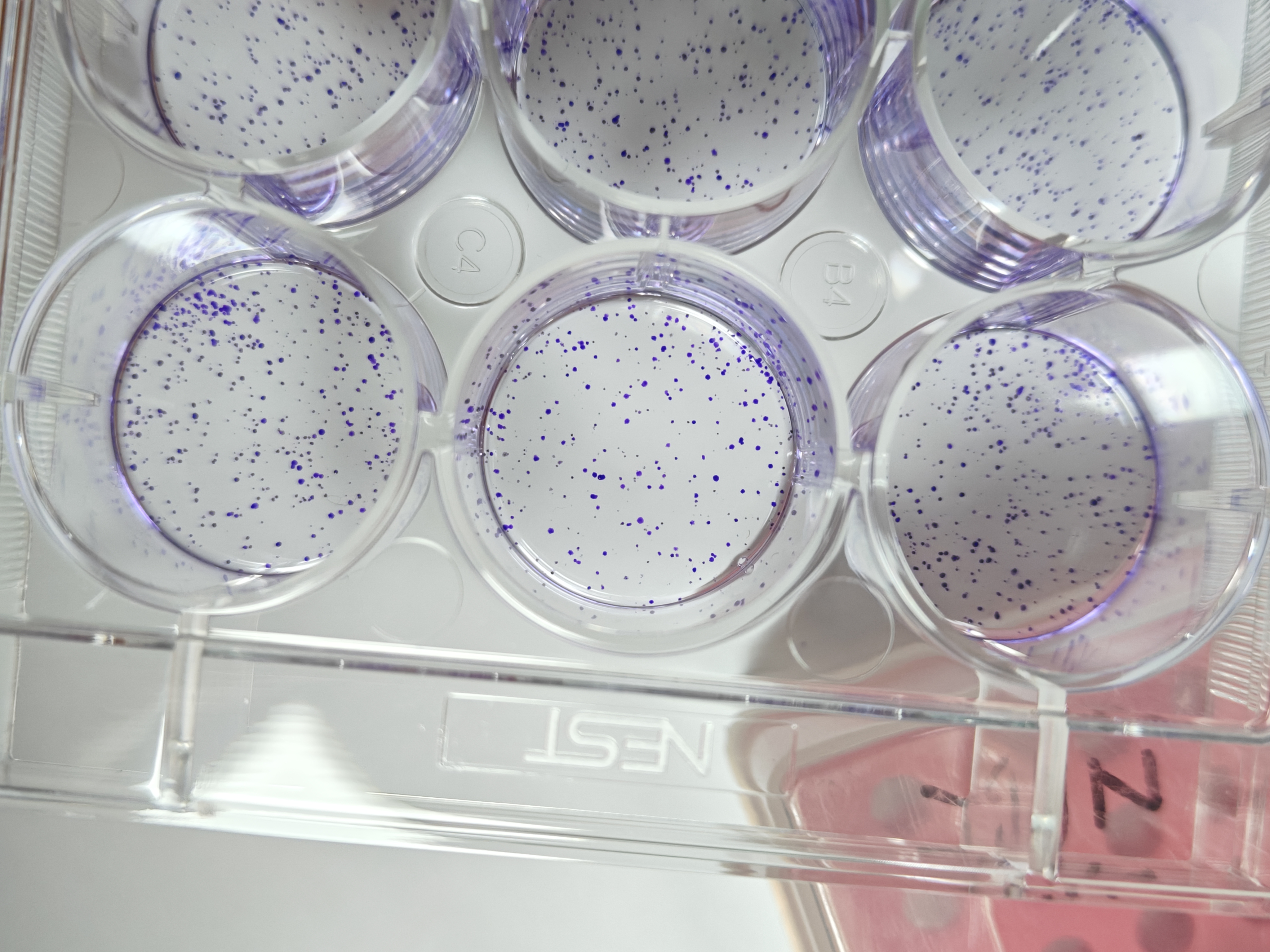

Supplement: Supplementary file 13 — Figure EV3 Source Data [file 44321_2026_460_MOESM13_ESM.zip › Source data Figure EV3/FIG EV3B/A549-LACZ.jpg]

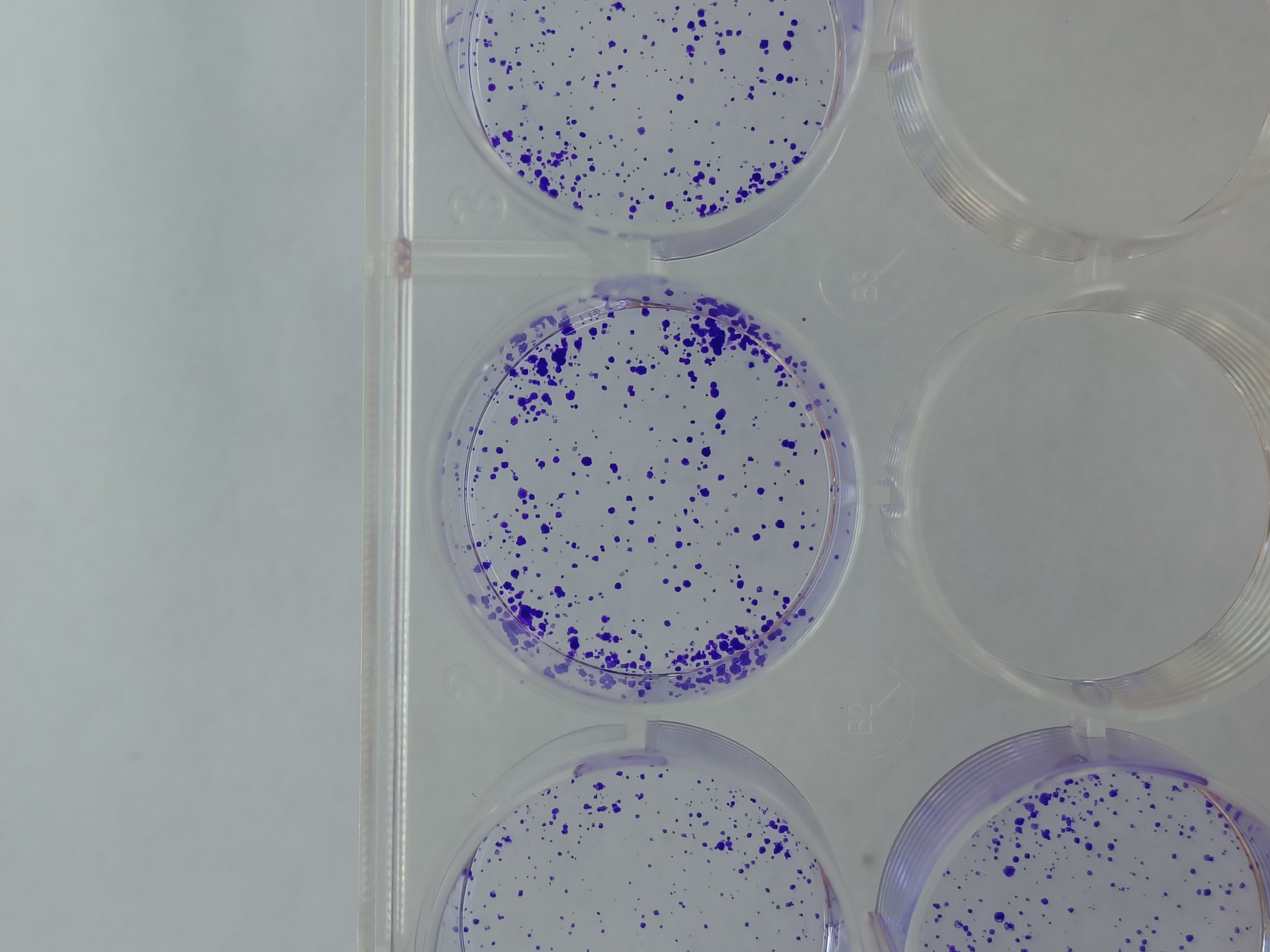

Supplement: Supplementary file 13 — Figure EV3 Source Data [file 44321_2026_460_MOESM13_ESM.zip › Source data Figure EV3/FIG EV3B/H460-KO PAMP.jpg]

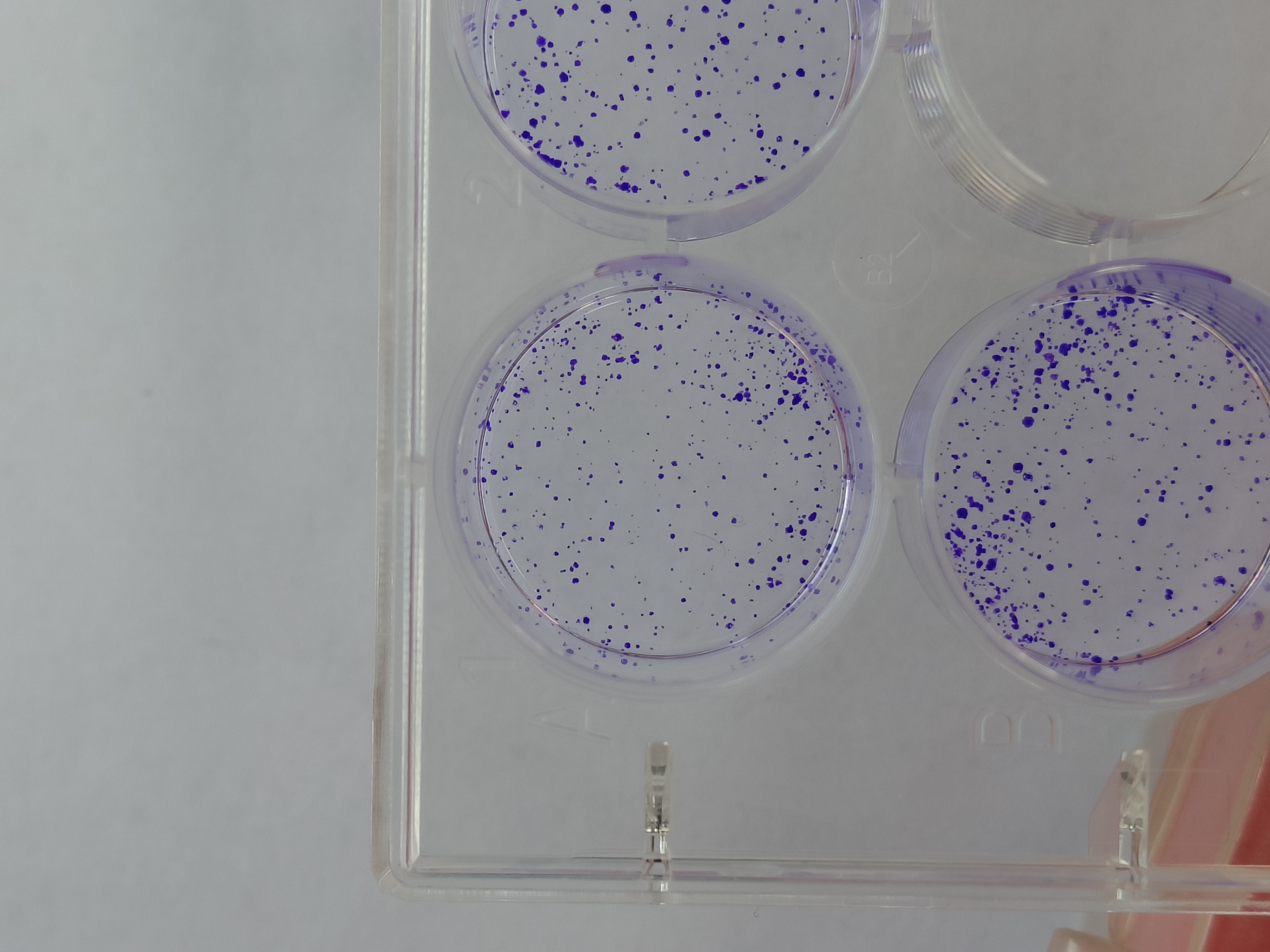

Supplement: Supplementary file 13 — Figure EV3 Source Data [file 44321_2026_460_MOESM13_ESM.zip › Source data Figure EV3/FIG EV3B/H460-lacz.jpg]

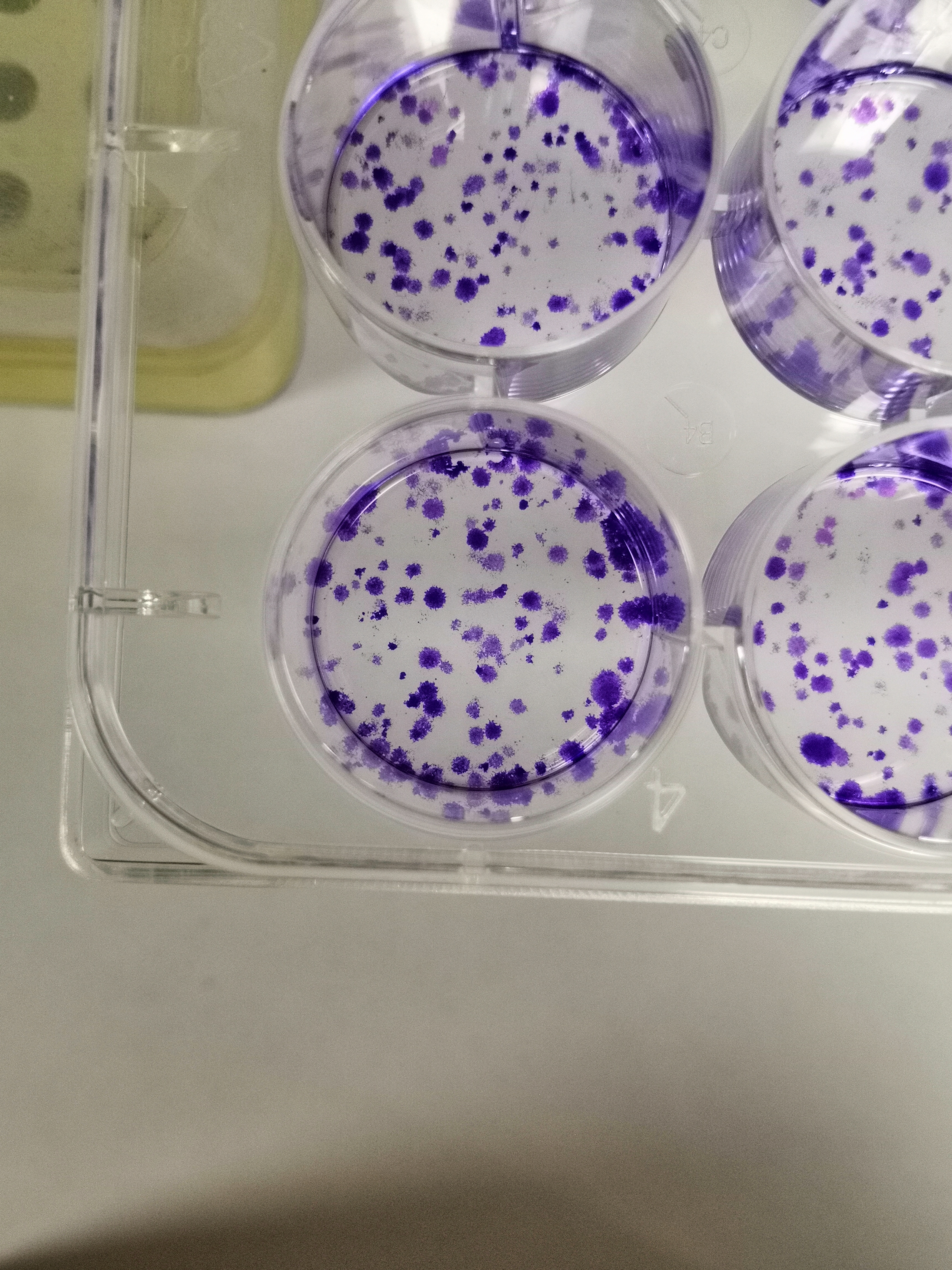

Supplement: Supplementary file 13 — Figure EV3 Source Data [file 44321_2026_460_MOESM13_ESM.zip › Source data Figure EV3/FIG EV3E/A549-EV.jpg]

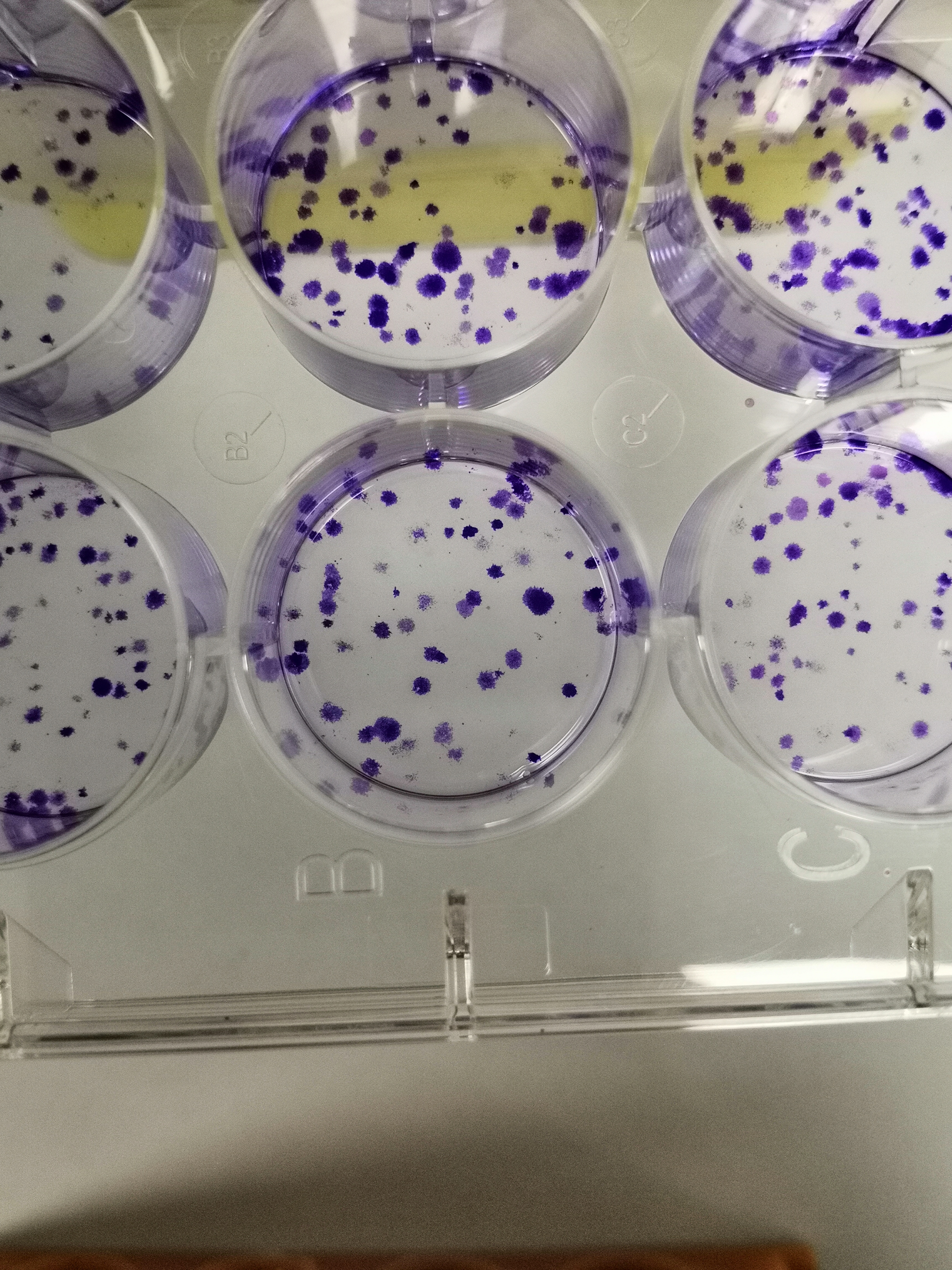

Supplement: Supplementary file 13 — Figure EV3 Source Data [file 44321_2026_460_MOESM13_ESM.zip › Source data Figure EV3/FIG EV3E/A549-PAMP.jpg]

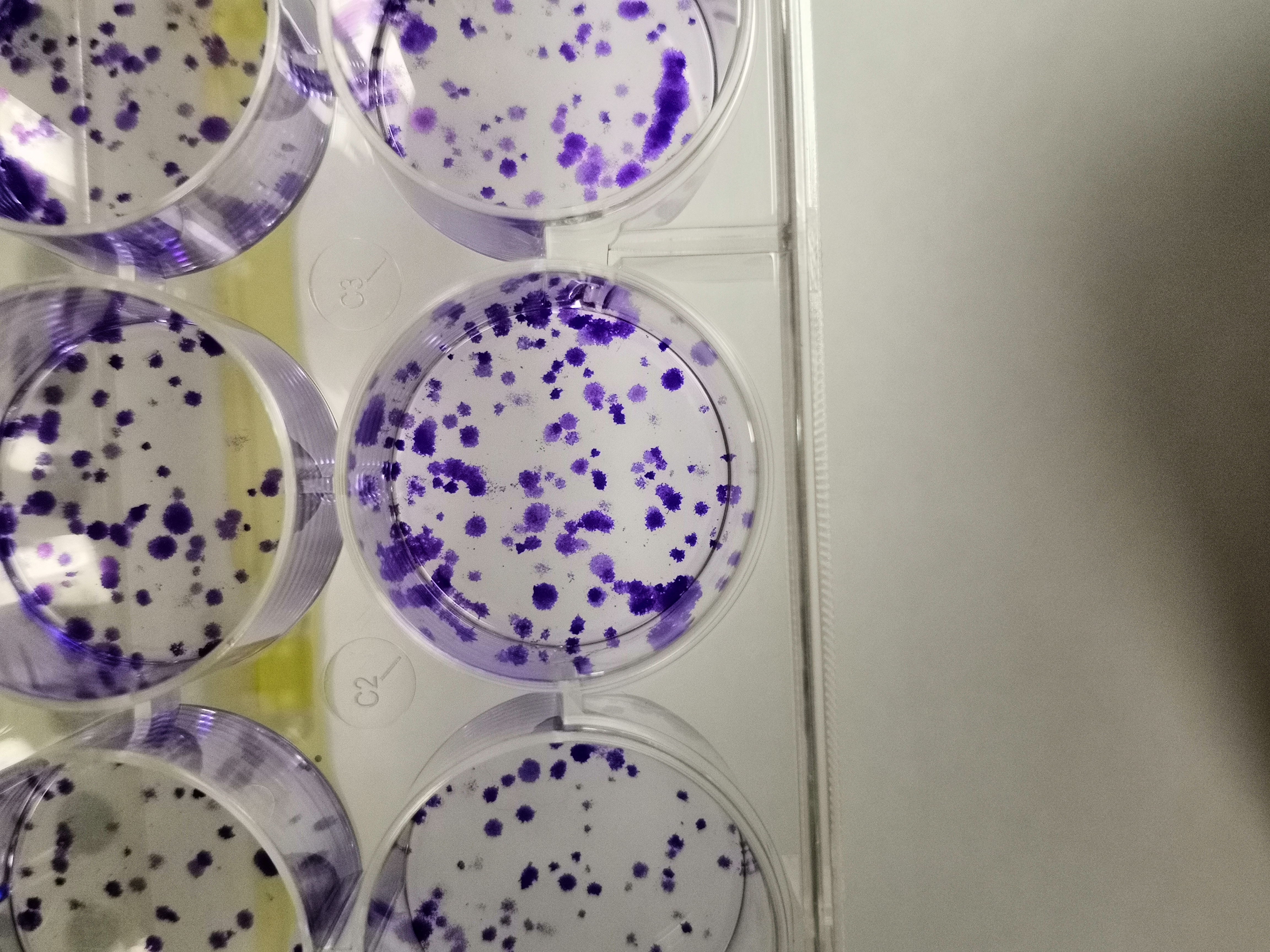

Supplement: Supplementary file 13 — Figure EV3 Source Data [file 44321_2026_460_MOESM13_ESM.zip › Source data Figure EV3/FIG EV3E/A549-PAMPmut.jpg]

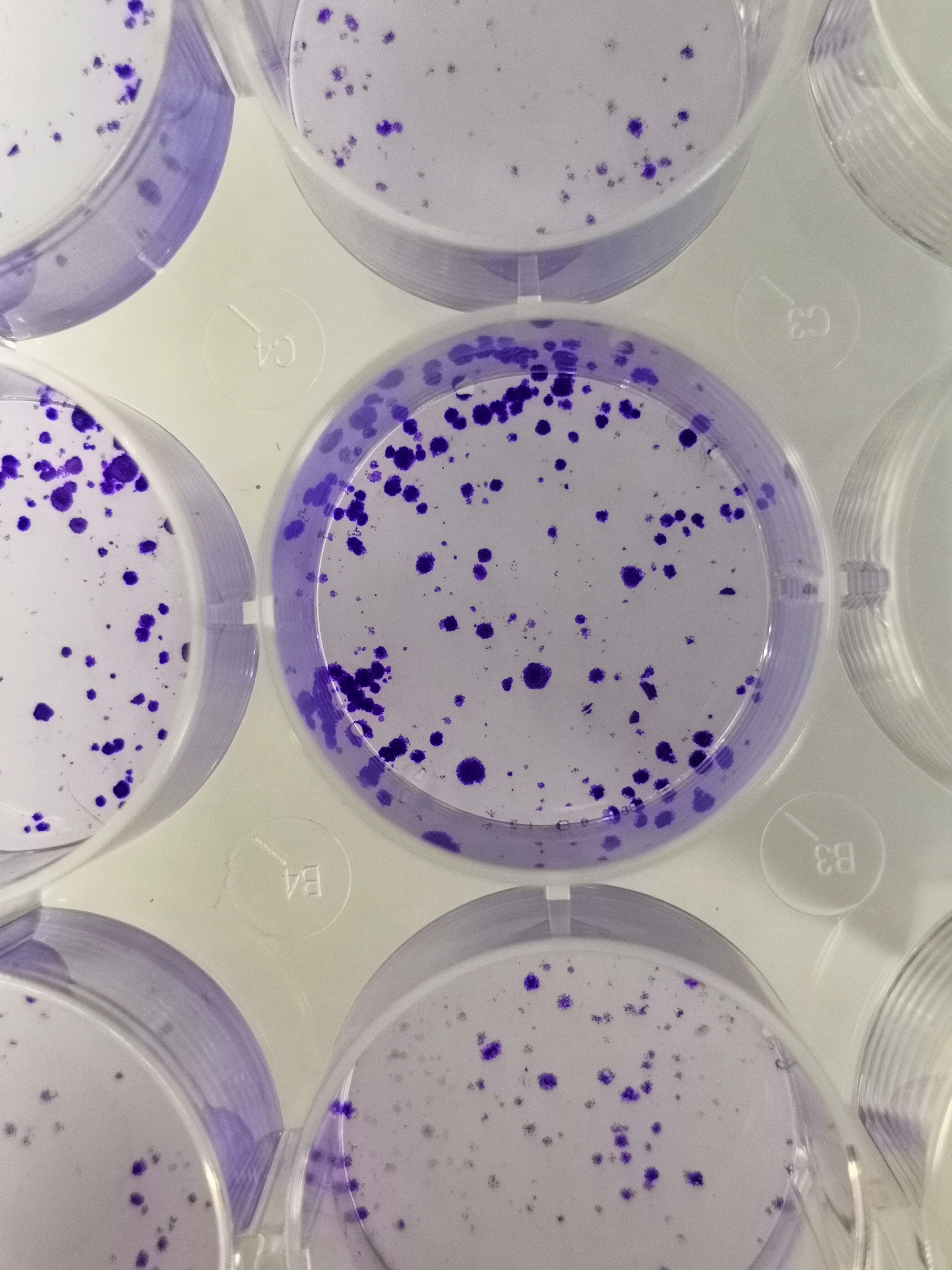

Supplement: Supplementary file 13 — Figure EV3 Source Data [file 44321_2026_460_MOESM13_ESM.zip › Source data Figure EV3/FIG EV3E/H460-EV.jpg]

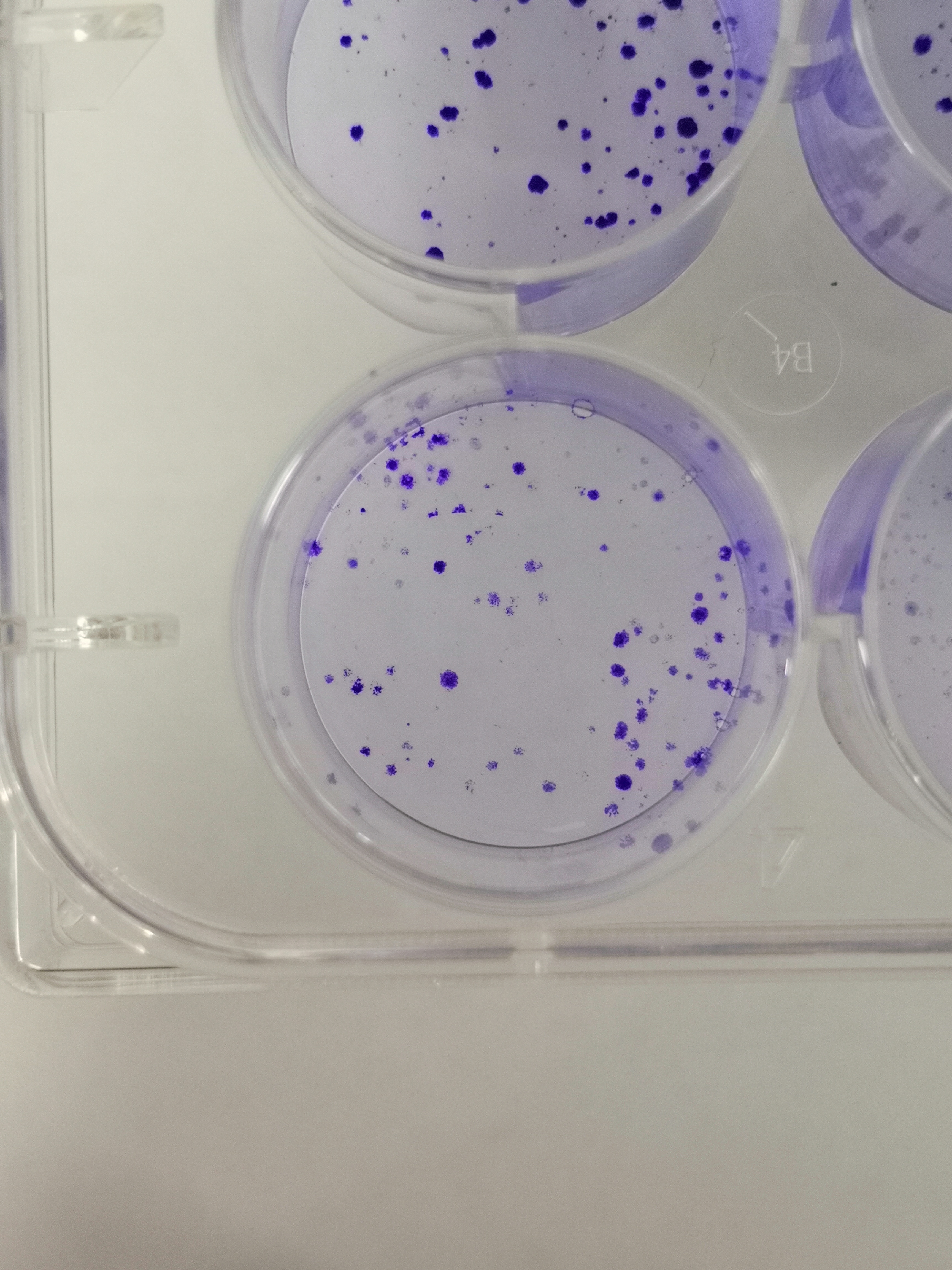

Supplement: Supplementary file 13 — Figure EV3 Source Data [file 44321_2026_460_MOESM13_ESM.zip › Source data Figure EV3/FIG EV3E/H460-PAMP.jpg]

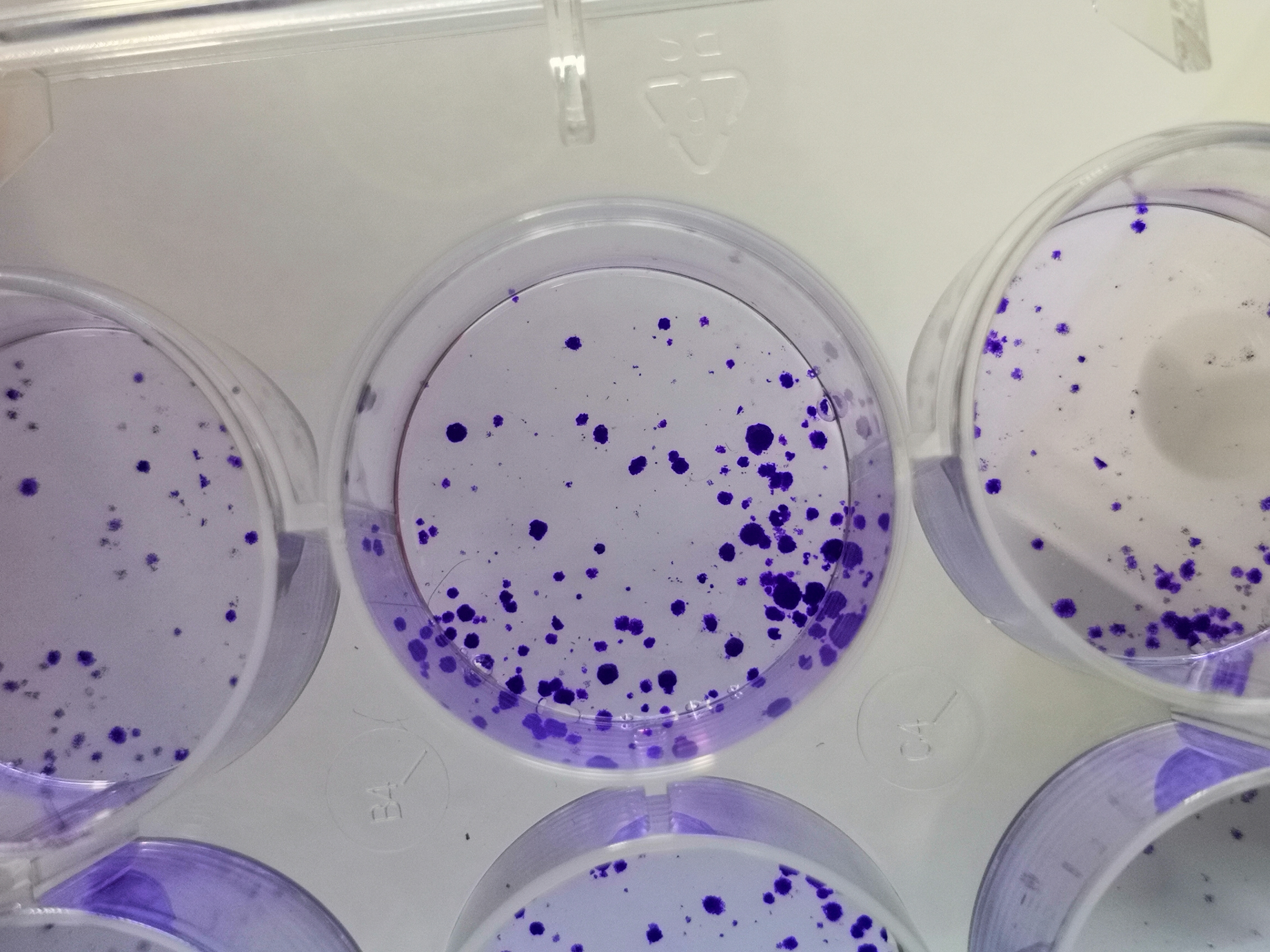

Supplement: Supplementary file 13 — Figure EV3 Source Data [file 44321_2026_460_MOESM13_ESM.zip › Source data Figure EV3/FIG EV3E/H460-PAMPmut.jpg]

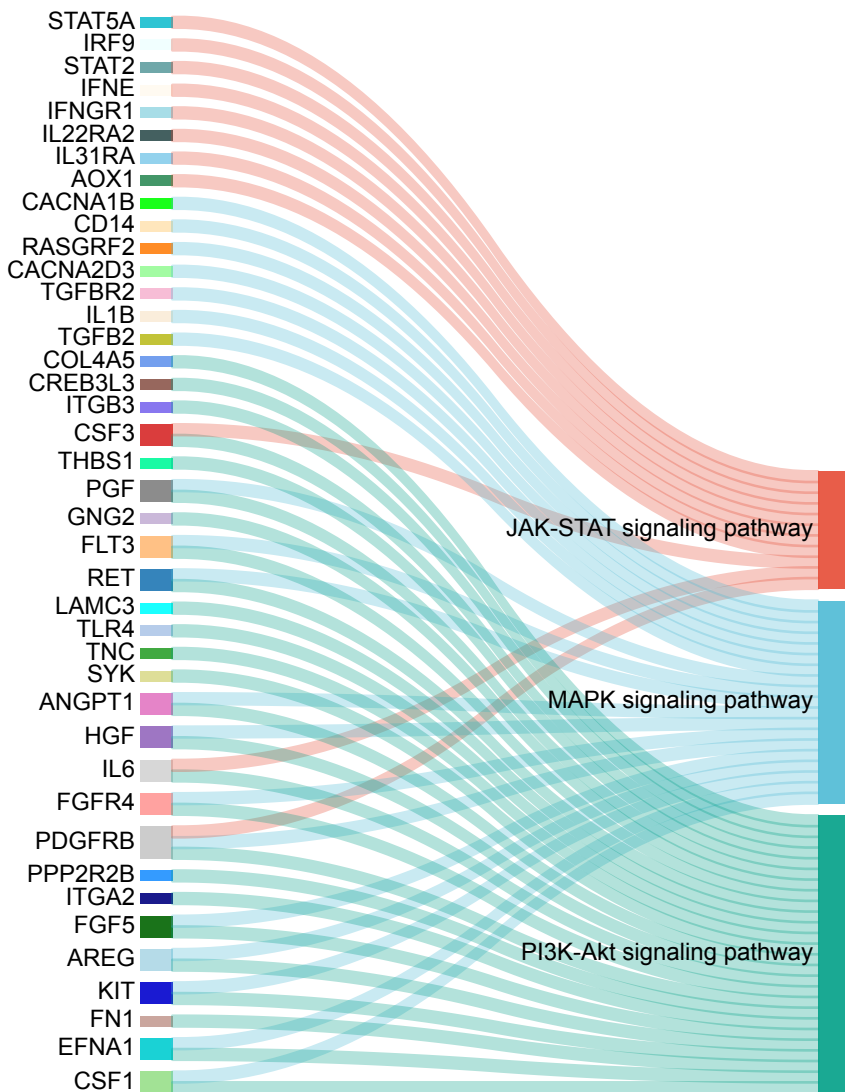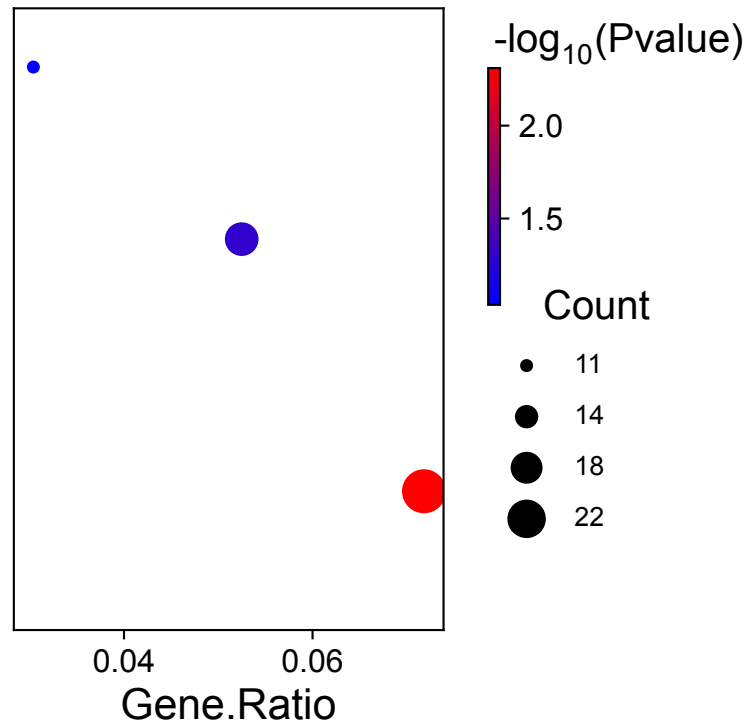

Supplement: Supplementary file 15 — Figure EV5 Source Data [file 44321_2026_460_MOESM15_ESM.zip › Source data Figure EV5/FIG EV5E.pdf]

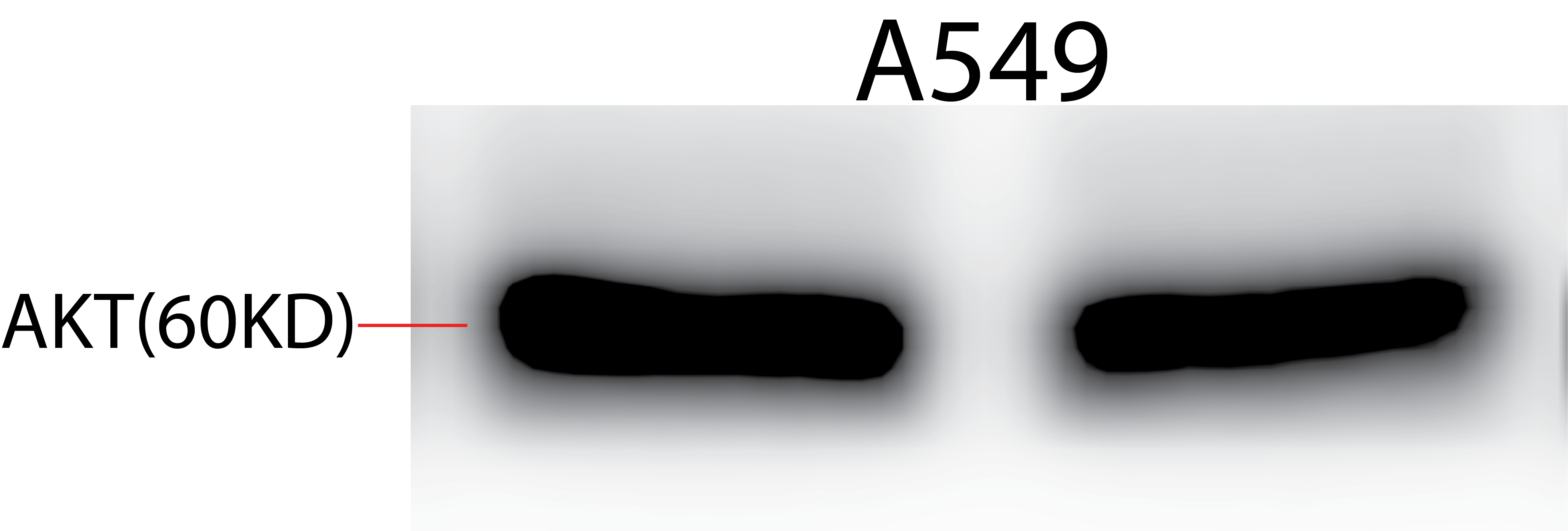

Supplement: Supplementary file 15 — Figure EV5 Source Data [file 44321_2026_460_MOESM15_ESM.zip › Source data Figure EV5/FIG EV5F/AKT-1.png]

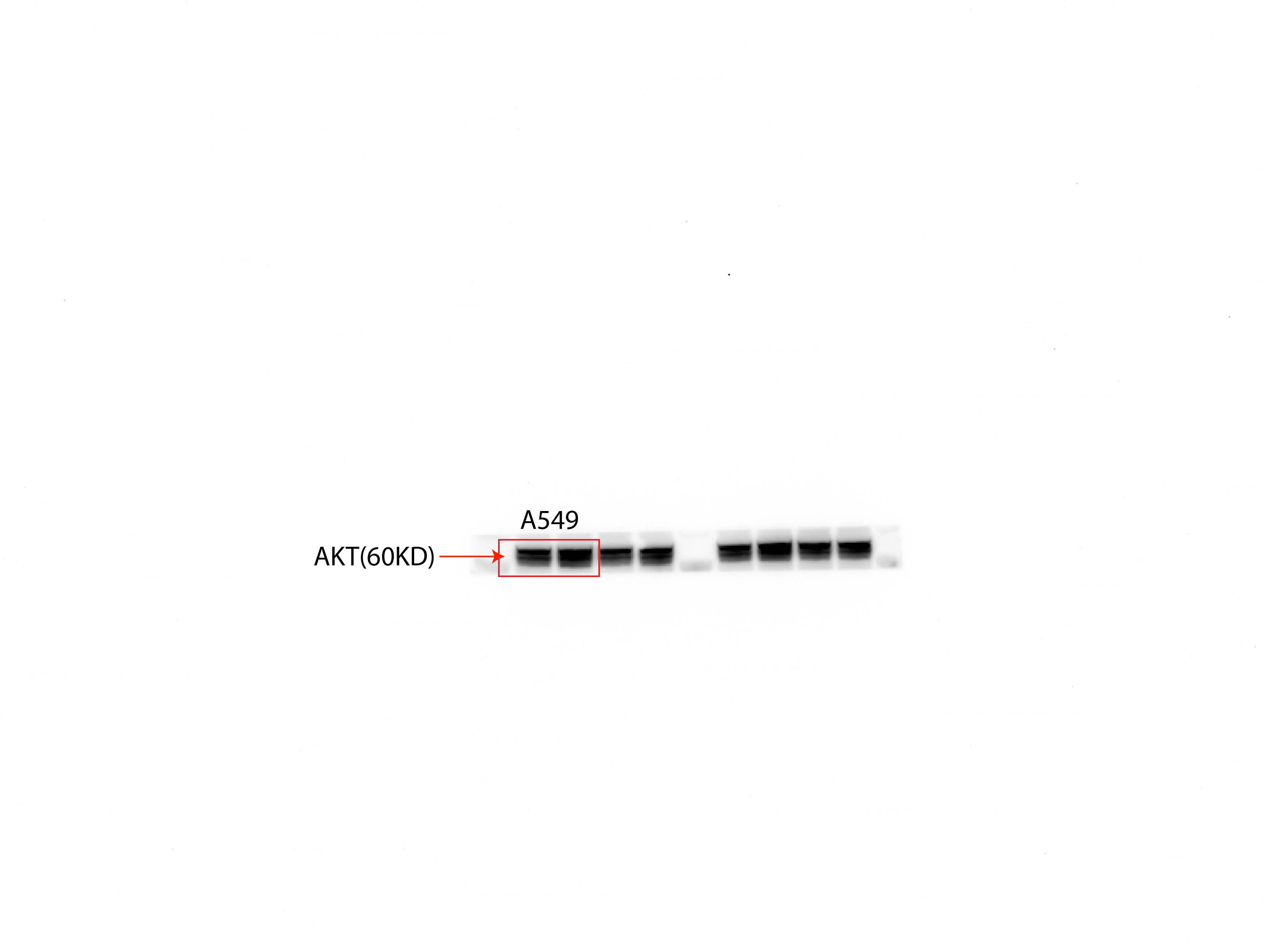

Supplement: Supplementary file 15 — Figure EV5 Source Data [file 44321_2026_460_MOESM15_ESM.zip › Source data Figure EV5/FIG EV5F/AKT-2.png]

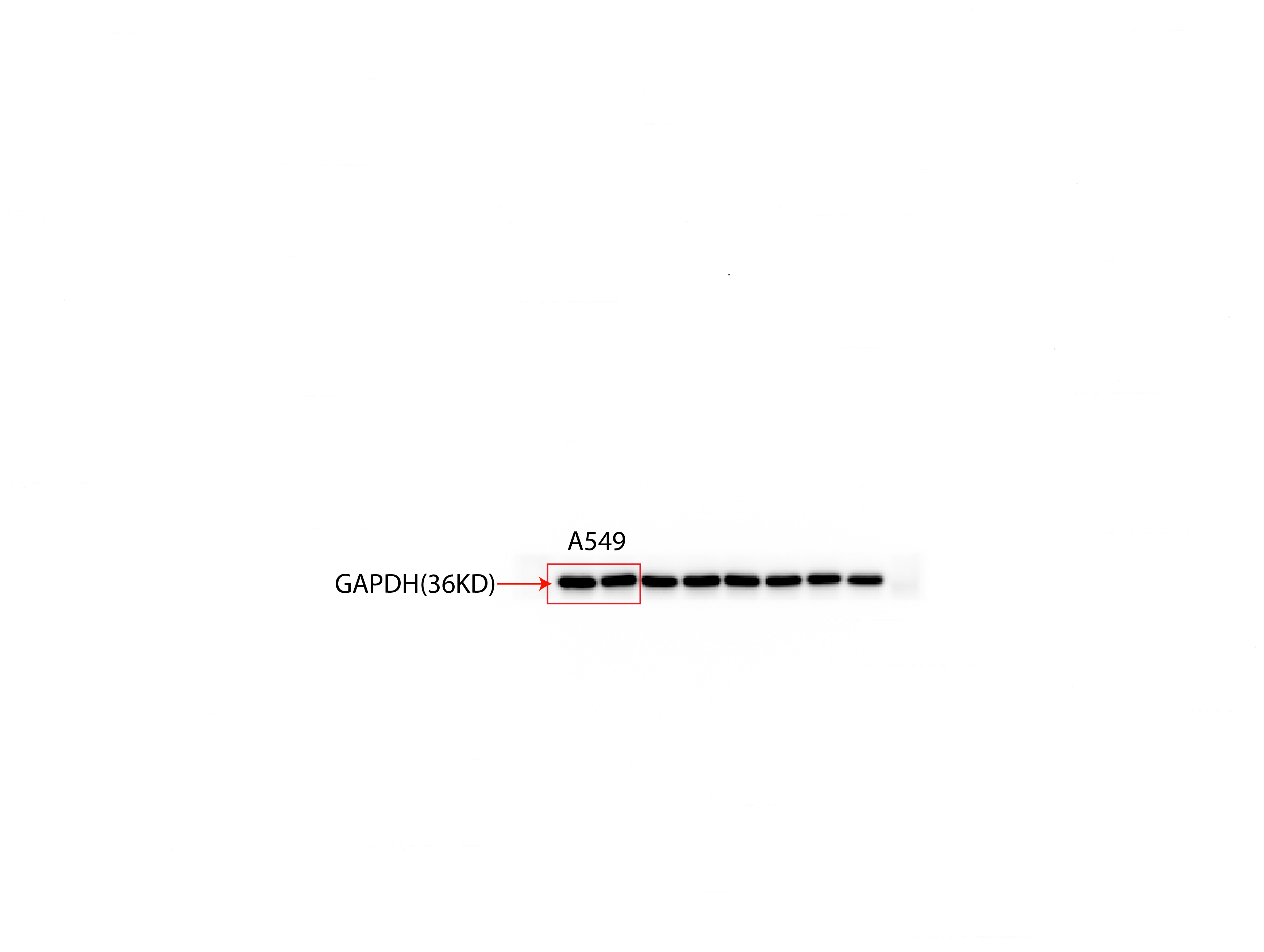

Supplement: Supplementary file 15 — Figure EV5 Source Data [file 44321_2026_460_MOESM15_ESM.zip › Source data Figure EV5/FIG EV5F/GAPDH-2.png]

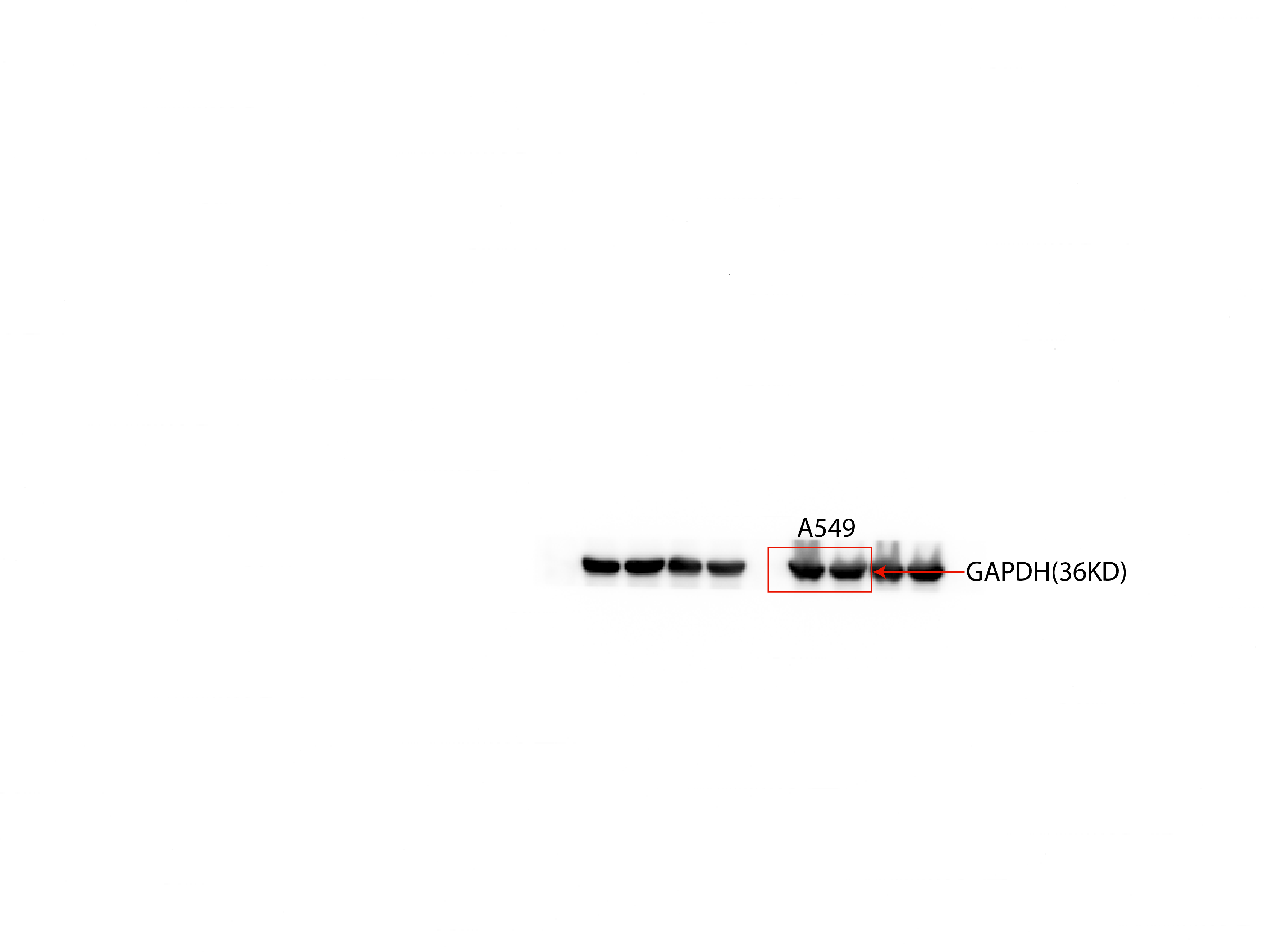

Supplement: Supplementary file 15 — Figure EV5 Source Data [file 44321_2026_460_MOESM15_ESM.zip › Source data Figure EV5/FIG EV5F/GAPDH1.png]

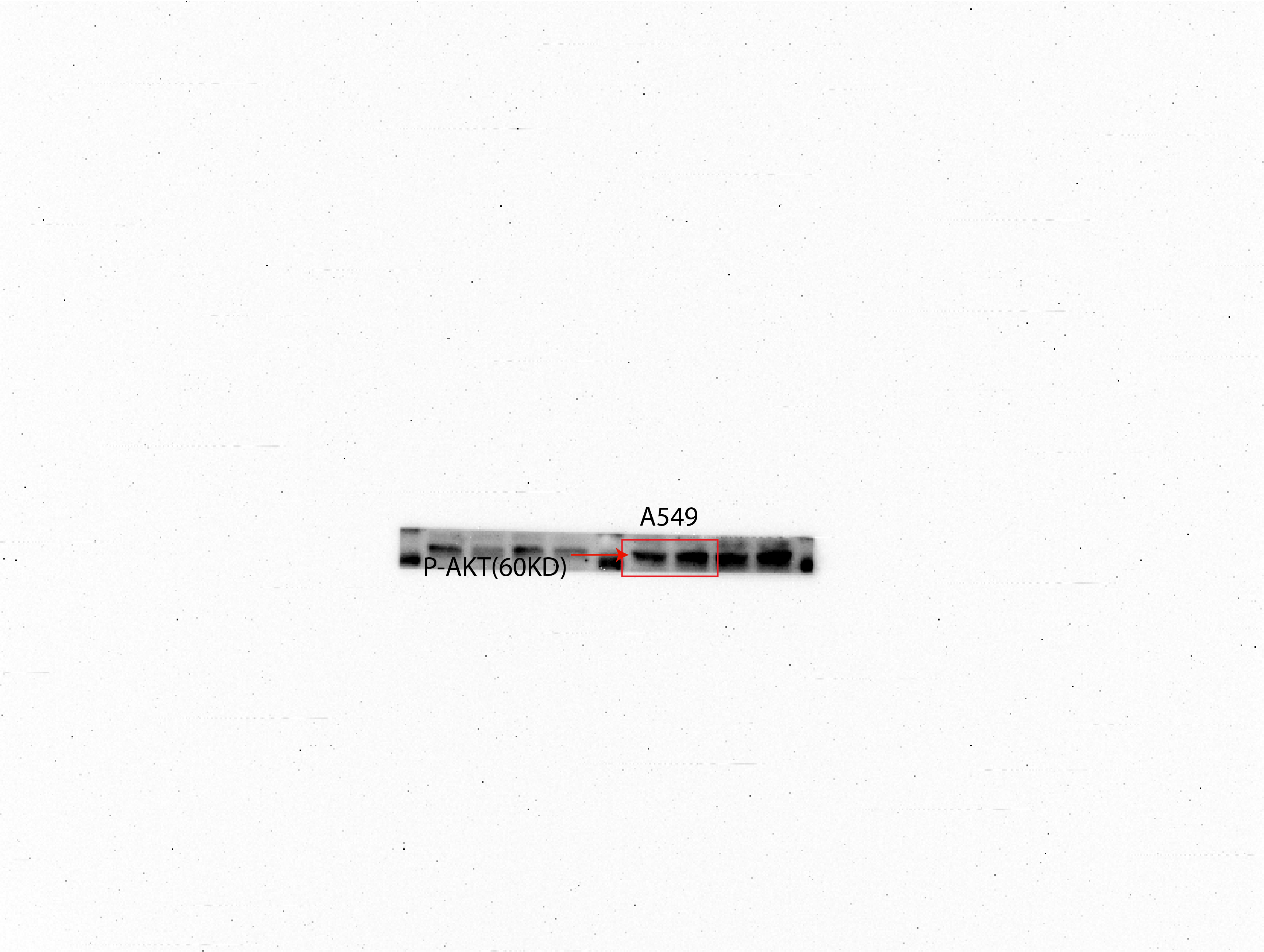

Supplement: Supplementary file 15 — Figure EV5 Source Data [file 44321_2026_460_MOESM15_ESM.zip › Source data Figure EV5/FIG EV5F/P-AKT-1.png]

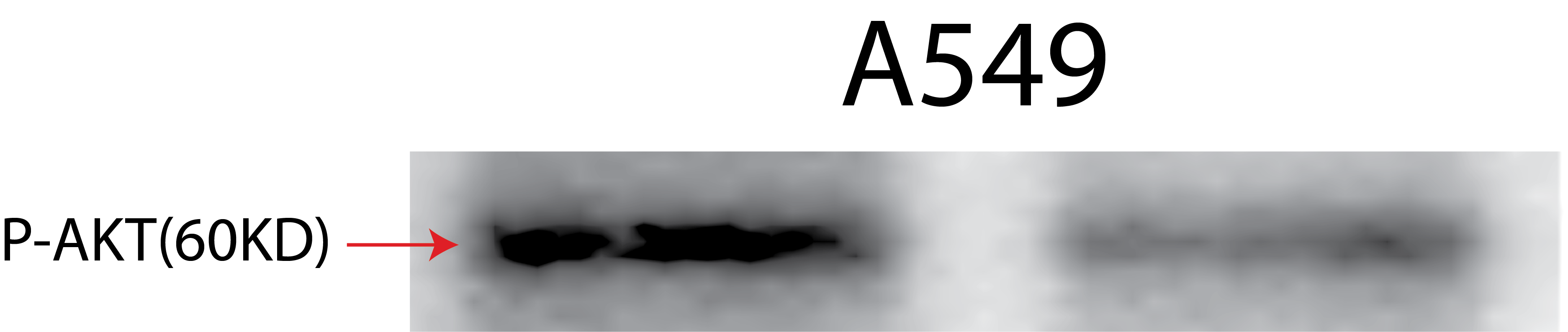

Supplement: Supplementary file 15 — Figure EV5 Source Data [file 44321_2026_460_MOESM15_ESM.zip › Source data Figure EV5/FIG EV5F/P-AKT-2.png]

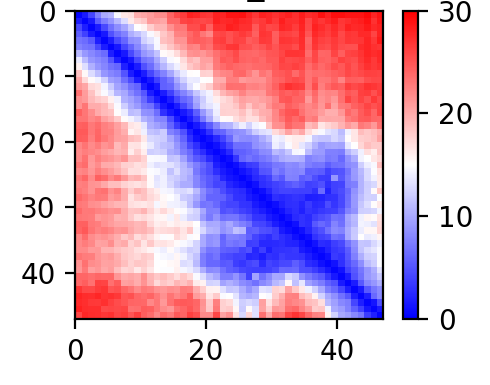

Supplement: Supplementary file 16 — Figure EV6 Source Data [file 44321_2026_460_MOESM16_ESM.zip › Source data Figure EV6/FIG EV6B.png]

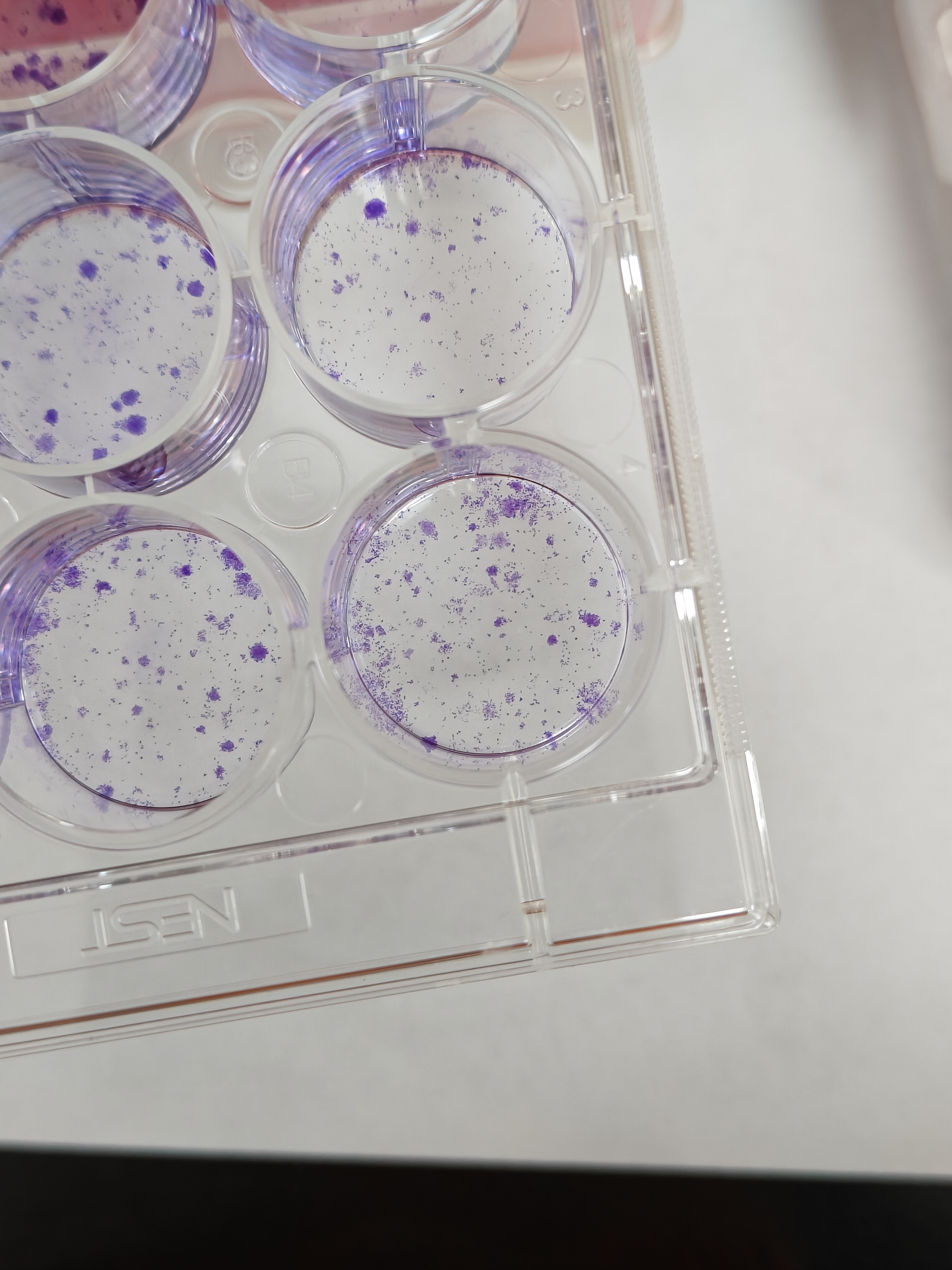

Supplement: Supplementary file 16 — Figure EV6 Source Data [file 44321_2026_460_MOESM16_ESM.zip › Source data Figure EV6/FIG EV6D/122.jpg]

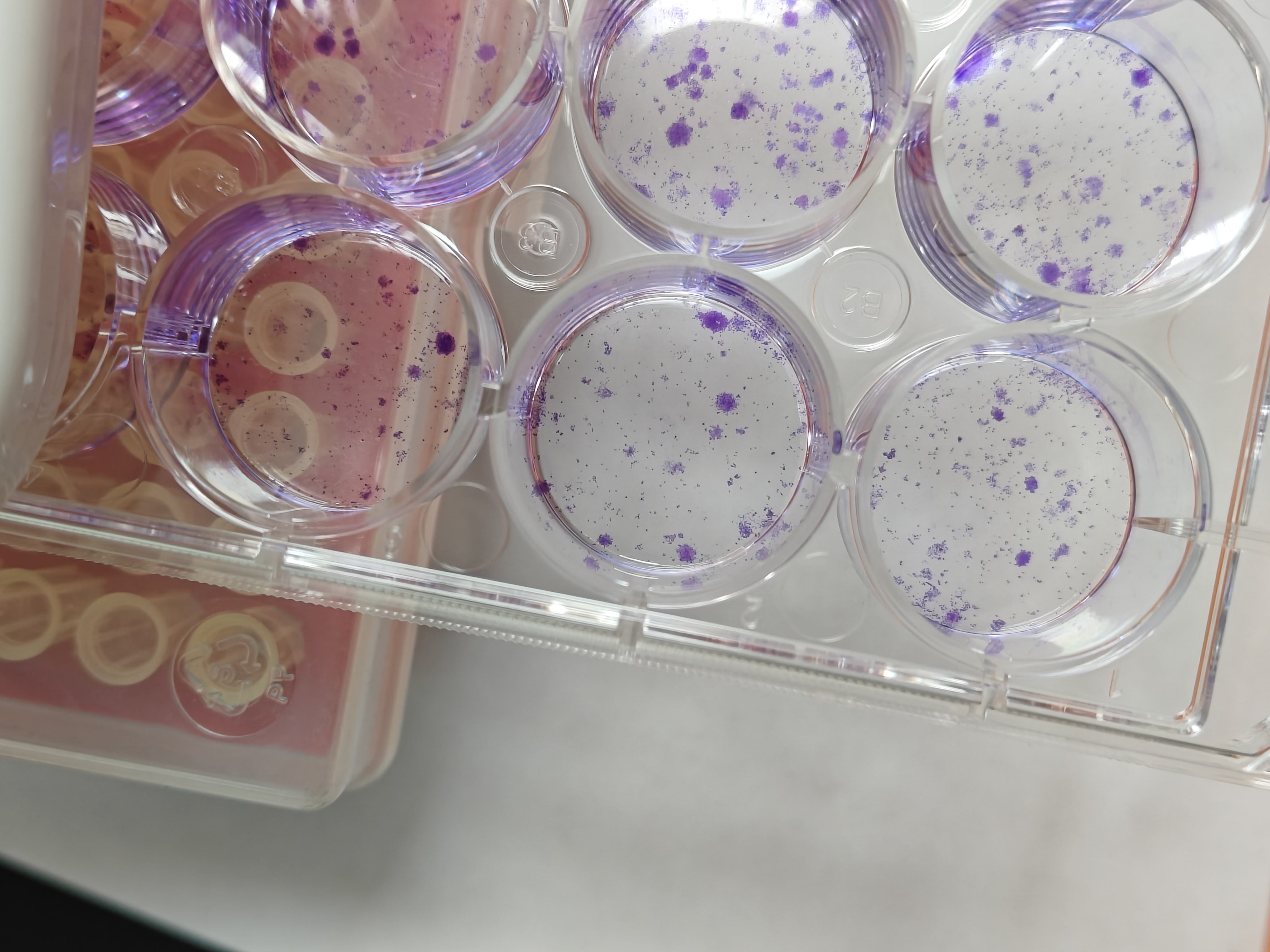

Supplement: Supplementary file 16 — Figure EV6 Source Data [file 44321_2026_460_MOESM16_ESM.zip › Source data Figure EV6/FIG EV6D/123.jpg]

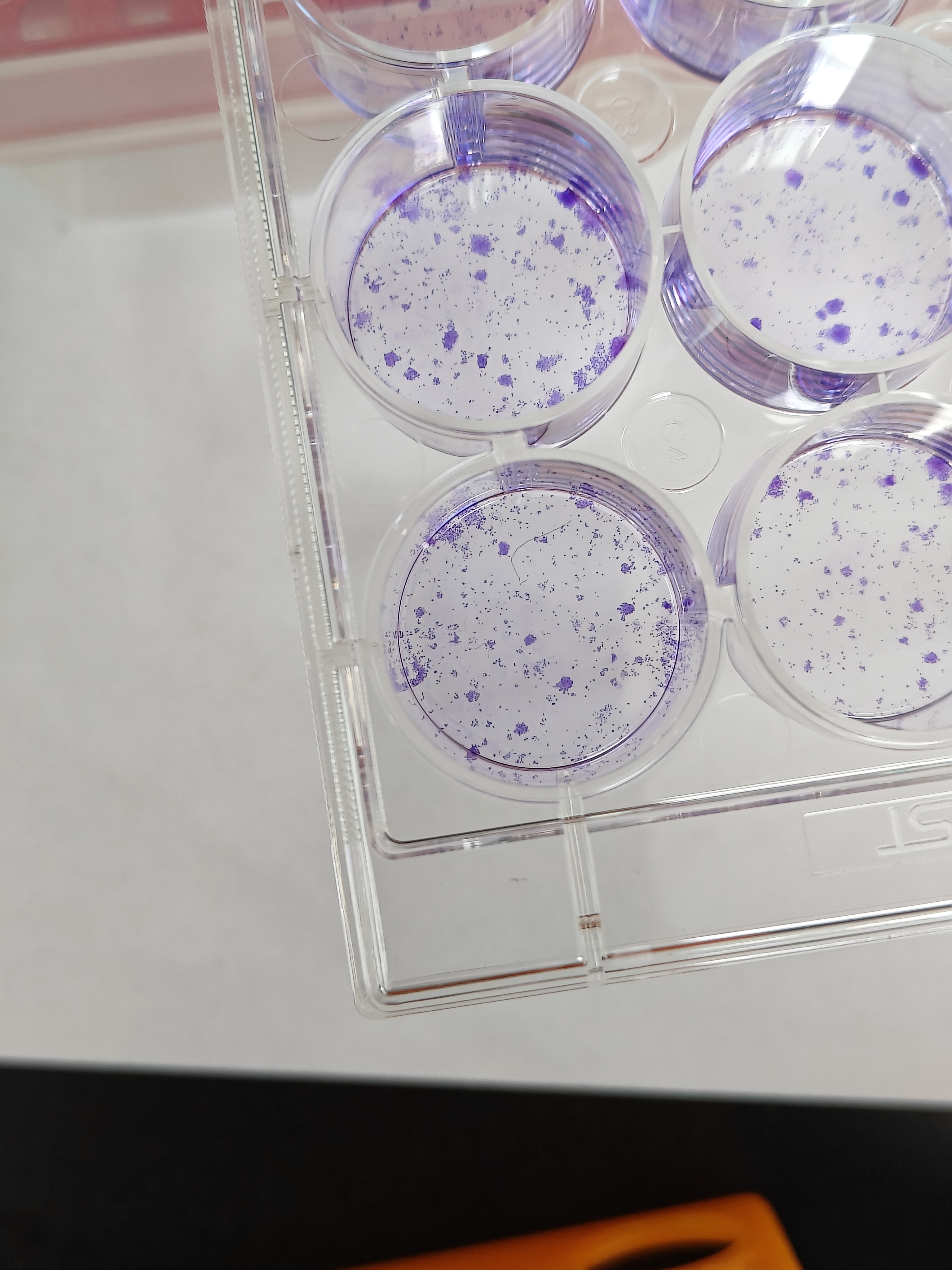

Supplement: Supplementary file 16 — Figure EV6 Source Data [file 44321_2026_460_MOESM16_ESM.zip › Source data Figure EV6/FIG EV6D/208.jpg]

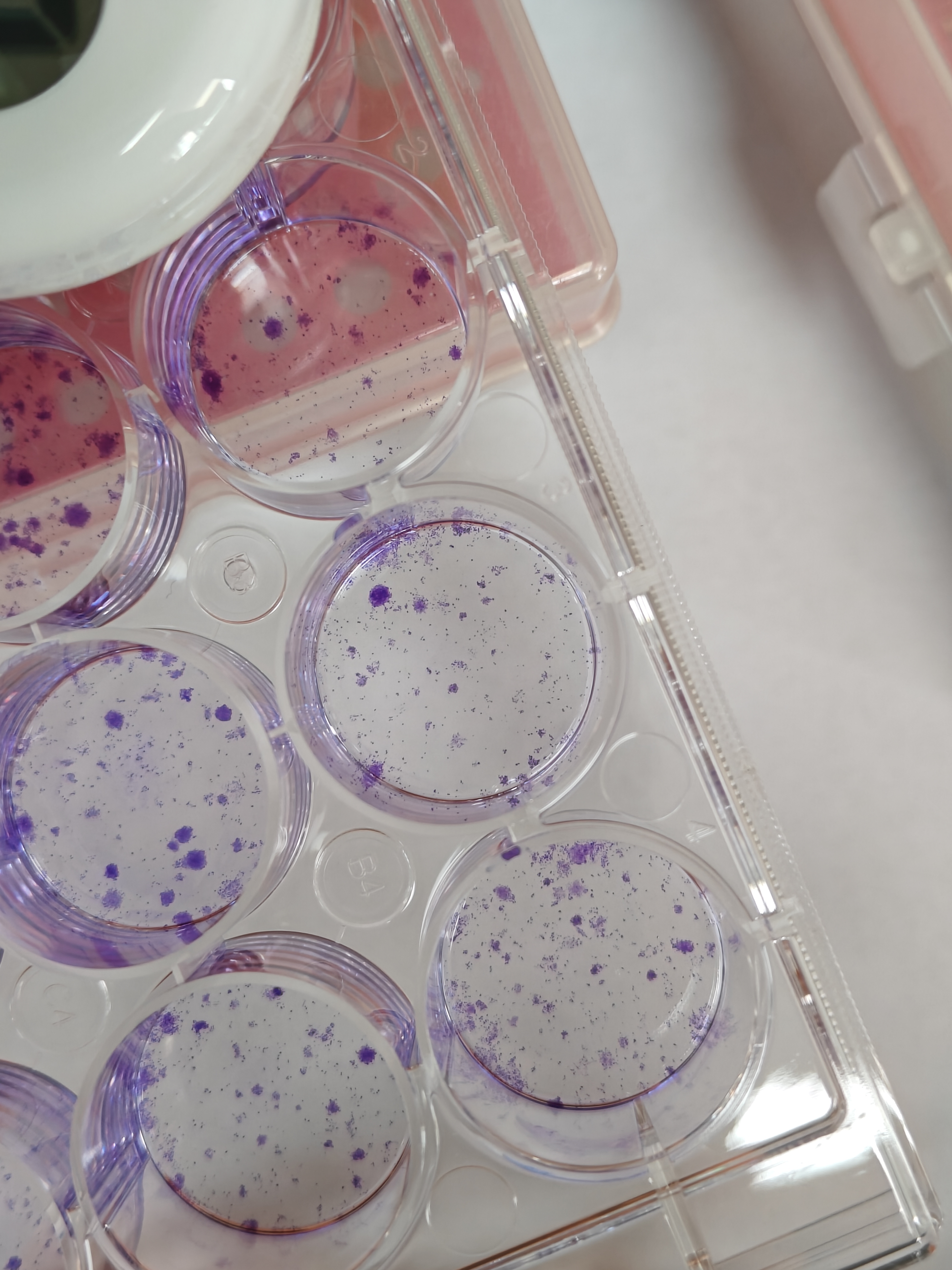

Supplement: Supplementary file 16 — Figure EV6 Source Data [file 44321_2026_460_MOESM16_ESM.zip › Source data Figure EV6/FIG EV6D/EV.jpg]

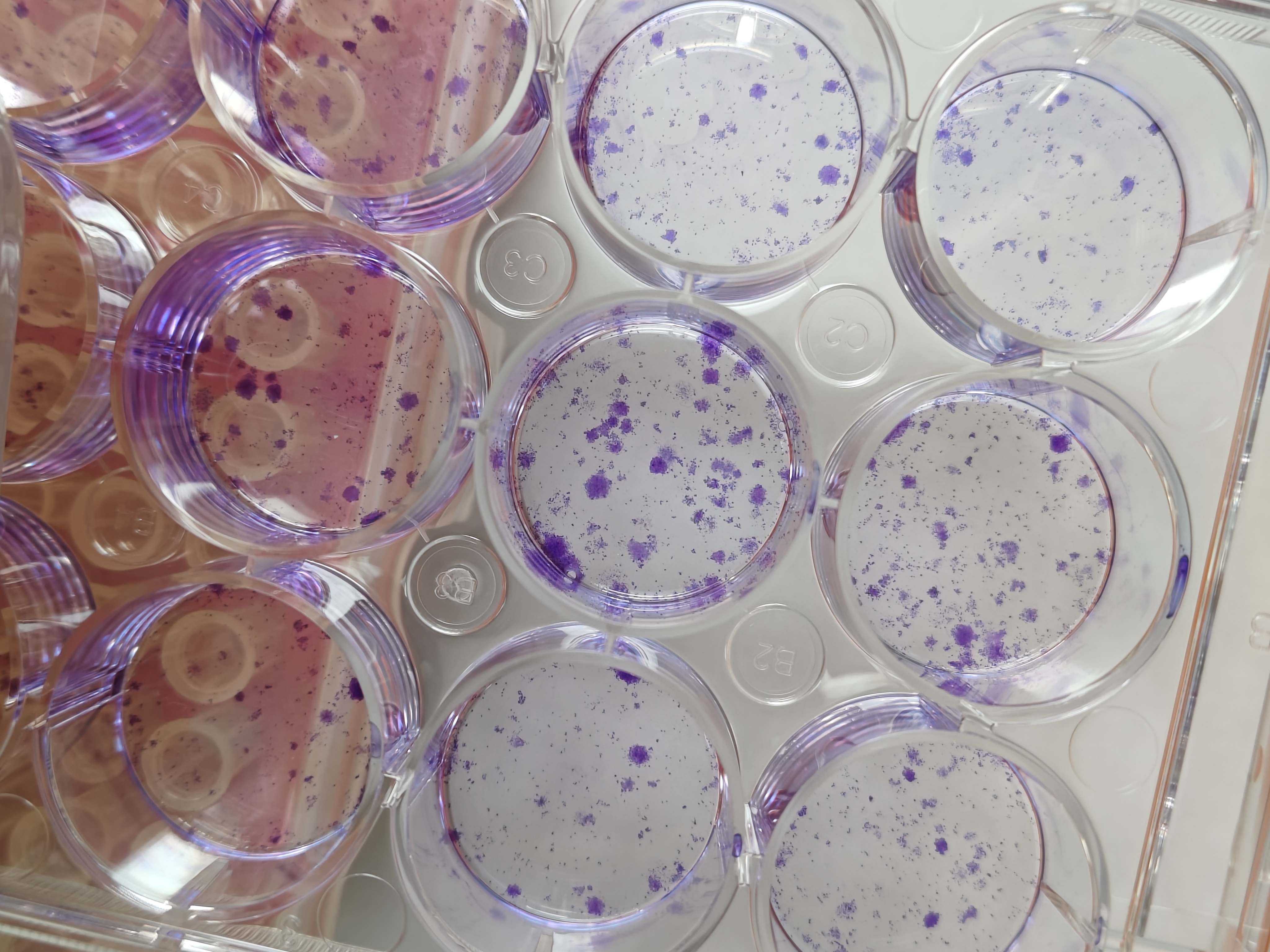

Supplement: Supplementary file 16 — Figure EV6 Source Data [file 44321_2026_460_MOESM16_ESM.zip › Source data Figure EV6/FIG EV6D/WT(PYCR1).jpg]

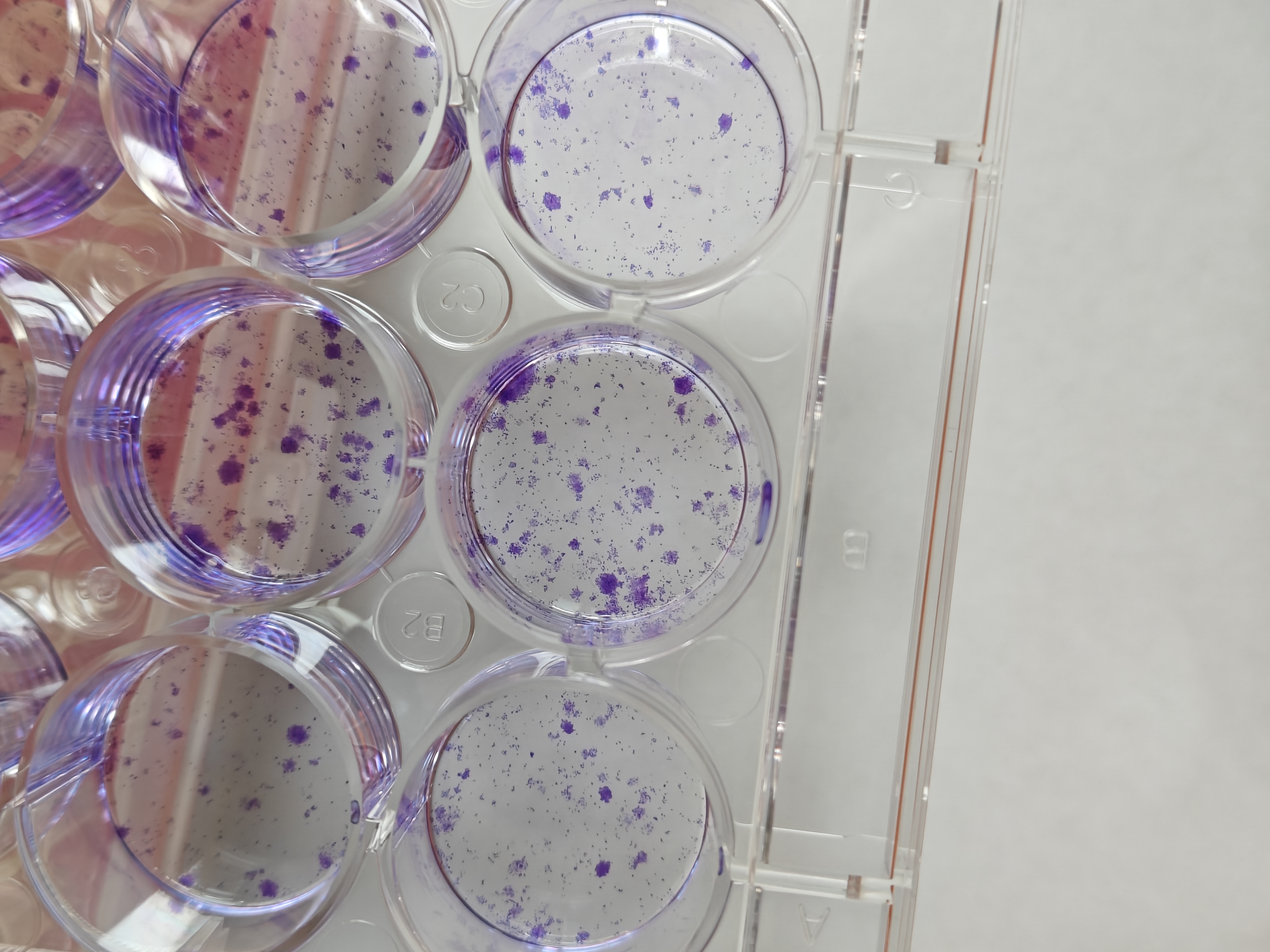

Supplement: Supplementary file 16 — Figure EV6 Source Data [file 44321_2026_460_MOESM16_ESM.zip › Source data Figure EV6/FIG EV6E/EV.jpg]

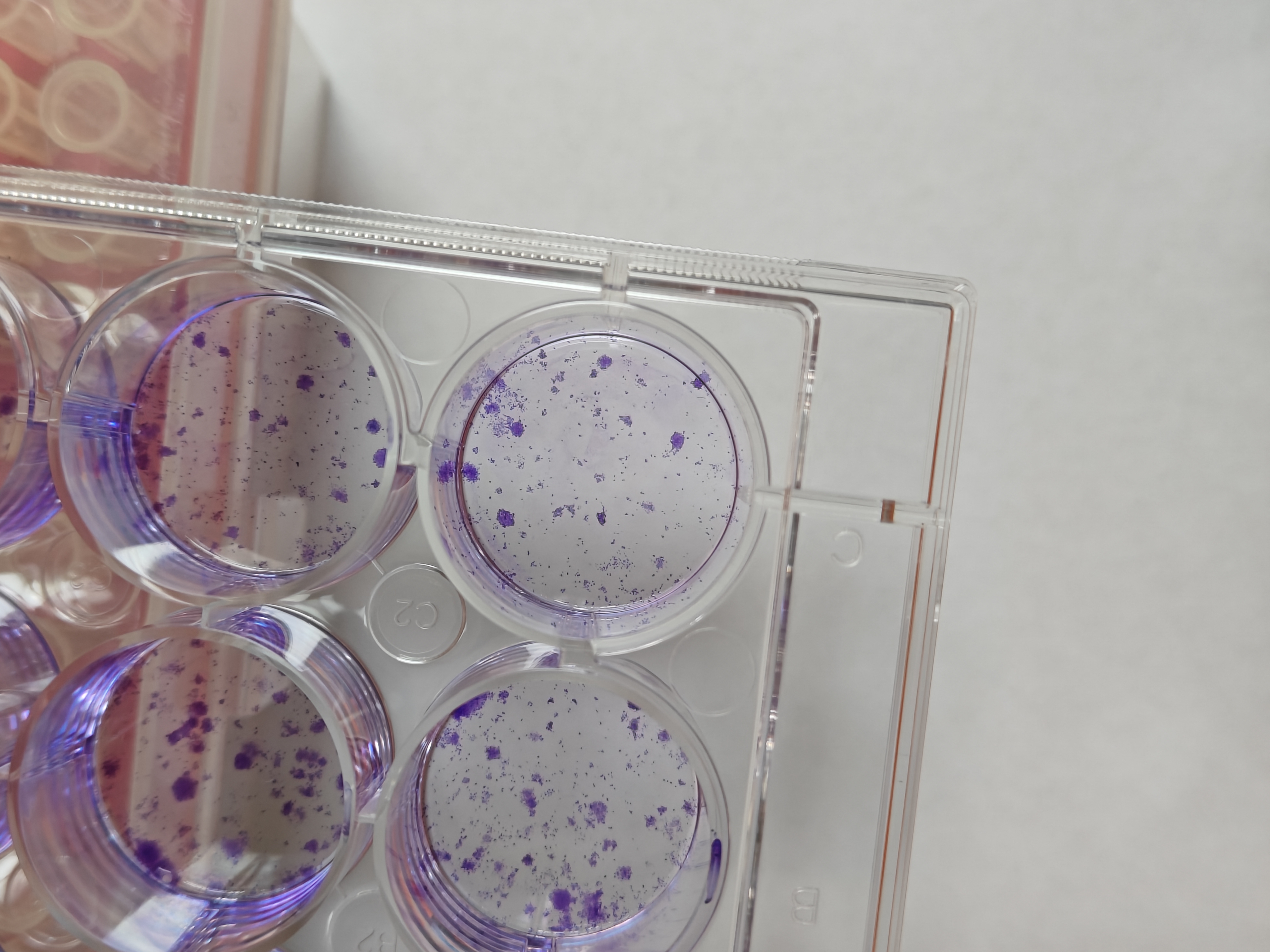

Supplement: Supplementary file 16 — Figure EV6 Source Data [file 44321_2026_460_MOESM16_ESM.zip › Source data Figure EV6/FIG EV6E/F16A.jpg]

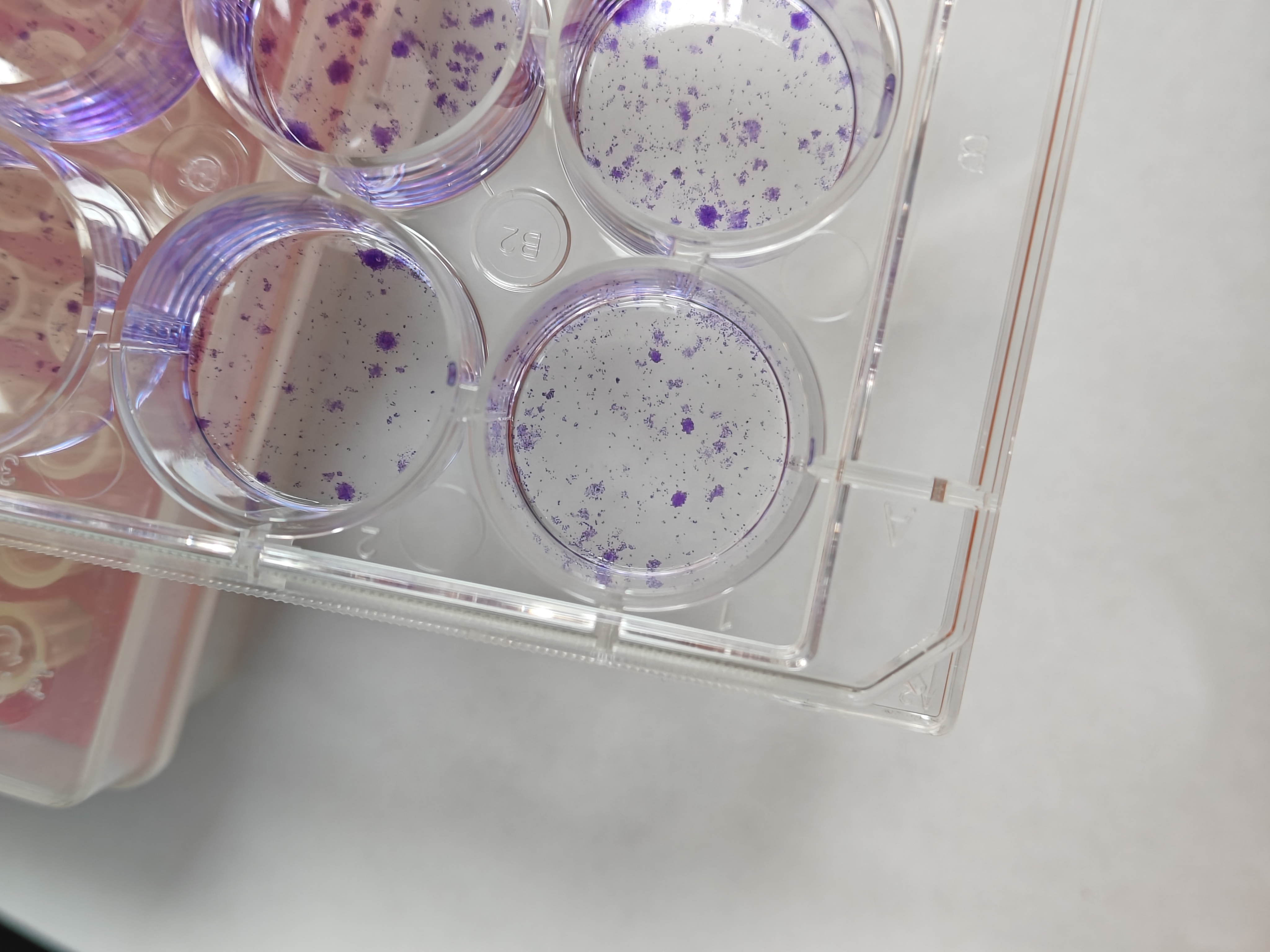

Supplement: Supplementary file 16 — Figure EV6 Source Data [file 44321_2026_460_MOESM16_ESM.zip › Source data Figure EV6/FIG EV6E/WT(PAMP).jpg]

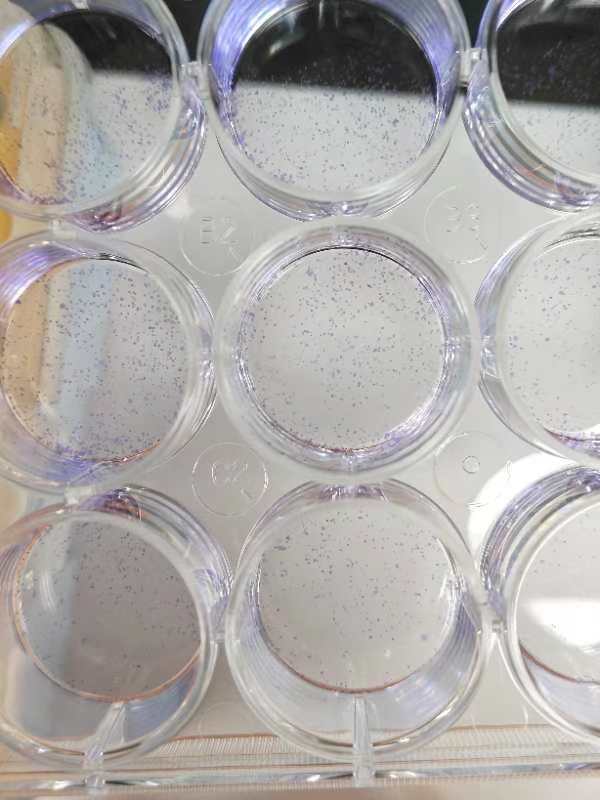

Supplement: Supplementary file 16 — Figure EV6 Source Data [file 44321_2026_460_MOESM16_ESM.zip › Source data Figure EV6/FIG EV6F/PAMP(F16A)+PYCR1(N123A).jpg]

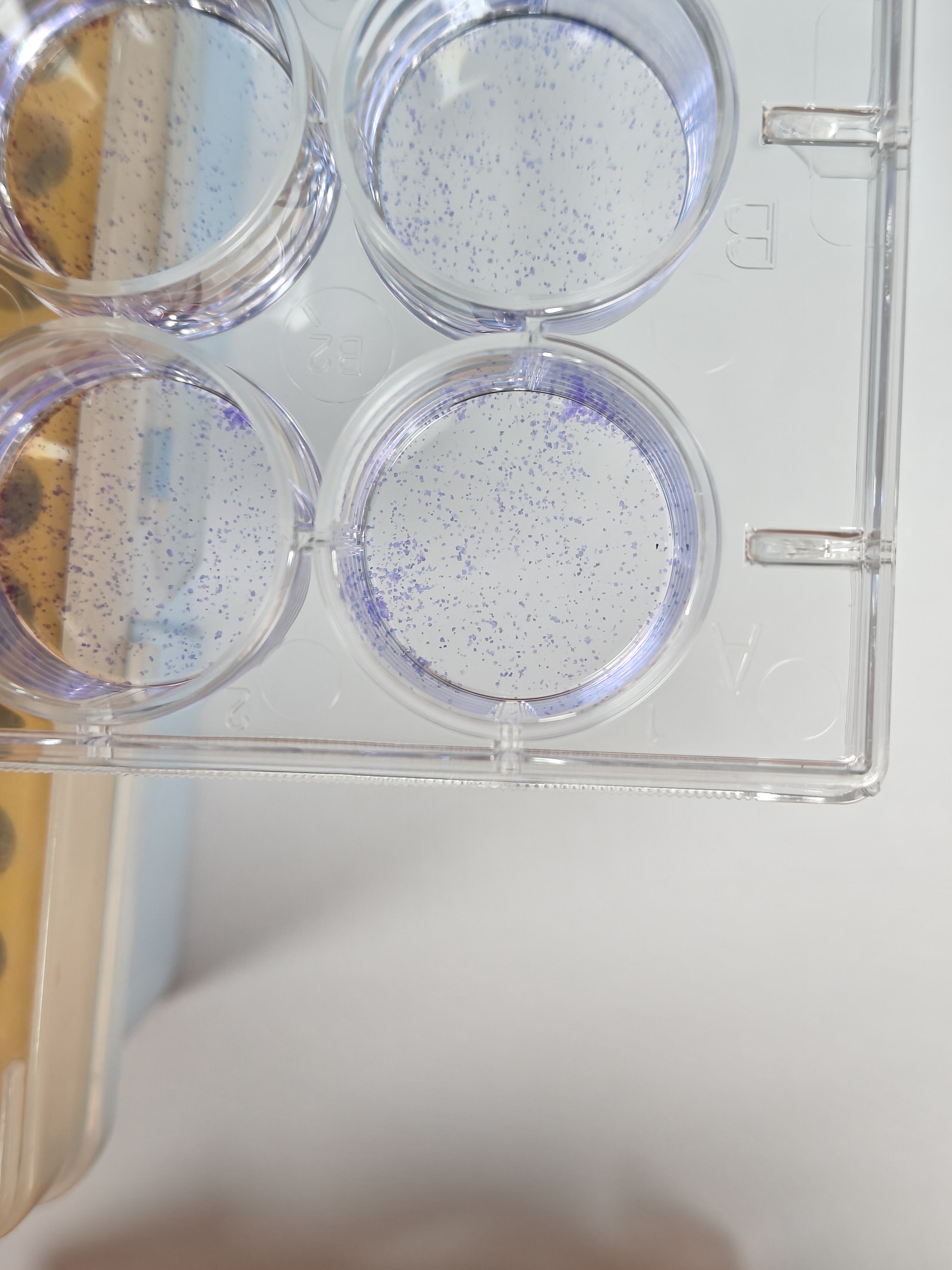

Supplement: Supplementary file 16 — Figure EV6 Source Data [file 44321_2026_460_MOESM16_ESM.zip › Source data Figure EV6/FIG EV6F/PAMP(F16A)+PYCR1.jpg]

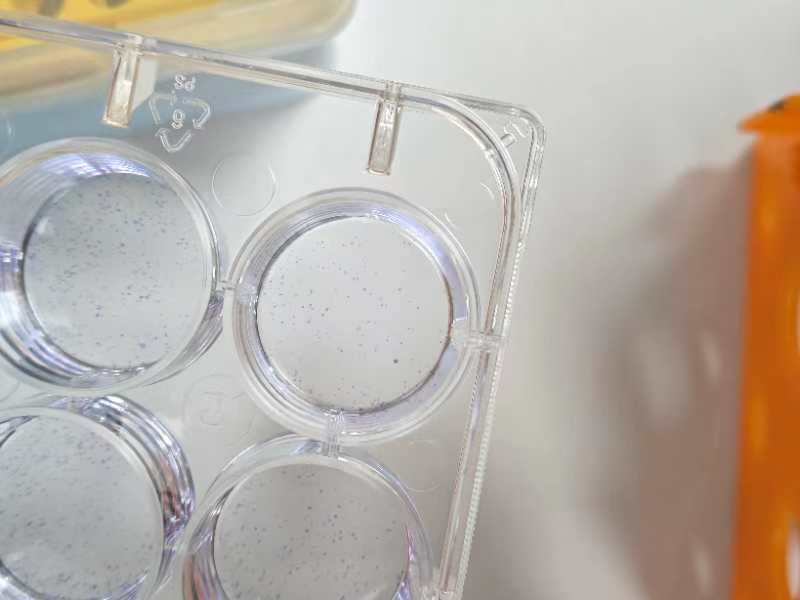

Supplement: Supplementary file 16 — Figure EV6 Source Data [file 44321_2026_460_MOESM16_ESM.zip › Source data Figure EV6/FIG EV6F/PAMP+PYCR1(N123A).jpg]

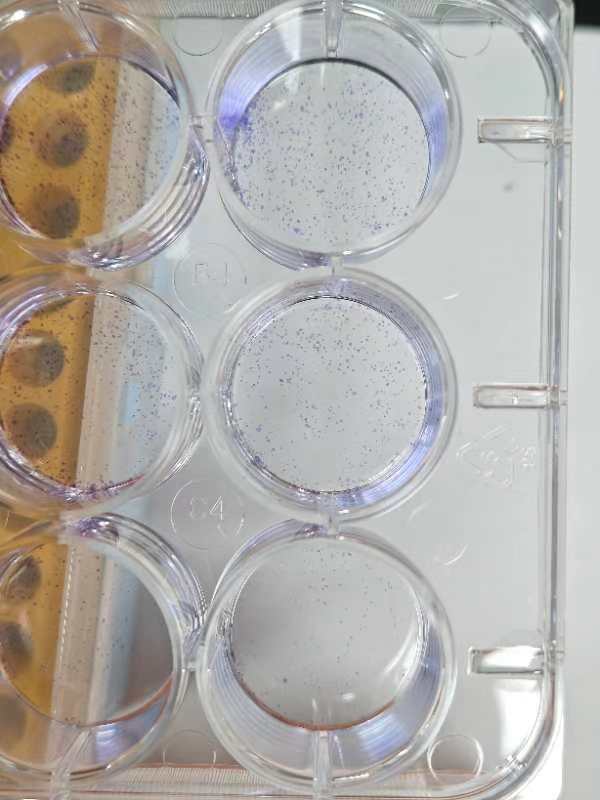

Supplement: Supplementary file 16 — Figure EV6 Source Data [file 44321_2026_460_MOESM16_ESM.zip › Source data Figure EV6/FIG EV6F/PAMP+PYCR1.jpg]

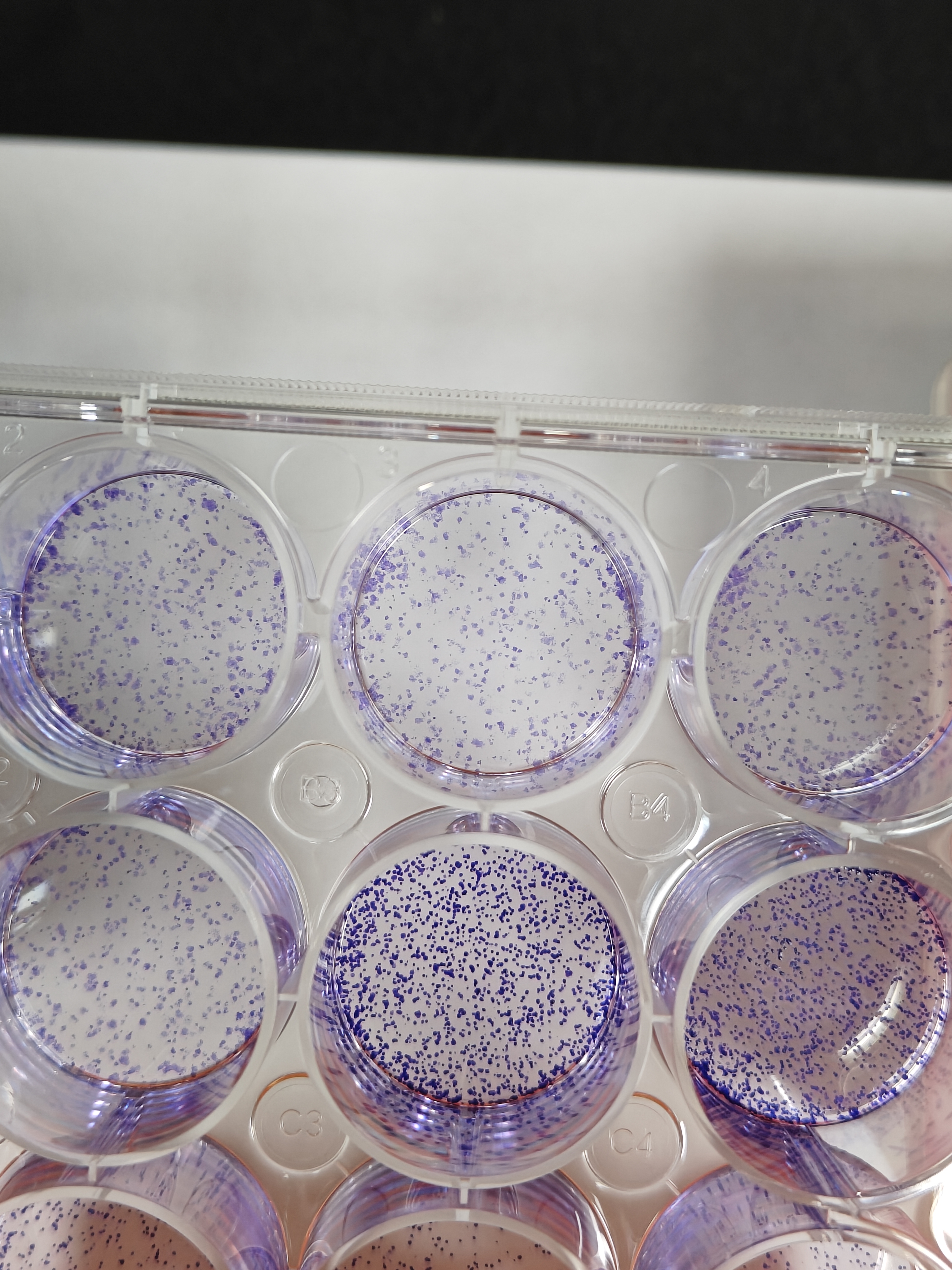

Supplement: Supplementary file 17 — Figure EV7 Source Data [file 44321_2026_460_MOESM17_ESM.zip › Source data Figure EV7/FIG EV7C/A549-100.jpg]

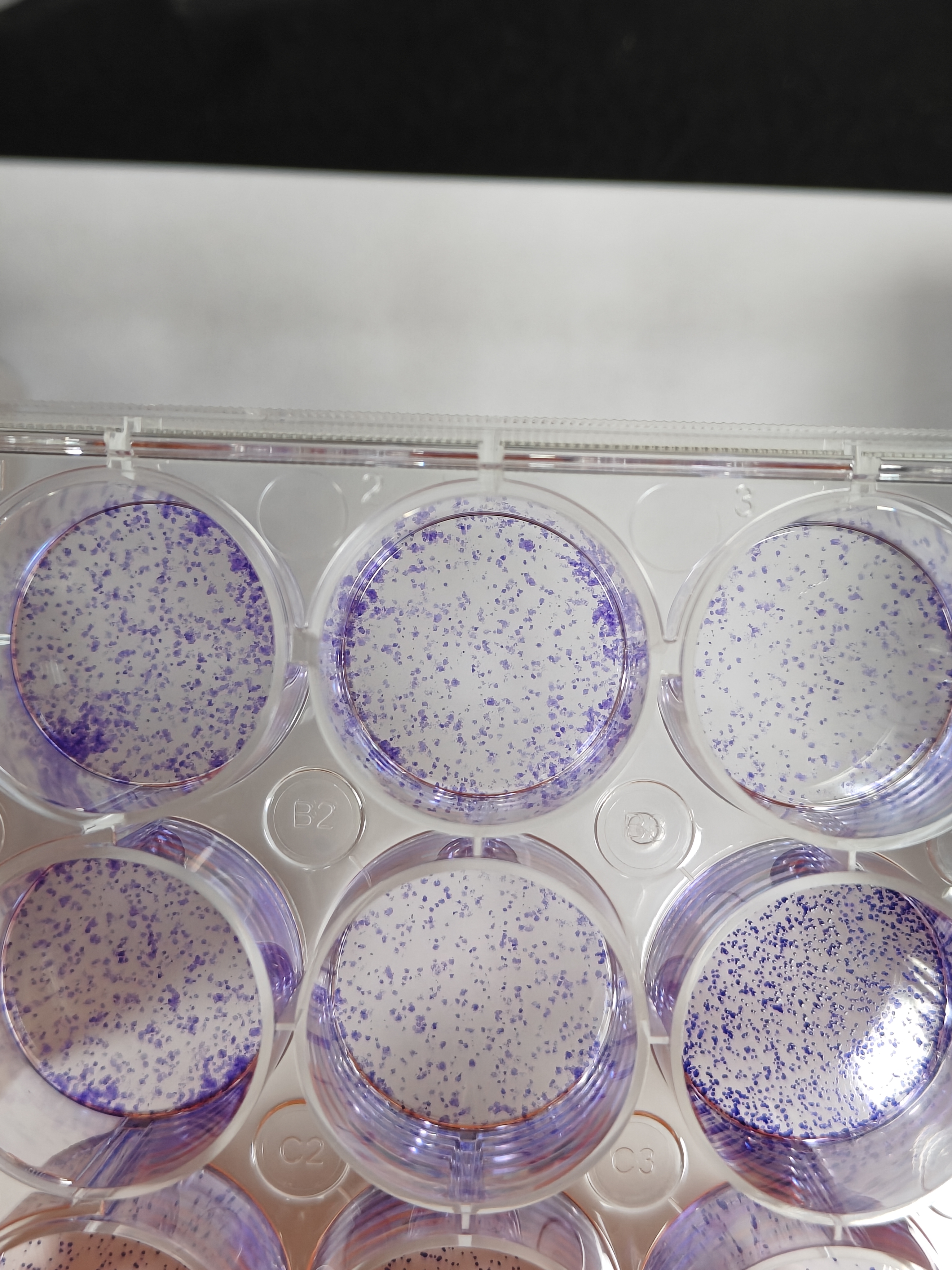

Supplement: Supplementary file 17 — Figure EV7 Source Data [file 44321_2026_460_MOESM17_ESM.zip › Source data Figure EV7/FIG EV7C/A549-12.5.jpg]

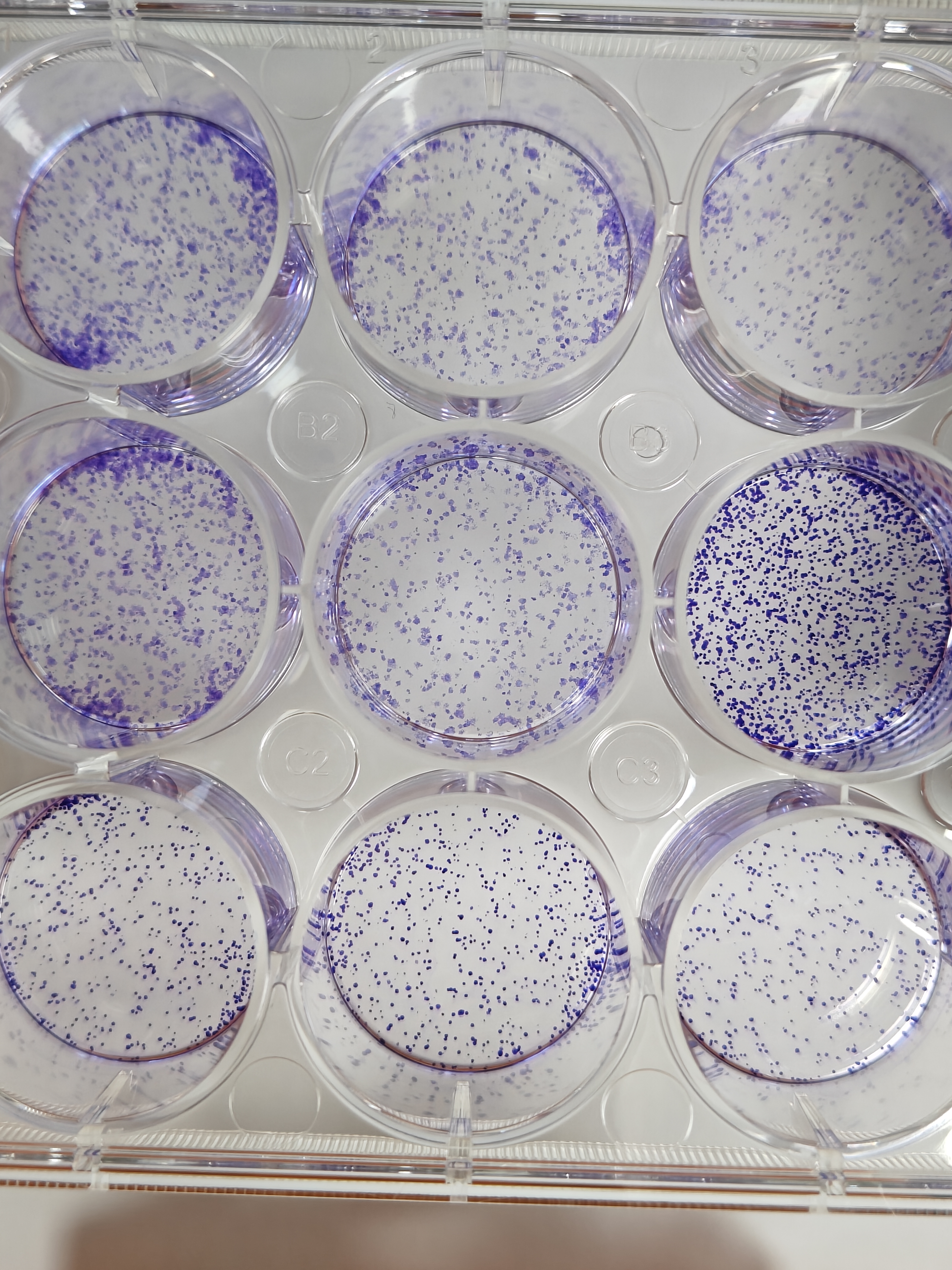

Supplement: Supplementary file 17 — Figure EV7 Source Data [file 44321_2026_460_MOESM17_ESM.zip › Source data Figure EV7/FIG EV7C/A549-25.jpg]

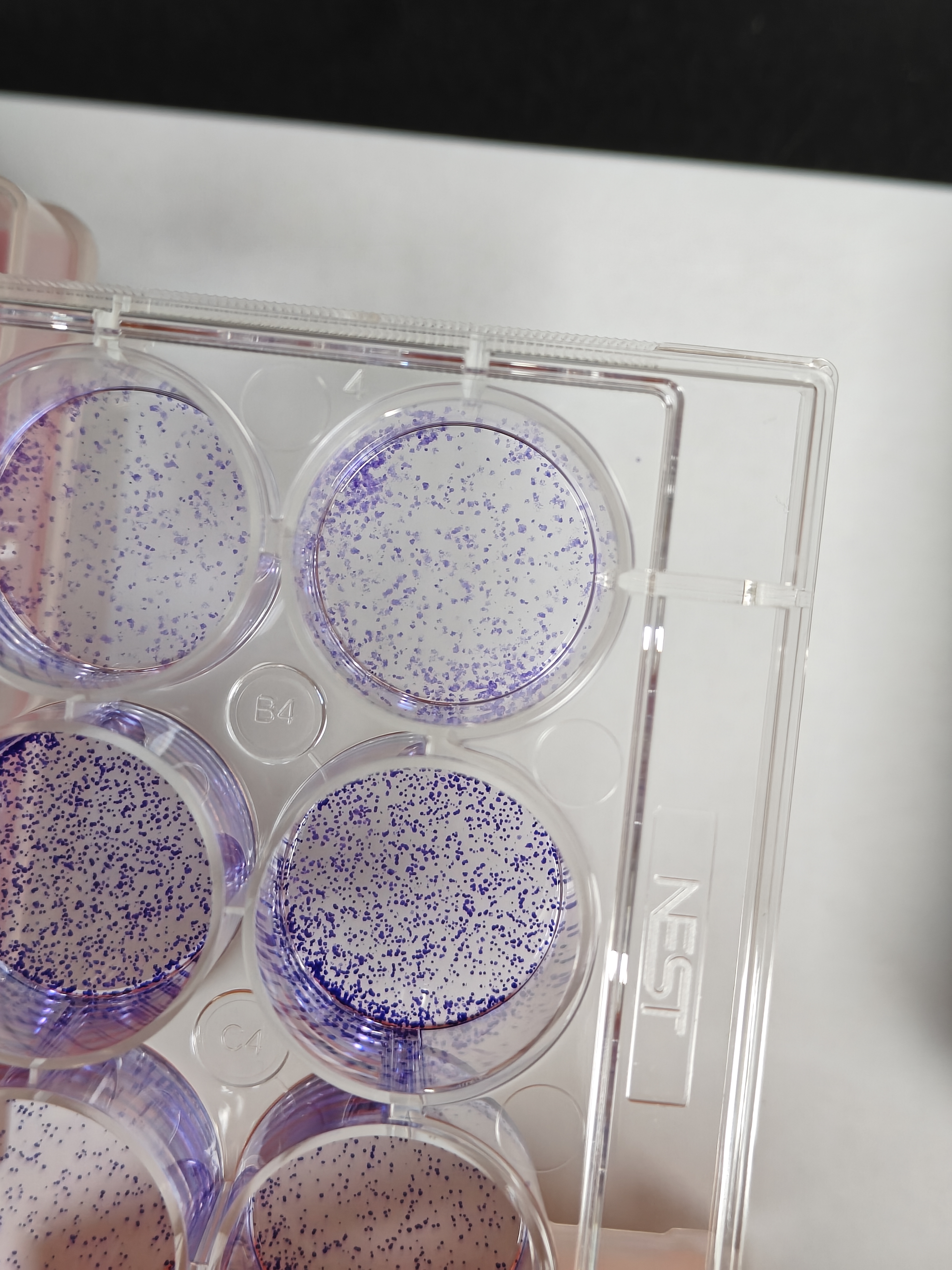

Supplement: Supplementary file 17 — Figure EV7 Source Data [file 44321_2026_460_MOESM17_ESM.zip › Source data Figure EV7/FIG EV7C/A549-50.jpg]

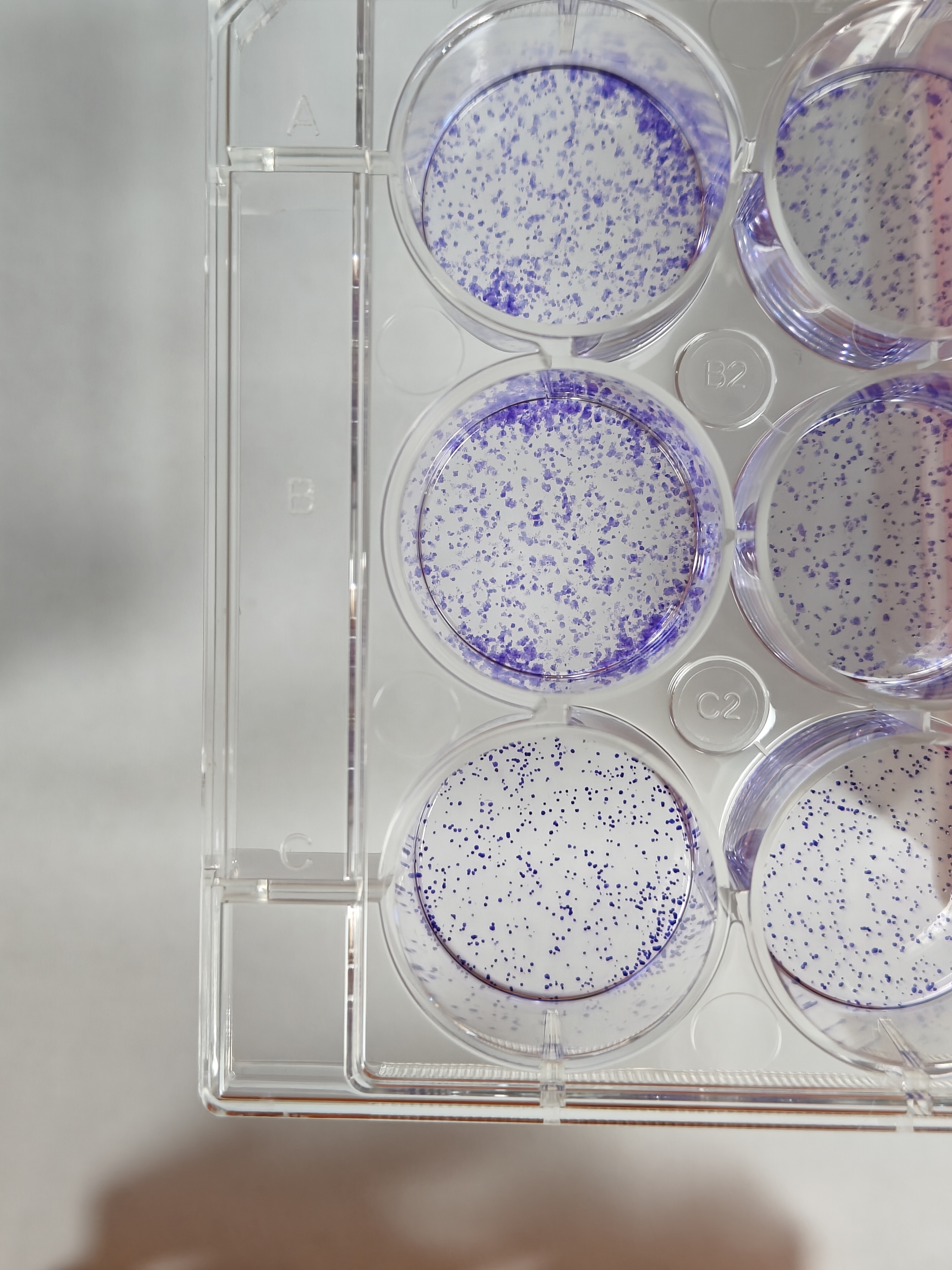

Supplement: Supplementary file 17 — Figure EV7 Source Data [file 44321_2026_460_MOESM17_ESM.zip › Source data Figure EV7/FIG EV7C/A549-6.25.jpg]

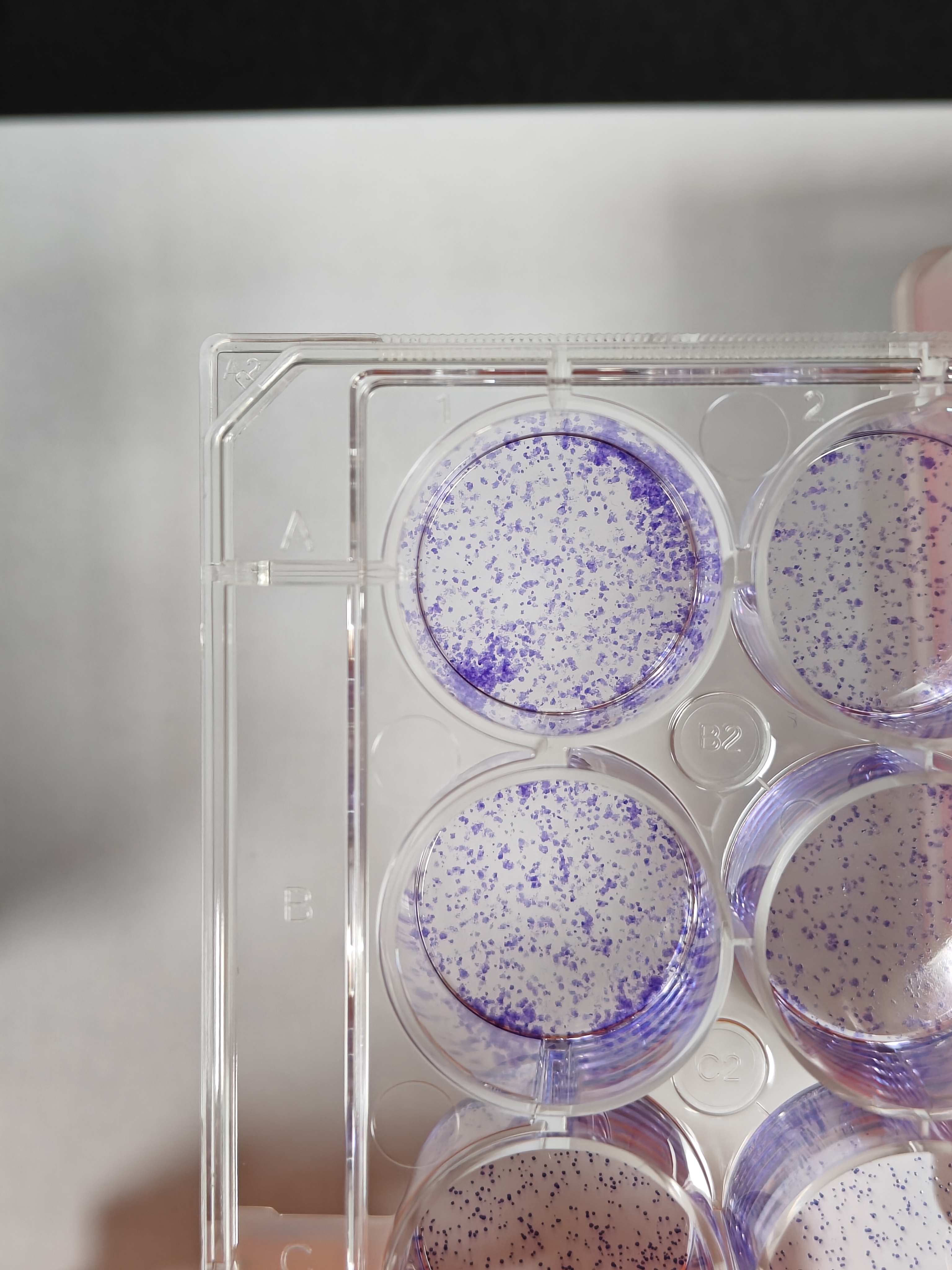

Supplement: Supplementary file 17 — Figure EV7 Source Data [file 44321_2026_460_MOESM17_ESM.zip › Source data Figure EV7/FIG EV7C/A549-C.jpg]

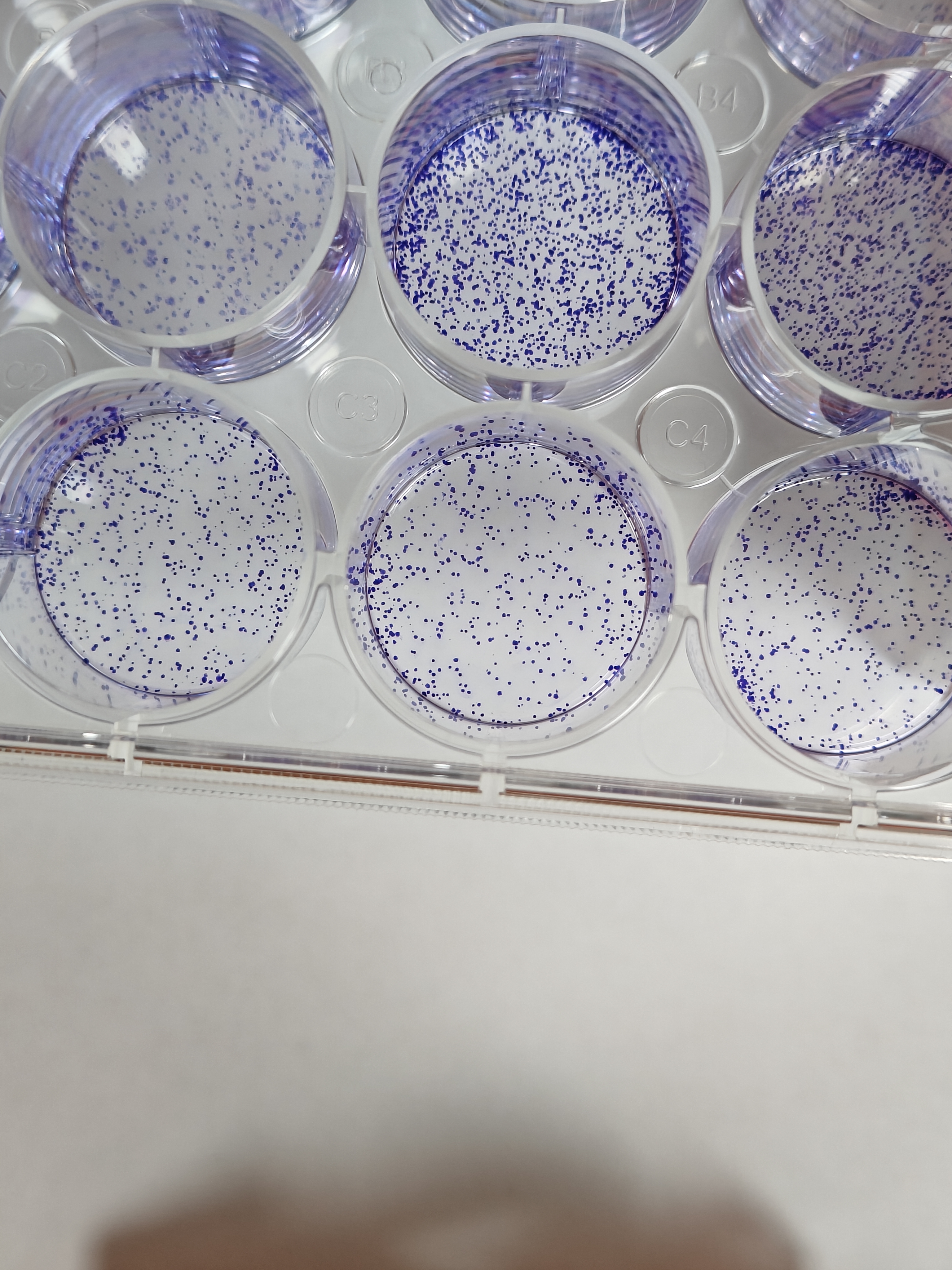

Supplement: Supplementary file 17 — Figure EV7 Source Data [file 44321_2026_460_MOESM17_ESM.zip › Source data Figure EV7/FIG EV7C/H460-100.jpg]

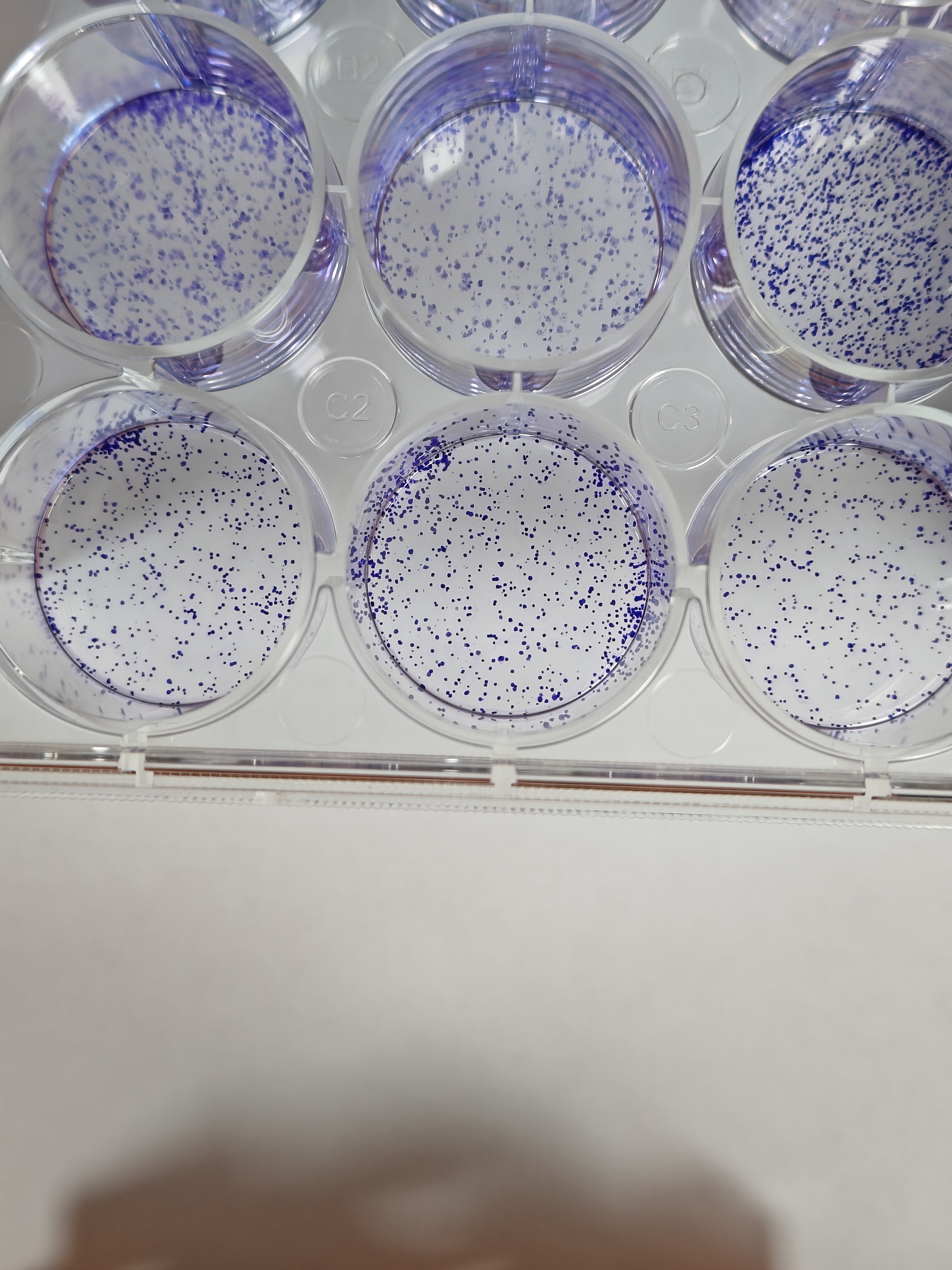

Supplement: Supplementary file 17 — Figure EV7 Source Data [file 44321_2026_460_MOESM17_ESM.zip › Source data Figure EV7/FIG EV7C/H460-12.5.jpg]

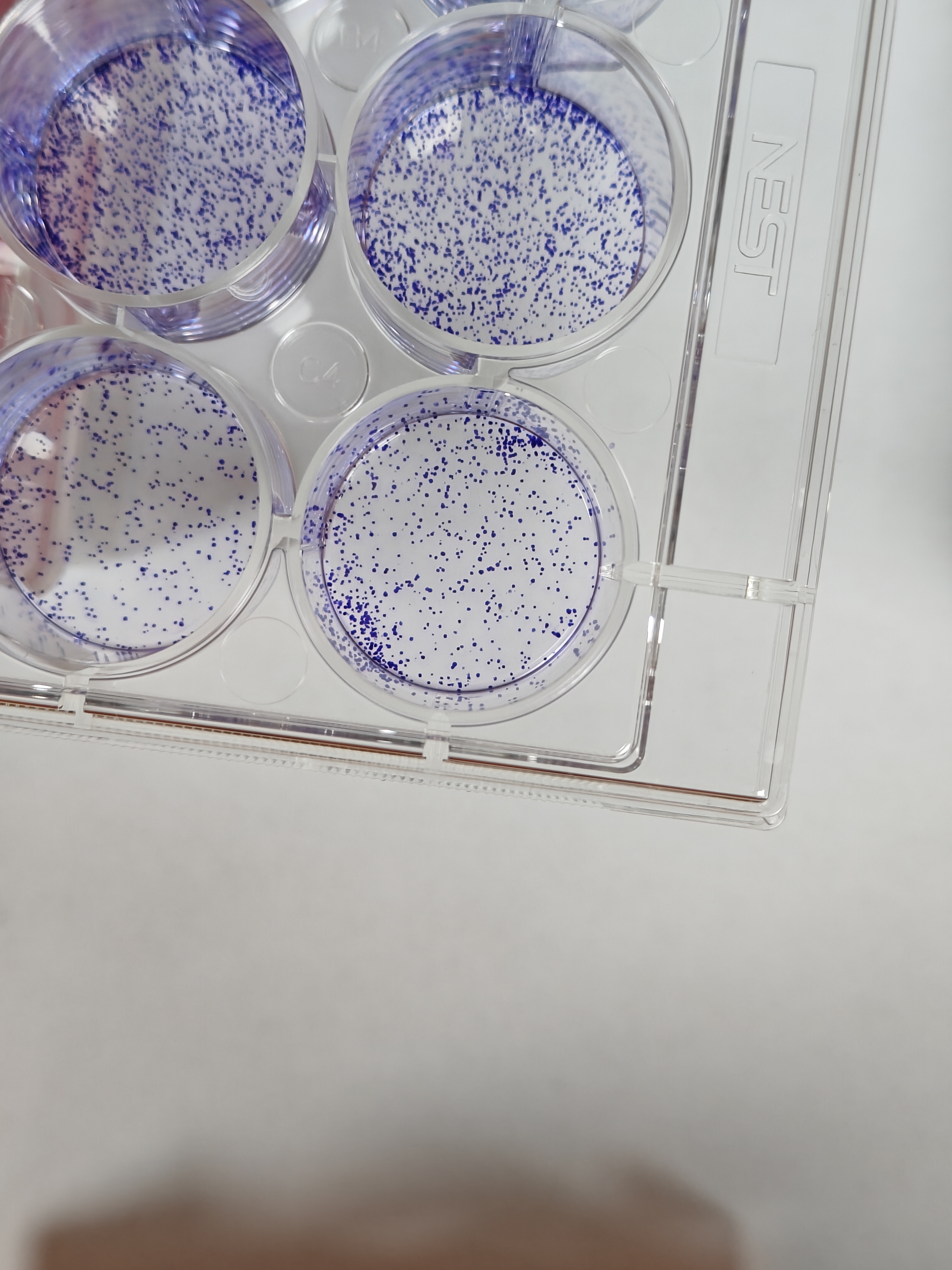

Supplement: Supplementary file 17 — Figure EV7 Source Data [file 44321_2026_460_MOESM17_ESM.zip › Source data Figure EV7/FIG EV7C/H460-25.jpg]

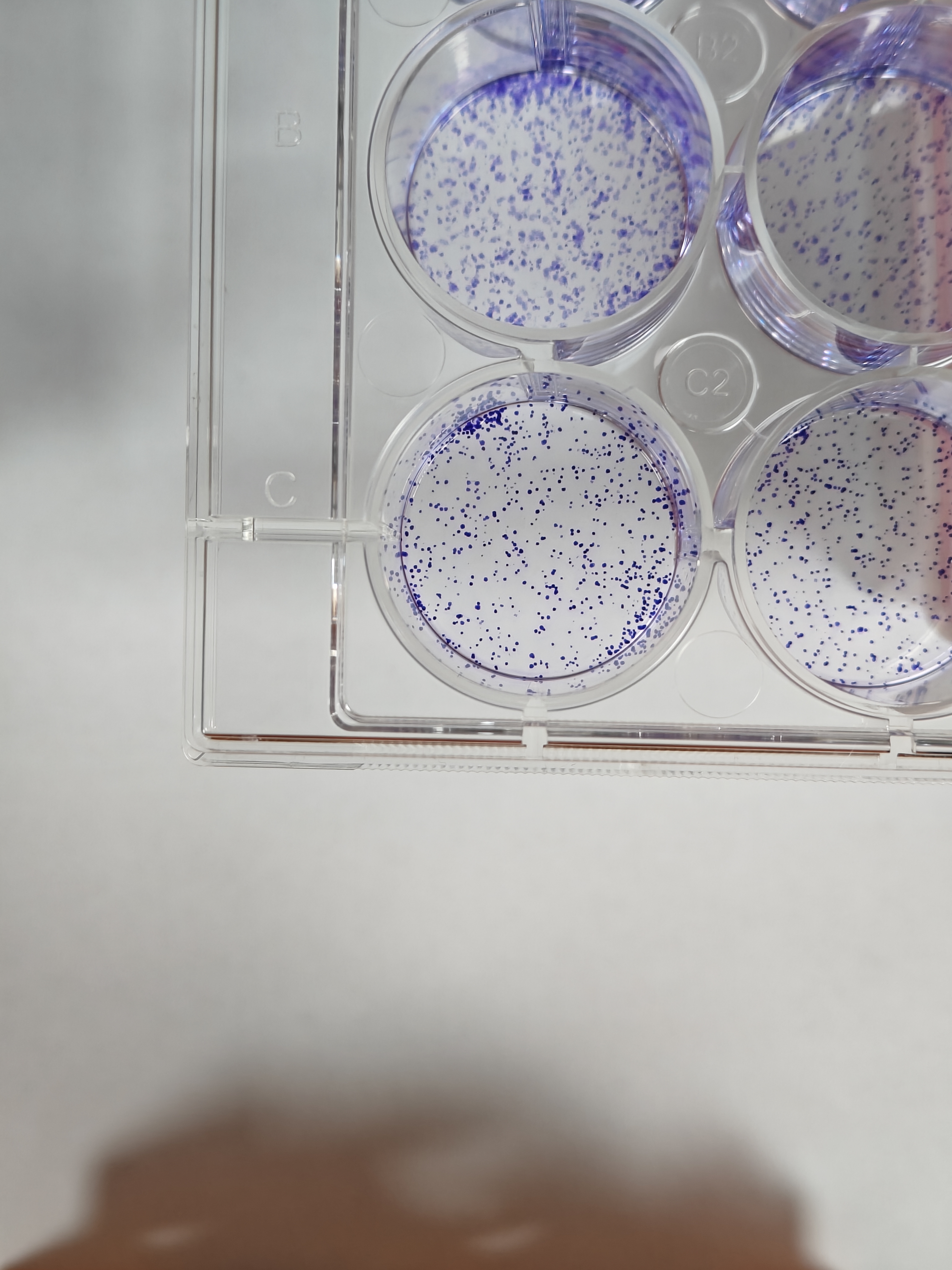

Supplement: Supplementary file 17 — Figure EV7 Source Data [file 44321_2026_460_MOESM17_ESM.zip › Source data Figure EV7/FIG EV7C/H460-50.jpg]

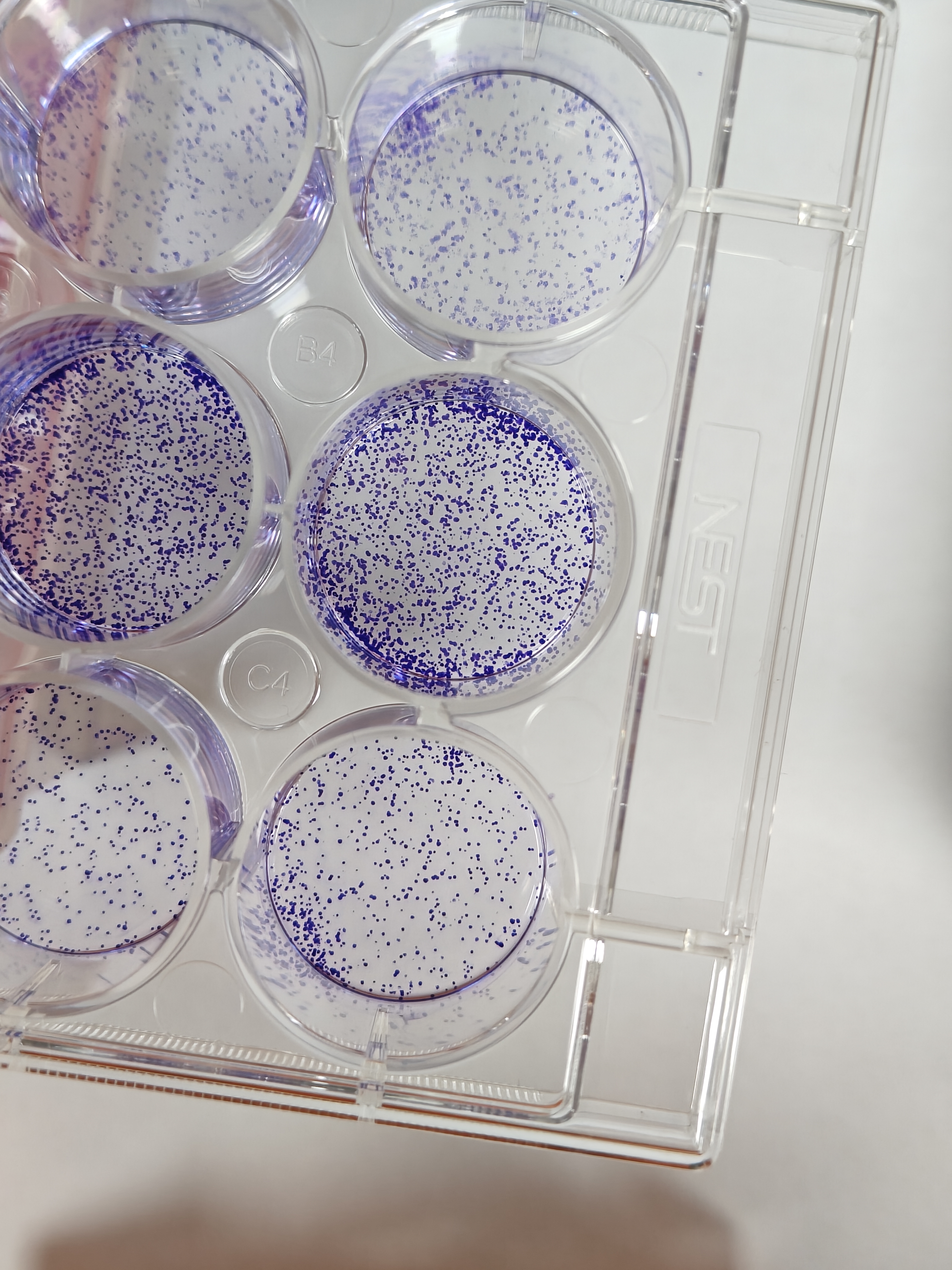

Supplement: Supplementary file 17 — Figure EV7 Source Data [file 44321_2026_460_MOESM17_ESM.zip › Source data Figure EV7/FIG EV7C/H460-6.25.jpg]

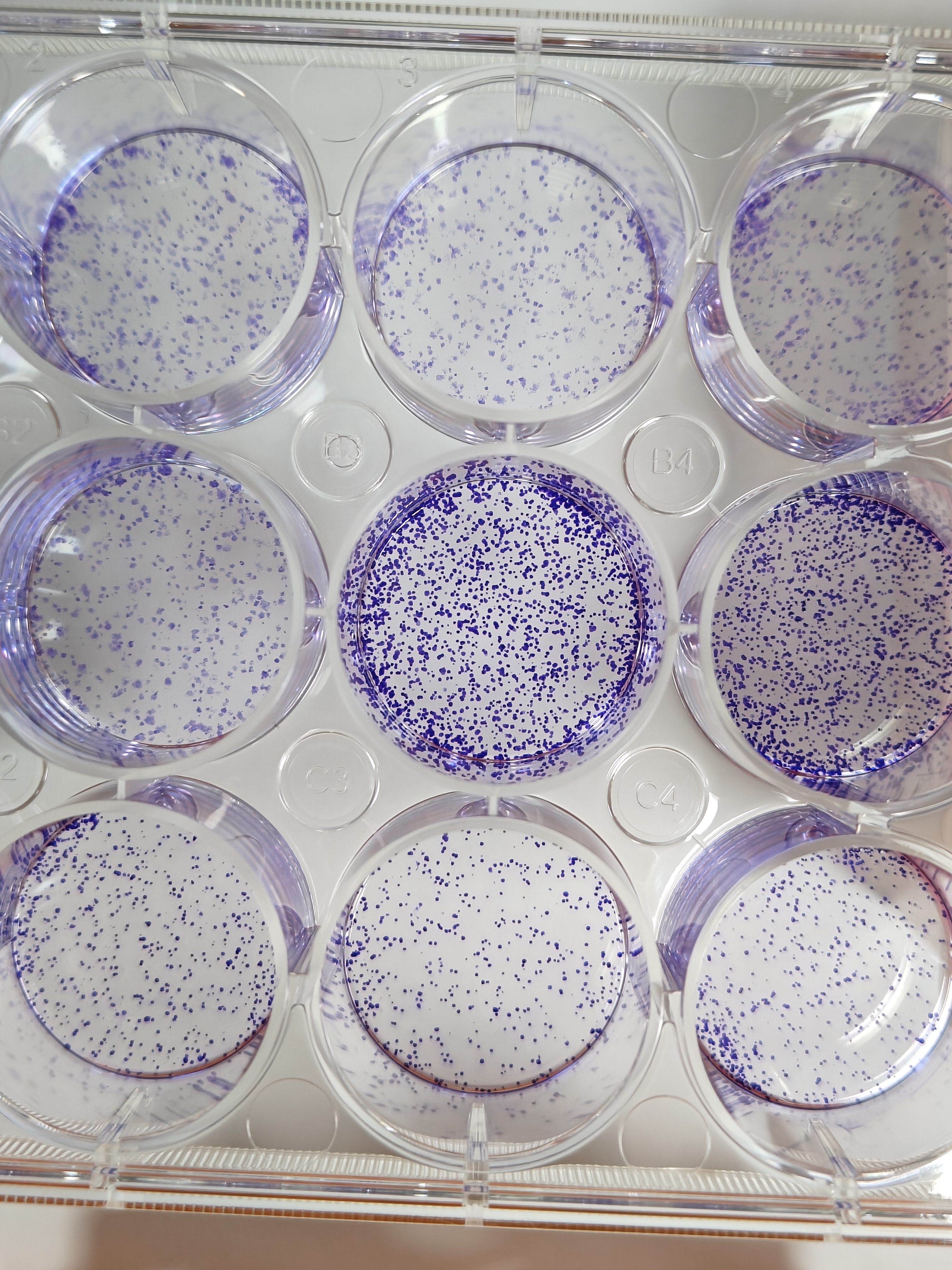

Supplement: Supplementary file 17 — Figure EV7 Source Data [file 44321_2026_460_MOESM17_ESM.zip › Source data Figure EV7/FIG EV7C/H460-C.jpg]

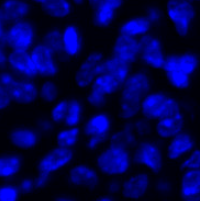

Supplement: Supplementary file 17 — Figure EV7 Source Data [file 44321_2026_460_MOESM17_ESM.zip › Source data Figure EV7/FIG EV7D/0.png]

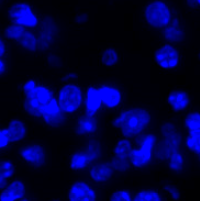

Supplement: Supplementary file 17 — Figure EV7 Source Data [file 44321_2026_460_MOESM17_ESM.zip › Source data Figure EV7/FIG EV7D/125.png]

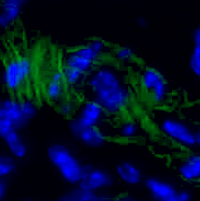

Supplement: Supplementary file 17 — Figure EV7 Source Data [file 44321_2026_460_MOESM17_ESM.zip › Source data Figure EV7/FIG EV7D/250.png]

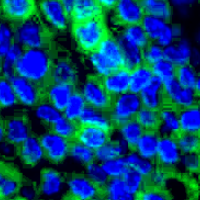

Supplement: Supplementary file 17 — Figure EV7 Source Data [file 44321_2026_460_MOESM17_ESM.zip › Source data Figure EV7/FIG EV7D/500.png]
