# Supplementary material for: PAMP orchestrates proline metabolic rewiring to suppress LUAD via PYCR1 inhibition
Source: EMBO Mol Med. 2026 Jun 9;18(7):2867–95. doi: 10.1038/s44321-026-00460-2 (PMC13365495; doi:10.1038/s44321-026-00460-2)
Supplement: Supplementary file 18 — Expanded View Figures [file 44321_2026_460_MOESM18_ESM.pdf]

Expanded View Figures

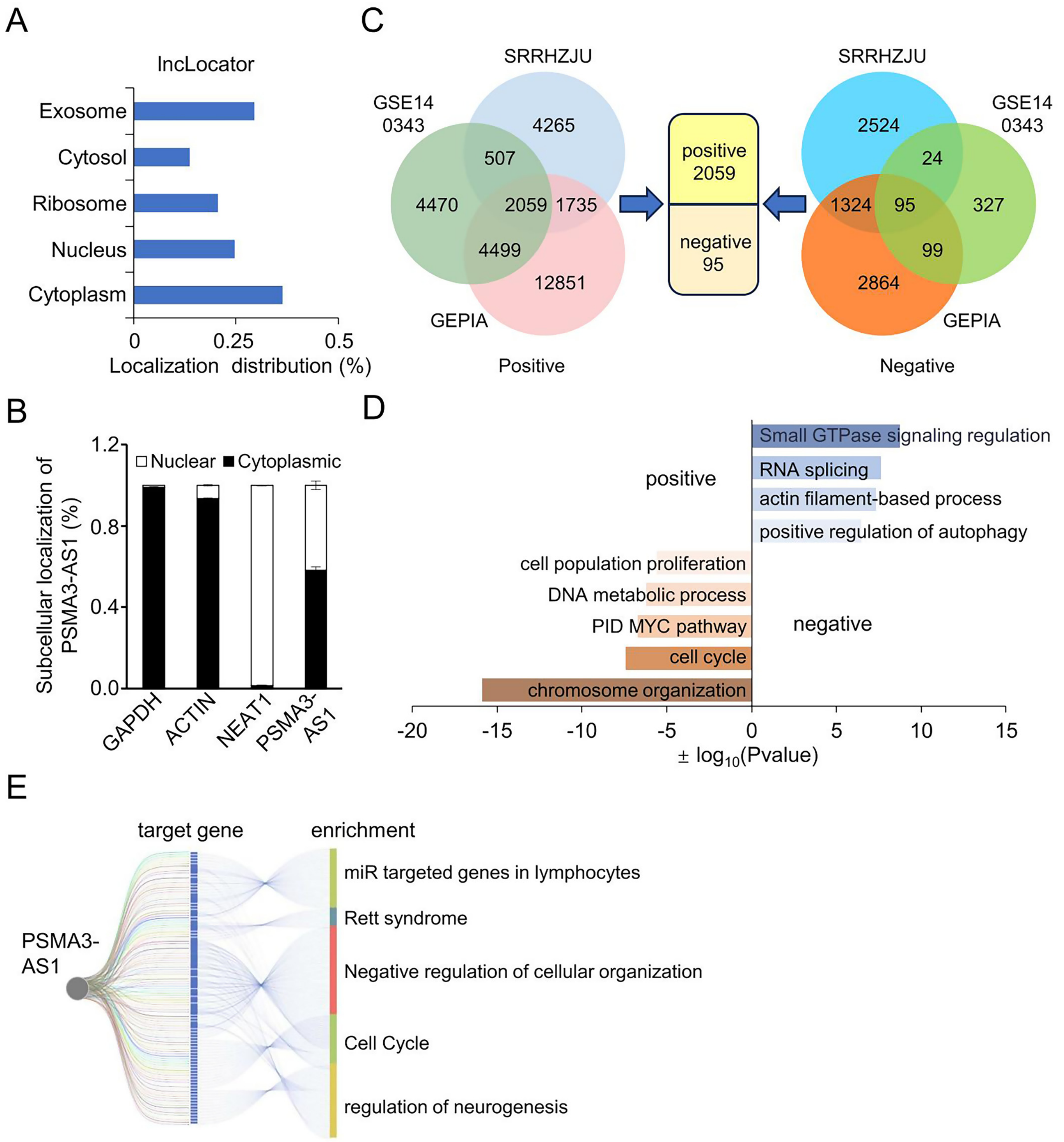

Figure EV1. PSMA3-AS1 localizes to both the nucleus and cytoplasm and correlates with proliferation.

(**A**) Predicted subcellular localization of PSMA3-AS1 using lncLocator. (**B**) qPCR analysis of cytoplasmic or nuclear PSMA3-AS1 RNA levels in A549 cells. GAPDH and ACTIN served as cytoplasmic controls, while NEAT1 served as a nuclear control ($n = 3$). (**C**) The gene intersection results of PSMA3-AS1 correlation analysis based on three RNA-seq data (Up: genes positively correlated with PSMA3-AS1; Down: genes negatively correlated with PSMA3-AS1). (**D**) Enrichment analysis of signaling pathways and biological processes derived from PSMA3-AS1 correlation analysis. (**E**) Sankey diagram showing the enrichment analysis results for mRNA genes associated with PSMA3-AS1 in the WMDS.netL network. Data were shown as mean ± SEM in (**B**). The correlation analysis retained all the genes with a *P* value less than 0.05; statistical analysis was performed using Pearson correlation (**C**, **E**). Source data are available online for this figure.

 © The Author(s)

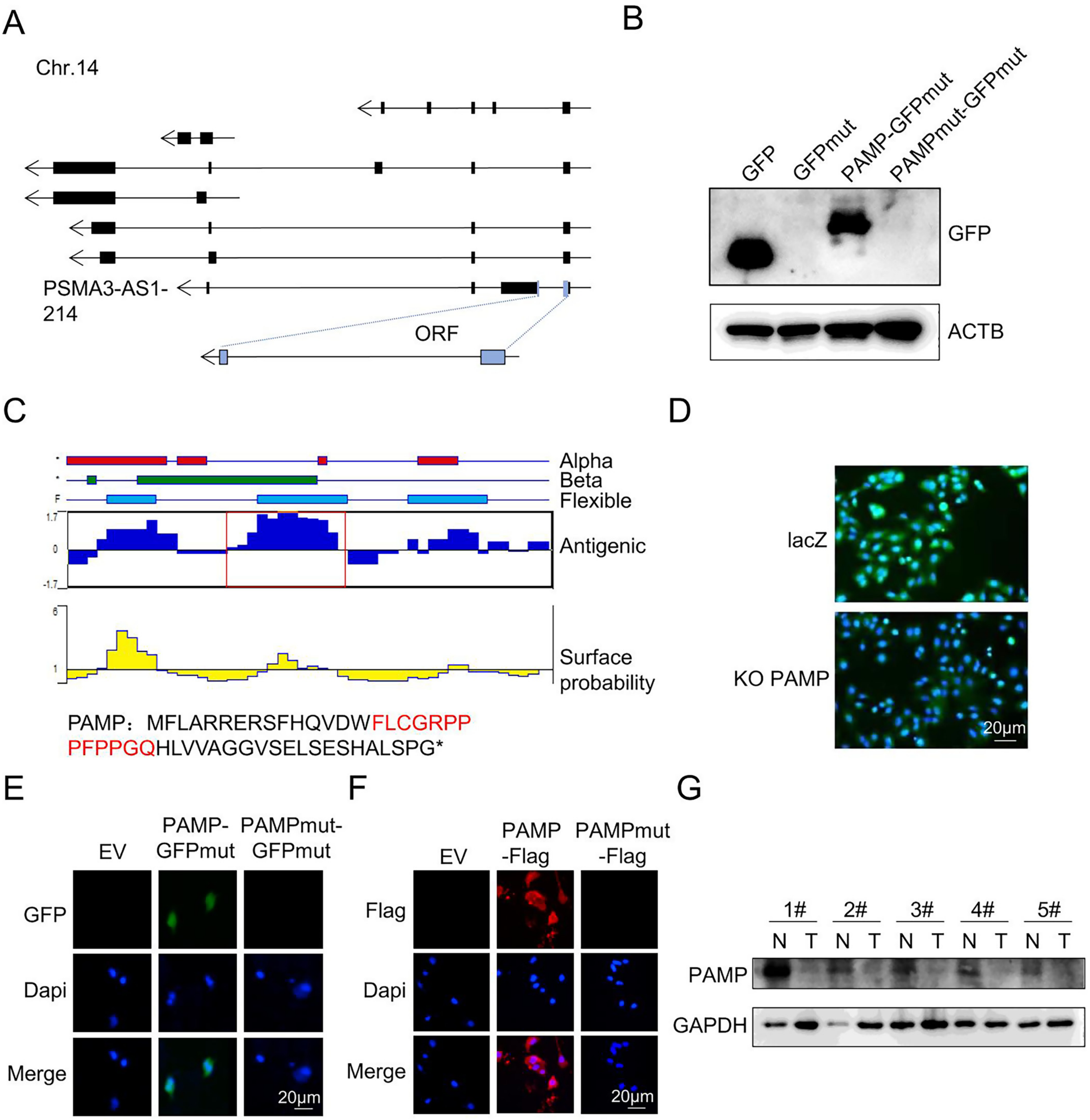

Figure EV2. Validation of PAMP translation, antibody specificity, and clinical relevance.

(**A**) Schematic representation of the genomic location of PSMA3-AS1. (**B**) Western blot analysis of fusion protein levels in A549 cells transfected with the indicated constructs for 24 h, using anti-GFP antibodies. (**C**) Prediction of the PAMP epitope using Protean software. The amino acid sequence highlighted in red was selected for polyclonal antibody preparation. (**D**) Immunostaining determined the PAMP antibody specificity by using KO-PAMP A549 cells. Nuclei were stained with DAPI (blue), Green color indicates the intensity of PAMP-specific signals. (**E**, **F**) Immunofluorescence detection of GFP and Flag signals in A549 cells transfected with the indicated constructs. Nuclei were stained with DAPI (blue). (**G**) Western blot analysis of PAMP peptide levels in an independent cohort of five pairs of LUAD tissues and matched adjacent normal tissues. Results were obtained from three independent experiments. Source data are available online for this figure.

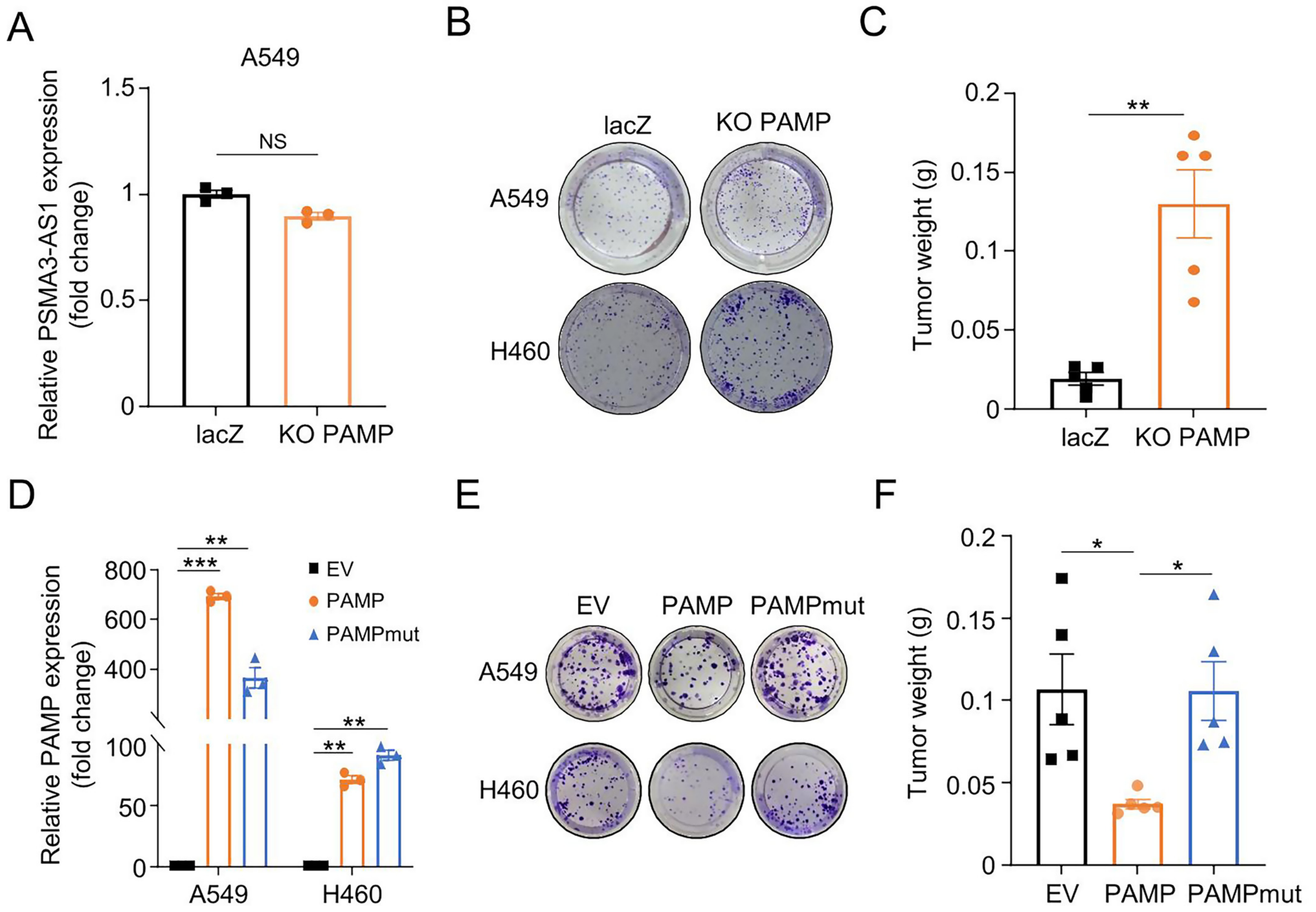

Figure EV3. PAMP functions as a tumor suppressor.

(**A**) qPCR analysis of PSAMA3-AS1 expression in A549 cells following PAMP knockout ($n = 3$). Primers were designed outside the PAMP region. Proline accumulation is presented as fold change relative to the LacZ control group, which was used as the reference condition and set to 1. (**B**) Colony formation assays assessing the impact of PAMP knockout on LUAD cell viability. (**C**) Quantification of tumor weight in xenograft mice from PAMP knockout and control mice ($n = 5$, $P = 0.001$). (**D**) qPCR analysis of PAMP RNA expression in LUAD cells overexpressing PAMP or PAMPmut constructs ($n = 3$, A549: P (EV vs PAMP) = 3.23E-04; P (PAMP vs PAMPmut) = 0.01; H460: P (EV vs PAMP) = 0.001; P (PAMP vs PAMPmut) = 0.002). Proline accumulation is presented as fold change relative to the EV control group, which was used as the reference condition and set to 1. (**E**) Colony formation assays to assess cell proliferation in LUAD cells overexpressing wild-type PAMP or PAMPmut. (**F**) Quantification of tumor weight in xenograft mice from EV, wild-type PAMP and PAMPmut mice ($n = 5$, P (EV vs PAMP) = 0.031; P (PAMP vs PAMPmut) = 0.017). Data were shown as mean ± SEM in (**C**, **D**, **F**). $^{*}P < 0.05$; $^{**}P < 0.01$; $^{***}P < 0.001$; two-tailed unpaired t-test. Source data are available online for this figure.

© The Author(s)

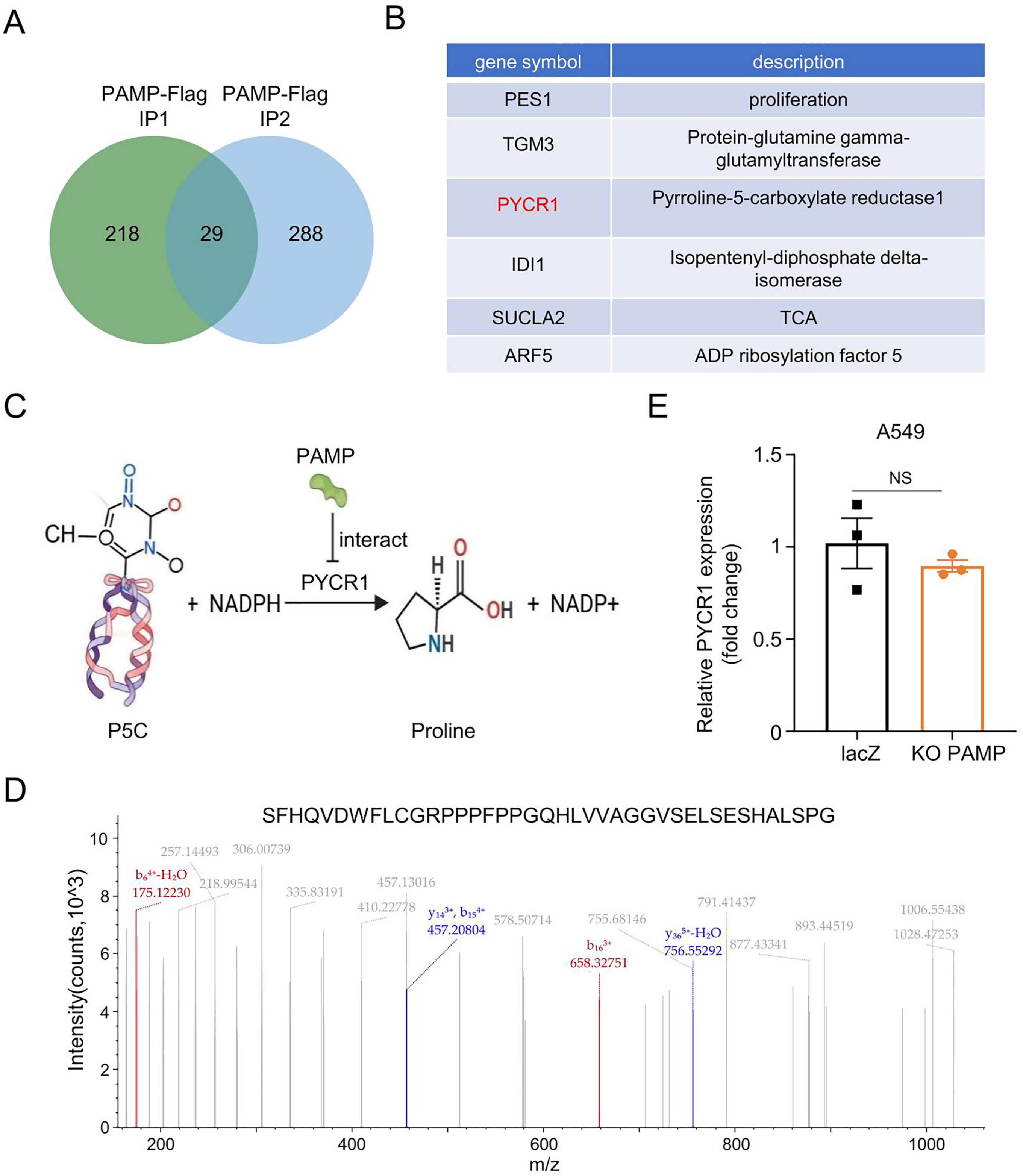

gene symbol	description
PES1	proliferation
TGM3	Protein-glutamine gamma-glutamyltransferase
PYCR1	Pyrroline-5-carboxylate reductase1
IDI1	Isopentenyl-diphosphate delta-isomerase
SUCLA2	TCA
ARF5	ADP ribosylation factor 5

◀ **Figure EV4. PYCR1 is a key interacting protein of PAMP.**

(**A**) Venn diagram comparing results from two independent Co-IP experiments. (**B**) Proteins identified by mass spectrometry analysis of Co-IP fractions. (**C**) Schematic illustrating the PYCR1-catalyzed reduction of P5C to proline, which is coupled with the oxidation of NADPH to $NADP^+$. The interaction of the regulatory factor PAMP with PYCR1 is depicted. (**D**) Endogenous PAMP-specific peptide segments enriched by Co-IP identified via mass spectrometry analysis. (**E**) qPCR analysis of PYCR1 expression in A549 cells following PAMP knockout ($n = 3$). Proline accumulation is presented as fold change relative to the LacZ control group, which was used as the reference condition and set to 1. Data were shown as mean ± SEM in (**E**). A two-tailed unpaired *t*-test was performed, with $P < 0.05$ considered statistically significant. Source data are available online for this figure.

 © The Author(s)

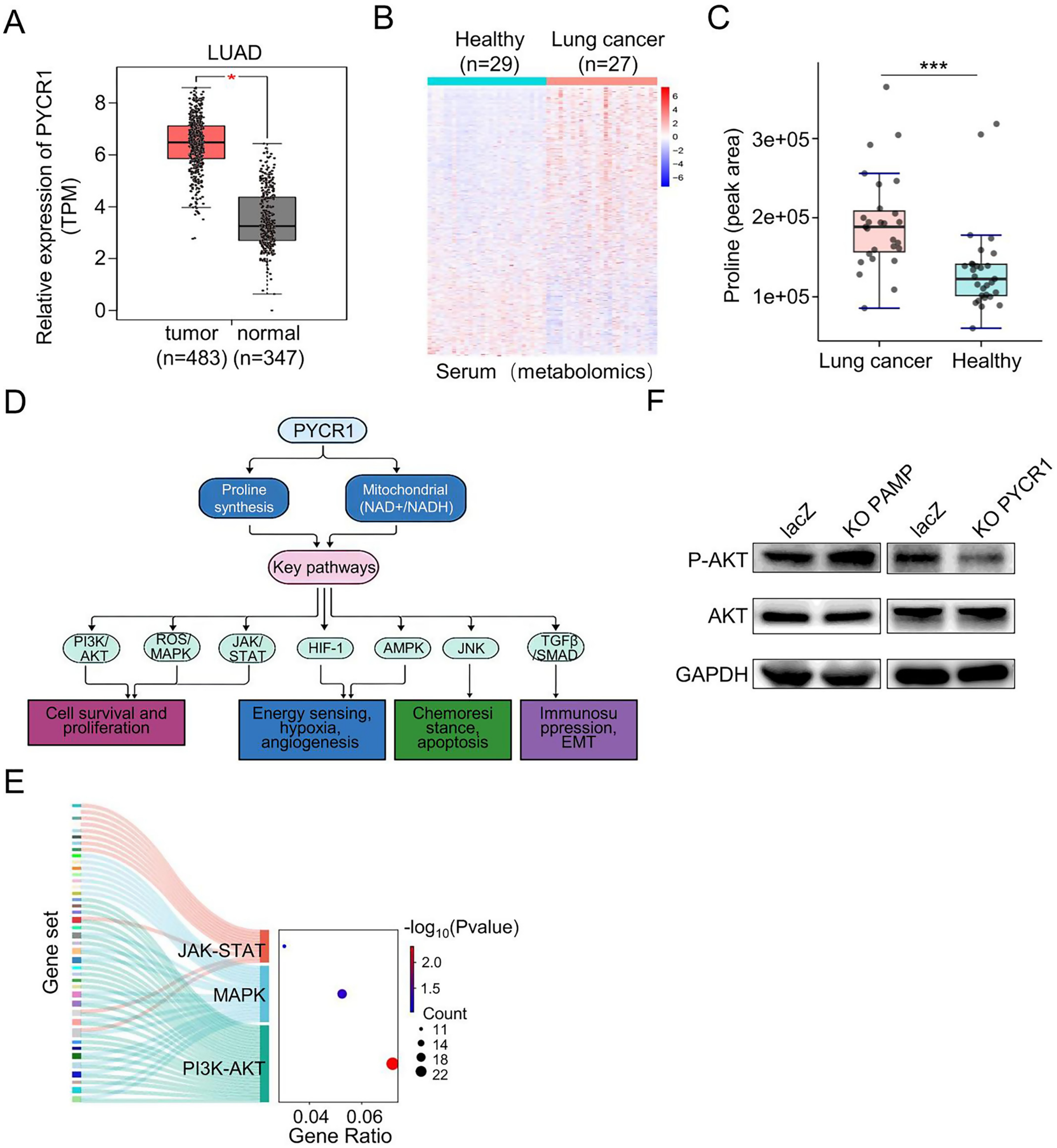
A
LUAD
Relative expression of PYCR1 (TPM)
tumor (n=483)
normal (n=347)
B
Healthy (n=29)
Lung cancer (n=27)
Serum (metabolomics)
C
***
Proline (peak area)
3e+05
2e+05
1e+05
Lung cancer
Healthy
D
PYCR1
Proline synthesis
Mitochondrial (NAD+/NADH)
Key pathways
PI3K/AKT
ROS/MAPK
JAK/STAT
HIF-1
AMPK
JNK
TGFβ/SMAD
Cell survival and proliferation
Energy sensing, hypoxia, angiogenesis
Chemoresistance, apoptosis
Immunosuppression, EMT
E
Gene set
JAK-STAT
MAPK
PI3K-AKT
-log10(Pvalue)
2.0
1.5
Count
11
14
18
22
0.04
0.06
Gene Ratio
F
lacZ
KO PAMP
lacZ
KO PYCR1
P-AKT
AKT
GAPDH

Figure EV5. PAMP-PYCR1 axis regulates AKT phosphorylation.

(**A**) Relative expression of PYCR1 in LUAD tissues and adjacent normal tissues from the TCGA cohort ($P = 8.7E\text{-}36$). (**B**) Metabolic heatmap showing alterations in plasma samples from lung cancer patients compared with healthy individuals. The color bar indicates log2 fold change. (**C**) Comparison of plasma proline levels between lung cancer patients and healthy individuals ($P = 8.72E\text{-}4$). (**D**) Schematic of PYCR1-centered metabolic and functional network. PYCR1 integrates signals from serum metabolomic features and regulates proline synthesis as well as mitochondrial NAD+/NADH homeostasis. These core functions converge on key pathways, subsequently driving diverse downstream biological processes including cell survival, proliferation, stress responses, and immunosuppression. (**E**) GSEA indicates that PAMP is significantly associated with the PI3K-Akt, JAK-STAT, and MAPK pathways, which are known regulators of cell proliferation as annotated in the SMPDB shown in (**D**). (**F**) Western blot analysis of phosphorylated AKT in lacZ, KO-PAMP, and KO PYCR1 cells. GAPDH serves as the loading control. Data are shown as mean ± SEM in (**C**). $^{***}P < 0.001$; two-tailed unpaired *t*-test. Box plots (**A**, **C**) show the median as the center line, the interquartile range (IQR; 25th–75th percentiles) as the box bounds, and whiskers extending to the minimum and maximum values within 1.5 × IQR. Source data are available online for this figure.

 © The Author(s)

A

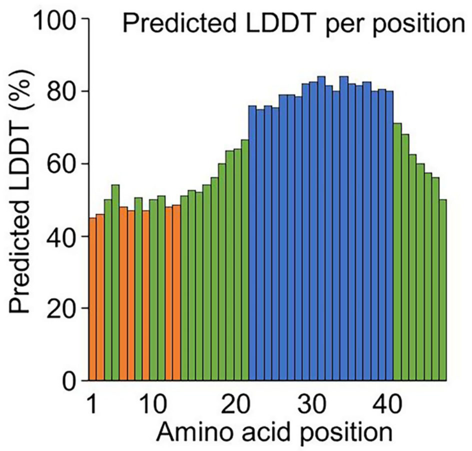

C

Protein Variation Effect Analyzer

PYCR1 residue_ alter (P32322)	PAMP dockig site	Provean		SIFT	
		score	Prediction(cutoff=-2.5)	score	Prediction(cutoff=-0.05)
T122A	L17	-1.56	neutral	0.014	damaging
N123A	F16	-7.99	deleterious	0.001	damaging
R200A	H42,A43	-1.6	neutral	0.655	tolerated
Q208A	A32	-5.2	deleterious	0.001	damaging

B

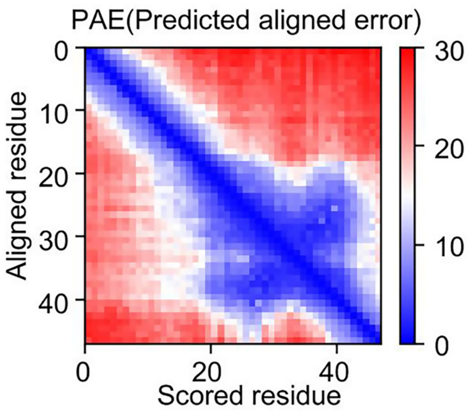

D E

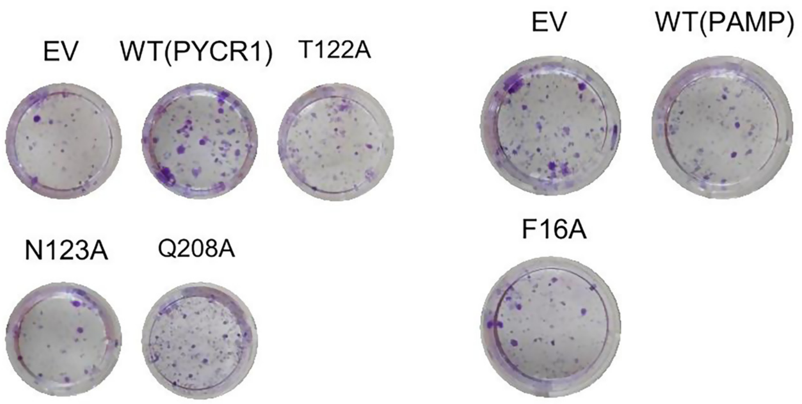

F

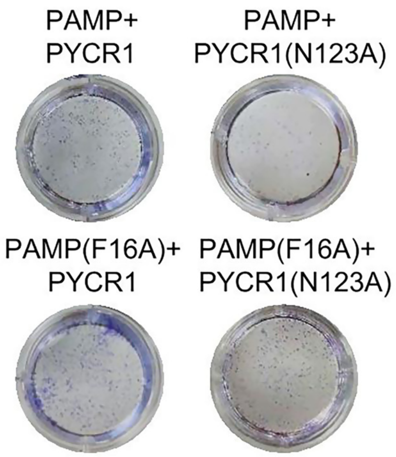

© The Author(s)

Figure EV6. PAMP-F16 and PYCR1-N123 binding sites are critical for LUAD proliferation.

(**A**) Residue-based pLDDT profile for the top AlphaFold2 models of PAMP conformations, derived from AlphaFold2 predictions. The structural model assessment distributions for the PAMP conformational ensemble come from PAMP-MSA shallow subsampling predictions. pLDDT, a local prediction score by AlphaFold2, assesses the reliability of residue geometry predictions. Scores range from 0 to 100, with higher values indicating more confidence: 90–100 (high precision), 70–90 (credible), 50–70 (low precision), and <50 (error). (**B**) Heatmaps illustrating the predicted alignment error (PAE) between residue pairs in the top-ranked model. The color scale highlights contrast between high-confidence and low-confidence regions. (**C**) Evaluation of amino acid variations at PYCR1 docking sites using Provean and SIFT. (**D**) Colony formation assays assessing cell proliferation in PAMP-mutated A549 cells. (**E**) Colony formation assays evaluating cell proliferation in PYCR1-mutated A549 cells. (**F**) Colony formation assays evaluating cell proliferation in cells co-expressing wild-type PAMP or binding-defective PAMP(F16A) with either wild-type PYCR1 or catalytically inactive PYCR1(N123A). Source data are available online for this figure.

 © The Author(s)

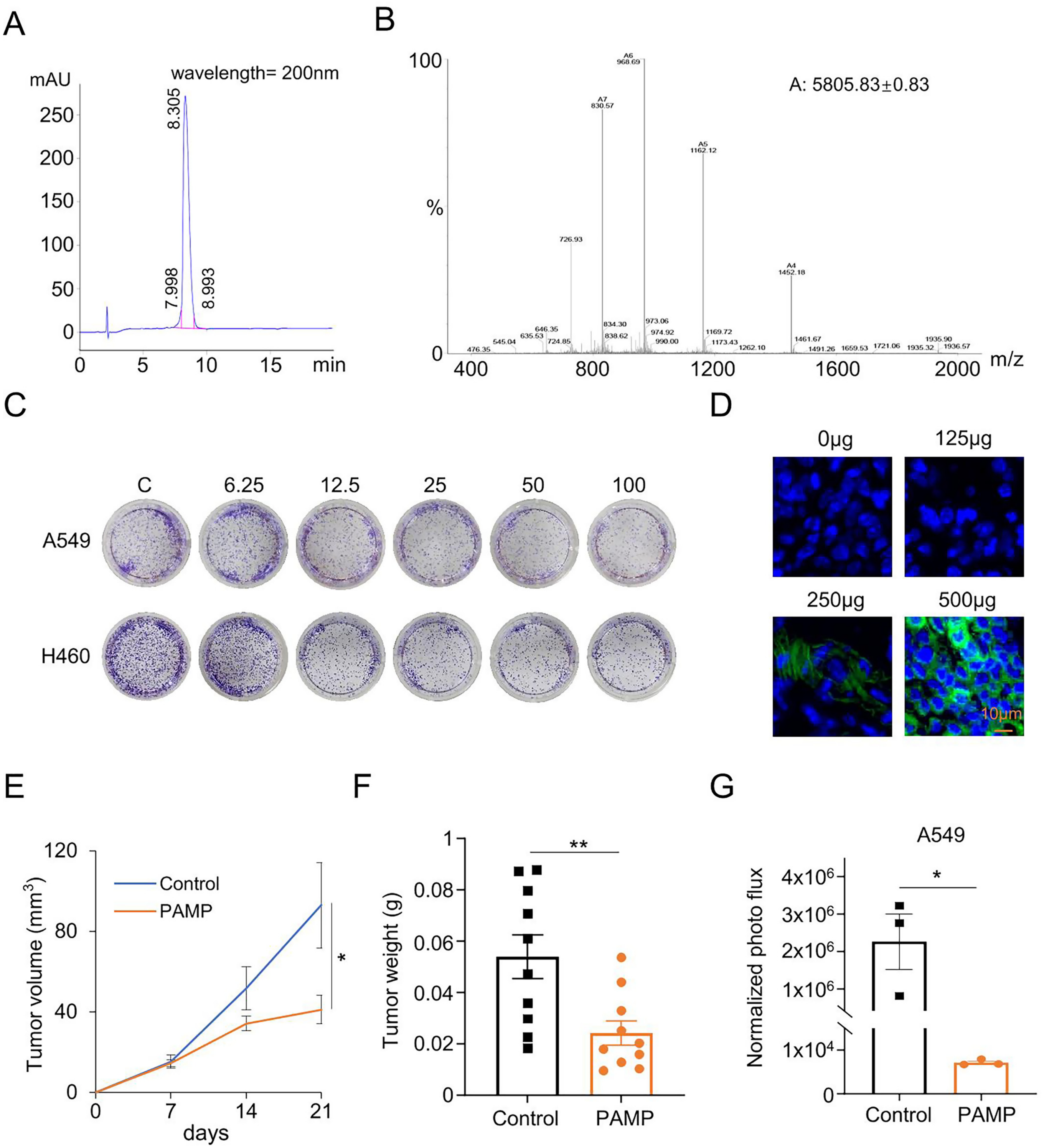

Figure EV7. Synthetic PAMP inhibits LUAD growth.

(**A**, **B**) Purity and molecular weight analysis of chemically synthesized PAMP peptide using HPLC (**A**) and LC-MS (**B**). (**C**) Colony formation assays evaluating cell proliferation in LUAD cells treated with synthetic PAMP. (**D**) FITC-labeled synthetic PAMP was injected into the peritoneal cavity of mice, and then FITC fluorescence in the lung tissue was detected using a fluorescence microscope; nuclei were stained with DAPI (blue). (**E**, **F**) Tumor growth was monitored every 7 days to generate a growth curve, and tumor weight was measured after surgical excision ($n = 5$, **E**: $P = 0.048$; **F**: $P = 0.036$). (**G**) Bioluminescence imaging was used to quantify lung colonization in PAMP-treated and control nude mice ($n = 3$, $P = 0.038$). Data were shown as mean ± SEM in (**E–G**). $^{*}P < 0.05$; two-tailed unpaired *t*-test. Source data are available online for this figure.
