# Supplementary material for: PAMP orchestrates proline metabolic rewiring to suppress LUAD via PYCR1 inhibition
Source: EMBO Mol Med. 2026 Jun 9;18(7):2867–95. doi: 10.1038/s44321-026-00460-2 (PMC13365495; doi:10.1038/s44321-026-00460-2)
Supplement: Supplementary file 3 — Peer Review File [file 44321_2026_460_MOESM3_ESM.pdf]

EMBO Molecular Medicine

PAMP orchestrates proline metabolic rewiring to suppress LUAD via PYCR1 inhibition

Yun Ma, Jiani Yi, Chengcai Zheng, Juze Yang, Jia Li, Mengqian Yu, Xinyi Qian, Jiayi Ren, Xiaofan Wu, Yan Lu, and Pengyuan Liu

Corresponding authors: Pengyuan Liu (pyliu@zju.edu.cn) , Yan Lu (yanlu76@zju.edu.cn)

Review Timeline:

Submission Date:	24th Sep 25
Editorial Decision:	30th Oct 25
Revision Received:	7th Apr 26
Editorial Decision:	29th Apr 26
Revision Received:	19th May 26
Accepted:	27th May 26

Editor: Lise Roth

Transaction Report:

(Note: With the exception of the correction of typographical or spelling errors that could be a source of ambiguity, letters and reports are not edited. Depending on transfer agreements, referee reports obtained elsewhere may or may not be included in this compilation. Referee reports are anonymous unless the Referee chooses to sign their reports.)

30th Oct 2025

Dear Dr. Liu,

Thank you for submitting your manuscript to EMBO Molecular Medicine and please accept my apologies for the delay in getting back to you as we were waiting for a referee report. Unfortunately, referee #1 has not yet got back to us, but given that both referees #2 and #3 make similar recommendations, we prefer to make a decision now to avoid further delay in the process. Should referee #1 provide a report, we will send it to you on the understanding that we will not ask you to carry out any extensive experiments beyond those requested in the attached reports from referees #2 and #3. As you will see from the reports below, the referees recognize the interest of the study and generally support publication of your work, subject to appropriate revisions.

Addressing the reviewers' concerns in full will be necessary for further considering the manuscript in our journal, and acceptance of the manuscript will entail a second round of review. EMBO Molecular Medicine encourages a single round of revision only and therefore, acceptance or rejection of the manuscript will depend on the completeness of your responses included in the next, final version of the manuscript. For this reason, and to save you frustration at the end, I would strongly discourage you from returning an incomplete revision.

If you would like to discuss further the points raised by the referees, I am available to do so via email or video. Let me know if you are interested in this option.

We are expecting your revised manuscript within three to four months, if you anticipate any delay, please contact us.

When submitting your revised manuscript, please carefully review the instructions that follow below. We perform an initial quality control of all revised manuscripts before re-review; failure to include requested items will delay the evaluation of your revision.

We require:

1) A .docx formatted version of the manuscript text (including legends for main figures, EV figures and tables). Please make sure that the changes are highlighted to be clearly visible.

2) Individual production quality figure files as .eps, .tif, .jpg (one file per figure). For guidance, download the 'Figure Guide PDF' (https://www.embopress.org/page/journal/17574684/authorguide#figureformat).

3) At EMBO Press we ask authors to provide source data for the main figures. Our source data coordinator will contact you to discuss which figure panels we would need source data for and will also provide you with helpful tips on how to upload and organize the files.

4) A .docx formatted letter INCLUDING the reviewers' reports and your detailed point-by-point responses to their comments. As part of the EMBO Press transparent editorial process, the point-by-point response is part of the Review Process File (RPF), which will be published alongside your paper.

5) A complete author checklist, which you can download from our author guidelines (https://www.embopress.org/page/journal/17574684/authorguide#submissionofrevisions). Please insert information in the checklist that is also reflected in the manuscript. The completed author checklist will also be part of the RPF.

6) All Materials and Methods need to be described in the main text using our 'Structured Methods' format. According to this format, the Methods section includes a Reagents and Tools Table (listing key reagents, experimental models, software and relevant equipment and including their sources and relevant identifiers) followed by a Methods and Protocols section describing the methods, ideally using a step-by-step protocol format. The aim is to facilitate adoption of the methodologies across labs. Please download and fill our Reagents and Tools Table template (.docx), which you can find in our author guidelines: https://www.embopress.org/page/journal/14693178/authorguide#structuredmethods.
When submitting your revised manuscript, please do not include the Reagents and Tools Table in the Methods section of the manuscript but upload it as a separate file choosing the file type "Reagent Table".
An example of a Method paper with Structured Methods can be found here: https://www.embopress.org/doi/10.15252/msb.20178071

7) Please note that all corresponding authors are required to supply an ORCID ID for their name upon submission of a revised manuscript.

8) It is mandatory to include a 'Data Availability' section after the Materials and Methods. Before submitting your revision, primary

datasets produced in this study need to be deposited in an appropriate public database, and the accession numbers and database listed under 'Data Availability'. Please remember to provide a reviewer password if the datasets are not yet public (see https://www.embopress.org/page/journal/17574684/authorguide#dataavailability).

In case you have no data that requires deposition in a public database, please state so in this section. Note that the Data Availability Section is restricted to new primary data that are part of this study.

9) For data quantification: please specify the name of the statistical test used to generate error bars and P values, the number (n) of independent experiments (specify technical or biological replicates) underlying each data point and the test used to calculate p-values in each figure legend. The figure legends should contain a basic description of n, P and the test applied. Graphs must include a description of the bars and the error bars (s.d., s.e.m.). Please provide exact p values.

10) Our journal encourages inclusion of *data citations in the reference list* to directly cite datasets that were re-used and obtained from public databases. Data citations in the article text are distinct from normal bibliographical citations and should directly link to the database records from which the data can be accessed. In the main text, data citations are formatted as follows: "Data ref: Smith et al, 2001" or "Data ref: NCBI Sequence Read Archive PRJNA342805, 2017". In the Reference list, data citations must be labeled with "[DATASET]". A data reference must provide the database name, accession number/identifiers and a resolvable link to the landing page from which the data can be accessed at the end of the reference. Further instructions are available at .

11) We replaced Supplementary Information with Expanded View (EV) Figures and Tables that are collapsible/expandable online. EV Figures should be cited as 'Figure EV1, Figure EV2" etc... in the text and their respective legends should be included in the main text after the legends of regular figures.
- For the figures that you do NOT wish to display as Expanded View figures, they should be bundled together with their legends in a single PDF file called *Appendix*, which should start with a short Table of Content. Appendix figures should be referred to in the main text as: "Appendix Figure S1, Appendix Figure S2" etc.
- Additional Tables/Datasets should be labeled and referred to as Table EV1, Dataset EV1, etc. Legends have to be provided in a separate tab in case of .xls files. Alternatively, the legend can be supplied as a separate text file (README) and zipped together with the Table/Dataset file.
See detailed instructions here:

12) The paper explained: EMBO Molecular Medicine articles are accompanied by a summary of the articles to emphasize the major findings in the paper and their medical implications for the non-specialist reader. Please provide a draft summary of your article highlighting
- the medical issue you are addressing,
- the results obtained and
- their clinical impact.
This may be edited to ensure that readers understand the significance and context of the research. Please refer to any of our published articles for an example.

13) Author contributions: CRediT has replaced the traditional author contributions section because it offers a systematic machine readable author contributions format that allows for more effective research assessment. Please remove the Authors Contributions from the manuscript and use the free text boxes beneath each contributing author's name in our system to add specific details on the author's contribution. More information is available in our guide to authors.

14) Disclosure statement and competing interests: We updated our journal's competing interests policy in January 2022 and request authors to consider both actual and perceived competing interests. Please review the policy https://www.embopress.org/competing-interests and update your competing interests if necessary.

15) Every published paper now includes a 'Synopsis' to further enhance discoverability. Synopses are displayed on the journal webpage and are freely accessible to all readers. They include a short stand first (maximum of 300 characters, including space) as well as 2-5 one-sentences bullet points that summarizes the paper. Please write the bullet points to summarize the key NEW findings. They should be designed to be complementary to the abstract - i.e. not repeat the same text. We encourage inclusion of key acronyms and quantitative information (maximum of 30 words / bullet point). Please use the passive voice. Please attach these in a separate file or send them by email, we will incorporate them accordingly.

Please also suggest a visual abstract to illustrate your article as a PNG file 550 px wide x 300-600 px high. A cropped portion of this image will serve as thumbnail for the table of content on our webpage.

16) As part of the EMBO Publications transparent editorial process initiative (see our Editorial at http://embomolmed.embopress.org/content/2/9/329), EMBO Molecular Medicine will publish online a Review Process File (RPF) to accompany accepted manuscripts.

In the event of acceptance, this file will be published in conjunction with your paper and will include the anonymous referee reports, your point-by-point response and all pertinent correspondence relating to the manuscript. Let us know whether you agree with the publication of the RPF and as here, if you want to remove or not any figures from it prior to publication.
Please note that the Authors checklist will be published at the end of the RPF.

EMBO Molecular Medicine has a "scooping protection" policy, whereby similar findings that are published by others during review or revision are not a criterion for rejection. Should you decide to submit a revised version, I do ask that you get in touch after three months if you have not completed it, to update us on the status.

I look forward to receiving your revised manuscript.

Yours sincerely,

Lise Roth

Lise Roth, PhD
Senior Editor
EMBO Molecular Medicine

***** Reviewer's comments *****

Referee #2 (Remarks for Author):

The manuscript is well-structured and clearly articulated, providing compelling and thorough evidence that the lncRNA-encoded micropeptide PAMP functions as a tumor suppressor in LUAD by inhibiting PYCR1-driven proline metabolism. The combination of bioinformatics analysis, experimental validation both in vitro and in vivo, and mechanistic exploration contributes to the strength and comprehensiveness of the study. Nonetheless, addressing a few issues could enhance the study's rigor, clarity, and reproducibility.

Major suggestions:

(1) The authors should more distinctly differentiate PAMP from previously identified lncRNA-encoded peptides that influence metabolism to clearly highlight the novel aspects beyond its role in proline metabolism.
(2) The introduction might briefly address the clinical importance of PYCR1 inhibitors and past attempts to target proline metabolism, setting the stage for their therapeutic potential.
(3) The demonstration of endogenous PAMP-PYCR1 interactions is warranted, as the current Co-IP analyses largely rely on overexpression systems.
(4) Although the PAMP-PYCR1 interaction site has been well explored, what are the downstream signaling pathways or metabolic changes besides proline?
(5) The authors show that PAMP knockout or overexpression affects proline levels and proliferation. Rescue experiments comparing wild-type PAMPs and the binding-defective PAMP-F16A mutant in PAMP knockout cells or using PYCR1 mutants incapable of binding PAMP, will provide conclusive evidence supporting a PYCR1-dependent mechanism.
(6) Quantitative measurements of PAMP abundance should be reported, such as copy number or molar concentration relative to PYCR1, and the impact on PYCR1 enzymatic activity should be quantified more accurately (e.g., by determining changes in Km or Vmax). This would provide stronger evidence for direct enzymatic inhibition.
(7) Addressing future optimization strategies, such as peptide modification or nanoparticle delivery, would enhance the translational relevance.
(8) Discuss how this peptide-based metabolic intervention differs from or improves upon existing small-molecule inhibitors of proline metabolism.
Minor suggestions:
(1) Consider adding schematic diagrams to Figures 2 and 4 that illustrate how PAMP interacts with PYCR1 and influences metabolic flux; this will aid readers who are not familiar with proline metabolism.
(2) Include more quantitative details in figures about statistical significance levels, sample sizes, and replicate numbers for in vitro and in vivo experiments. Statistical annotations, including n values and the number of replicates, should be presented

consistently throughout the manuscript.
(3) Abbreviations such as WMDS-netL, LUAD, and FPKM should be explained the first time they appear to ensure clarity for general readers.
(4) Please provide catalog numbers for antibodies and include cell line authentication details, even though the Materials and Methods section is already comprehensive.

Referee #3 (Comments on Novelty/Model System for Author):

the authors explore the function of the identified micropeptide at multiple levels, including cell, mouse and clinical data.

Referee #3 (Remarks for Author):

In this study, the authors identified a 48-amino acid micropeptide named PAMP, encoded by the lncRNA PSMA3-AS1, as a novel tumor suppressor in lung adenocarcinoma (LUAD). The authors showed that PAMP is significantly downregulated in LUAD tissues and positively correlates with favorable prognosis. They showed that PAMP inhibits LUAD cell proliferation in vitro and suppresses tumor growth in vivo. Moreover, PAMP interacts with PYCR1, decreasing proline accumulation. And synthetic PAMP administration effectively reduces intracellular proline levels and impairing tumor progression, offering a new opportunity for metabolic intervention in precision oncology.
In general, the manuscript contains interesting data and the experiments are well performed. However, some of the interpretation/conclusions are not sufficiently supported by the data as indicated below. The following concerns need to be addressed.

Major concerns:
1, Line 209-211, the authors claimed that: Mass spectrometry analysis of the GFP immunoprecipitates identified a unique peptide fragment corresponding to PAMP (Fig .2F), further supporting its endogenous translation. However, the provided data is not sufficient to support its endogenous translation
2, Line 214-216, the antibody specificity needs to be determined by immunostaining using KO or KD cells. Moreover, in Fig. 4E, the authors showed that PAMP and PYCR1 colocalized using immunofluorescence. However, the figure resolution is not good enough to show their localization in mitochondria given that PYCR1 is a mitochondrial enzyme.
3, Line 236-237, the authors showed that knockdown of the PAMP significantly accelerated the LUAD tumor development in vivo (Fig 3I-K). It is better to provide the result of complementing the KO cells with wild-type PAMP or PAMPmut, which can strengthen their conclusion that the micropeptide PAMP functions as a tumor suppressor.
4, Line 293-297, based on Fig.5K and L, the authors claimed that PAMP's impact on proline synthesis is dependent on PYCR1 activity. However, the data do not provided enough evidence to support their conclusion, which only show that PAMP and PYCR1 function epistatically on proline synthesis.
The authors showed that PAMP does not affect PYCR1 expression. Although they mentioned that PAMP association affects PYCR1 activity, however, no direct evidence supports that PAMP affects PYCR1 activity. How PAMP interaction affects PYCR1 activity remains unclear.

5, Proteasome 20S subunit alpha 3 antisense RNA 1 (PSMA3-AS1), has been reported as a tumor promoter LncRNA in a wide range of human malignancies. Several studies also showed that it promotes A549 and H460 cells proliferation (Wang L, 2020. Oncology Rep; Cheng G, 2021. Adv Clin Exp Med; Kan L, Biol Direct. 2023). Whether the PSMA3-AS1 in the manuscript is the same one as above? If so, the authors need to discuss the discrepancy in their results, which will help the readers to understand the function of the conserved lncRNA.

Minor concerns:
1, Result section: some of the subheadings are kind of too long, should be succinct/concise.
2, Fig S1B, the colors indicating nuclear and cytoplasmic fractions are mislabeled.
3, In Figure legends, Fig.4D and E are not described correctly.
4, The GSEA analysis in Fig 4F should include FDR.

Responses to Reviewer Comments

We sincerely thank the Editor and all reviewers for their careful evaluation of our manuscript and for their constructive and insightful comments. Their feedback has substantially improved the quality and clarity of the revised version. Below, we provide the reviewers' comments in *italicized* font, with specific concerns numbered for ease of reference. Major revisions have been incorporated into the manuscript, and the corresponding changes are indicated by page and line numbers. All modifications are highlighted in red in the revised manuscript.

Referee #2 (Remarks for Author):

The manuscript is well-structured and clearly articulated, providing compelling and thorough evidence that the lncRNA-encoded micropeptide PAMP functions as a tumor suppressor in LUAD by inhibiting PYCR1-driven proline metabolism. The combination of bioinformatics analysis, experimental validation both in vitro and in vivo, and mechanistic exploration contributes to the strength and comprehensiveness of the study. Nonetheless, addressing a few issues could enhance the study's rigor, clarity, and reproducibility.

Response: We sincerely thank the reviewer for the positive and encouraging evaluation of our work. We also appreciate the reviewer's thoughtful suggestions for further improving the rigor, clarity, and reproducibility of the manuscript. We have carefully addressed each comment point by point below.

Major suggestions

(1) The authors should more distinctly differentiate PAMP from previously identified lncRNA-encoded peptides that influence metabolism to clearly highlight the novel aspects beyond its role in proline metabolism.

Response: We thank the reviewer for this insightful suggestion. We fully agree that clearly distinguishing the micropeptide PAMP from previously reported lncRNA-encoded peptides is crucial for highlighting the novelty of our findings. In the revised manuscript, we have expanded the Discussion to provide a comparative analysis of representative metabolism-related lncRNA-encoded peptides and the distinct mechanism of PAMP (**page 12**, **lines 440-459**). The table below summarizes the major differences.

Micropeptide	Targeted process	Mechanism	Key differences from PAMP
**PAMP (This study)**	Proline metabolism	Directly binds to and inhibits the enzymatic activity of PYCR1.	Acts via direct inhibition of a metabolic enzyme's activity.
**HOXB-AS3**	Glucose metabolism (Warburg effect)	Regulates the PKM1/PKM2 splice switch to influence glycolytic flux.	Targets alternative splicing to indirectly modulate metabolism.

Micropeptide	Targeted process	Mechanism	Key differences from PAMP
**ASAP**	Mitochondrial energy metabolism	Enhances the activity of ATP synthase (Complex V).	Functions as an activator of a metabolic enzyme complex.
**XBP1SBM**	Glutamine metabolism	Acts through the XBP1s/VEGF axis to enhance glutamine uptake.	Exerts effect indirectly through a transcriptional signaling axis.
**LncRIM**	Iron metabolism	Binds to and inhibits NF2, leading to LATS1 inhibition and YAP activation.	Functions by modulating an upstream signaling pathway (Hippo/YAP).

As summarized above, while several lncRNA-encoded peptides have been reported to influence cellular metabolism, their effects are generally mediated through indirect mechanisms, such as regulation of alternative splicing (e.g., HOXB-AS3), activation of enzyme complexes (e.g., ASAP), or modulation of upstream signaling pathways (e.g., XBP1SBM and LncRIM). In contrast, our study reveals that PAMP functions as a direct and specific inhibitor of the metabolic enzyme PYCR1. PAMP physically interacts with PYCR1 and suppresses its catalytic activity, thereby reducing proline biosynthesis. This mechanism of direct enzymatic inhibition is a distinctive and novel aspect of PAMP, setting it apart from previously reported lncRNA-encoded peptides and introducing a new mode of metabolic regulation by micropeptides.

(2) The introduction might briefly address the clinical importance of PYCR1 inhibitors and past attempts to target proline metabolism, setting the stage for their therapeutic potential.

Response: We thank the reviewer for this valuable suggestion. In the revised Introduction, we have added a brief discussion of the clinical relevance of targeting proline metabolism and the current status of PYCR1-related therapeutic strategies. Specifically, we note that several inhibitors targeting PYCR1 or proline metabolism have been explored in preclinical studies, including the PYCR1 inhibitor PYCR1-IN-1 and PRODH inhibitors such as L-selenomethionine, quinolinic acid, phenylbenzimidazole, and ascivystatin. However, the development of highly effective and selective inhibitors suitable for clinical application remains challenging (**pages 5-6, lines 144-148**). In this context, PAMP is of particular interest because it acts as a naturally occurring and specific inhibitor of PYCR1 through a mechanism distinct from that of conventional small molecules. These features suggest that PAMP may provide a new conceptual framework and a promising therapeutic avenue for LUAD treatment (**pages 11-12, 429-459**).

(3) The demonstration of endogenous PAMP-PYCR1 interactions is warranted, as the current Co-IP analyses largely rely on overexpression systems.

Response: We thank the reviewer for this important point. To address the concern regarding the endogenous PAMP-PYCR1 interaction, we performed additional experiments under endogenous conditions. Specifically, using an anti-PYCR1 antibody in Co-IP assays, we successfully detected endogenous PAMP and further identified PAMP-specific peptide

sequences by LC-MS. In addition, we examined the mitochondrial co-localization of PAMP and PYCR1 under endogenous conditions, which provided further evidence supporting their physical interaction (**Fig 4D, 4E**).

(4) Although the PAMP-PYCR1 interaction site has been well explored, what are the downstream signaling pathways or metabolic changes besides proline?

Response: We thank the reviewer for this insightful question. To systematically identify downstream pathways potentially regulated by the PAMP–PYCR1 axis, we first queried the SMPDB database and found that PYCR1 and proline are potentially linked to several key pathways, including JNK, HIF-1, TGF-beta, PI3K–Akt, JAK–STAT, AMPK, and MAPK signaling. We then analyzed the RNA-seq data from KO-PAMP cells to determine whether these pathways were enriched. GSEA showed significant enrichment of the PI3K–Akt, JAK–STAT, and MAPK signaling pathways, all of which are well documented to be directly associated with cell proliferation. Based on these findings, we focused our experimental validation on these candidate pathways using both KO-PAMP and KO-PYCR1 A549 cells. Notably, PAMP knockout increased p-AKT levels, whereas PYCR1 knockout decreased p-AKT levels, supporting AKT as a key downstream mediator of the PAMP–PYCR1–proline axis (**page 9, lines 304-315** and **Fig EV5D- 5F**).

With respect to metabolic changes beyond proline, untargeted metabolomic profiling of KO-PAMP cells revealed alterations in a broad range of metabolites. However, based on current annotation from HMDB and other available datasets, proline is the only metabolite directly linked to PYCR1 activity. Therefore, proline is presently the only clearly defined metabolite downstream of the PAMP–PYCR1 axis. To further delineate the broader metabolic consequences of this pathway, we plan to perform more focused metabolomic analyses in future studies, particularly targeting metabolic processes most likely to be regulated by PYCR1.

(5) The authors show that PAMP knockout or overexpression affects proline levels and proliferation. Rescue experiments comparing wild-type PAMPs and the binding-defective PAMP-F16A mutant in PAMP knockout cells or using PYCR1 mutants incapable of binding PAMP, will provide conclusive evidence supporting a PYCR1-dependent mechanism.

Response: We thank the reviewer for this constructive suggestion. To directly test whether the effects of PAMP on proline metabolism and cell proliferation depend on its interaction with PYCR1, we performed the recommended rescue experiments using wild-type PAMP, the binding-defective PAMP(F16A) mutant, and the catalytically inactive PYCR1(N123A) mutant. Our results showed that co-expression of PAMP(F16A) with wild-type PYCR1 significantly increased both cell proliferation and proline accumulation, whereas these effects were markedly attenuated when PAMP(F16A) was co-expressed with the catalytically inactive PYCR1(N123A) mutant. Notably, the double-mutant combination, PAMP(F16A) plus PYCR1(N123A), produced only partial restoration of these phenotypes. Together, these findings provide strong evidence that PAMP regulates proline metabolism and cell growth through a PYCR1-dependent mechanism that requires both physical interaction with PYCR1 and intact PYCR1 enzymatic activity. These results have been incorporated into the revised manuscript (**page 10, lines 340-347** and **Fig 6K, 6L, EV6F**).

(6) Quantitative measurements of PAMP abundance should be reported, such as copy number or molar concentration relative to PYCR1, and the impact on PYCR1 enzymatic activity should be quantified more accurately (e.g., by determining changes in Km or Vmax). This would provide stronger evidence for direct enzymatic inhibition.

Response: We thank the reviewer for this valuable suggestion. We agree that quantitative measurement of PAMP abundance relative to PYCR1, together with a more precise kinetic analysis of PYCR1 inhibition, would provide stronger support for direct enzymatic inhibition. In the revised study, we attempted to quantify PAMP abundance as suggested; however, accurate determination of its absolute copy number or molar concentration relative to PYCR1 was technically difficult under the current experimental conditions. Therefore, to more directly assess the inhibitory effect of PAMP on PYCR1, we performed enzyme kinetic analyses in conjunction with proline measurements.

Our results showed that PAMP decreased the Vmax of PYCR1 from 10.12 to 6.394 and reduced the Km from 2.401 to 2.042. These findings support a mixed-type inhibitory mechanism, suggesting that PAMP impairs both substrate binding and catalytic turnover of PYCR1, ultimately leading to reduced proline synthesis. These data have now been incorporated into the revised manuscript (**page 10, lines 366-370** and **Fig 7H**).

(7) Addressing future optimization strategies, such as peptide modification or nanoparticle delivery, would enhance the translational relevance.

Response: We sincerely thank the reviewer for raising this important point. We agree that discussing optimization strategies, such as peptide modification and nanoparticle-based delivery, would enhance the translational relevance of our findings on PAMP. In the revised manuscript, we have added a brief discussion on this aspect.

Specifically, lipid nanoparticle (LNP) technology may provide an effective platform for the delivery of PAMP. For example, surface modification of LNPs with cyclic RGD (c-RGD) peptides could enable selective targeting of $\alpha v\beta 3$ integrin, which is highly expressed in tumor vasculature and in certain tumor cells, thereby enhancing tumor-directed delivery. In addition, incorporation of cell-penetrating peptides such as TAT may improve intracellular uptake. Together, these delivery and targeting strategies may help improve the stability, specificity, and therapeutic potential of PAMP, and thus represent promising directions for future translational development (**pages 11-12, lines 429-437**).

(8) Discuss how this peptide-based metabolic intervention differs from or improves upon existing small-molecule inhibitors of proline metabolism.

Response: We thank the reviewer for this valuable comment. Peptide-based metabolic intervention by PAMP differs from existing small-molecule inhibitors of proline metabolism in several important respects. Conventional small-molecule inhibitors, including PYCR1 and PRODH inhibitors, generally target the catalytic sites of metabolic enzymes, which may be associated with limited selectivity, off-target toxicity, and susceptibility to acquired resistance. By contrast, PAMP is an endogenous micropeptide that may regulate PYCR1 through mechanisms less readily achieved by small molecules, such as modulation of protein–protein interactions or allosteric effects on PYCR1 activity and oligomerization.

Moreover, as a naturally occurring peptide, PAMP may have potential advantages in biocompatibility and safety, given that its degradation products are endogenous amino acids. Thus, PAMP represents a distinct and potentially advantageous mode of metabolic intervention. Through direct inhibition of PYCR1 and consequent suppression of proline metabolism, PAMP

limits LUAD growth and highlights a promising therapeutic avenue for future development. This discussion has now been incorporated into the revised manuscript (**page 12, lines 440-459**).

Minor suggestions:

(1) Consider adding schematic diagrams to Figures 2 and 4 that illustrate how PAMP interacts with PYCR1 and influences metabolic flux; this will aid readers who are not familiar with proline metabolism.

Response: We thank the reviewer for this excellent suggestion. In the revised manuscript, we added a schematic diagram to Figures 4 to illustrate the proposed interaction between PAMP and PYCR1 and its consequent impact on proline metabolic flux, which will greatly aid reader comprehension (**Fig EV4C**).

(2) Include more quantitative details in figures about statistical significance levels, sample sizes, and replicate numbers for in vitro and in vivo experiments. Statistical annotations, including n values and the number of replicates, should be presented consistently throughout the manuscript.

Response: We thank the reviewer for this important suggestion. We have now incorporated more comprehensive quantitative information into all figures, including exact sample sizes (n values), replicate numbers, and statistical significance annotations. In addition, we have revised the figure labels and legends to present this information in a consistent manner across all in vitro and in vivo experiments.

(3) Abbreviations such as WMDS-netL, LUAD, and FPKM should be explained the first time they appear to ensure clarity for general readers.

Response: We thank the reviewer for this suggestion. In the revised manuscript, all abbreviations, including WMDS-netL, LUAD, and FPKM, are now spelled out at first mention to ensure clarity for general readers.

(4) Please provide catalog numbers for antibodies and include cell line authentication details, even though the Materials and Methods section is already comprehensive.

Response: We thank the reviewer for this helpful suggestion. In the revised manuscript, we have added the catalog numbers for all antibodies to the **Reagents and Tools table** and included cell line authentication information in the **Supplementary Information** to further improve methodological transparency and reproducibility.

Referee #3 (Remarks for Author):

In this study, the authors identified a 48-amino acid micropeptide named PAMP, encoded by the lncRNA PSMA3-AS1, as a novel tumor suppressor in lung adenocarcinoma (LUAD). The authors showed that PAMP is significantly downregulated in LUAD tissues and positively

correlates with favorable prognosis. They showed that PAMP inhibits LUAD cell proliferation in vitro and suppresses tumor growth in vivo. Moreover, PAMP interacts with PYCR1, decreasing proline accumulation. And synthetic PAMP administration effectively reduces intracellular proline levels and impairing tumor progression, offering a new opportunity for metabolic intervention in precision oncology.
In general, the manuscript contains interesting data and the experiments are well performed. However, some of the interpretation/conclusions are not sufficiently supported by the data as indicated below. The following concerns need to be addressed.

Response: We sincerely thank the reviewer for the thoughtful assessment of our manuscript and for recognizing the interest of the study and the quality of the experimental work. We also greatly appreciate the reviewer's constructive concerns regarding aspects of data interpretation and the strength of certain conclusions. We have carefully addressed each comment point by point below.

Major concerns:

(1) Line 209-211, the authors claimed that: Mass spectrometry analysis of the GFP immunoprecipitates identified a unique peptide fragment corresponding to PAMP (Fig .2F), further supporting its endogenous translation. However, the provided data is not sufficient to support its endogenous translation.

Response: We thank the reviewer for this important point. To address this concern, we performed additional experiments under endogenous conditions. Specifically, we carried out Co-IP assays using an anti-PYCR1 antibody to capture endogenous protein complexes from cell lysates under native conditions, followed by LC-MS/MS analysis. In these PYCR1 immunoprecipitates, we identified unique peptide sequences matching the predicted PAMP ORF, whereas these peptides were not detected in the isotype-matched IgG control. These results provide stronger evidence that PAMP is endogenously translated under physiological conditions and exists in association with endogenous PYCR1 (**Fig 4D and Fig EV4D**).

(2) Line 214-216, the antibody specificity needs to be determined by immunostaining using KO or KD cells. Moreover, in Fig. 4E, the authors showed that PAMP and PYCR1 colocalized using immunofluorescence. However, the figure resolution is not good enough to show their localization in mitochondria given that PYCR1 is a mitochondrial enzyme.

Response: We thank the reviewer for these constructive suggestions. To validate antibody specificity, we performed immunostaining in PAMP KO cells and observed a marked reduction in PAMP signal compared with control cells, supporting the specificity of the antibody **(page7, lines 216-217** and **Fig EV2D)**.

To address the concern regarding subcellular localization, we additionally performed high-resolution confocal imaging in combination with MitoTracker staining. These experiments showed clear co-localization of PAMP and PYCR1 within mitochondria, consistent with the known mitochondrial localization of PYCR1 **(Fig 4E)**.

(3) Line 236-237, the authors showed that knockdown of the PAMP significantly accelerated the LUAD tumor development in vivo (Fig 3I-K). It is better to provide the result of

complementing the KO cells with wild-type PAMP or PAMPmut, which can strengthen their conclusion that the micropeptide PAMP functions as a tumor suppressor.

Response: We thank the reviewer for this valuable suggestion. To further substantiate the tumor-suppressive role of PAMP, we performed complementation experiments by re-expressing either wild-type PAMP or the mutant form in PAMP-deficient cells and then evaluated their effects in a xenograft mouse model. We found that reintroduction of wild-type PAMP effectively rescued the accelerated tumor growth phenotype caused by PAMP loss, whereas the mutant form failed to do so. These results provide direct in vivo evidence that PAMP functions as a tumor suppressor and that this effect depends on its intact functional activity (**Figs 3J, 3K and EV3F**).

(4) Line 293-297, based on Fig.5K and L, the authors claimed that PAMP's impact on proline synthesis is dependent on PYCR1 activity. However, the data do not provided enough evidence to support their conclusion, which only show that PAMP and PYCR1 function epistatically on proline synthesis.
The authors showed that PAMP does not affect PYCR1 expression. Although they mentioned that PAMP association affects PYCR1 activity, however, no direct evidence supports that PAMP affects PYCR1 activity. How PAMP interaction affects PYCR1 activity remains unclear.

Response: We thank the reviewer for this insightful comment. We agree that the data shown in Fig. 5K and L support a functional epistatic relationship between PAMP and PYCR1 in the regulation of proline synthesis, but do not by themselves provide definitive proof that PAMP directly modulates PYCR1 enzymatic activity. In response to this concern, we have revised the wording in the manuscript to more accurately state that PAMP regulates proline metabolism in a **PYCR1-dependent** manner, rather than claiming direct enzymatic inhibition based solely on these data. Specifically, we now clarify that PYCR1 knockdown markedly reduced proline accumulation and that, in the absence of PYCR1, PAMP overexpression no longer altered proline levels. These findings indicate that the effect of PAMP on proline metabolism requires PYCR1 (**page 9, lines 300-303** and **Fig 5K, 5L**). We have also acknowledged in the revised manuscript that the precise mechanism by which PAMP influences PYCR1 activity requires further investigation.

Regarding the reviewer's question of how PAMP binding may affect PYCR1 activity, our data confirm that PAMP interacts with PYCR1 without altering PYCR1 expression. To further explore the possible mechanistic basis of this effect, we considered the established structural organization of PYCR1. Its N-terminal Rossmann fold domain (residues 1–162) is responsible for NAD(P)H cofactor binding and hydride transfer, whereas the C-terminal α-helical domain contributes to P5C substrate recognition and homooligomerization into the active decameric complex (Christensen *EM et al, 2017. J Biol Chem; Bogner AN et al, 2021. Amino acids*). These domains are spatially coordinated and jointly form the functional catalytic architecture of the enzyme. In our study, computational analyses using PROVEAN and SIFT identified N123 within the Rossmann fold as a functionally important residue, and subsequent interaction assays suggested that the PAMP F16 residue is critical for binding to PYCR1 involving the N123 region. Based on these observations, we propose that PAMP binding may induce conformational perturbations in the Rossmann fold and thereby interfere with cofactor binding,

substrate positioning, interdomain communication, or higher-order assembly of PYCR1, ultimately reducing catalytic activity without affecting protein abundance. Because this model remains inferential, we have presented it cautiously in the revised manuscript and noted that future structural studies, such as X-ray crystallography, NMR, or cryo-EM, will be needed to define the underlying mechanism more precisely (**page 11, lines 413-419**).

(5) Proteasome 20S subunit alpha 3 antisense RNA 1 (PSMA3-AS1), has been reported as a tumor promoter LncRNA in a wide range of human malignancies. Several studies also showed that it promotes A549 and H460 cells proliferation (Wang L, 2020. Oncology Rep; Cheng G, 2021. Adv Clin Exp Med; Kan L, Biol Direct. 2023). Whether the PSMA3-AS1 in the manuscript is the same one as above? If so, the authors need to discuss the discrepancy in their results, which will help the readers to understand the function of the conserved lncRNA.

Response: We thank the reviewer for this insightful comment. The PSMA3-AS1 examined in our study is indeed the same lncRNA reported in previous studies (*Wang et al., Oncology Reports, 2020; Cheng et al., Advances in Clinical and Experimental Medicine, 2021; Kan et al., Biology Direct, 2023*). We agree that the apparent discrepancy between our findings and these earlier reports warrants discussion.

To ensure the robustness of our results, we performed loss-of-function experiments using two independent siRNAs, thereby reducing the likelihood of off-target effects. This approach is more stringent than the single-siRNA strategy used in the prior studies. In addition, our integrated analysis of multiple independent clinical datasets, including TCGA, GTEx, GEO, and our own LUAD cohort, consistently showed that PSMA3-AS1 is significantly downregulated in LUAD tissues. These clinical observations support a tumor-suppressive role for PSMA3-AS1 in LUAD and are consistent with our functional results.

We speculate that the divergent findings may reflect context-dependent functions of PSMA3-AS1, potentially influenced by differences in cellular state, genetic background, or laboratory-specific characteristics of the A549 and H460 cell lines. To address this possibility, we verified the identity and purity of our cell lines by STR profiling, which supported the validity of our experimental system (**Supplementary Information**). In the revised manuscript, we have expanded the Discussion to address this discrepancy and to emphasize that the function of PSMA3-AS1 may be context dependent and merits further investigation (**page 11, lines 390-398**).

Minor concerns:

1, Result section: some of the subheadings are kind of too long, should be succinct/concise.

Response: We thank the reviewer for this helpful suggestion. In the revised manuscript, we have shortened several subheadings in the Results section to make them more concise and reader-friendly, thereby improving the overall clarity and readability of the manuscript.

2, Fig S1B, the colors indicating nuclear and cytoplasmic fractions are mislabeled.

Response: We thank the reviewer for pointing this out. In the revised manuscript, we have corrected the color labels in Fig. S1B to accurately indicate the nuclear and cytoplasmic

fractions (**Fig EV1B**).

3, In Figure legends, Fig.4D and E are not described correctly.

Response: We thank the reviewer for identifying this error. In the revised manuscript, the legend descriptions for Fig. 4D and E have been revised to accurately reflect the corresponding data shown in the figures.

4, The GSEA analysis in Fig 4F should include FDR.

Response: We thank the reviewer for this helpful suggestion. In the revised manuscript, we have added the FDR values (i.e., adjusted p value) to the GSEA results shown in Fig. 4F to provide a more complete and rigorous presentation of the enrichment analysis.

29th Apr 2026

Dear Prof. Liu,

Thank you for submitting your revised study. The referees have evaluated your revised manuscript and as you will see below, they are satisfied with the revisions. We will therefore be able to accept your manuscript once the following editorial matters are addressed:

1/ Manuscript text:
- Please remove the red font text and only keep in track changes mode any new modification.
- Please note that author Xiaofan Wu is missing in the submission system.
- Update the headings and order of the sections in the manuscript text to: Abstract / The Paper Explained / Introduction / Results / Discussion / Methods / Data Availability / Acknowledgements / Disclosure and Competing Interests Statement / References / Figure Legends / Expanded View Figure Legends.
- Move the ethics approval to the Methods, delete the heading "Declarations". Remove Consent for publication and Availability of data and materials.
- Methods:
o Remove the reagents and tools table from manuscript text and upload it as a separate file.
o Human participants: State details of authority granting ethics approval, provide reference number for approval.
o Cells: please indicate whether the cells were authenticated and tested for mycoplasma contamination.
o Antibodies: please provide dilutions / concentrations. Please provide additional information on the anti-PAMP antibody, particularly on the generation of synthetic peptides.
o Mice: please provide origin, as well as housing and husbandry conditions. Include a statement of compliance with ethical regulations.
o Statistics: please include a statement about sample size, blinding, randomization and exclusion/inclusion criteria.
- Data Availability section: it should only list newly generated datasets that have not been previously published. Metabolomics data should be deposited in an adequate repository.
- Merge Funding with Acknowledgments.
- Please remove Author contributions from the manuscript text, and provide instead CRediT (Contributor Role Taxonomy) terms in the submission system. These replace a narrative author contribution section in the manuscript.
- 'Competing interests' should be renamed to 'Disclosure and Competing Interests Statement'.

2/ Figures:
- Please provide individual data points in your graphs where possible.
- Please define axis unit for all graphs (including % or fold change).
- Provide and define scale bars for all pictures.
- Please remove the figures from the manuscript text, they should be uploaded as separate files only. Add the expanded view figure legends to the manuscipt text, after the main figure legends.
- Figure callouts are missing for Fig. 1G, I, J in the text. There is a callout for Fig. 1K, which does not exist.
- Table EV1and Table EV2 should be uploaded as as two separate files.
- Please address the queries from our data editors in the figure legends:
1. Please note that the exact p values are not provided in the legends of figures 3C, D, F, H, I, K; 4H, 5A, D, E, G, H, J, L; 6D, E, H, I, K, L; 7E, F; EV3 C, D, F; EV7 E-G
2. Please note that the box plots need to be defined in terms of minima, maxima, centre, bounds of box and whiskers, and percentile in the legends of figures 1C, D; EV5 A, C
3. Please note that information related to n is missing in the legends of figure 4A
4. Please note that scale bar and its definition are missing for figures 2G, 7A, EV2 D-F; EV7 D

3/ Checklist:
Check that the following applies to your study and correct if needed:
- Primary cultures
- Animal observed in or captured from the field
- Ethics/ specimen and field samples

Fill in the following sections:
- Housing and husbandry conditions
- Experimental study design and statistics, all sections
- Ethics / human participants - authority grnatic ethics approval

4/ Source Data
- Please check the images provided for Fig 2C, as we cannot open them all.
- Western blots should be annotated.

5/ Remove the synopsis and visual abstract from the manuscript text and upload them as individual files.

6/ As part of the EMBO Publications transparent editorial process initiative (see our Editorial at http://embomolmed.embopress.org/content/2/9/329), EMBO Molecular Medicine will publish online a Review Process File (RPF) to accompany accepted manuscripts.
This file will be published in conjunction with your paper and will include the anonymous referee reports, your point-by-point response and all pertinent correspondence relating to the manuscript. Let us know whether you agree with the publication of the RPF and as here, if you want to remove or not any figures from it prior to publication.
Please note that the Authors checklist will be published at the end of the RPF.

I look forward to receiving your revised manuscript.

Yours sincerely,

Lise Roth

Lise Roth, PhD
Senior Editor
EMBO Molecular Medicine

***** Reviewer's comments *****

Referee #2 (Remarks for Author):

The authors have adequately addressed the comments.

Referee #3 (Comments on Novelty/Model System for Author):

the authors explore the function of the identified micropeptide at multiple levels, including cell, mouse and clinical data.

Referee #3 (Remarks for Author):

The authors have addressed my concerns satisfactorily.

The authors addressed the remaining editorial issues.

27th May 2026

Dear Prof. Liu,

Thank you for sending your revised files. We are pleased to inform you that your manuscript is accepted for publication and is now being sent to our publisher to be included in the next available issue of EMBO Molecular Medicine.

Your manuscript will be processed for publication by EMBO Press. It will be copy edited and you will receive page proofs prior to publication. Please note that you will be contacted by Springer Nature Author Services to complete licensing and payment information.

You may qualify for financial assistance for your publication charges - either via a Springer Nature fully open access agreement or an EMBO initiative. Check your eligibility: https://link.springer.com/journal/44321/how-to-publish-with-us

Should you be planning a Press Release on your article, please notify the production team via this form in order to coordinate publication and release dates: https://forms.cloud.microsoft/e/eH4A8JVP6g

If you have any questions, please do not hesitate to contact the Editorial Office. Thank you for your contribution to EMBO Molecular Medicine.

Yours sincerely,

Lise Roth, Ph.D
Senior Editor
EMBO Molecular Medicine

>>> Please note that it is EMBO Molecular Medicine policy for the transcript of the editorial process (containing referee reports and your response letter) to be published as an online supplement to each paper. If you do NOT want this, you will need to inform the Editorial Office via email immediately. More information is available here: https://link.springer.com/partners/embo-press/editorial-policies#Peer%20review

EMBO Press Author Checklist

Corresponding Author Name: Pengyuan Liu; Yan Lu	
Journal Submitted to: EMBO Molecular Medicine	
Manuscript Number: EMM-2025-22684	

USEFUL LINKS FOR COMPLETING THIS FORM
The EMBO Journal - Author Guidelines
EMBO Reports - Author Guidelines
Molecular Systems Biology - Author Guidelines
EMBO Molecular Medicine - Author Guidelines

Reporting Checklist for Life Science Articles (updated January
This checklist is adapted from Materials Design Analysis Reporting (MDAR) Checklist for Authors. MDAR establishes a minimum set of requirements in transparent reporting in the life sciences (see Statement of Task: 10.31222/osf.io/9sm4x). Please follow the journal's guidelines in preparing your
Please note that a copy of this checklist will be published alongside your article.

Abridged guidelines for figures
1. Data
The data shown in figures should satisfy the following conditions:
- the data were obtained and processed according to the field's best practice and are presented to reflect the results of the experiments in an accurate and unbiased manner.
- ideally, figure panels should include only measurements that are directly comparable to each other and obtained with the same assay.
- plots include clearly labeled error bars for independent experiments and sample sizes. Unless justified, error bars should not be shown for technical
- if n<5, the individual data points from each experiment should be plotted. Any statistical test employed should be justified.
- Source Data should be included to report the data underlying figures according to the guidelines set out in the authorship guidelines on Data

2. Captions
Each figure caption should contain the following information, for each panel where they are relevant:
- a specification of the experimental system investigated (eg cell line, species name).
- the assay(s) and method(s) used to carry out the reported observations and measurements.
- an explicit mention of the biological and chemical entity(ies) that are being measured.
- an explicit mention of the biological and chemical entity(ies) that are altered/varied/perturbed in a controlled manner.
- the exact sample size (n) for each experimental group/condition, given as a number, not a range;
- a description of the sample collection allowing the reader to understand whether the samples represent technical or biological replicates (including how many animals, litters, cultures, etc.).
- a statement of how many times the experiment shown was independently replicated in the laboratory.
- definitions of statistical methods and measures:
  - common tests, such as t-test (please specify whether paired vs. unpaired), simple χ2 tests, Wilcoxon and Mann-Whitney tests, can be unambiguously identified by name only, but more complex techniques should be described in the methods section;
  - are tests one-sided or two-sided?
  - are there adjustments for multiple comparisons?
  - exact statistical test results, e.g., P values = x but not P values < x;
  - definition of 'center values' as median or average;
  - definition of error bars as s.d. or s.e.m.

Please complete ALL of the questions below.
Select "Not Applicable" only when the requested information is not relevant for your study.

Materials

Newly Created Materials	Information included in the manuscript?	In which section is the information available? (Reagents and Tools Table, Materials and Methods, Figures, Data Availability Section)
New materials and reagents need to be available; do any restrictions apply?	Not Applicable	

Antibodies	Information included in the manuscript?	In which section is the information available? (Reagents and Tools Table, Materials and Methods, Figures, Data Availability Section)
For **antibodies** provide the following information:<br>- Commercial antibodies: RRID (if possible) or supplier name, catalogue number and or/clone number<br>- Non-commercial: RRID or citation	Yes	Reagents and Tools Table

DNA and RNA sequences	Information included in the manuscript?	In which section is the information available? (Reagents and Tools Table, Materials and Methods, Figures, Data Availability Section)
**Short novel DNA or RNA including primers, probes**: provide the sequences.	Yes	Reagents and Tools Table

Cell materials	Information included in the manuscript?	In which section is the information available? (Reagents and Tools Table, Materials and Methods, Figures, Data Availability Section)
**Cell lines:** Provide species information, strain. Provide accession number in repository **OR** supplier name, catalog number, clone number, and/**OR** RRID.	Yes	Reagents and Tools Table
**Primary cultures:** Provide species, strain, sex of origin, genetic modification status.	Not Applicable	
Report if the cell lines were recently **authenticated** (e.g., by STR profiling) and tested for mycoplasma contamination.	Yes	Supplementary information

Experimental animals	Information included in the manuscript?	In which section is the information available? (Reagents and Tools Table, Materials and Methods, Figures, Data Availability Section)
**Laboratory animals or Model organisms:** Provide species, strain, sex, age, genetic modification status. Provide accession number in repository **OR** supplier name, catalog number, clone number, **OR** RRID.	Yes	Methods
**Animal observed in or captured from the field:** Provide species, sex, and age where possible.	Not Applicable	
Please detail **housing and husbandry conditions**.	Yes	Methods

Plants and microbes	Information included in the manuscript?	In which section is the information available? (Reagents and Tools Table, Materials and Methods, Figures, Data Availability Section)
**Plants:** provide species and strain, ecotype and cultivar where relevant, unique accession number if available, and source (including location for collected wild specimens).	Not Applicable	
**Microbes:** provide species and strain, unique accession number if available, and source.	Not Applicable	

Human research participants	Information included in the manuscript?	In which section is the information available? (Reagents and Tools Table, Materials and Methods, Figures, Data Availability Section)
If collected and within the bounds of privacy constraints report on age, sex and gender or ethnicity for all study participants.	Not Applicable	

Core facilities	Information included in the manuscript?	In which section is the information available? (Reagents and Tools Table, Materials and Methods, Figures, Data Availability Section)
If your work benefited from core facilities, was their service mentioned in the acknowledgments section?	Yes	Acknowledgments

Design

Study protocol	Information included in the manuscript?	In which section is the information available? (Reagents and Tools Table, Materials and Methods, Figures, Data Availability Section)
If study protocol has been **pre-registered, provide DOI in the manuscript**. For clinical trials, provide the trial registration number **OR** cite DOI.	Not Applicable	
Report the **clinical trial registration number** (at ClinicalTrials.gov or equivalent), where applicable.	Not Applicable	

Laboratory protocol	Information included in the manuscript?	In which section is the information available? (Reagents and Tools Table, Materials and Methods, Figures, Data Availability Section)
Provide DOI OR other citation details if **external detailed step-by-step protocols** are available.	Not Applicable	

Experimental study design and statistics	Information included in the manuscript?	In which section is the information available? (Reagents and Tools Table, Materials and Methods, Figures, Data Availability Section)
Include a statement about **sample size** estimate even if no statistical methods were used.	Yes	Methods
Were any steps taken to minimize the effects of subjective bias when allocating animals/samples to treatment (e.g. **randomization procedure**)? If yes, have they been described?	Yes	Figure 7
Include a statement about **blinding** even if no blinding was done.	Yes	Methods
Describe **inclusion/exclusion criteria** if samples or animals were excluded from the analysis. Were the criteria pre-established?<br><br>If sample or data points were omitted from analysis, report if this was due to attrition or intentional exclusion and provide justification.	Yes	Methods
For every figure, are **statistical tests** justified as appropriate? Do the data meet the assumptions of the tests (e.g., normal distribution)? Describe any methods used to assess it. Is there an estimate of variation within each group of data? Is the variance similar between the groups that are being statistically compared?	Yes	Methods

Sample definition and in-laboratory replication	Information included in the manuscript?	In which section is the information available? (Reagents and Tools Table, Materials and Methods, Figures, Data Availability Section)
In the figure legends: state number of times the experiment was **replicated** in laboratory.	Yes	figure legends
In the figure legends: define whether data describe **technical or biological replicates**.	Yes	figure legends

Ethics

Ethics	Information included in the manuscript?	In which section is the information available? (Reagents and Tools Table, Materials and Methods, Figures, Data Availability Section)
Studies involving **human participants**: State details of **authority granting ethics approval** (IRB or equivalent committee(s), provide reference number for approval.	Yes	Methods
Studies involving **human participants**: Include a statement confirming that **informed consent** was obtained from all subjects and that the experiments conformed to the principles set out in the WMA Declaration of Helsinki and the Department of Health and Human Services Belmont Report.	Yes	Methods
Studies involving **human participants: For publication of patient photos**, include a statement confirming that consent to publish was obtained.	Not Applicable	
Studies involving experimental **animals**: State details of **authority granting ethics approval** (IRB or equivalent committee(s), provide reference number for approval. Include a statement of compliance with ethical regulations.	Yes	Methods
Studies involving **specimen and field samples:** State if relevant **permits** obtained, provide details of authority approving study; if none were required, explain why.	Not Applicable	

Dual Use Research of Concern (DURC)	Information included in the manuscript?	In which section is the information available? (Reagents and Tools Table, Materials and Methods, Figures, Data Availability Section)
Could your study fall under dual use research restrictions? Please check biosecurity documents and list of **select agents and toxins** (CDC): https://www.selectagents.gov/sat/list.htm	Not Applicable	
If you used a select agent, is the security level of the lab appropriate and reported in the manuscript?	Not Applicable	
If a study is subject to dual use research of concern regulations, is the name of the **authority granting approval and reference number** for the regulatory approval provided in the manuscript?	Not Applicable	

Reporting

The MDAR framework recommends adoption of discipline-specific guidelines, established and endorsed through community initiatives. Journals have their own policy about requiring specific guidelines and recommendations to complement MDAR.

Adherence to community standards	Information included in the manuscript?	In which section is the information available? (Reagents and Tools Table, Materials and Methods, Figures, Data Availability Section)
State if relevant guidelines or checklists (e.g., **ICMJE, MIBBI, ARRIVE, PRISMA**) have been followed or provided.	Not Applicable	
For **tumor marker prognostic studies,** we recommend that you follow the **REMARK** reporting guidelines (see link list at top right). See author guidelines, under 'Reporting Guidelines'. Please confirm you have followed these guidelines.	Not Applicable	
For **phase II and III randomized controlled trials**, please refer to the **CONSORT** flow diagram (see link list at top right) and submit the CONSORT checklist (see link list at top right) with your submission. See author guidelines, under 'Reporting Guidelines'. Please confirm you have submitted this list.	Not Applicable	

Data Availability

Data availability	Information included in the manuscript?	In which section is the information available? (Reagents and Tools Table, Materials and Methods, Figures, Data Availability Section)
Have **primary datasets** been deposited according to the journal's guidelines (see 'Data Deposition' section) and the respective accession numbers provided in the Data Availability Section?	Yes	Data availability
Were **human clinical and genomic datasets** deposited in a public access-controlled repository in accordance to ethical obligations to the patients and to the applicable consent agreement?	Not Applicable	
Are **computational models** that are central and integral to a study available without restrictions in a machine-readable form? Were the relevant accession numbers or links provided?	Not Applicable	
If publicly available data were reused, provide the respective **data citations in the reference list**.	Yes	References
